# Supplementary material for: N-terminal α-amino SUMOylation of cofilin-1 is critical for its regulation of actin depolymerization
Source: Nat Commun. 2023 Sep 14;14:5688. doi: 10.1038/s41467-023-41520-2 (PMC10502023; doi:10.1038/s41467-023-41520-2)
Supplement: Supplementary file 1 — Supplementary Information [file 41467_2023_41520_MOESM1_ESM.pdf]

SUPPLEMENTARY FIGURES AND LEGENDS

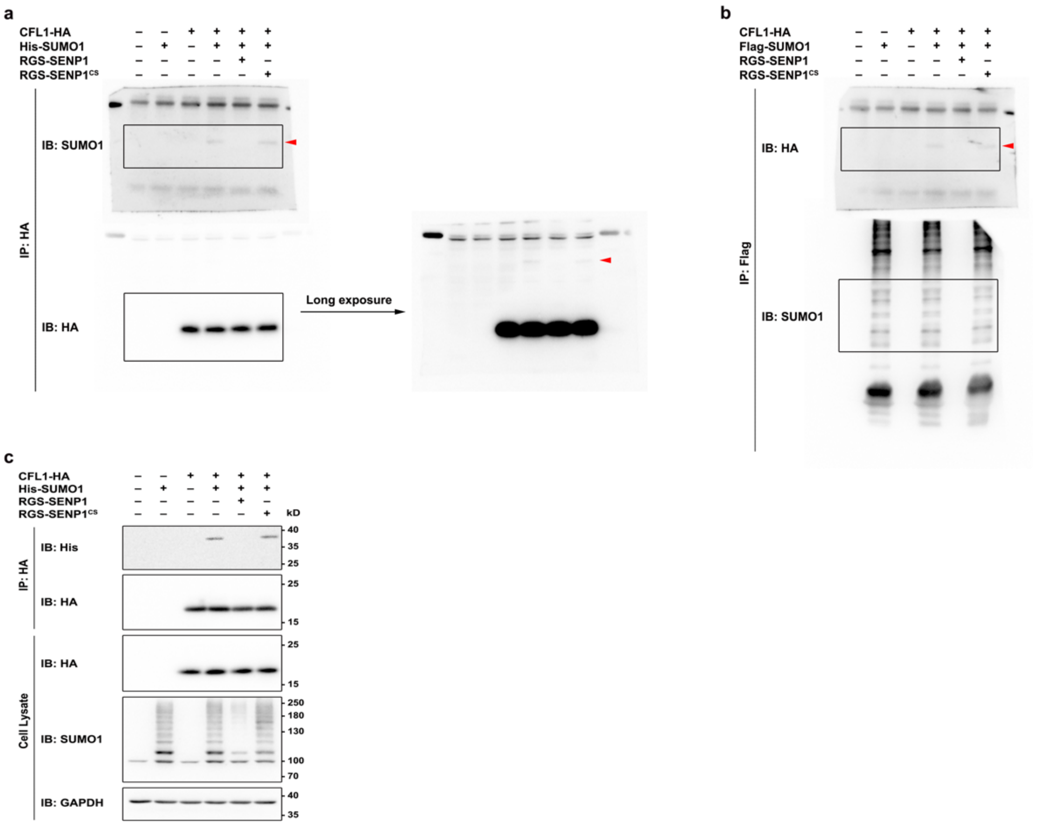

Supplementary Figure 1. CFL1 is modified by SUMO1. a, b Uncropped Western blot images of Fig. 1a, b. Boxed areas are shown in Fig. 1a, b. For (**a**) IB: HA, a long exposure image is shown at right to reveal the SUMOylated CFL1 (red arrowhead). **c** SUMOylated CFL1 can be detected by anti-His antibody. Lysates from CHO-K1 cells transiently transfected with empty vector (−), CFL1-HA, His-SUMO1, RGS-SENP1, and RGS-SENP1^{CS} at various combinations as indicated for 24 hours were subjected to De-IP with the anti-HA antibody, which was followed by IB using anti-His and anti-HA antibodies. The original lysates were analyzed by IB with anti-HA and anti-SUMO1 antibodies for input, and anti-GAPDH antibody for loading control. The blot was performed once.

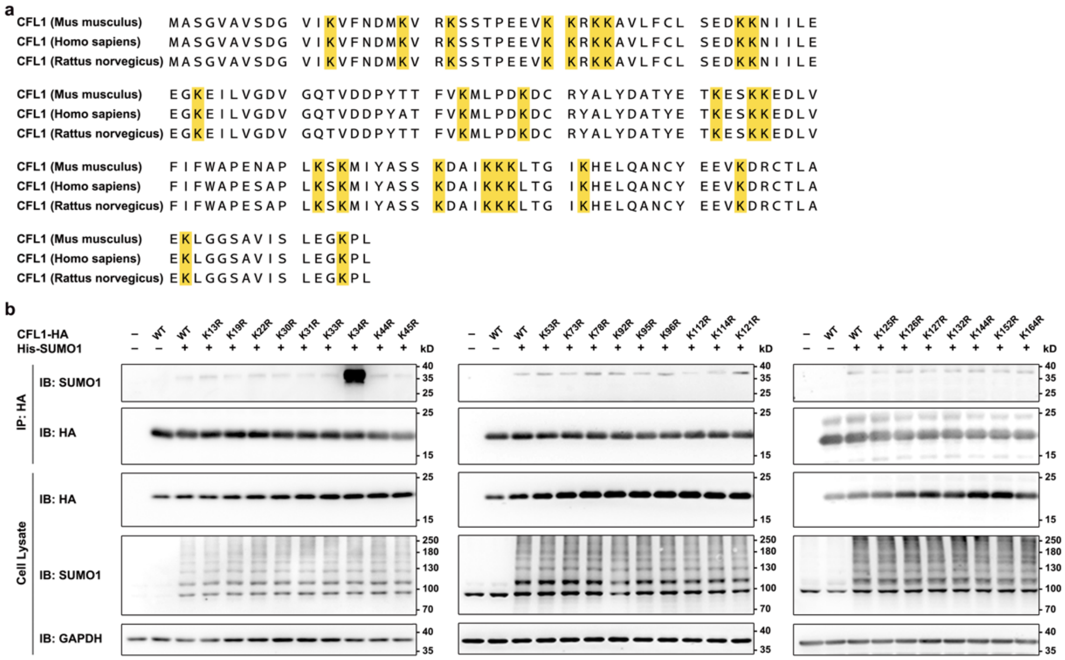

Supplementary Figure 2. Substitutions of individual lysines of CFL1 with arginines did not eliminate the conjugation by SUMO1. a Alignment of CFL1 proteins from mouse (Mus musculus), human (Homo sapiens) and rat (Rattus norvegicus). The 25 internal lysines (all conserved) are indicated in yellow. **b** K → R mutations of CFL1 did not eliminate SUMOylation. CHO-K1 cells were transiently transfected with the vector control (−) or CFL1-HA WT or one of the K → R mutants of CFL1-HA as indicated. His-SUMO1 was included as indicated to enhance SUMO conjugation. At 24 hours post transfection cell lysates were made and subjected to De-IP and IB as in Fig. 1a. All blots are representatives of at least three independent experiments.

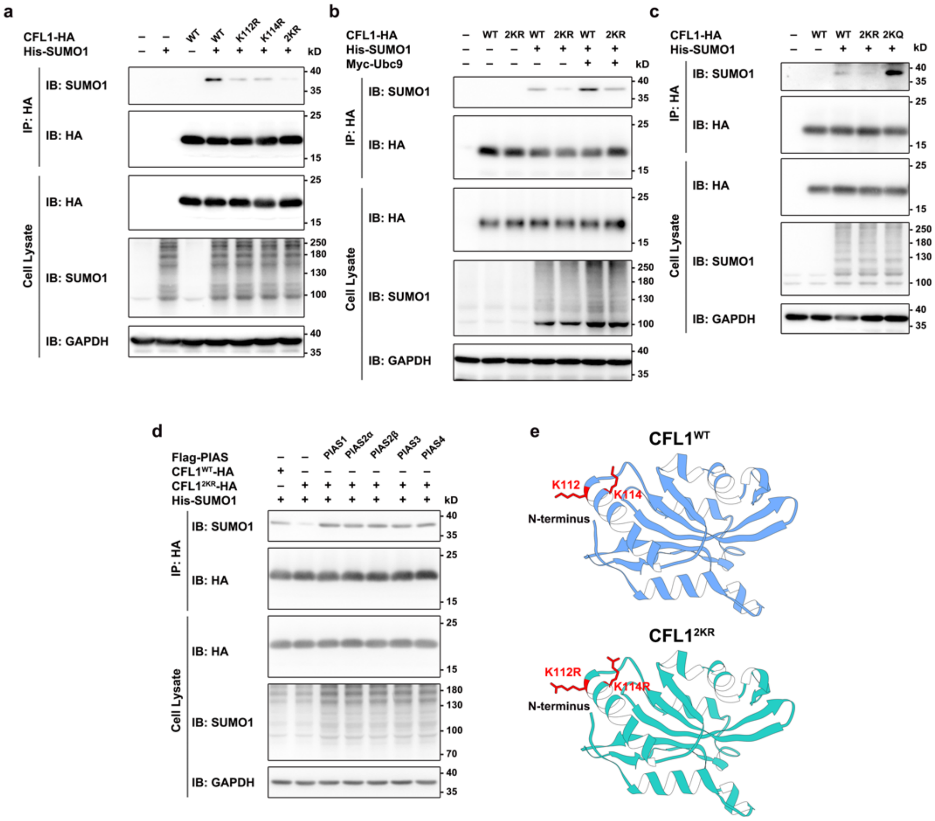

Supplementary Figure 3. Mutations at K112 and K114 alter but do not abolish CFL1 SUMOylation. a CFL1 K112/114R (2KR) mutant exhibited strongly reduced SUMOylation than CFL1 wild type (WT). CHO-K1 cells were transiently transfected with the vector control (−), His-SUMO1, and wild-type (WT) CFL1-HA or K112R, K114R, or 2KR mutant of CFL1-HA as indicated for 24 hours. Cell lysates were subjected to De-IP and IB as in Fig. 1a. **b** Co-expression of Ubc9 increased the level of CFL1^{2KR} SUMOylation. Similar to (**a**) but Myc-Ubc was included in the transfection as indicated. **c** CFL1 K112/114Q (2KQ) mutant exhibited markedly enhanced CFL1 SUMOylation than CFL1 WT. Similar to (**a**) but CFL1^{2KQ}-HA was used in the transfection as indicated. **d** Co-expression of SUMO E3 ligases (PIAS family) increased the level of CFL1^{2KR} SUMOylation. Similar to (**b**) but different PIAS isoforms were used in place of Myc-Ubc9. All blots are representatives of at least three independent experiments. **e** The structures of CFL1^{WT} and CFL1^{2KR}

predicted by ColabFold. The N-terminus, lysine 112 (K112), lysine 114 (K114), as well as the arginine substitutions in the 2KR mutant are indicated.

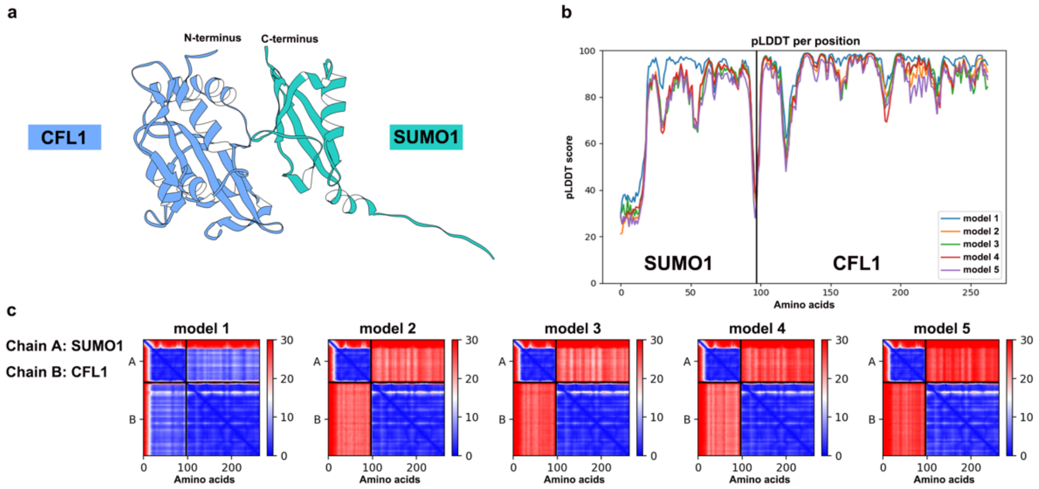

Supplementary Figure 4. The spatial relationship between CFL1 and SUMO1 predicted by ColabFold. a The structure and spatial relationship of wild-type mouse CFL1 and SUMO1 predicted by ColabFold. **b** The predicted local difference distance test (pLDDT) scores of 5 modeled CFL1 and SUMO1 structures. **c** The predicted assigned error (PAE) maps of the 5 modeled CFL1 and SUMO1 structures. The PAE values are indicated in the colored bars to the right of each map. Source data are provided as Source Data file.

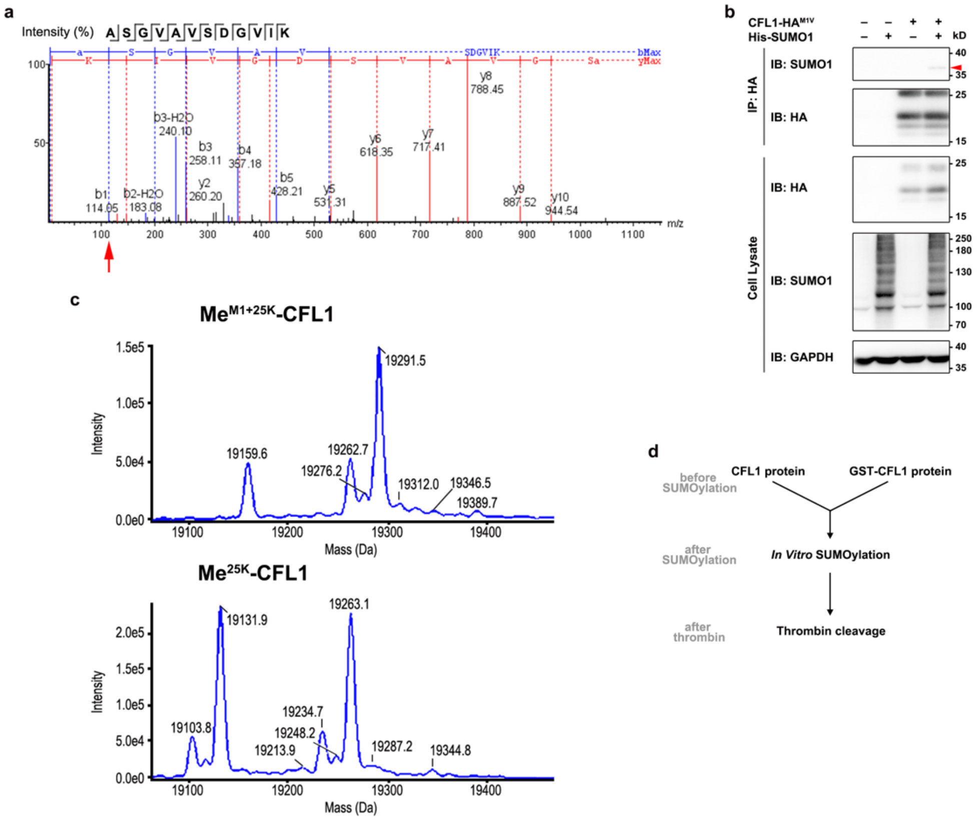

Supplementary Figure 5. The free N-terminus α-amino group is required for SUMO1 conjugation of CFL1, related to Fig. 2. a MS/MS spectrum of a CFL1 peptide showing acetylation at the N-terminus based on collision-activated dissociation (CAD). Endogenous CFL1 proteins were purified from Neuro-2a cells that over-expressed Myc-Naa60 by De-IP with the anti-CFL1 antibody. The products were digested with trypsin and subjected to LC-MS/MS analysis. The arrow indicates the 42 Da increase on the alanine (A) at the N-terminus due to covalent attachment of an acetyl group after the initial methionine was cleaved, demonstrating that the N-terminal α-NH_2 group of CFL1 was protected by acetylation. **b** SUMO1 is conjugated to the N-terminal α-NH_2 group of CFL1 independently of the initial methionine. CHO-K1 cells transiently transfected with the vector control (−), M1V mutant of CFL1-HA, and His-SUMO1 as indicated for 24 hours. Lysates were subjected to De-IP and IB as in Fig. 1a. Red arrowhead

indicates SUMOylated CFL1^{M1V}-HA. The blots are representatives of at least three independent experiments. **c** Confirmation of CFL1 methylation. After *in vitro* methylation and SENP2 cleavage of the purified SUMO3-tagged CFL1, the products were subject to LC-MS. Methylation of all lysines and the α-NH_2 group of N-terminal methionine in Me^{M1+25K}-CFL1 protein gave rise to the peak at 19291.5 Da with a mass shift of 731.9 Da compared to theoretical molecular weight of CFL1 (18559.6 Da). Methylation of all lysines in Me^{25K}-CFL1 protein gave rise to the peak at 19263.1 Da with a mass shift of 703.5 Da compared to theoretical molecular weight of CFL1. **d** Diagram for the workflow of determining whether the internal lysines of CFL1 can be SUMOylated *in vitro*. Purified wild-type CFL1 and N-terminal GST-tagged CFL1 (GST-CFL1) proteins were SUMOylated *in vitro* first and then digested by thrombin to cleave the N-terminal GST-tag. Source data are provided as Source Data file.

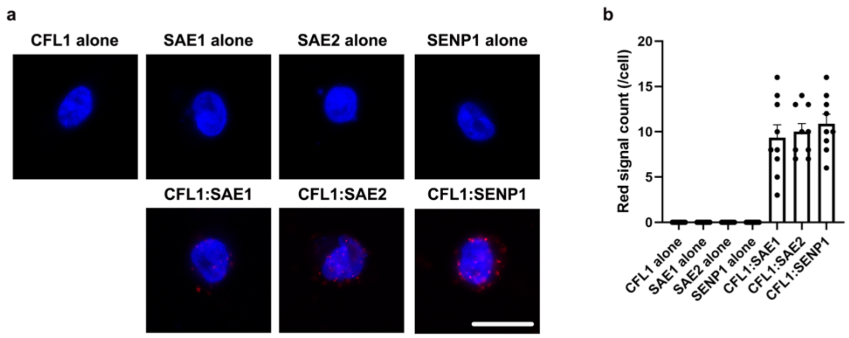

Supplementary Figure 6. CFL1 interacted with E1 enzyme subunits and SENP1. a Proximity ligation assay (PLA) of CFL1 with SAE1, SAE2 and SENP1 in CHO-K1 cells suggests that CFL1 comes into close proximity with the E1 enzyme subunits and SENP1, respectively (red dots). Nuclei were stained with DAPI (blue). Scale bar = 50 μm. **b** The quantification of red signal counts of (**a**). Analysis of red dots counts was done at the original magnification with ImageJ software. n=9, Data correspond to the mean ± SEM of three independent experiments. Source data are provided as Source Data file.

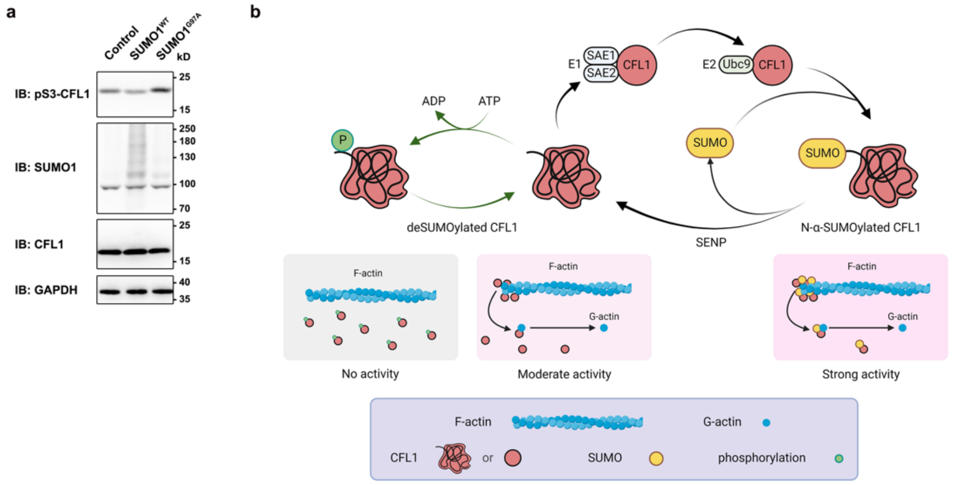

Supplementary Figure 7. N-α-SUMOylation of CFL1 enhances actin depolymerization via both phosphorylation-dependent and -independent mechanisms. a CFL1 SUMOylation suppressed CFL1 phosphorylation at Ser3. Lysates from CHO-K1 cells transiently transfected with empty vector (−), His-SUMO1^{WT} or His-SUMO1^{G97A} for 24 hours were analyzed by IB with anti-pS3-CFL1, anti-SUMO1, and anti-CFL1 antibodies, as well as anti-GAPDH antibody for loading control. The blot is the representative of at least three independent experiments. **b** Schematic model showing that the N-terminus of CFL1 is modified by SUMO1, which directly enhances the binding to F-actin and the disassembly of actin filaments by the non-phosphorylated CFL1. In addition, the N-α-SUMOylation of CFL1 also renders it not phosphorylatable by LIM kinases, leading to an overall reduction of phosphorylated, i.e., inactive CFL1. The diagram is created with BioRender.com.
