# Supplementary figures and images for: N-terminal α-amino SUMOylation of cofilin-1 is critical for its regulation of actin depolymerization (part 2 of 2)
Source: Nat Commun. 2023 Sep 14;14:5688. doi: 10.1038/s41467-023-41520-2 (PMC10502023; doi:10.1038/s41467-023-41520-2)

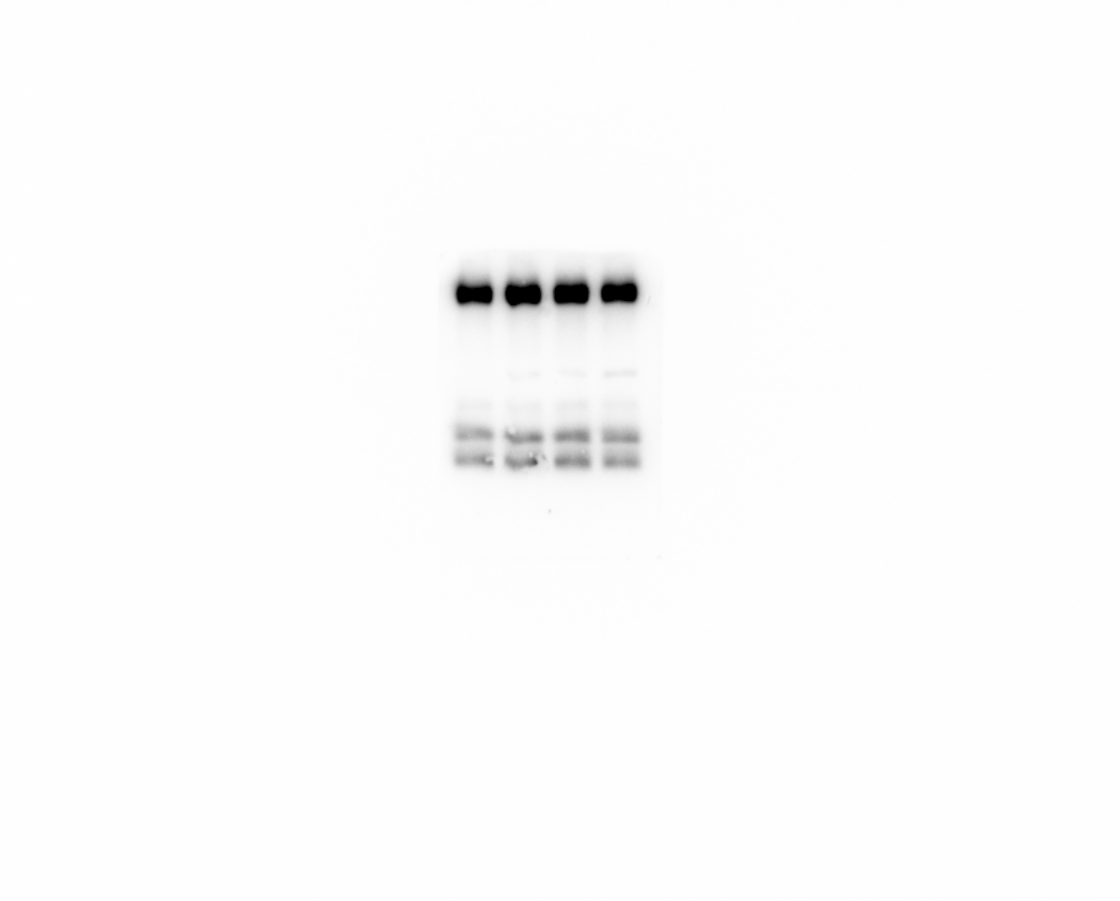

Supplement: Supplementary file 4 — Source Data [file 41467_2023_41520_MOESM4_ESM.zip › Source Data/Uncropped and Unprocessed Scans/Fig. 3d/IP CFL1 (mouse); IB SUMO1 (rabbit).tif]

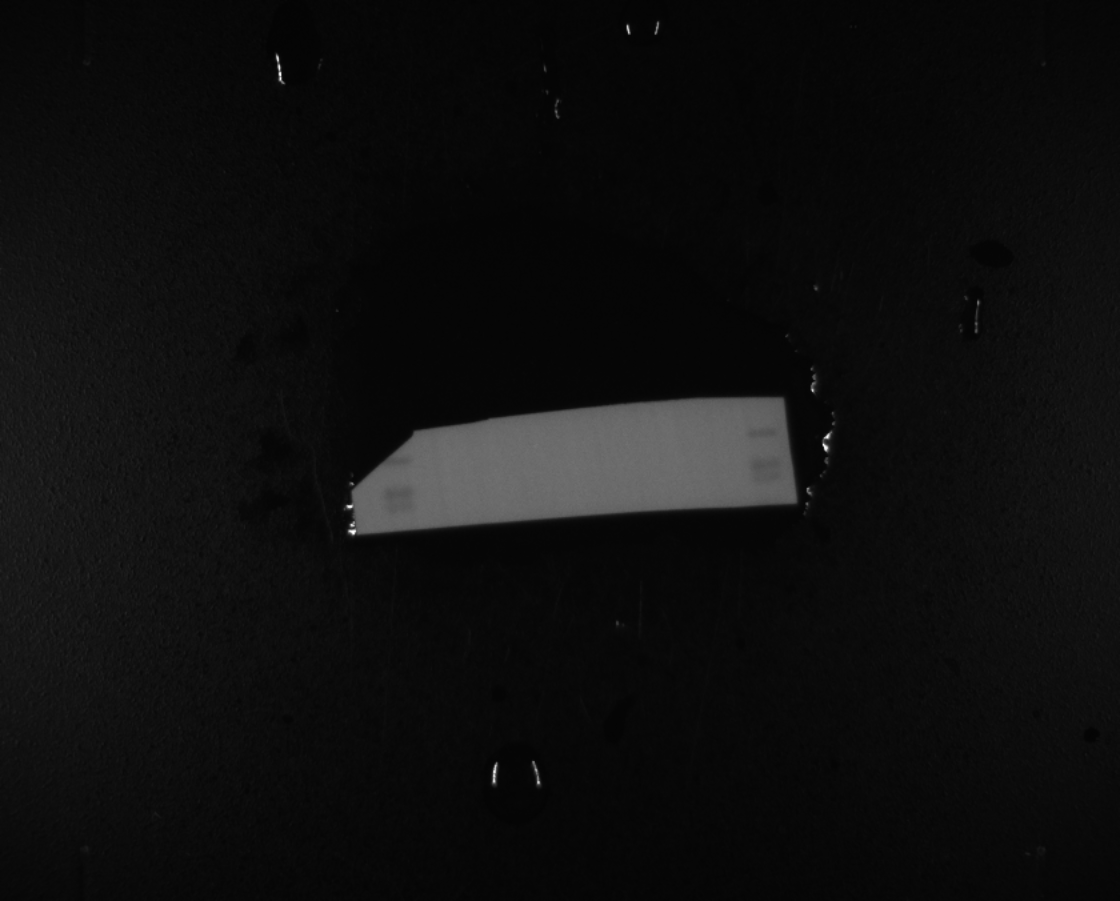

Supplement: Supplementary file 4 — Source Data [file 41467_2023_41520_MOESM4_ESM.zip › Source Data/Uncropped and Unprocessed Scans/Fig. 3e/IB GAPDH - Marker.tif]

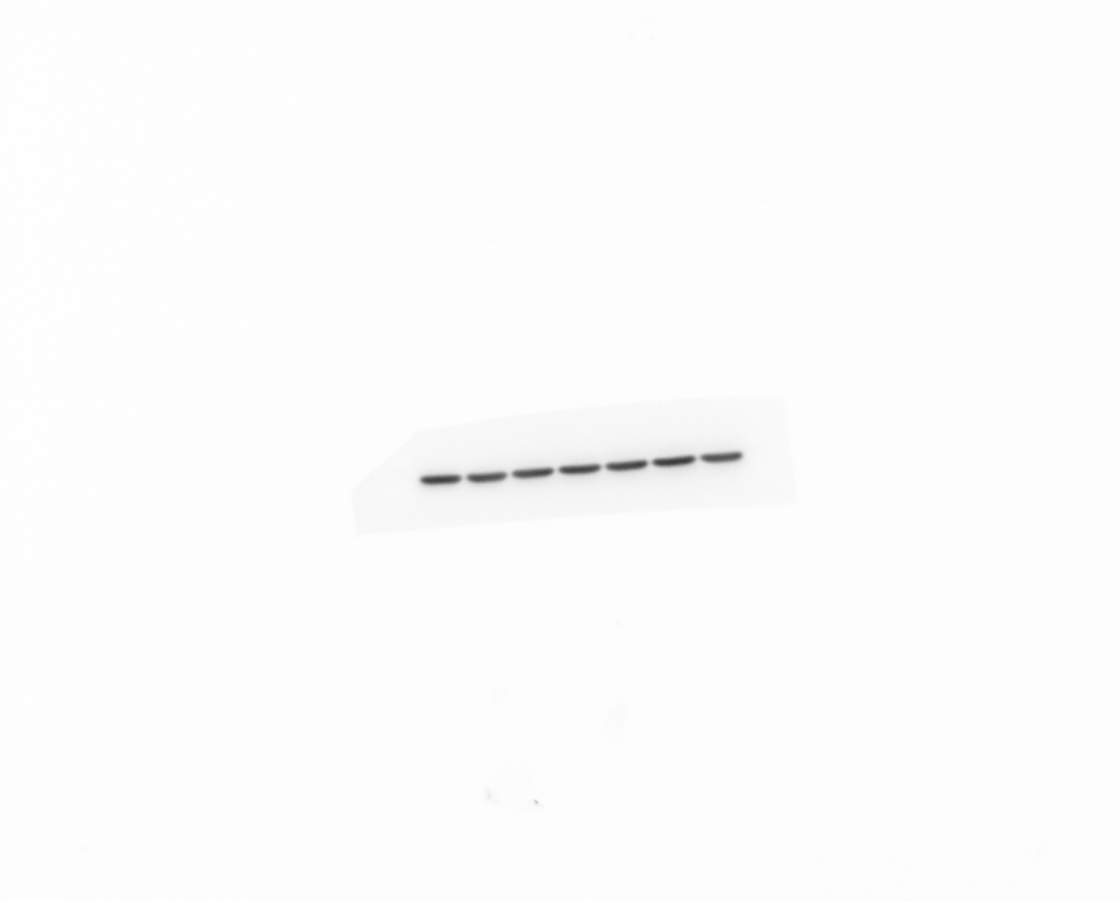

Supplement: Supplementary file 4 — Source Data [file 41467_2023_41520_MOESM4_ESM.zip › Source Data/Uncropped and Unprocessed Scans/Fig. 3e/IB GAPDH.tif]

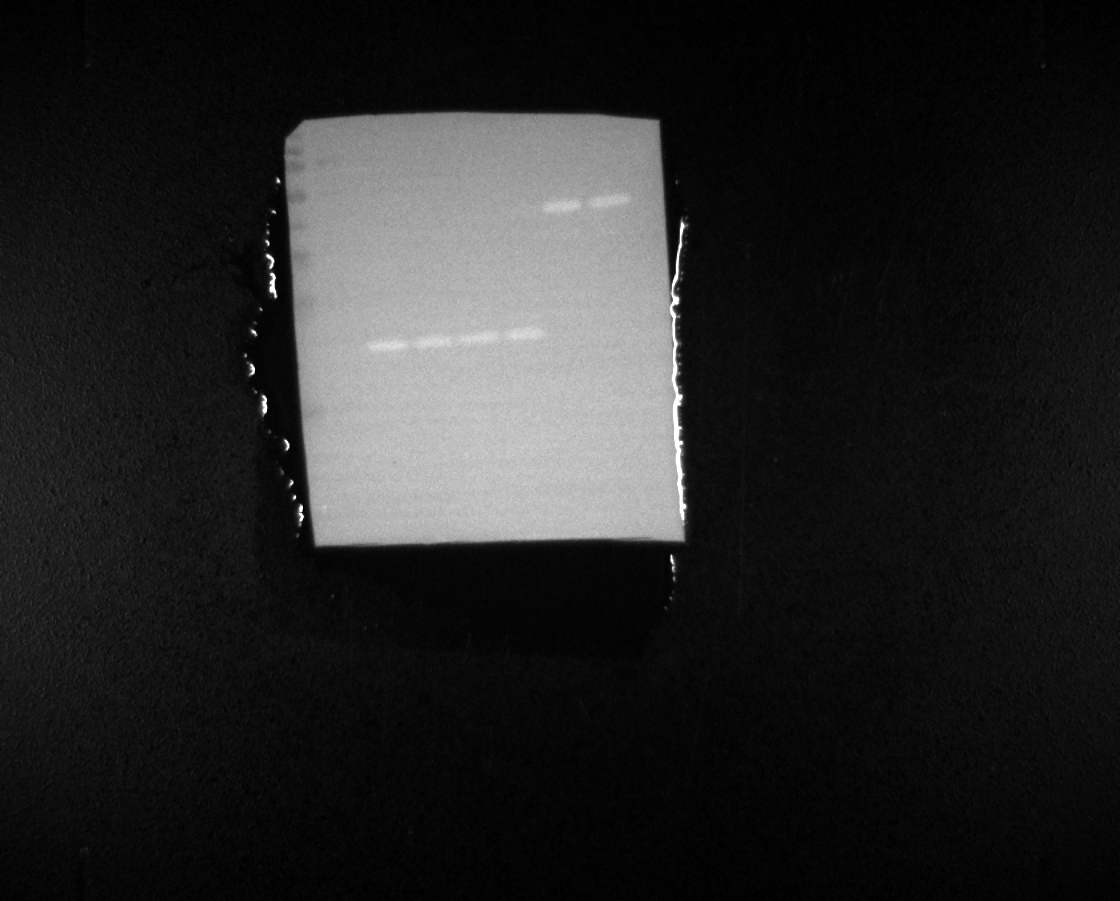

Supplement: Supplementary file 4 — Source Data [file 41467_2023_41520_MOESM4_ESM.zip › Source Data/Uncropped and Unprocessed Scans/Fig. 3e/IB HA - Marker.tif]

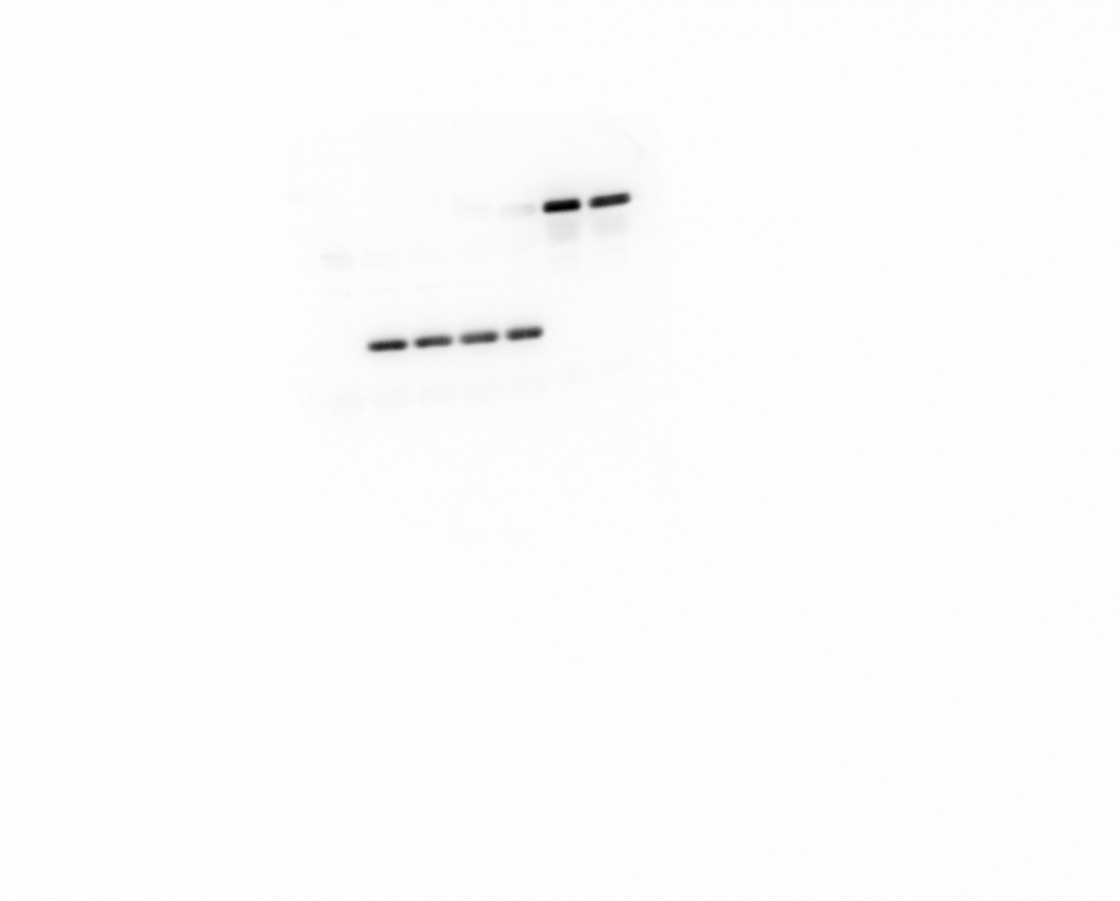

Supplement: Supplementary file 4 — Source Data [file 41467_2023_41520_MOESM4_ESM.zip › Source Data/Uncropped and Unprocessed Scans/Fig. 3e/IB HA.tif]

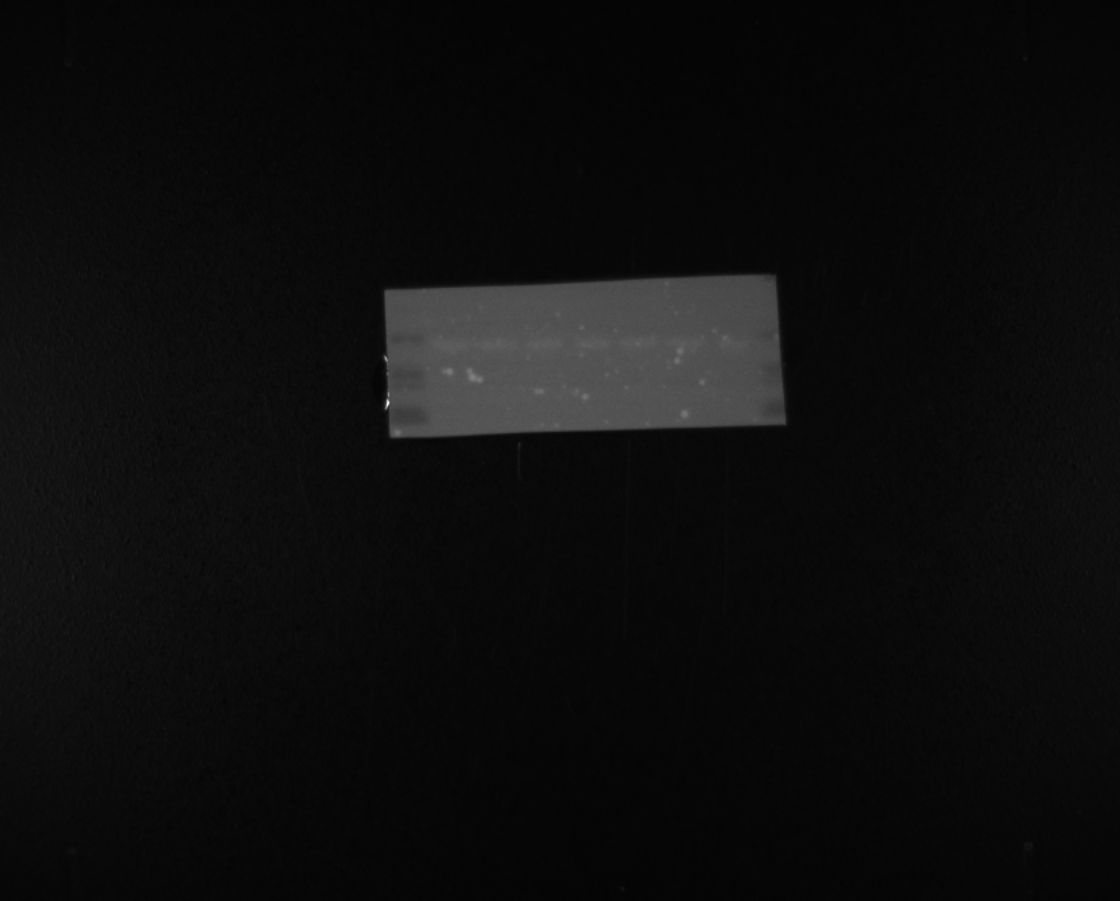

Supplement: Supplementary file 4 — Source Data [file 41467_2023_41520_MOESM4_ESM.zip › Source Data/Uncropped and Unprocessed Scans/Fig. 3e/IB SAE1 - Marker.tif]

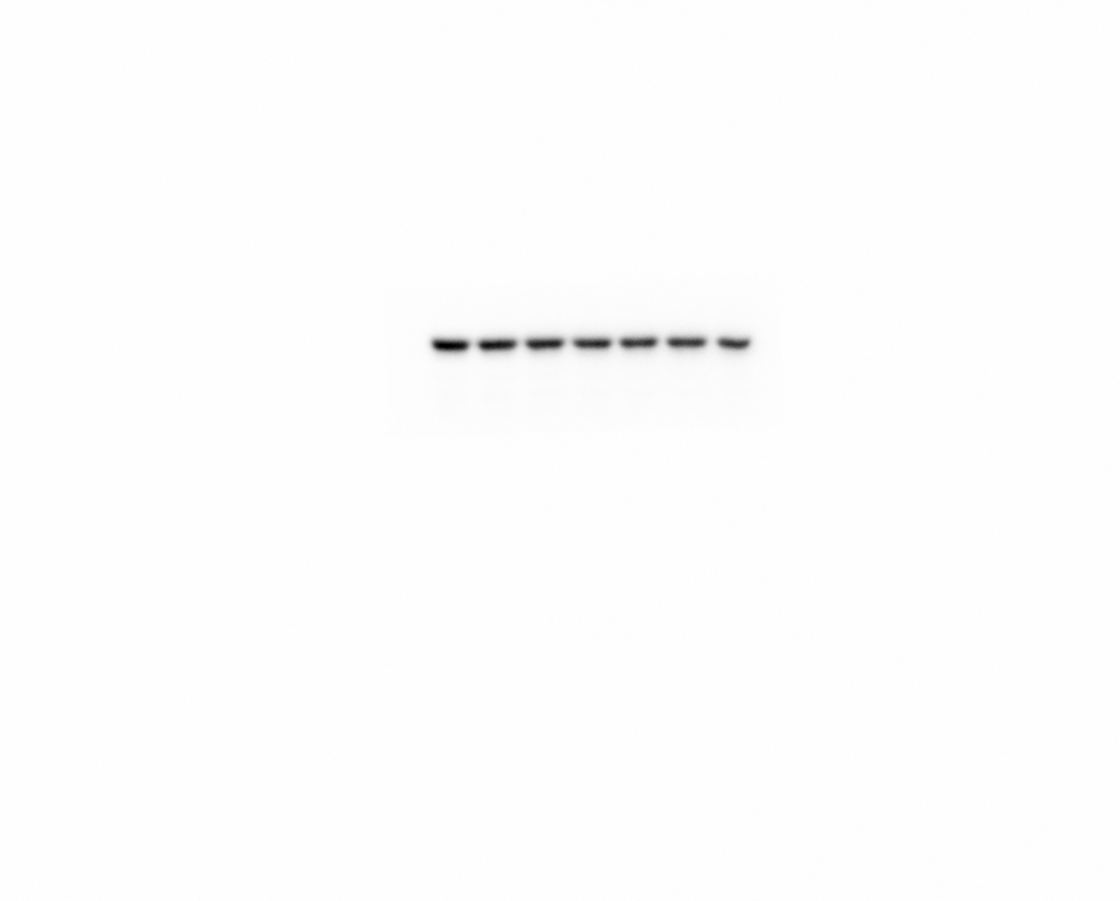

Supplement: Supplementary file 4 — Source Data [file 41467_2023_41520_MOESM4_ESM.zip › Source Data/Uncropped and Unprocessed Scans/Fig. 3e/IB SAE1.tif]

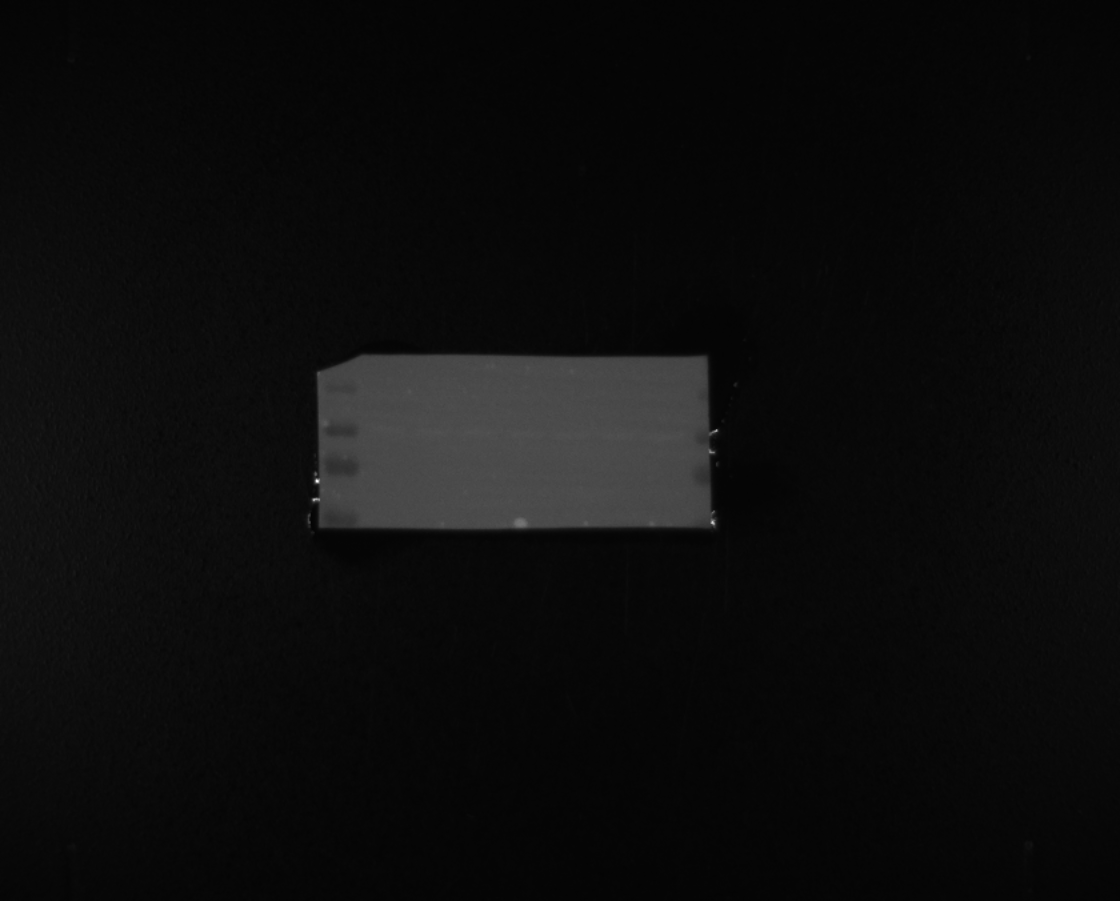

Supplement: Supplementary file 4 — Source Data [file 41467_2023_41520_MOESM4_ESM.zip › Source Data/Uncropped and Unprocessed Scans/Fig. 3e/IB SAE2 - Marker.tif]

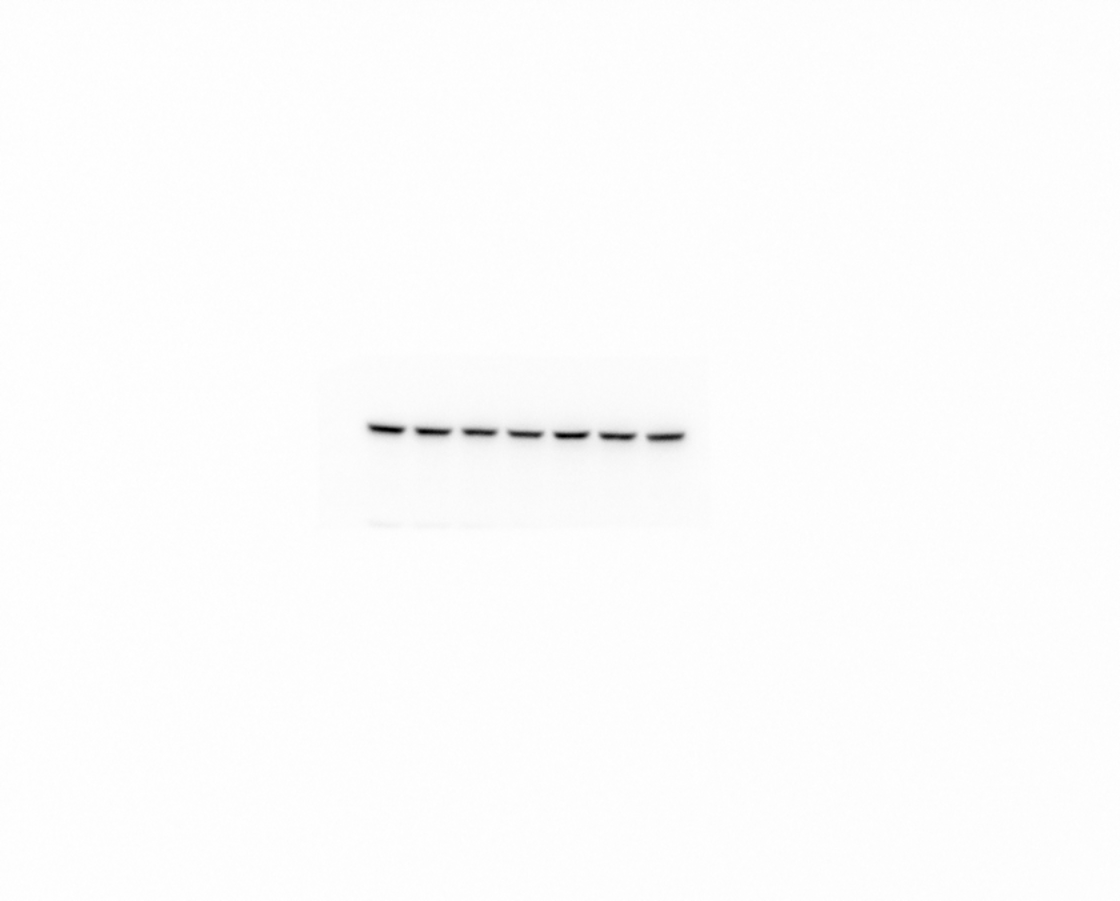

Supplement: Supplementary file 4 — Source Data [file 41467_2023_41520_MOESM4_ESM.zip › Source Data/Uncropped and Unprocessed Scans/Fig. 3e/IB SAE2.tif]

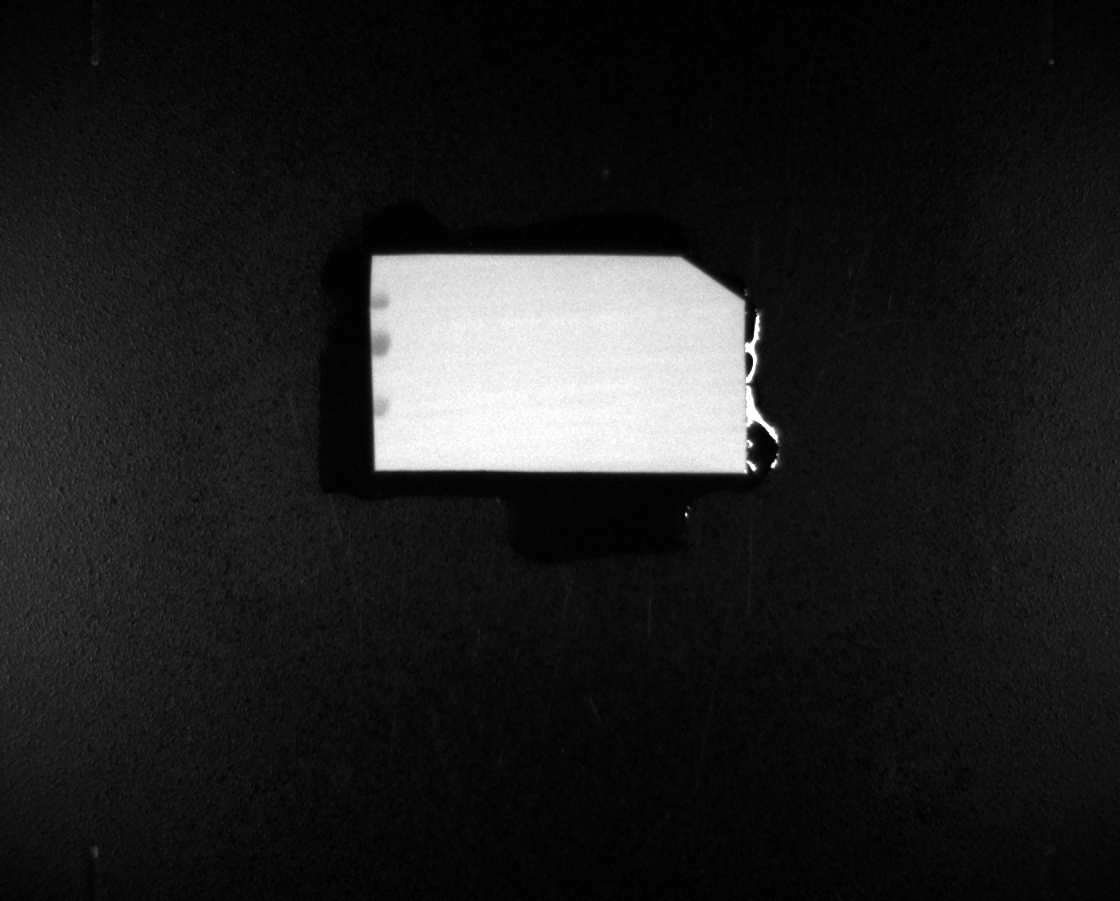

Supplement: Supplementary file 4 — Source Data [file 41467_2023_41520_MOESM4_ESM.zip › Source Data/Uncropped and Unprocessed Scans/Fig. 3e/IB SENP1 - Marker.tif]

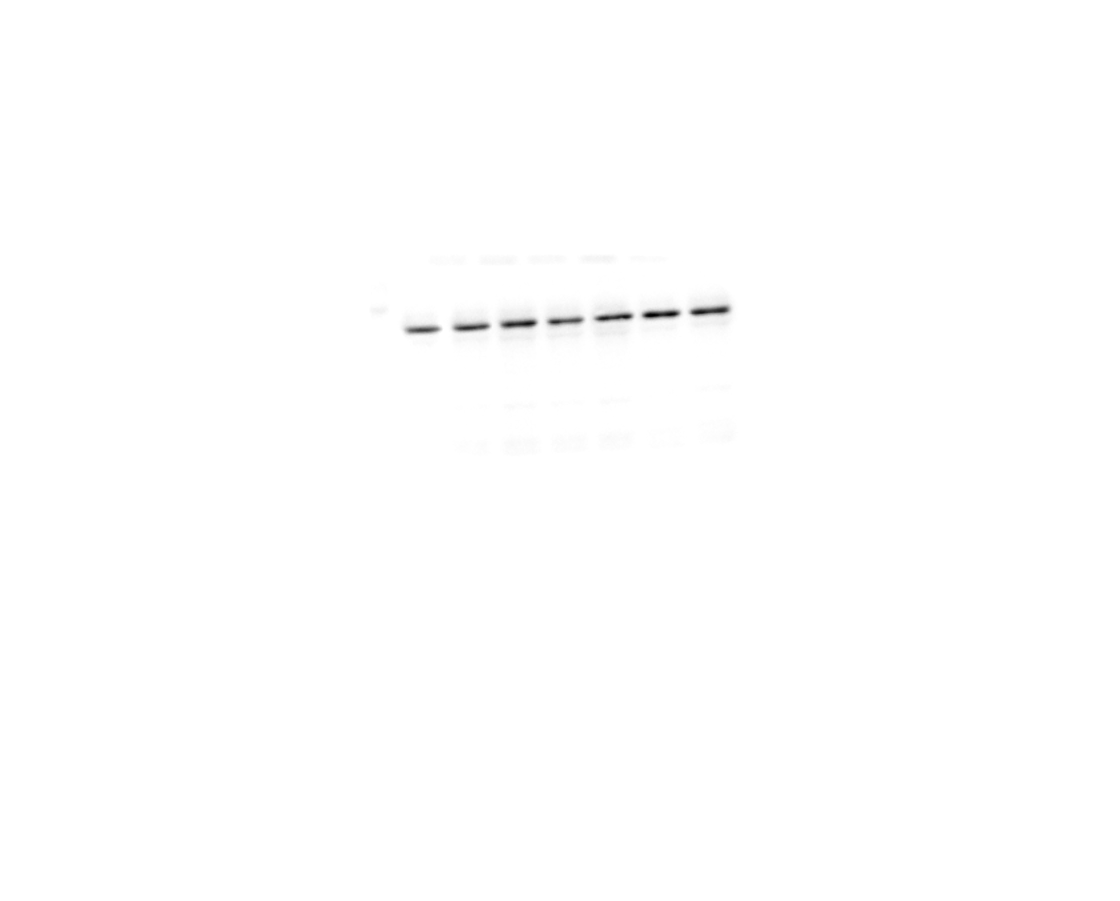

Supplement: Supplementary file 4 — Source Data [file 41467_2023_41520_MOESM4_ESM.zip › Source Data/Uncropped and Unprocessed Scans/Fig. 3e/IB SENP1.tif]

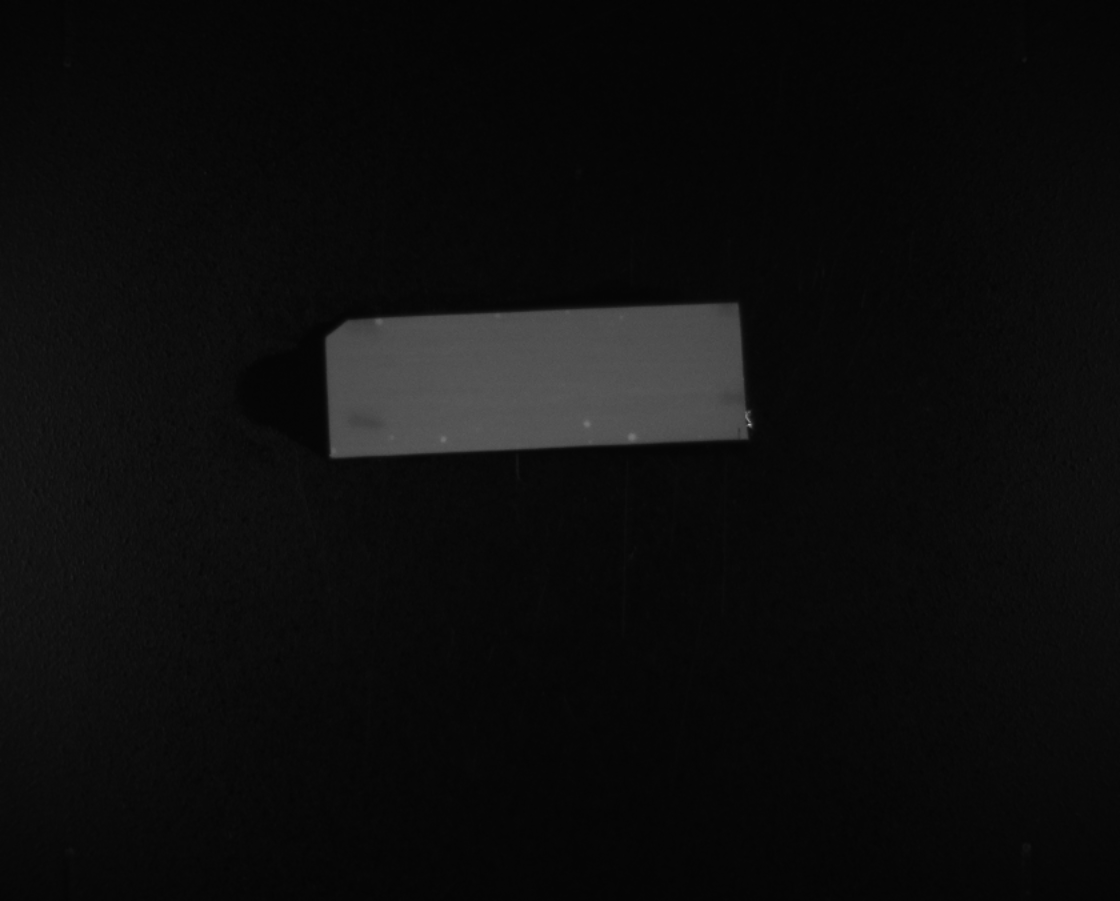

Supplement: Supplementary file 4 — Source Data [file 41467_2023_41520_MOESM4_ESM.zip › Source Data/Uncropped and Unprocessed Scans/Fig. 3e/IB Ubc9 - Marker.tif]

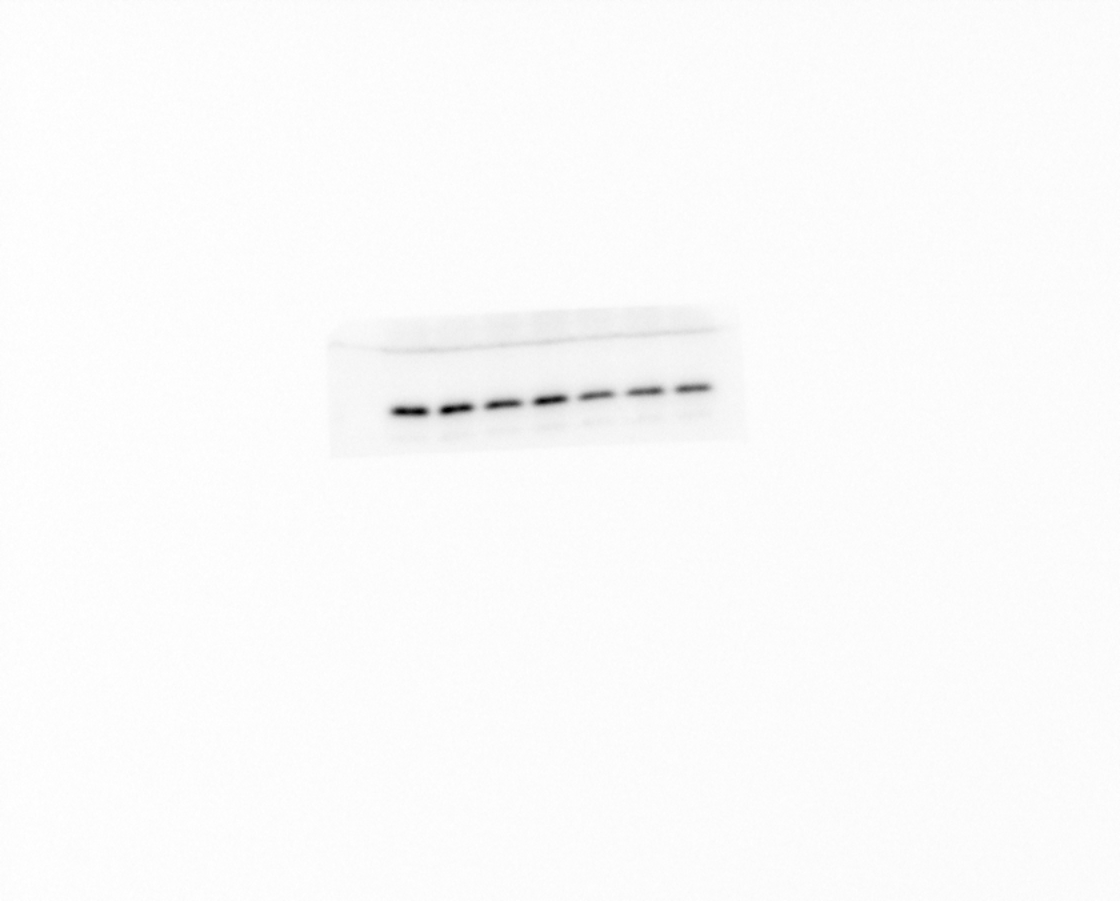

Supplement: Supplementary file 4 — Source Data [file 41467_2023_41520_MOESM4_ESM.zip › Source Data/Uncropped and Unprocessed Scans/Fig. 3e/IB Ubc9.tif]

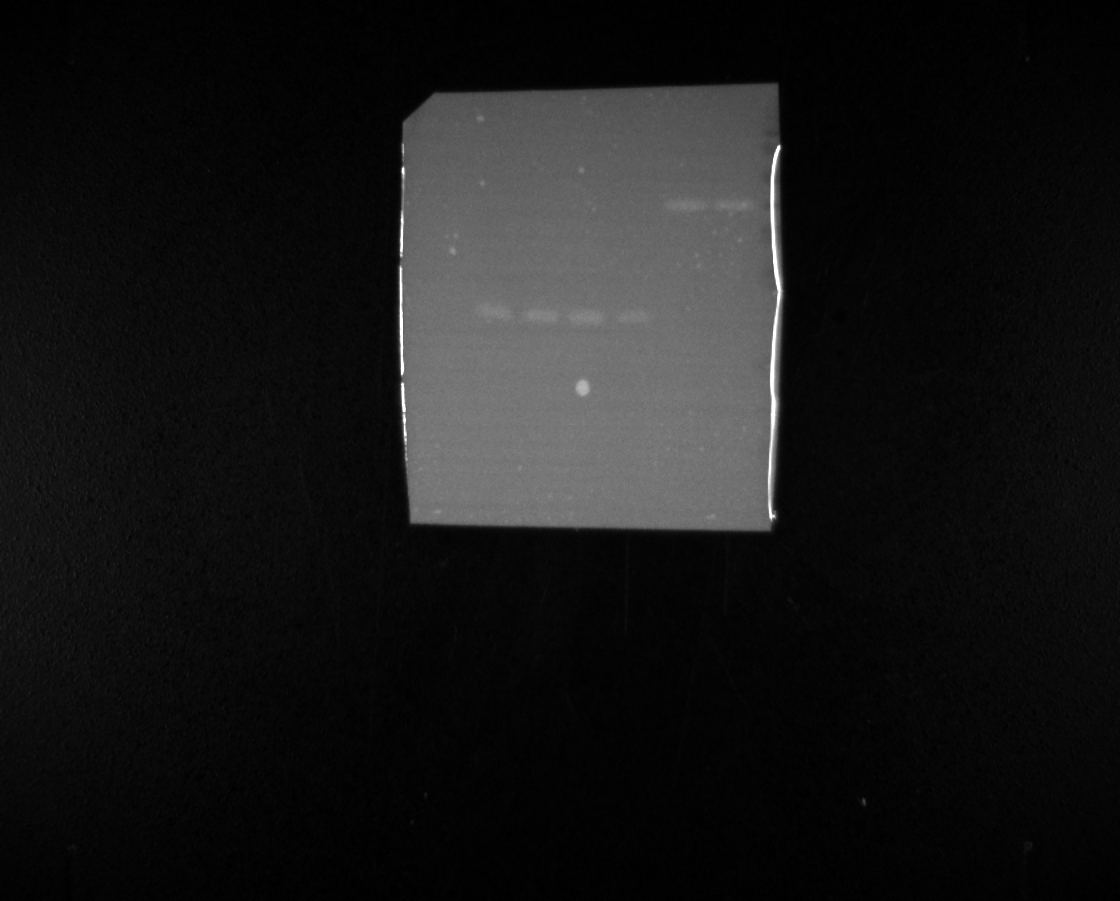

Supplement: Supplementary file 4 — Source Data [file 41467_2023_41520_MOESM4_ESM.zip › Source Data/Uncropped and Unprocessed Scans/Fig. 3e/IP HA; IB HA - Marker.tif]

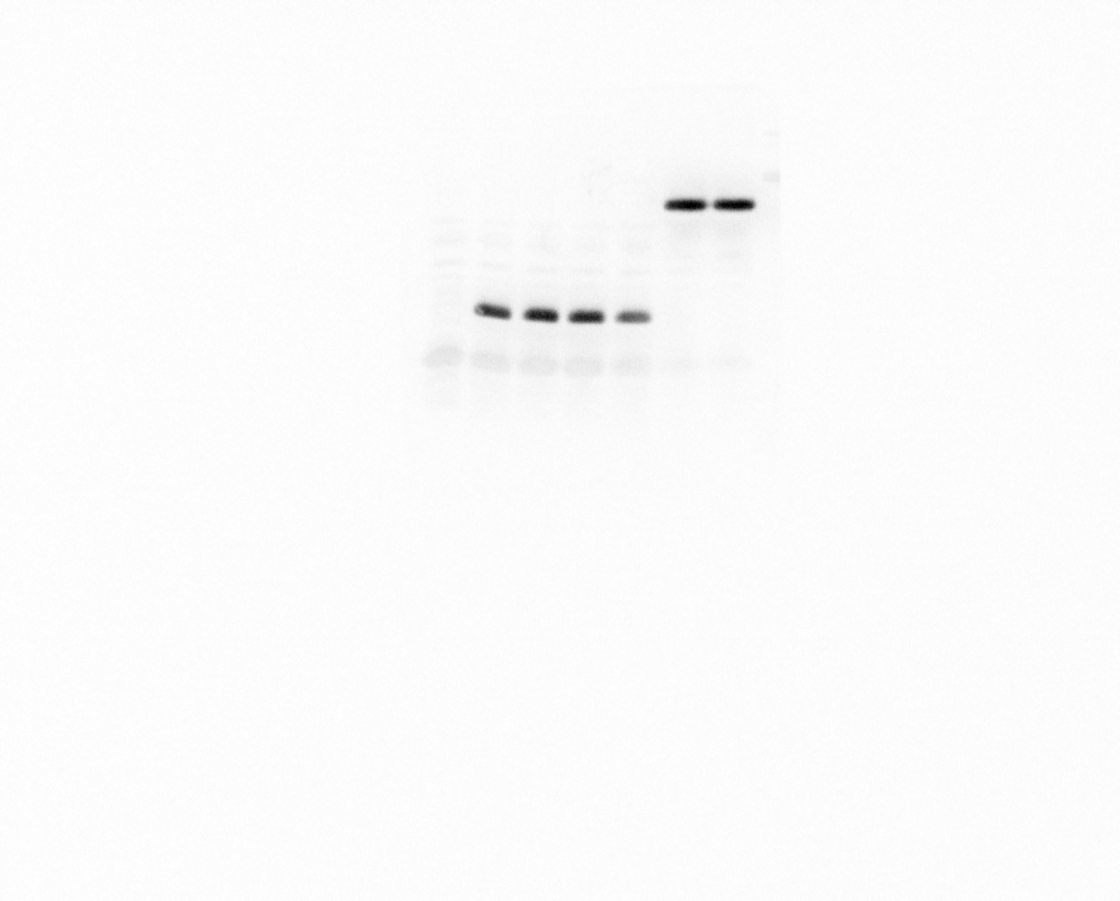

Supplement: Supplementary file 4 — Source Data [file 41467_2023_41520_MOESM4_ESM.zip › Source Data/Uncropped and Unprocessed Scans/Fig. 3e/IP HA; IB HA.tif]

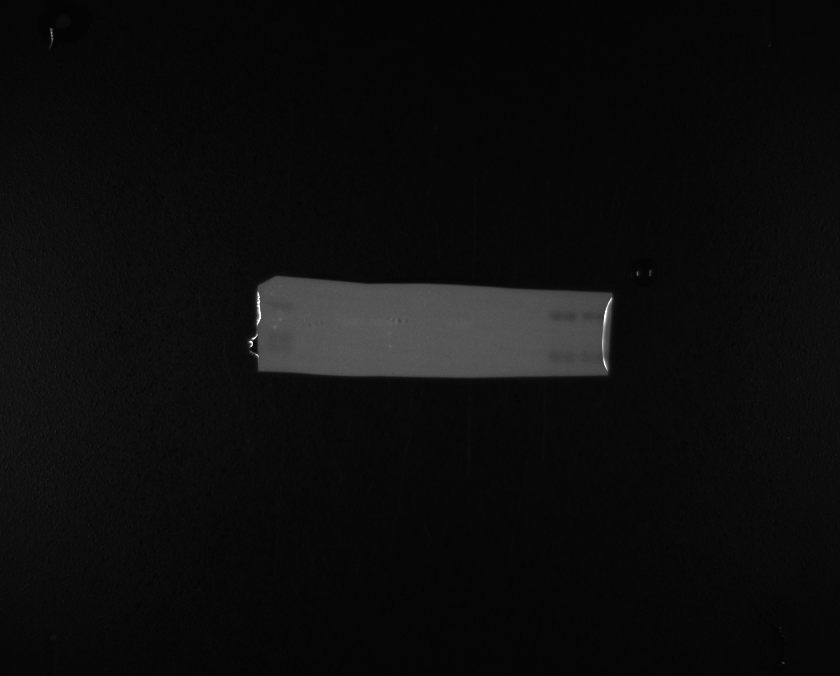

Supplement: Supplementary file 4 — Source Data [file 41467_2023_41520_MOESM4_ESM.zip › Source Data/Uncropped and Unprocessed Scans/Fig. 3e/IP HA; IB SAE1 - Marker.tif]

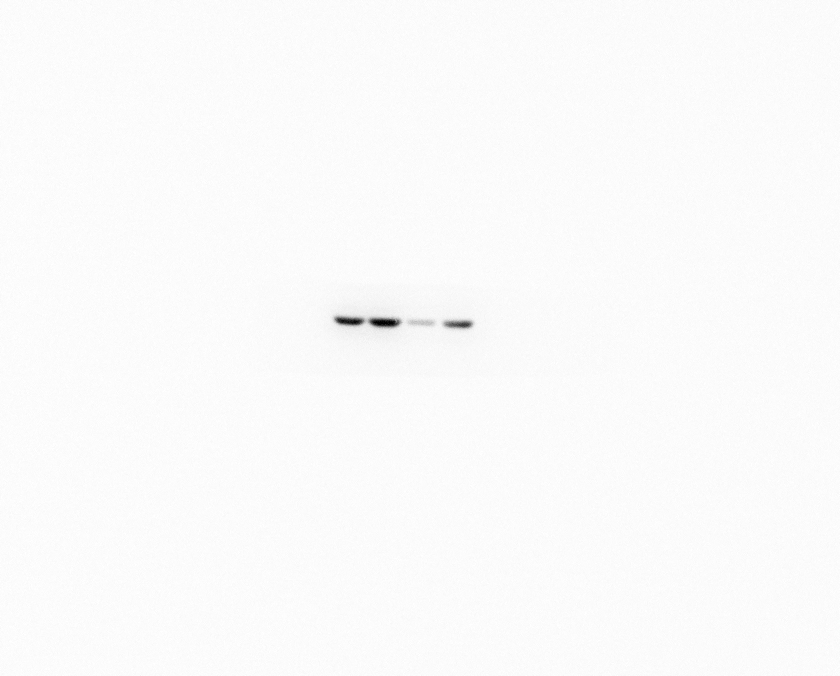

Supplement: Supplementary file 4 — Source Data [file 41467_2023_41520_MOESM4_ESM.zip › Source Data/Uncropped and Unprocessed Scans/Fig. 3e/IP HA; IB SAE1.tif]

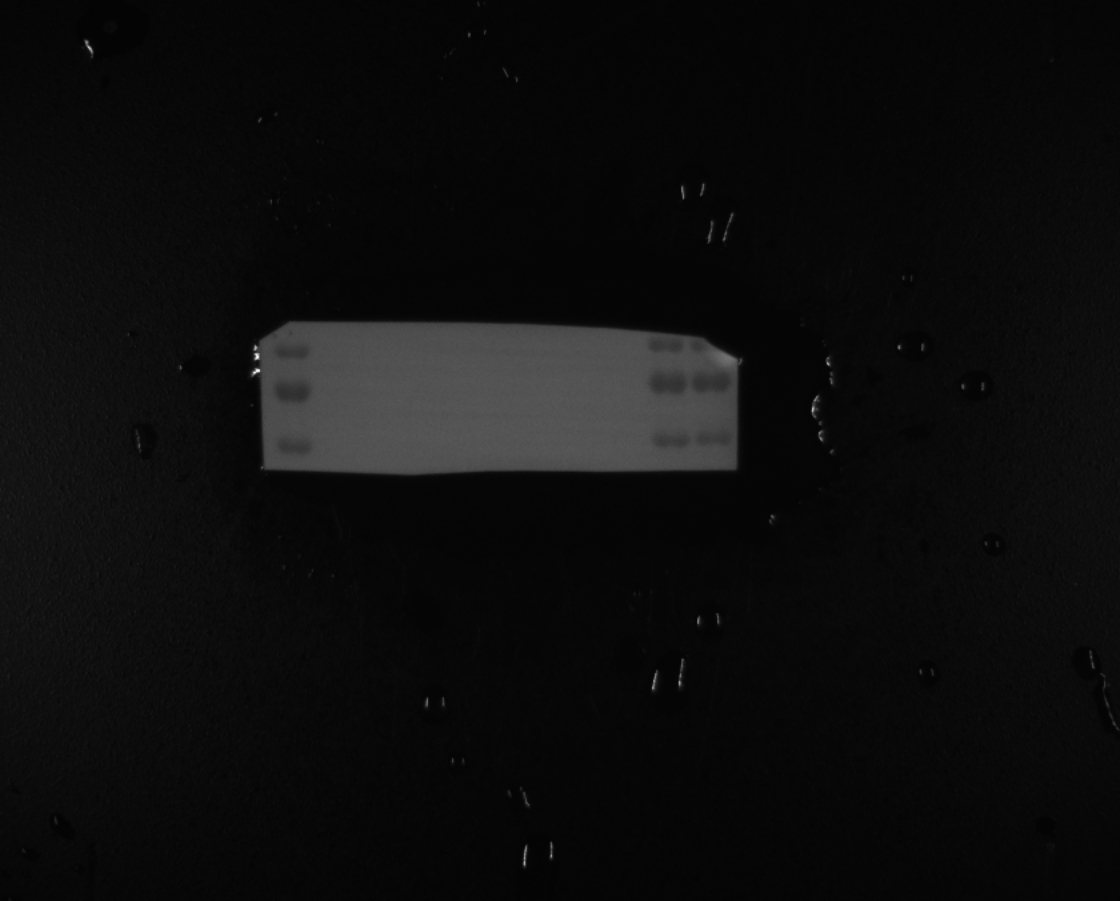

Supplement: Supplementary file 4 — Source Data [file 41467_2023_41520_MOESM4_ESM.zip › Source Data/Uncropped and Unprocessed Scans/Fig. 3e/IP HA; IB SAE2 - Marker.tif]

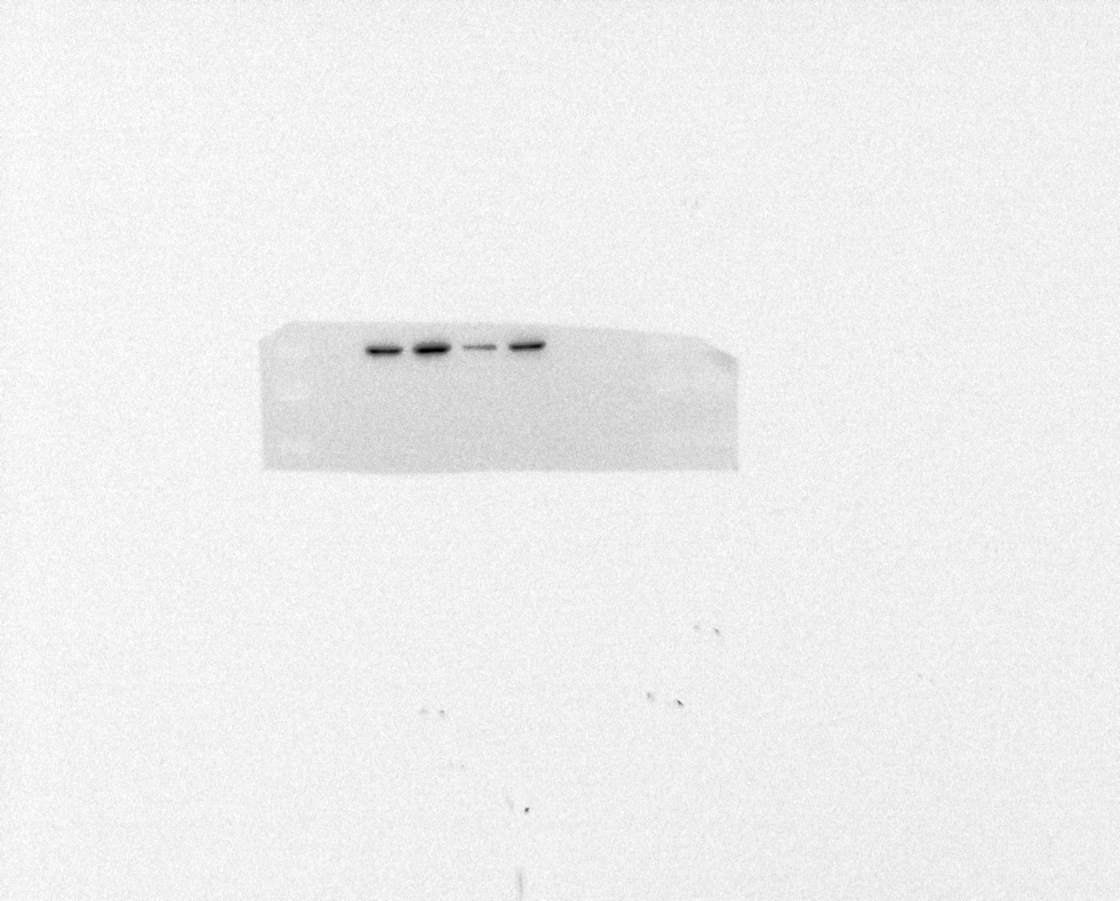

Supplement: Supplementary file 4 — Source Data [file 41467_2023_41520_MOESM4_ESM.zip › Source Data/Uncropped and Unprocessed Scans/Fig. 3e/IP HA; IB SAE2.tif]

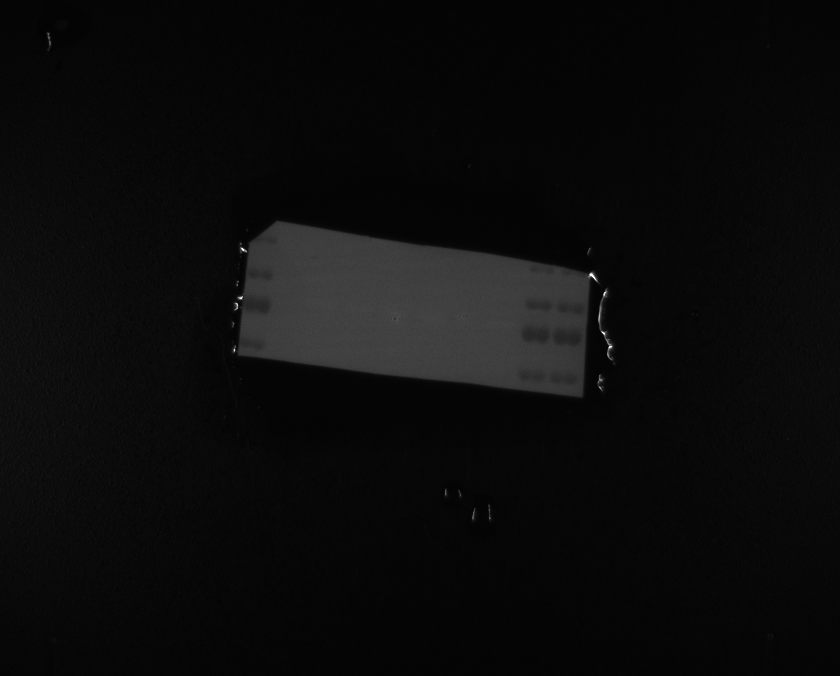

Supplement: Supplementary file 4 — Source Data [file 41467_2023_41520_MOESM4_ESM.zip › Source Data/Uncropped and Unprocessed Scans/Fig. 3e/IP HA; IB SENP1 - Marker.tif]

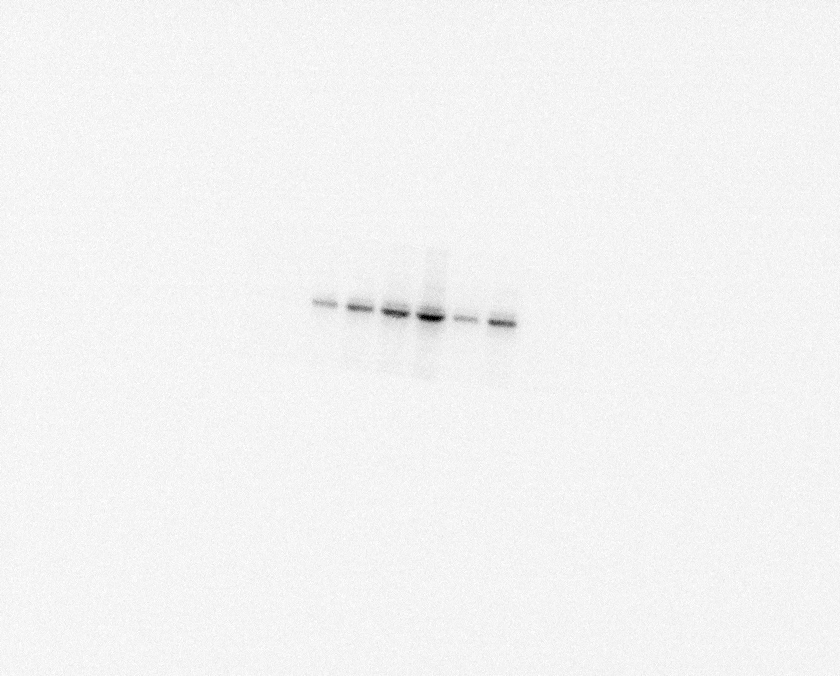

Supplement: Supplementary file 4 — Source Data [file 41467_2023_41520_MOESM4_ESM.zip › Source Data/Uncropped and Unprocessed Scans/Fig. 3e/IP HA; IB SENP1.tif]

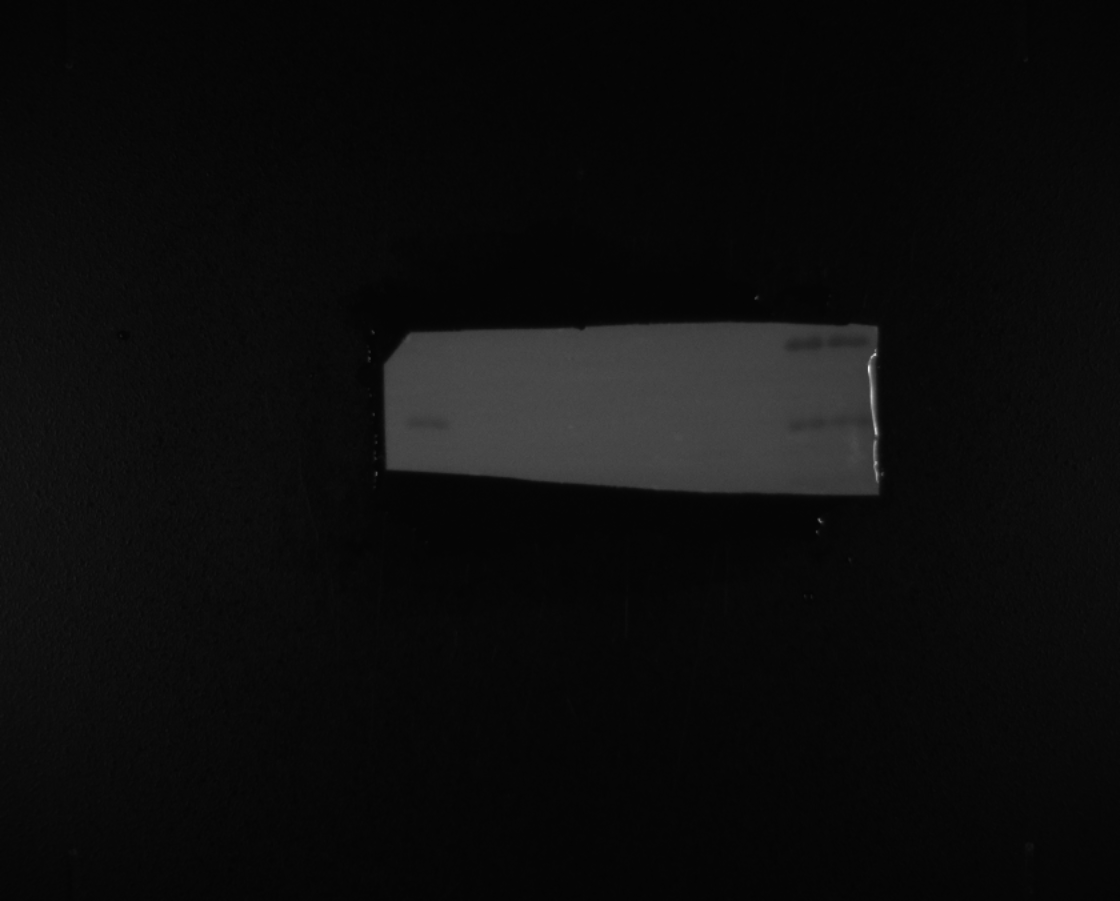

Supplement: Supplementary file 4 — Source Data [file 41467_2023_41520_MOESM4_ESM.zip › Source Data/Uncropped and Unprocessed Scans/Fig. 3e/IP HA; IB Ubc9 - Marker.tif]

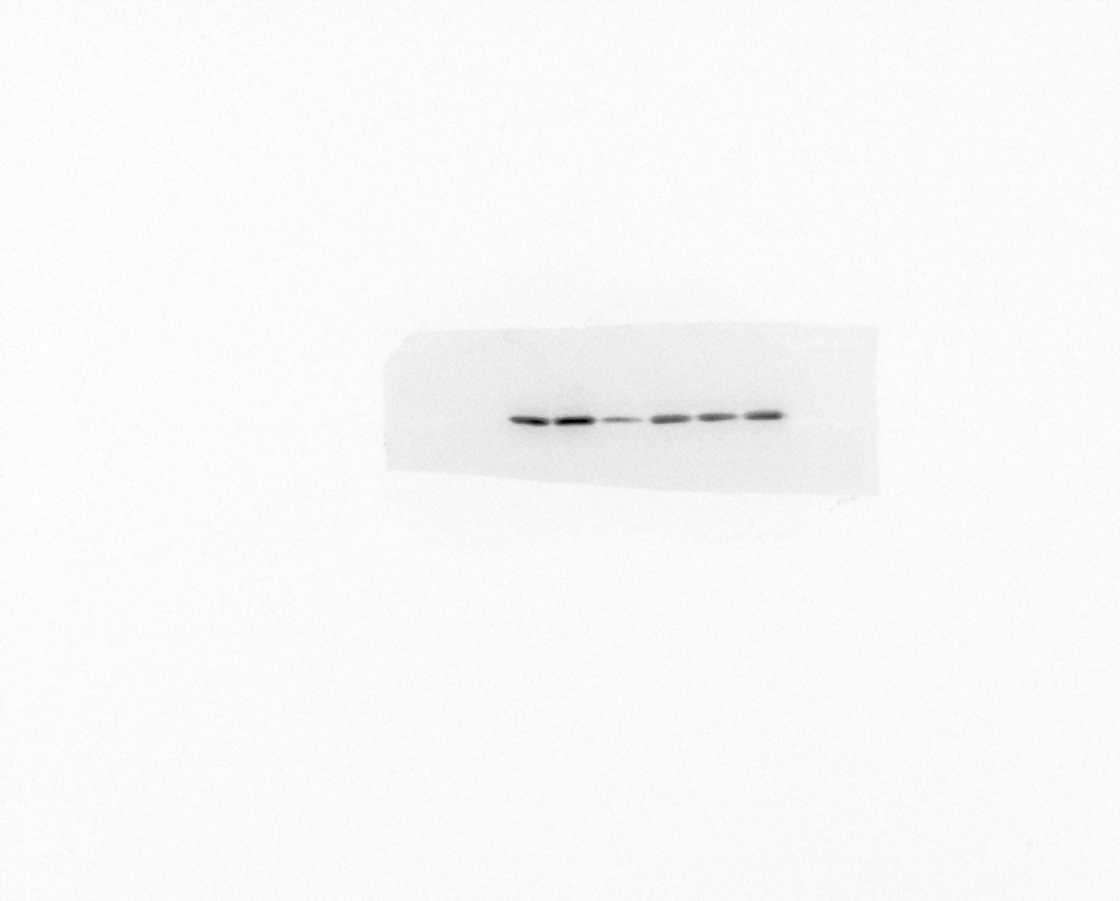

Supplement: Supplementary file 4 — Source Data [file 41467_2023_41520_MOESM4_ESM.zip › Source Data/Uncropped and Unprocessed Scans/Fig. 3e/IP HA; IB Ubc9.tif]

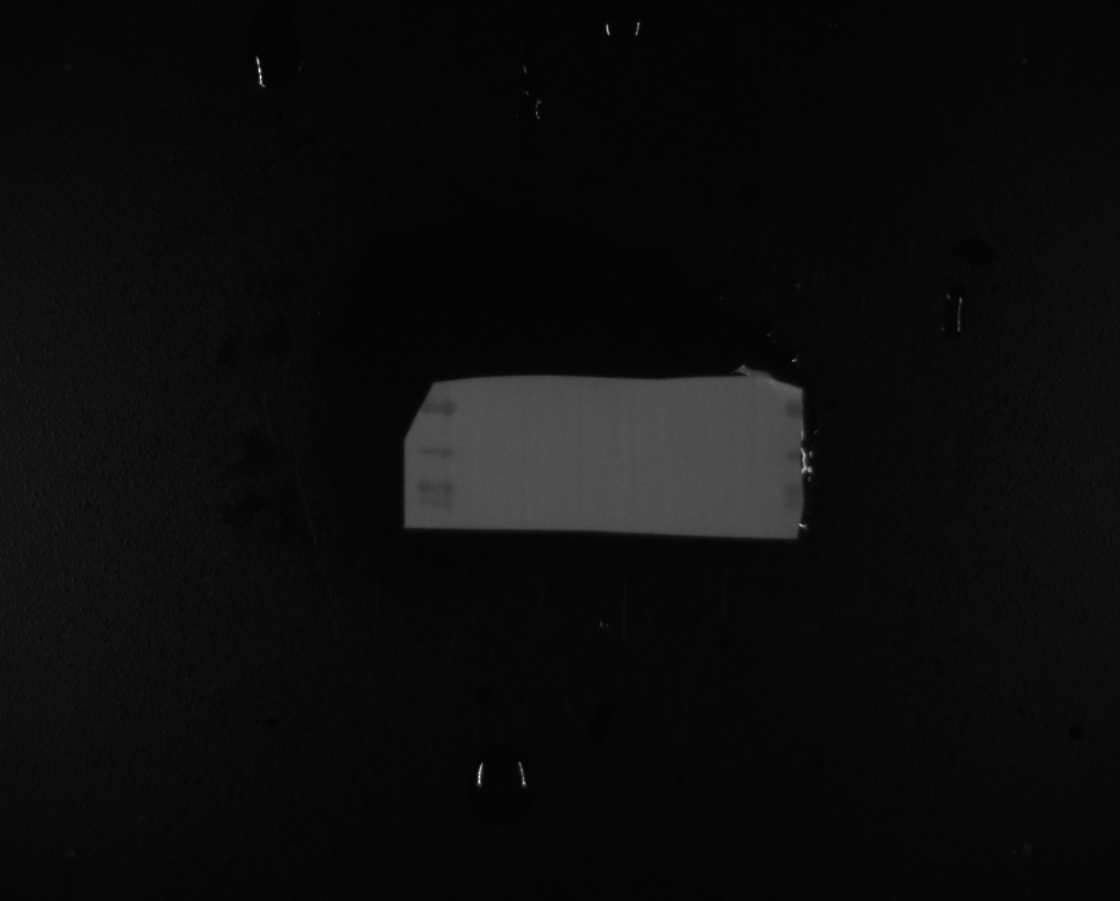

Supplement: Supplementary file 4 — Source Data [file 41467_2023_41520_MOESM4_ESM.zip › Source Data/Uncropped and Unprocessed Scans/Fig. 3f/IB GAPDH - Marker.tif]

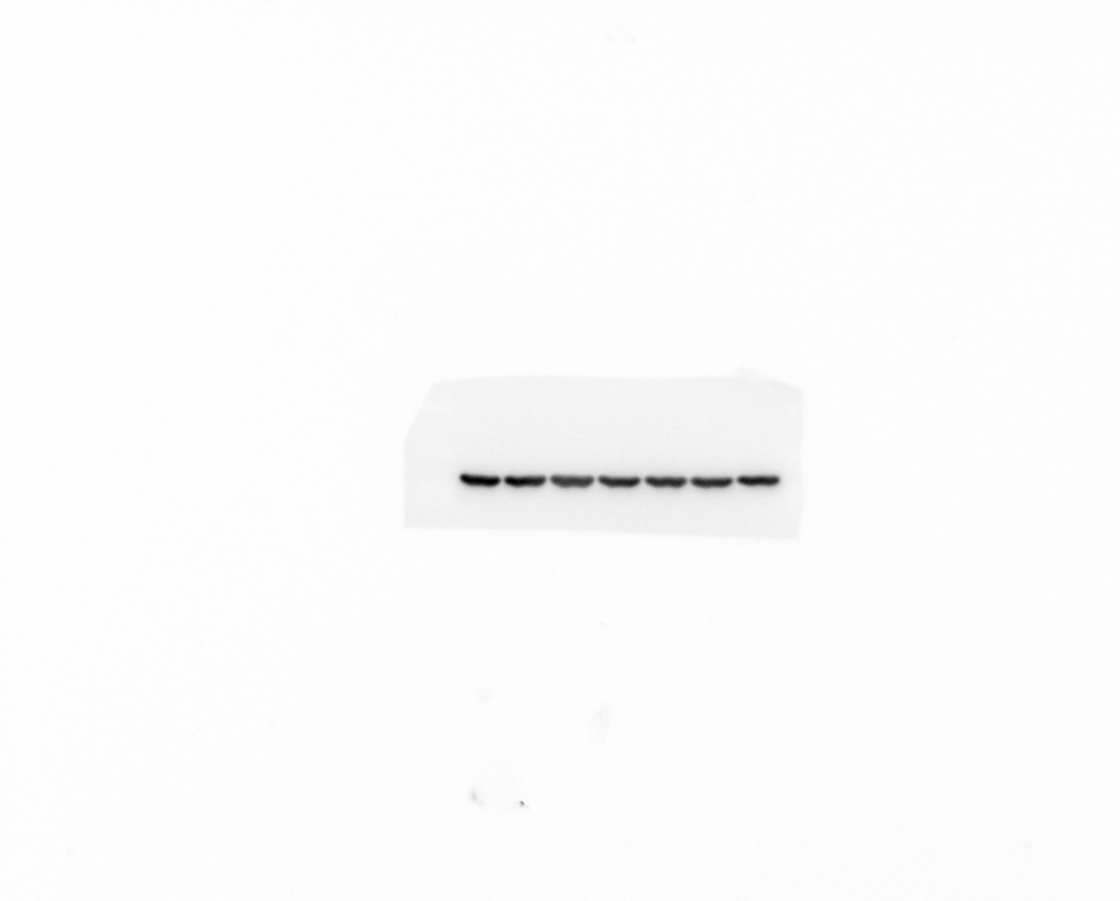

Supplement: Supplementary file 4 — Source Data [file 41467_2023_41520_MOESM4_ESM.zip › Source Data/Uncropped and Unprocessed Scans/Fig. 3f/IB GAPDH.tif]

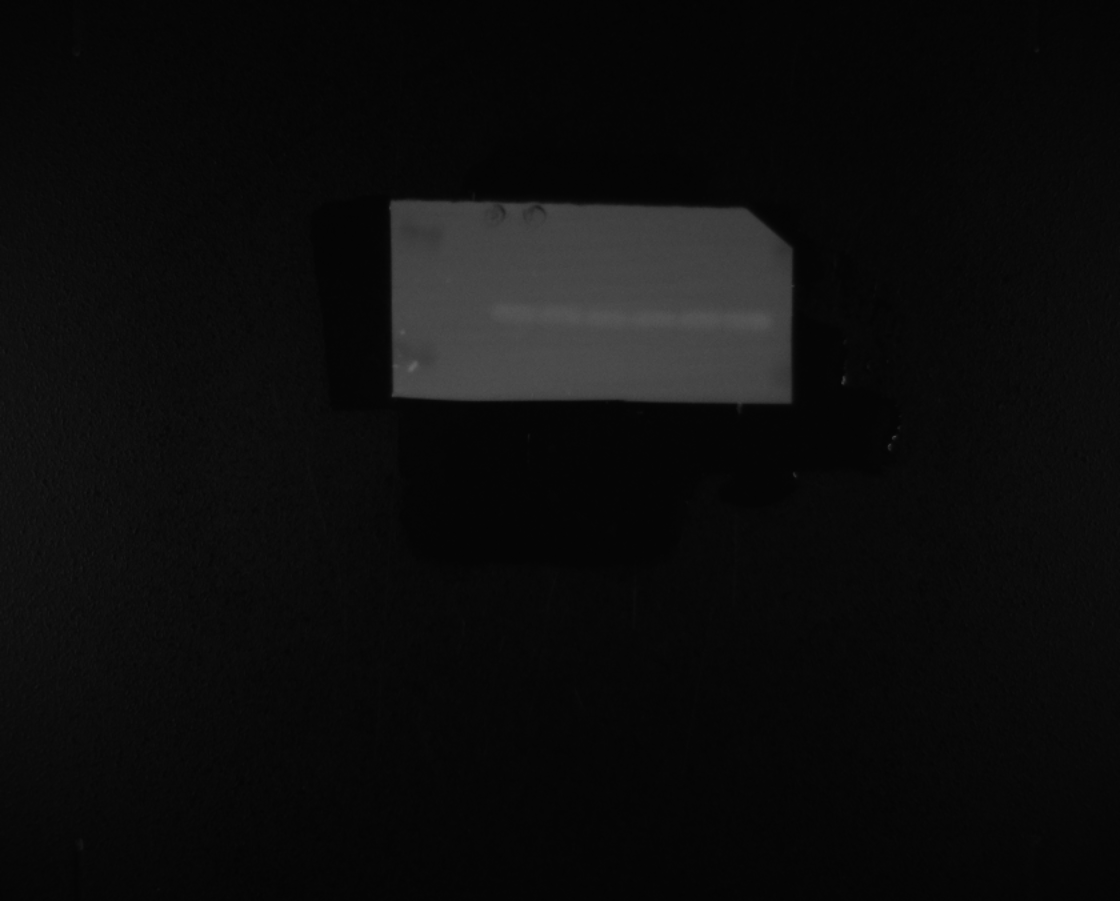

Supplement: Supplementary file 4 — Source Data [file 41467_2023_41520_MOESM4_ESM.zip › Source Data/Uncropped and Unprocessed Scans/Fig. 3f/IB HA - Marker.tif]

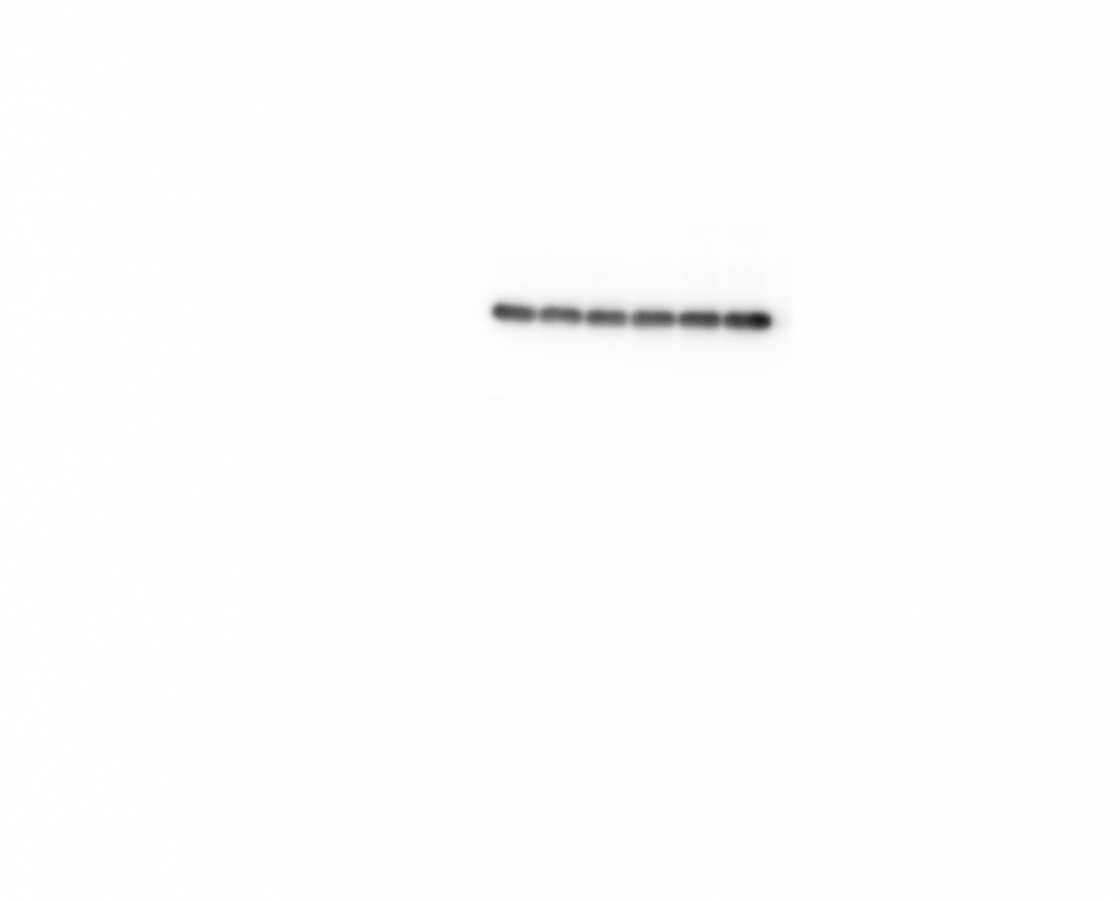

Supplement: Supplementary file 4 — Source Data [file 41467_2023_41520_MOESM4_ESM.zip › Source Data/Uncropped and Unprocessed Scans/Fig. 3f/IB HA.tif]

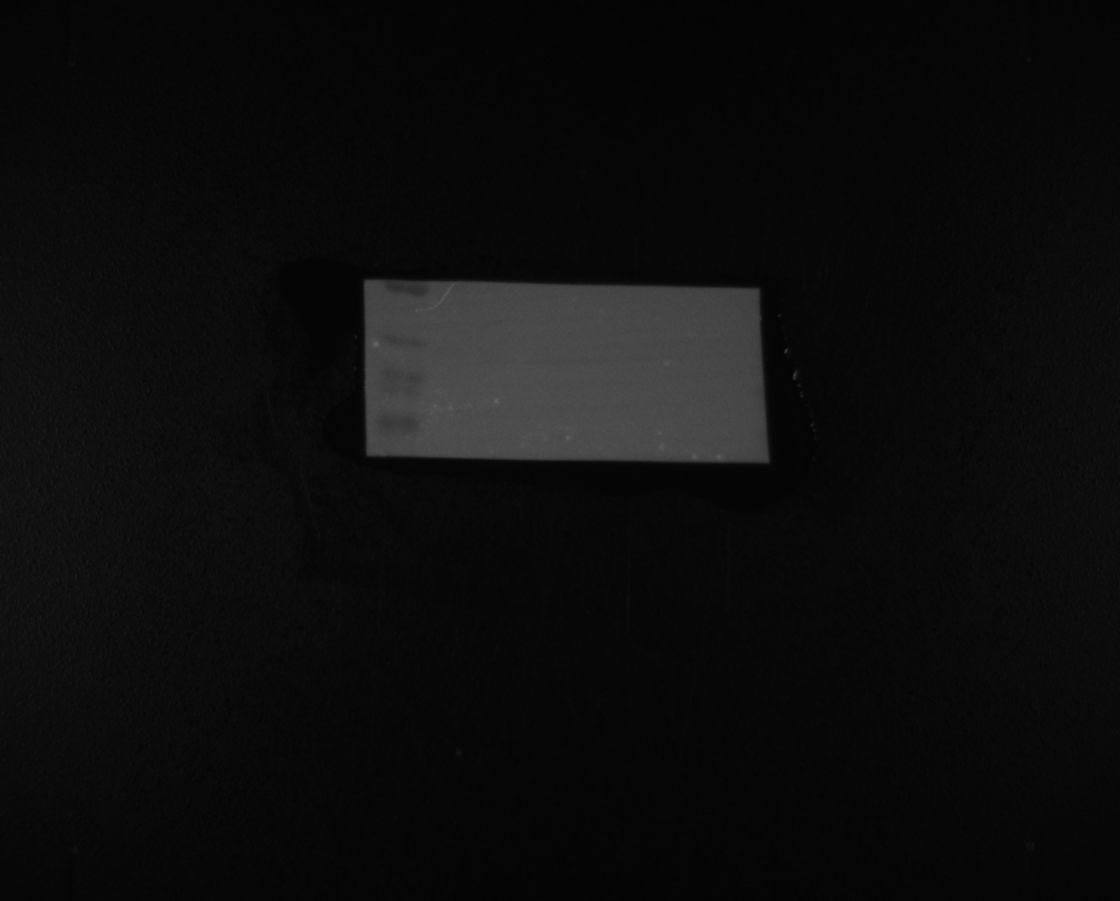

Supplement: Supplementary file 4 — Source Data [file 41467_2023_41520_MOESM4_ESM.zip › Source Data/Uncropped and Unprocessed Scans/Fig. 3f/IB SAE1 - Marker.tif]

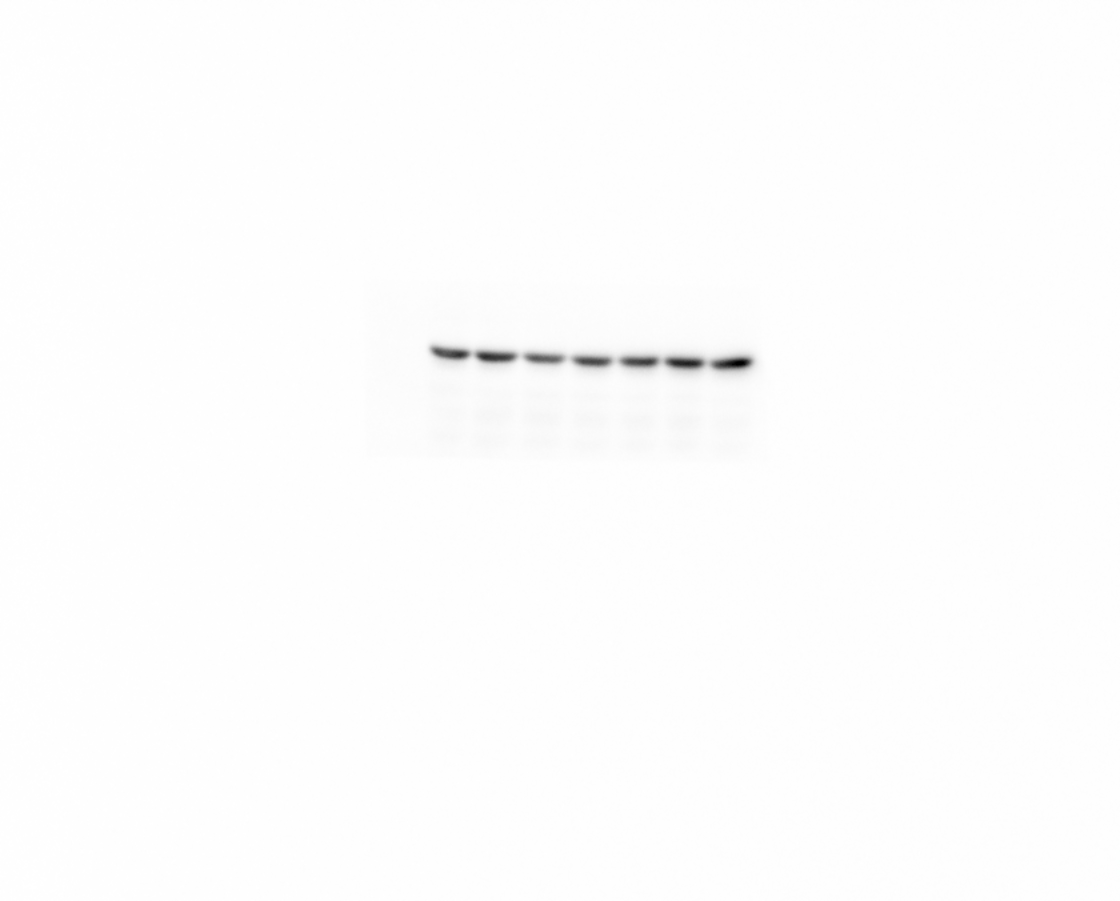

Supplement: Supplementary file 4 — Source Data [file 41467_2023_41520_MOESM4_ESM.zip › Source Data/Uncropped and Unprocessed Scans/Fig. 3f/IB SAE1.tif]

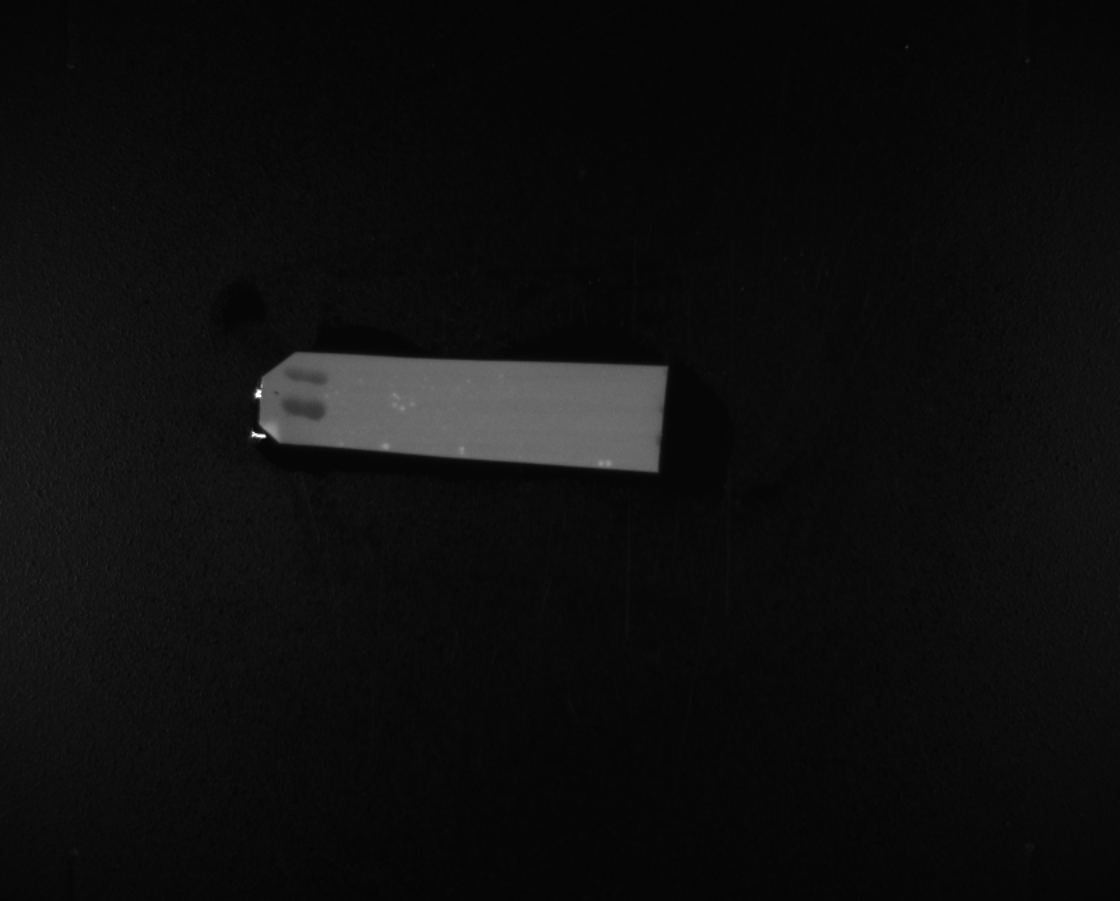

Supplement: Supplementary file 4 — Source Data [file 41467_2023_41520_MOESM4_ESM.zip › Source Data/Uncropped and Unprocessed Scans/Fig. 3f/IB SAE2 - Marker.tif]

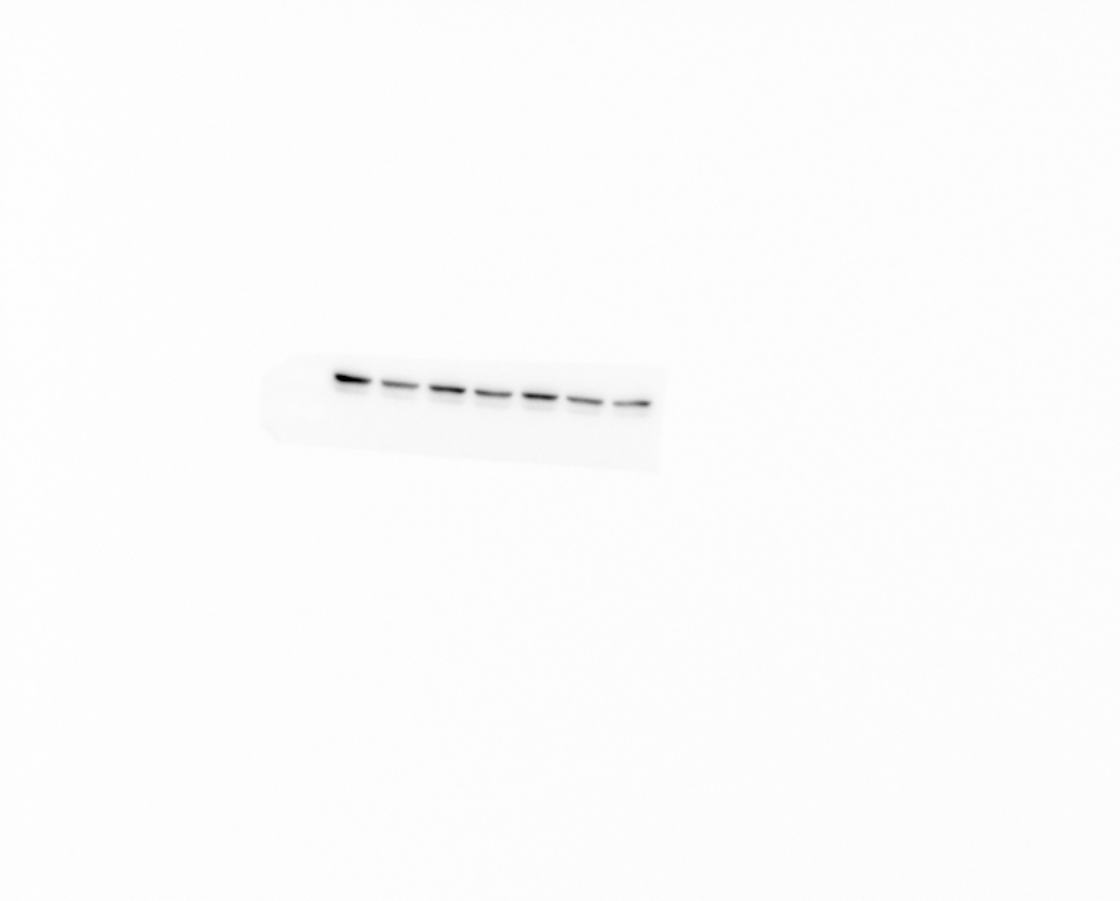

Supplement: Supplementary file 4 — Source Data [file 41467_2023_41520_MOESM4_ESM.zip › Source Data/Uncropped and Unprocessed Scans/Fig. 3f/IB SAE2.tif]

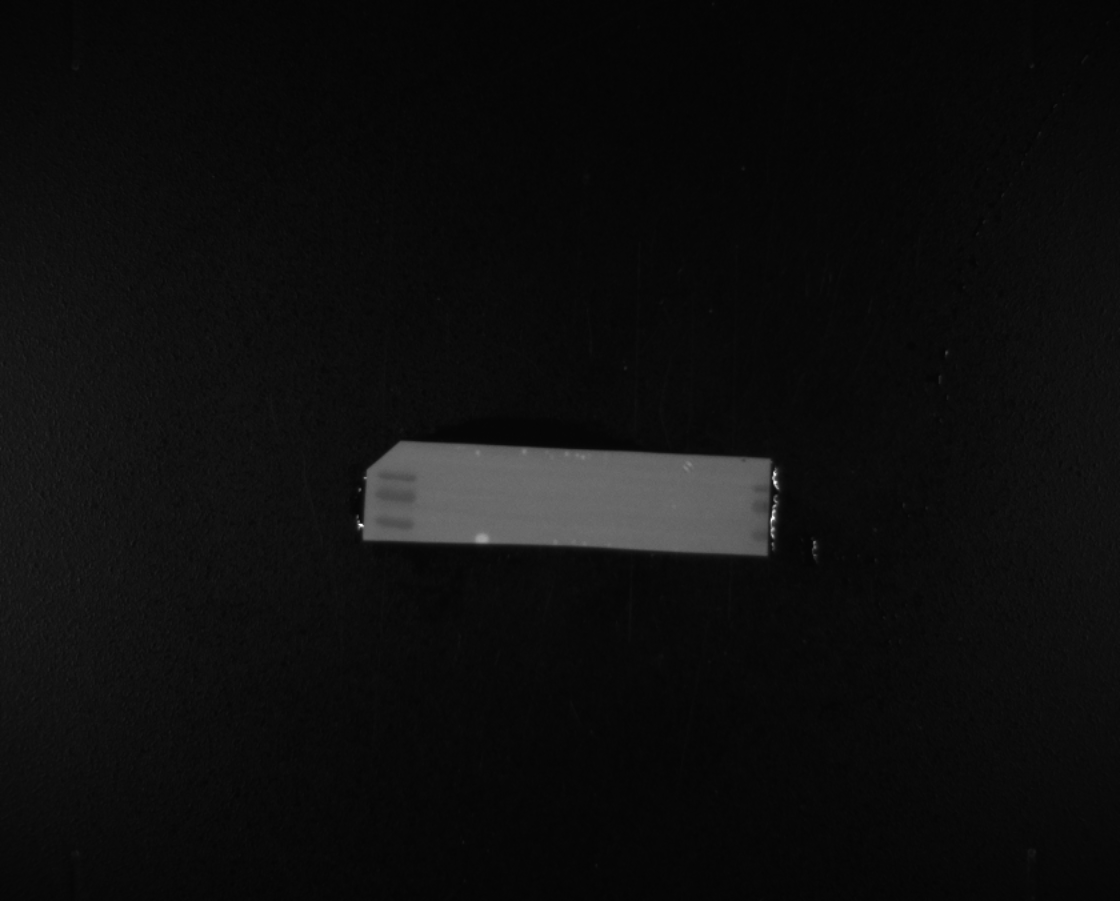

Supplement: Supplementary file 4 — Source Data [file 41467_2023_41520_MOESM4_ESM.zip › Source Data/Uncropped and Unprocessed Scans/Fig. 3f/IB SENP1 - Marker.tif]

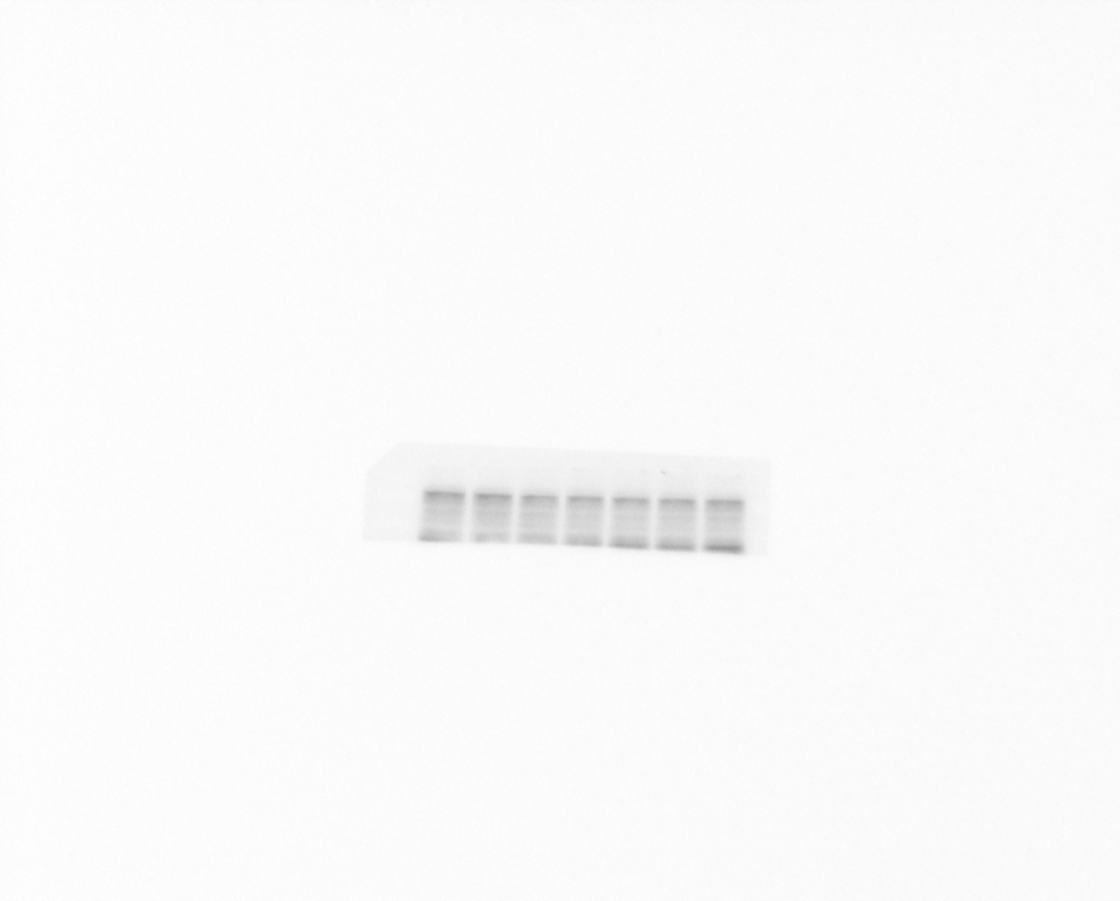

Supplement: Supplementary file 4 — Source Data [file 41467_2023_41520_MOESM4_ESM.zip › Source Data/Uncropped and Unprocessed Scans/Fig. 3f/IB SENP1.tif]

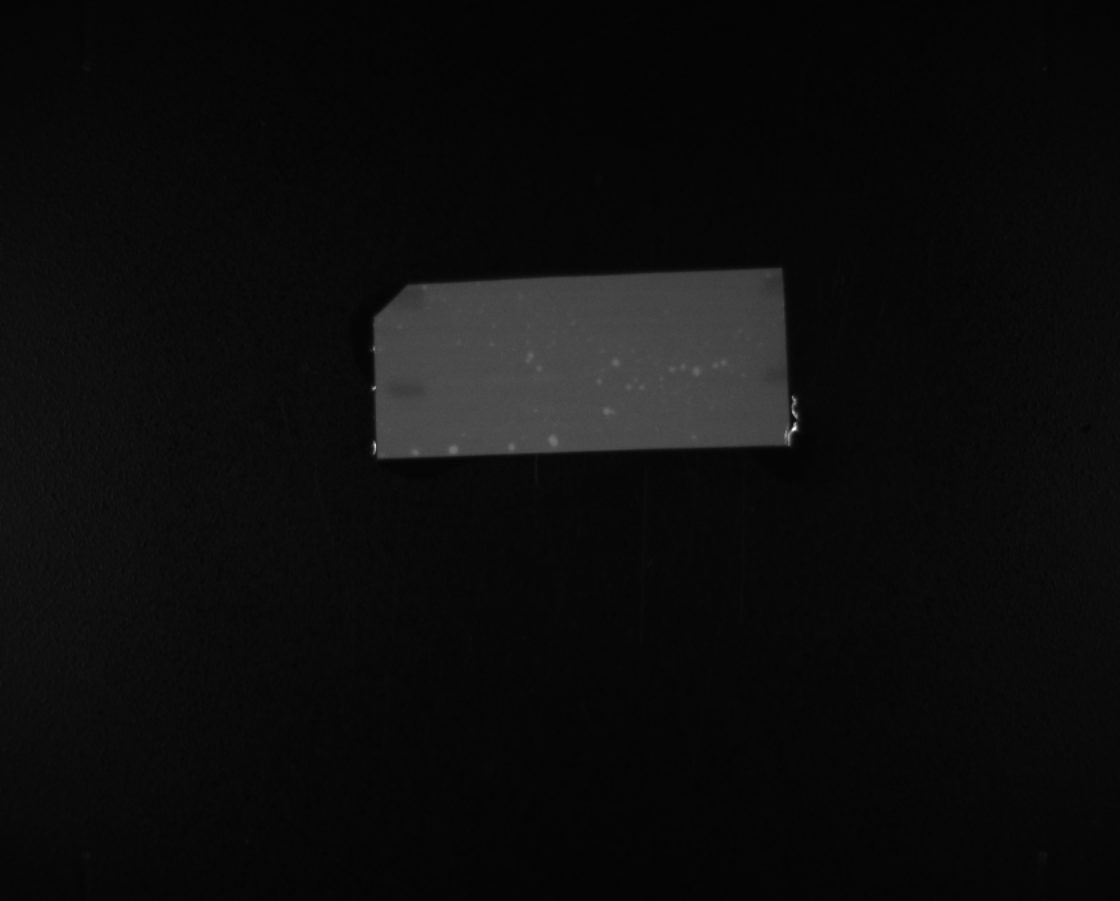

Supplement: Supplementary file 4 — Source Data [file 41467_2023_41520_MOESM4_ESM.zip › Source Data/Uncropped and Unprocessed Scans/Fig. 3f/IB Ubc9 - Marker.tif]

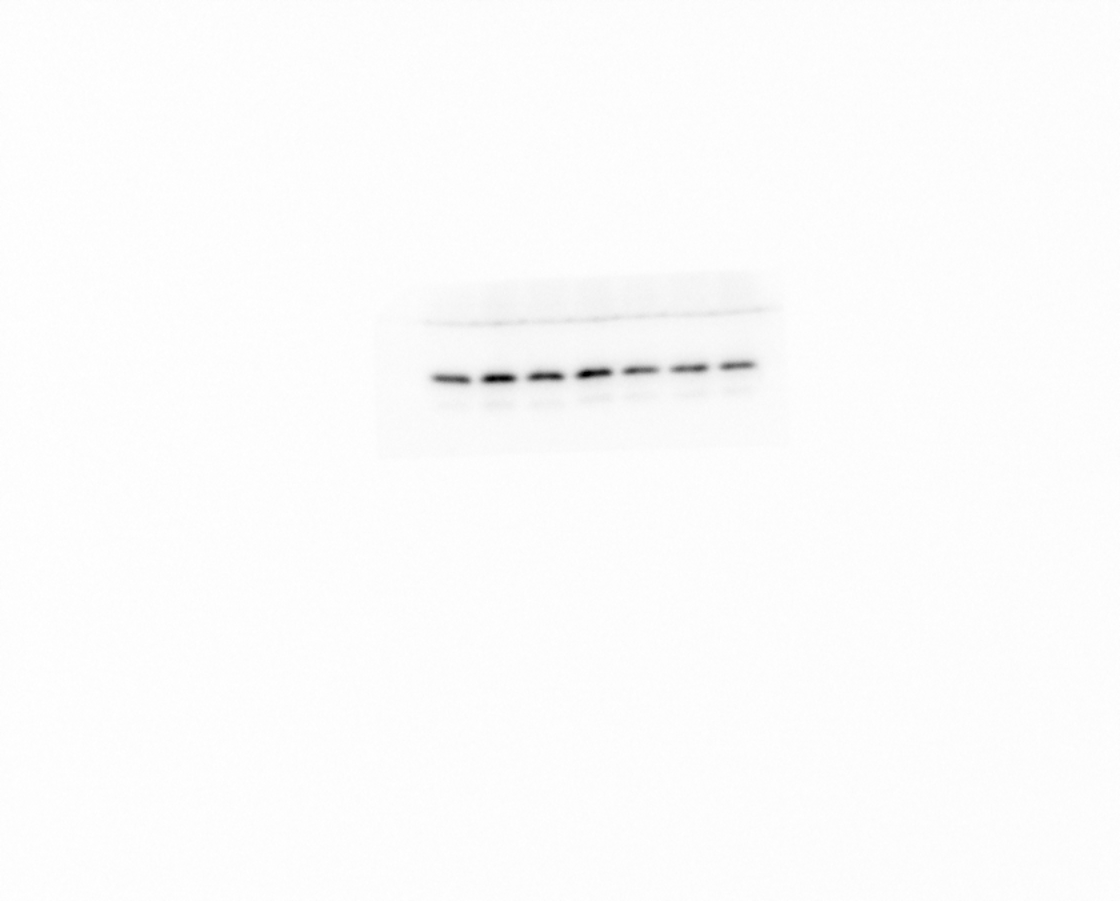

Supplement: Supplementary file 4 — Source Data [file 41467_2023_41520_MOESM4_ESM.zip › Source Data/Uncropped and Unprocessed Scans/Fig. 3f/IB Ubc9.tif]

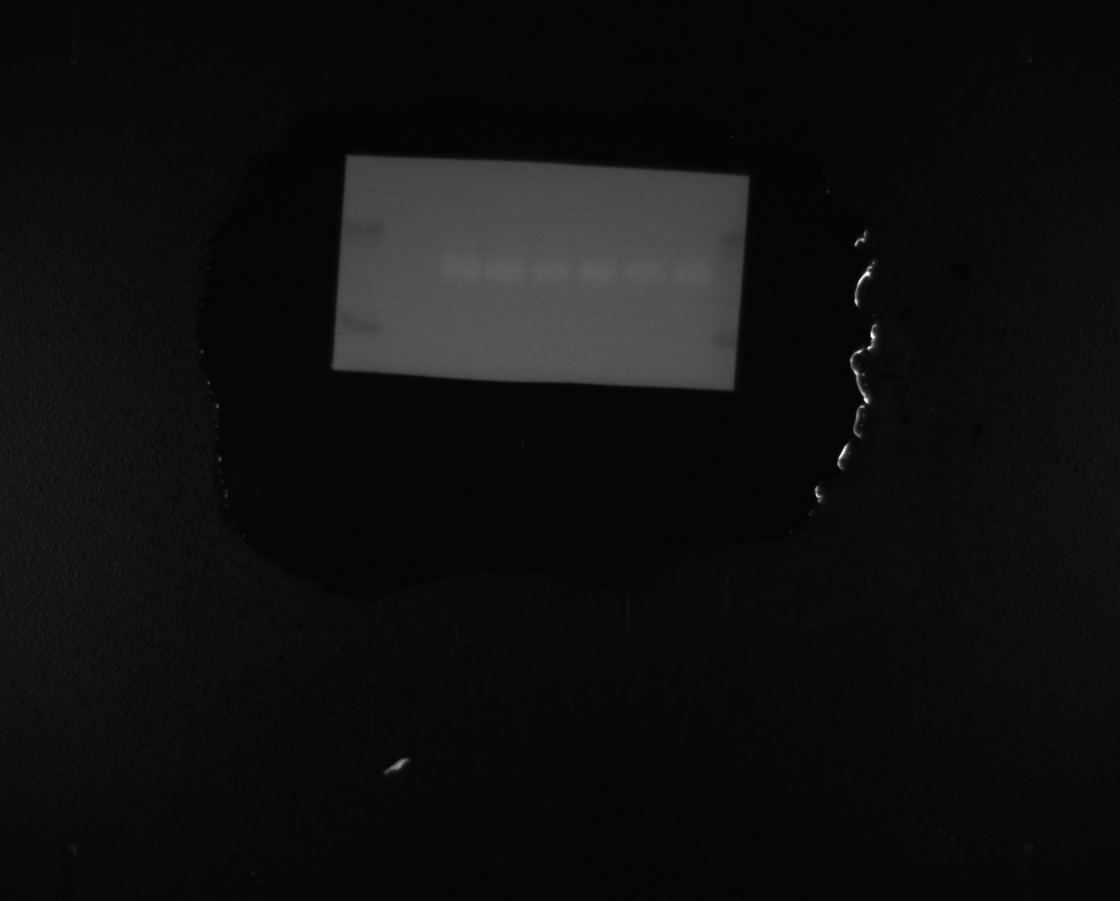

Supplement: Supplementary file 4 — Source Data [file 41467_2023_41520_MOESM4_ESM.zip › Source Data/Uncropped and Unprocessed Scans/Fig. 3f/IP HA; IB HA - Marker.tif]

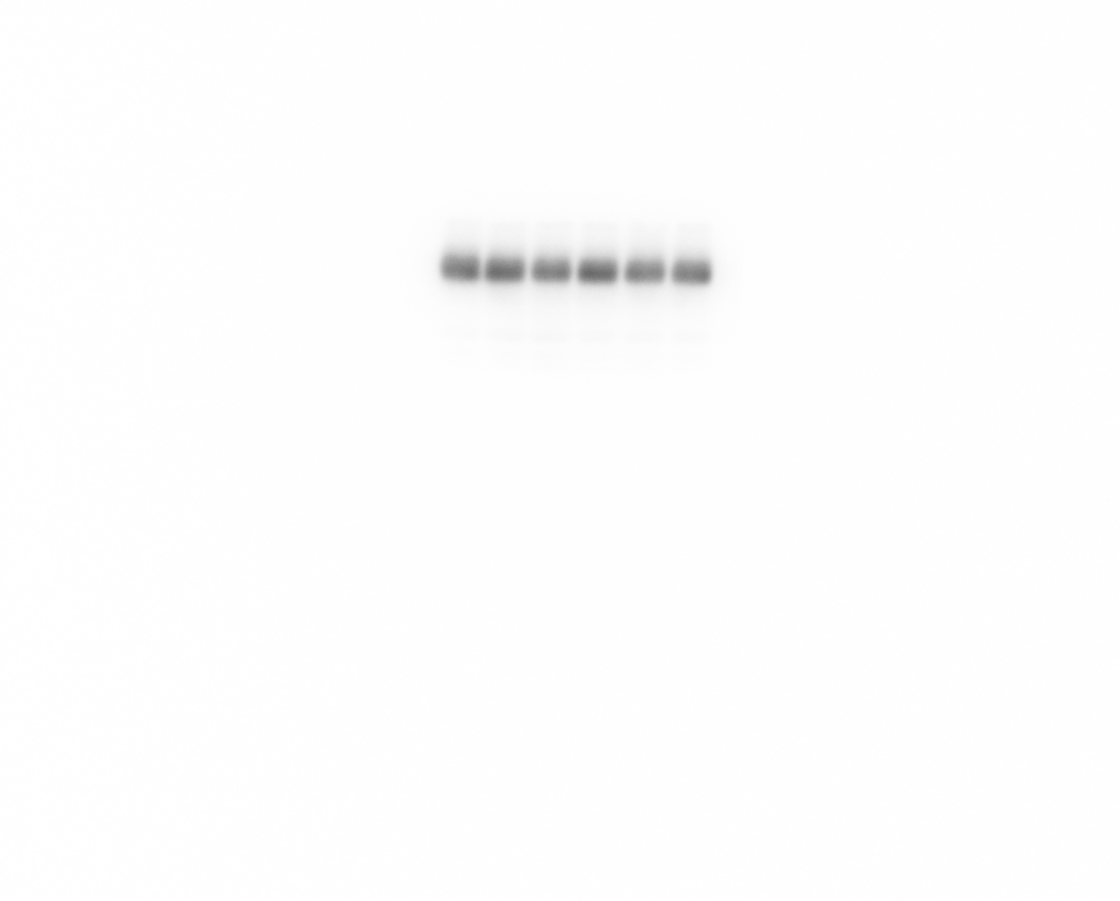

Supplement: Supplementary file 4 — Source Data [file 41467_2023_41520_MOESM4_ESM.zip › Source Data/Uncropped and Unprocessed Scans/Fig. 3f/IP HA; IB HA.tif]

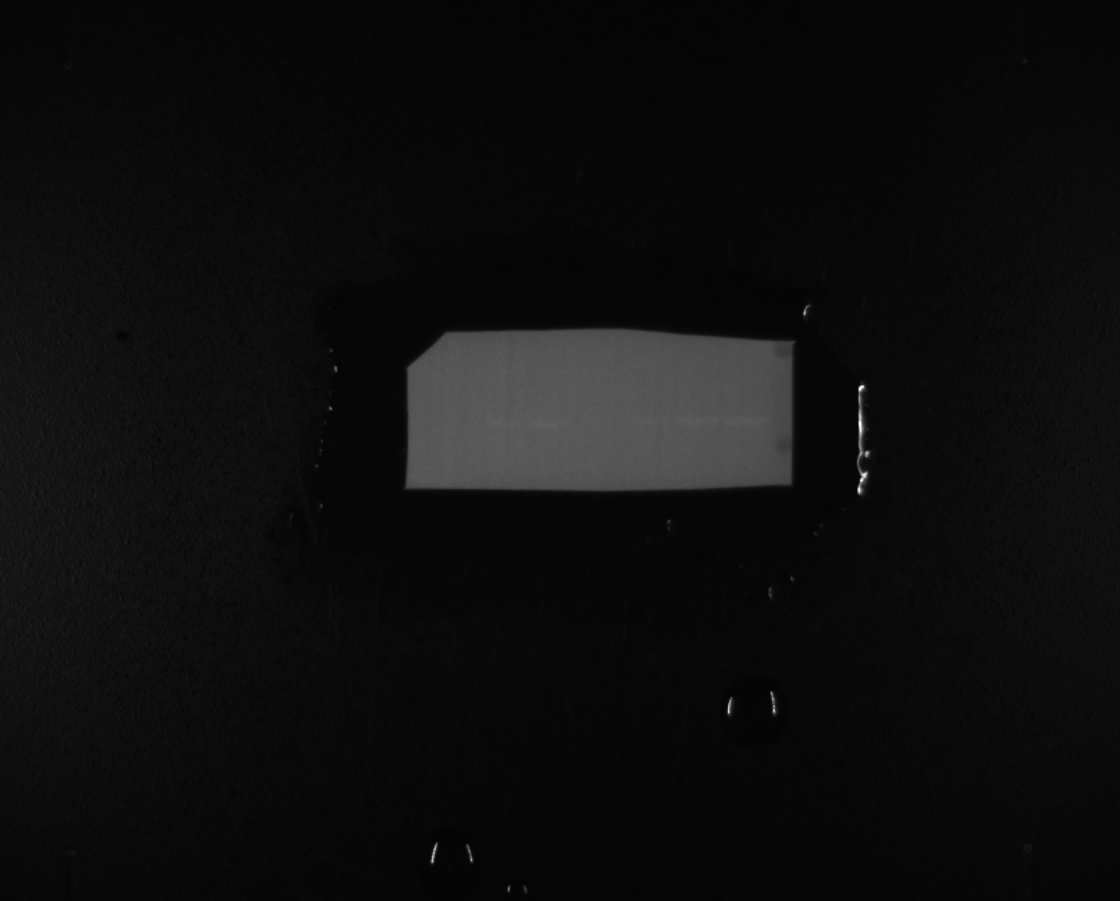

Supplement: Supplementary file 4 — Source Data [file 41467_2023_41520_MOESM4_ESM.zip › Source Data/Uncropped and Unprocessed Scans/Fig. 3f/IP HA; IB SAE1 - Marker.tif]

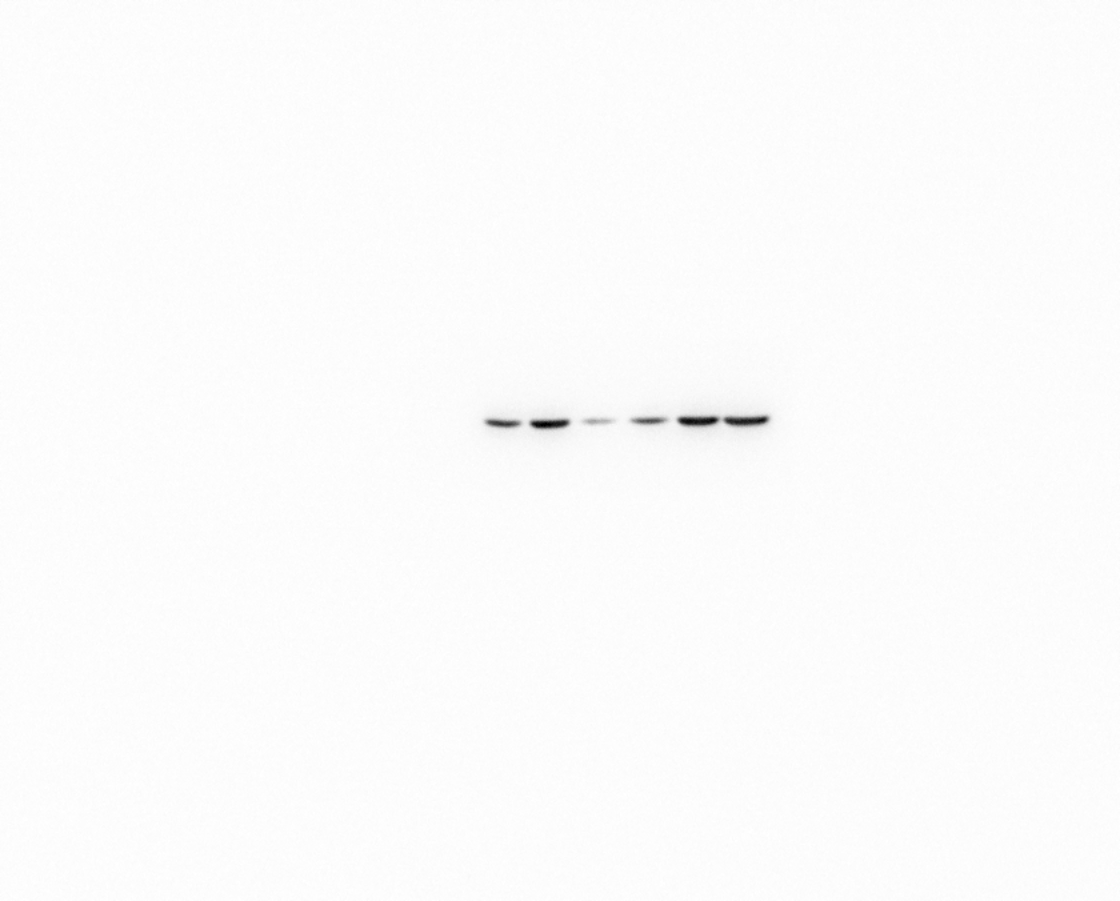

Supplement: Supplementary file 4 — Source Data [file 41467_2023_41520_MOESM4_ESM.zip › Source Data/Uncropped and Unprocessed Scans/Fig. 3f/IP HA; IB SAE1.tif]

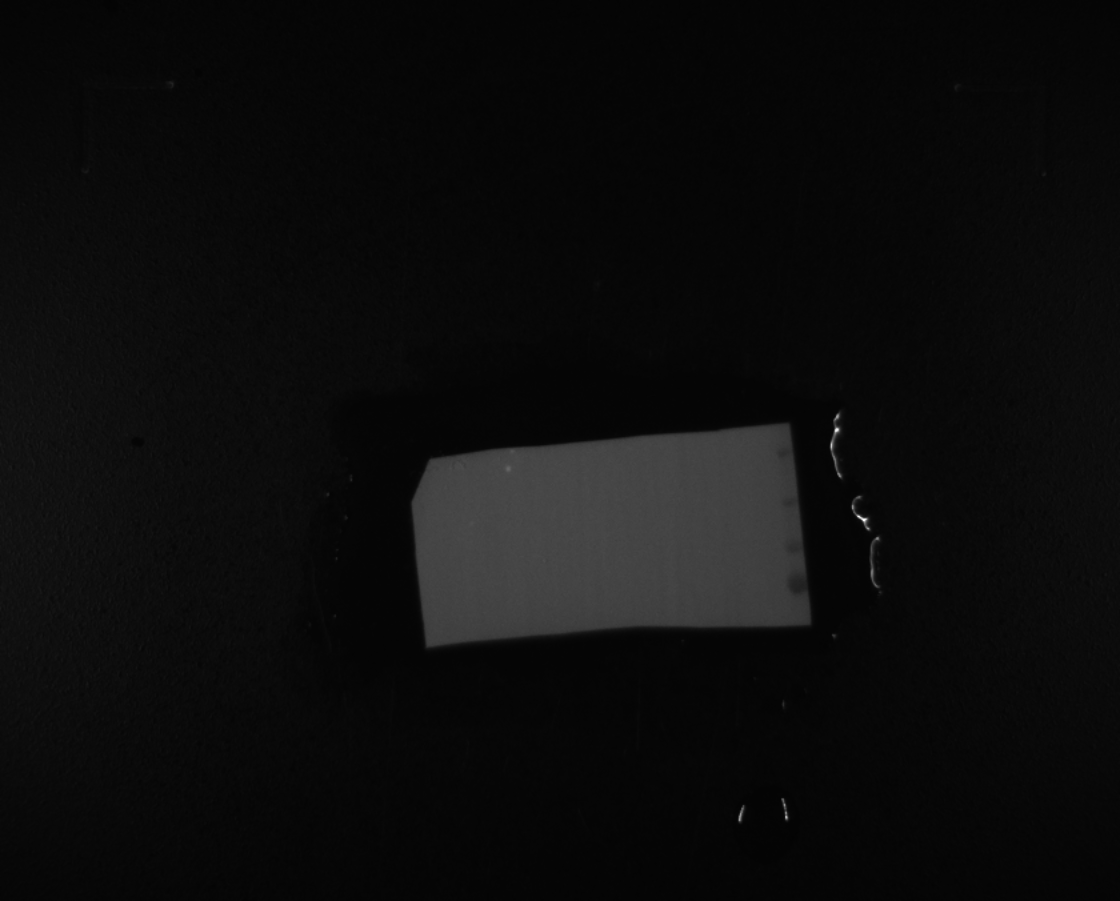

Supplement: Supplementary file 4 — Source Data [file 41467_2023_41520_MOESM4_ESM.zip › Source Data/Uncropped and Unprocessed Scans/Fig. 3f/IP HA; IB SAE2 - Marker.tif]

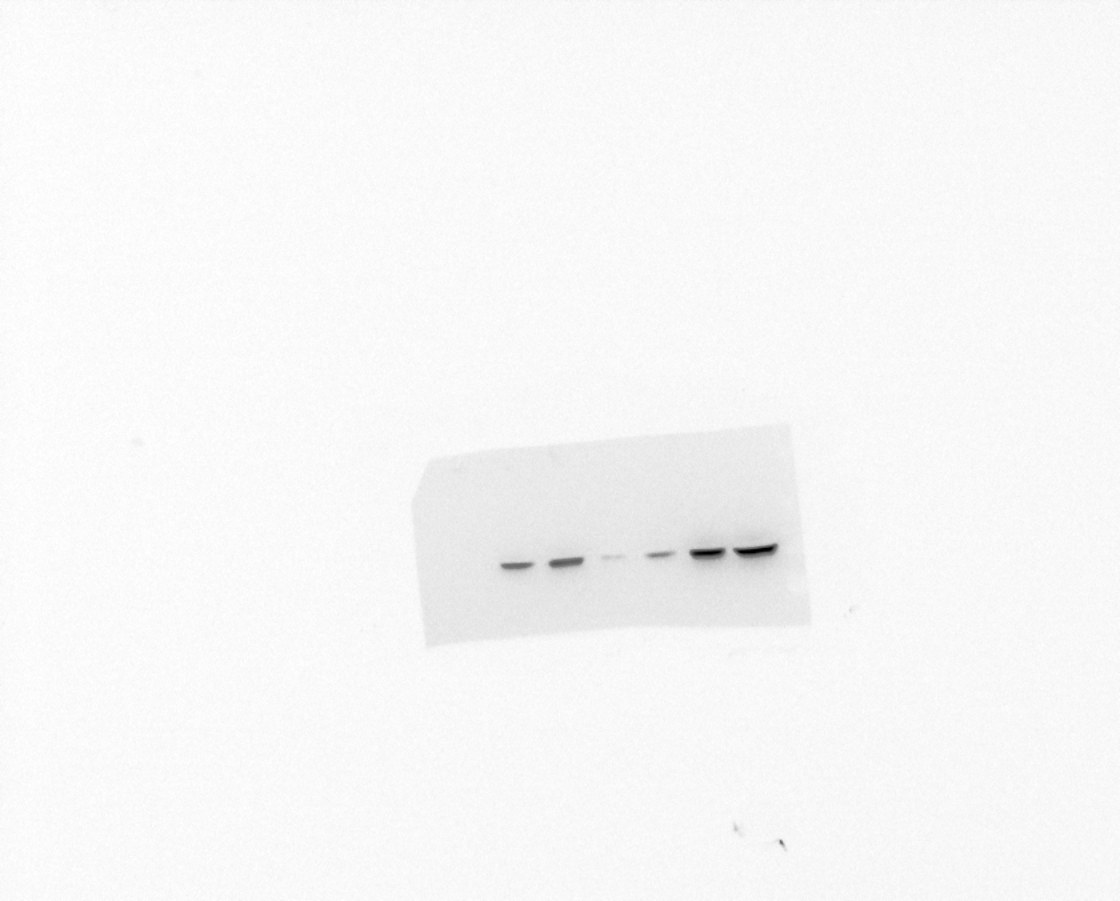

Supplement: Supplementary file 4 — Source Data [file 41467_2023_41520_MOESM4_ESM.zip › Source Data/Uncropped and Unprocessed Scans/Fig. 3f/IP HA; IB SAE2.tif]

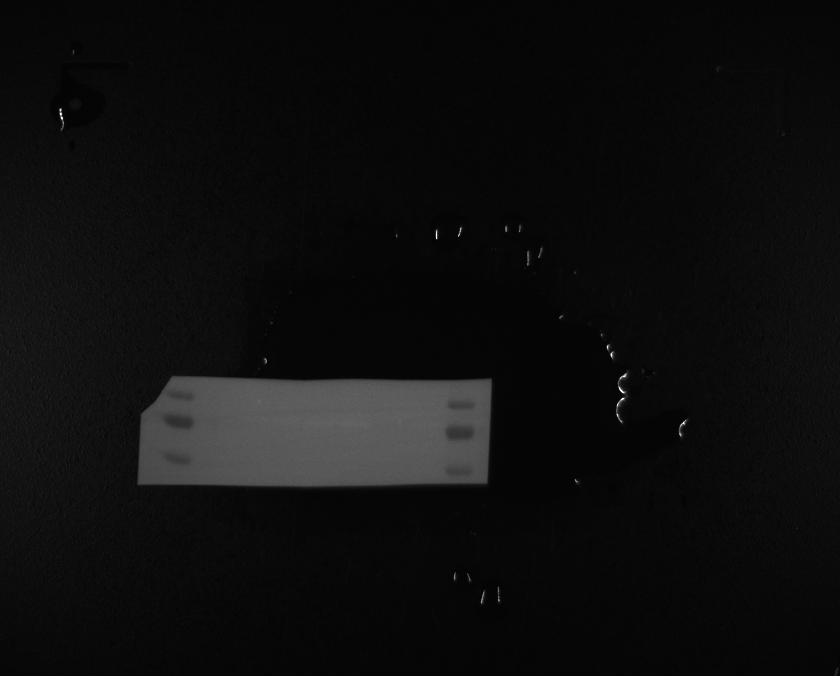

Supplement: Supplementary file 4 — Source Data [file 41467_2023_41520_MOESM4_ESM.zip › Source Data/Uncropped and Unprocessed Scans/Fig. 3f/IP HA; IB SENP1 - Marker.tif]

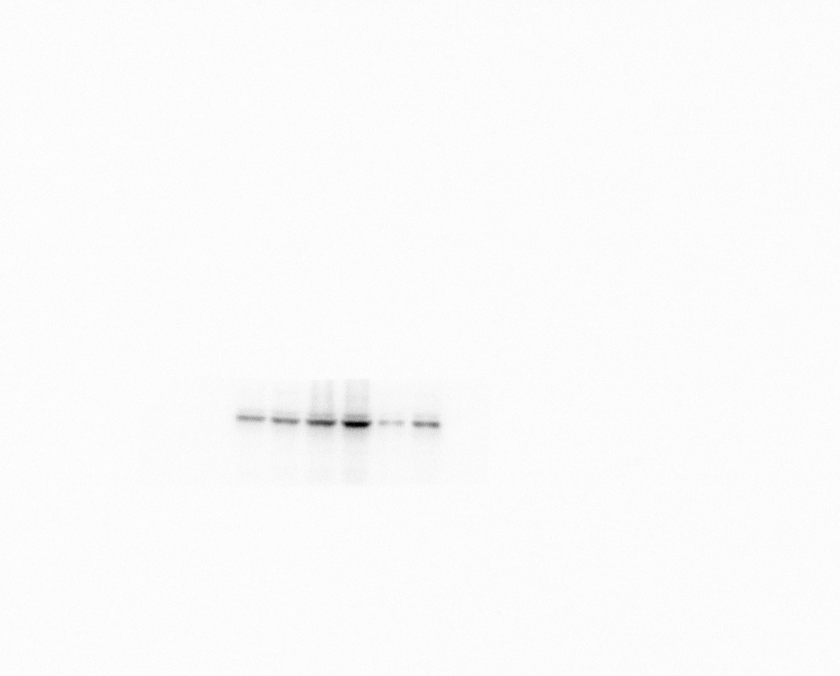

Supplement: Supplementary file 4 — Source Data [file 41467_2023_41520_MOESM4_ESM.zip › Source Data/Uncropped and Unprocessed Scans/Fig. 3f/IP HA; IB SENP1.tif]

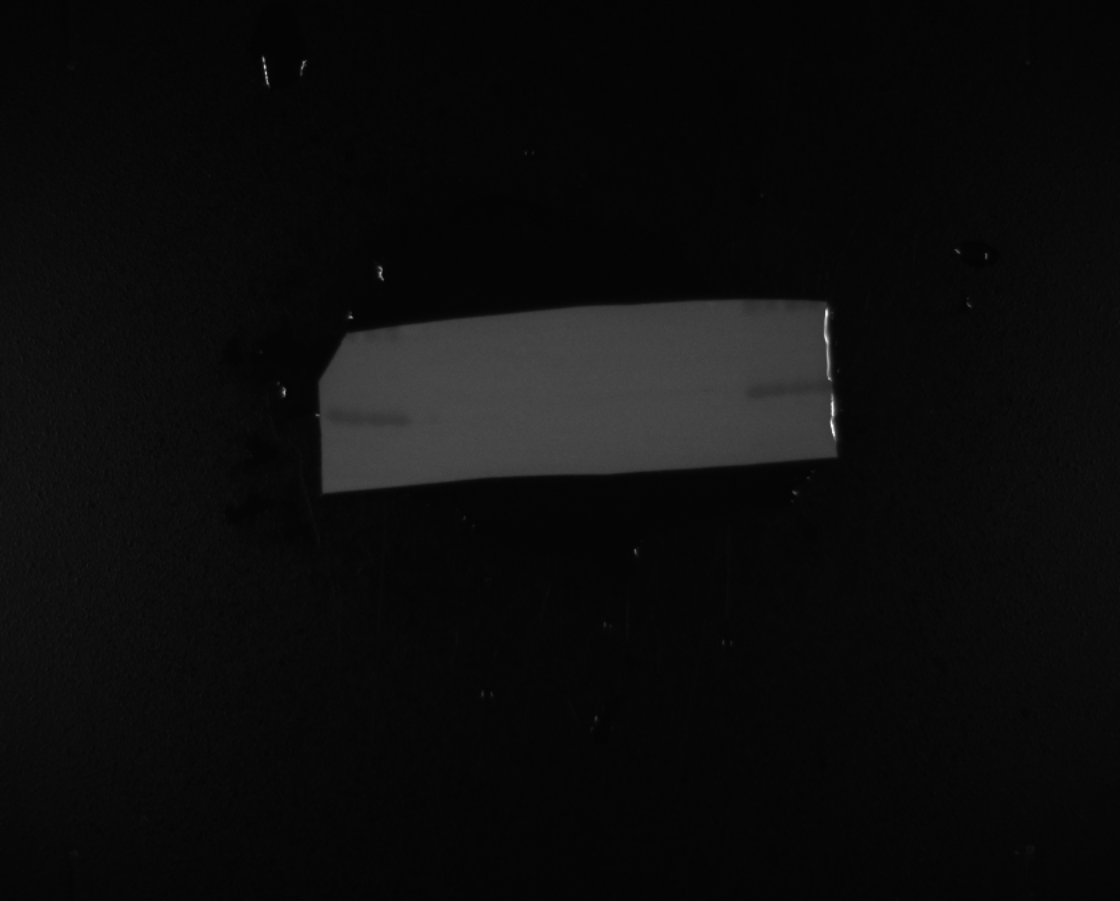

Supplement: Supplementary file 4 — Source Data [file 41467_2023_41520_MOESM4_ESM.zip › Source Data/Uncropped and Unprocessed Scans/Fig. 3f/IP HA; IB Ubc9 - Marker.tif]

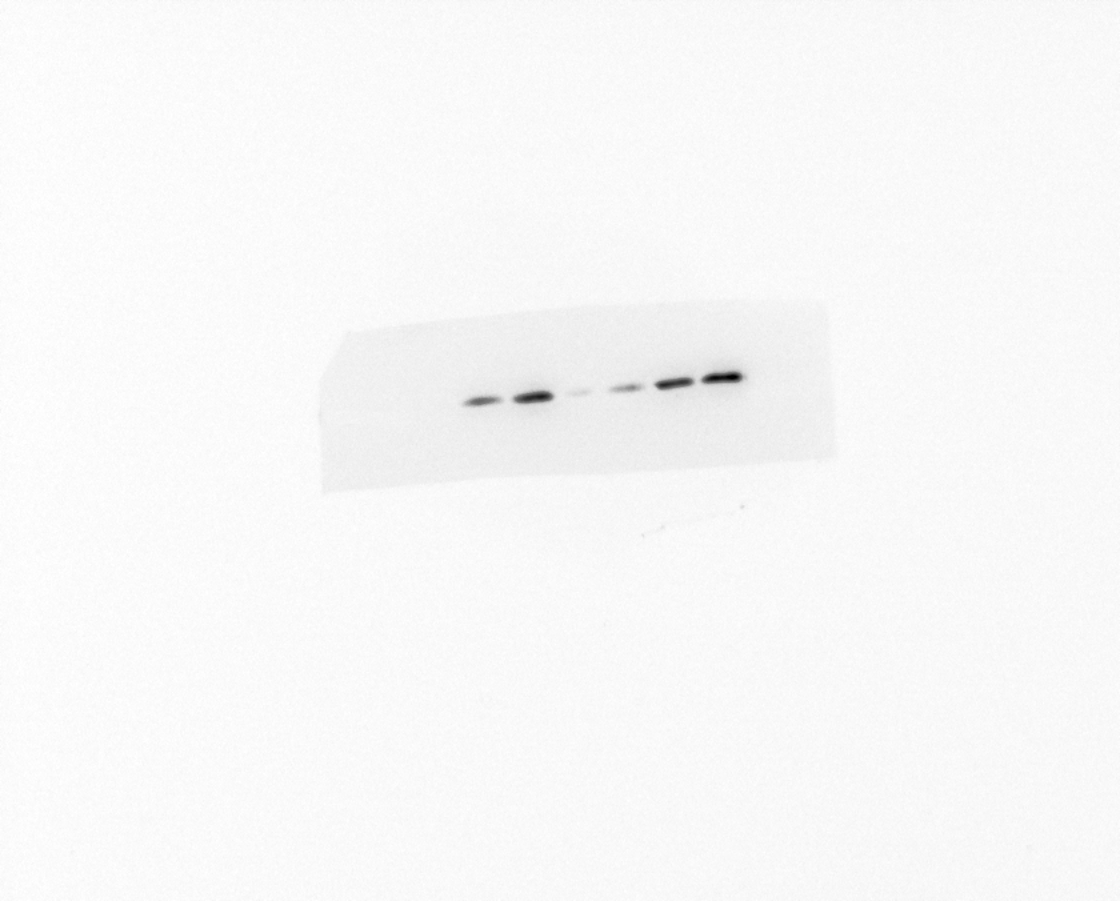

Supplement: Supplementary file 4 — Source Data [file 41467_2023_41520_MOESM4_ESM.zip › Source Data/Uncropped and Unprocessed Scans/Fig. 3f/IP HA; IB Ubc9.tif]

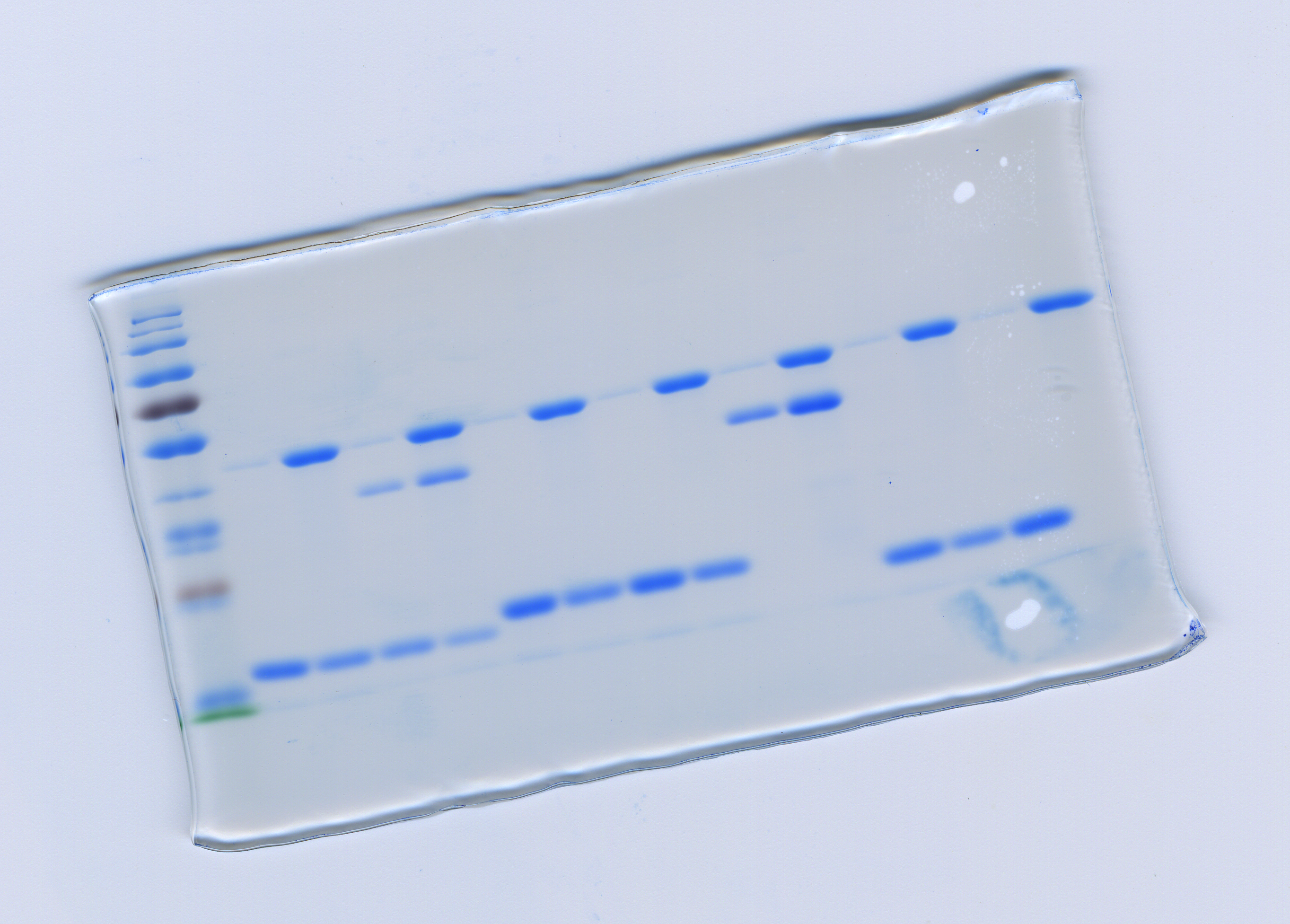

Supplement: Supplementary file 4 — Source Data [file 41467_2023_41520_MOESM4_ESM.zip › Source Data/Uncropped and Unprocessed Scans/Fig. 4b/CBB.tif]

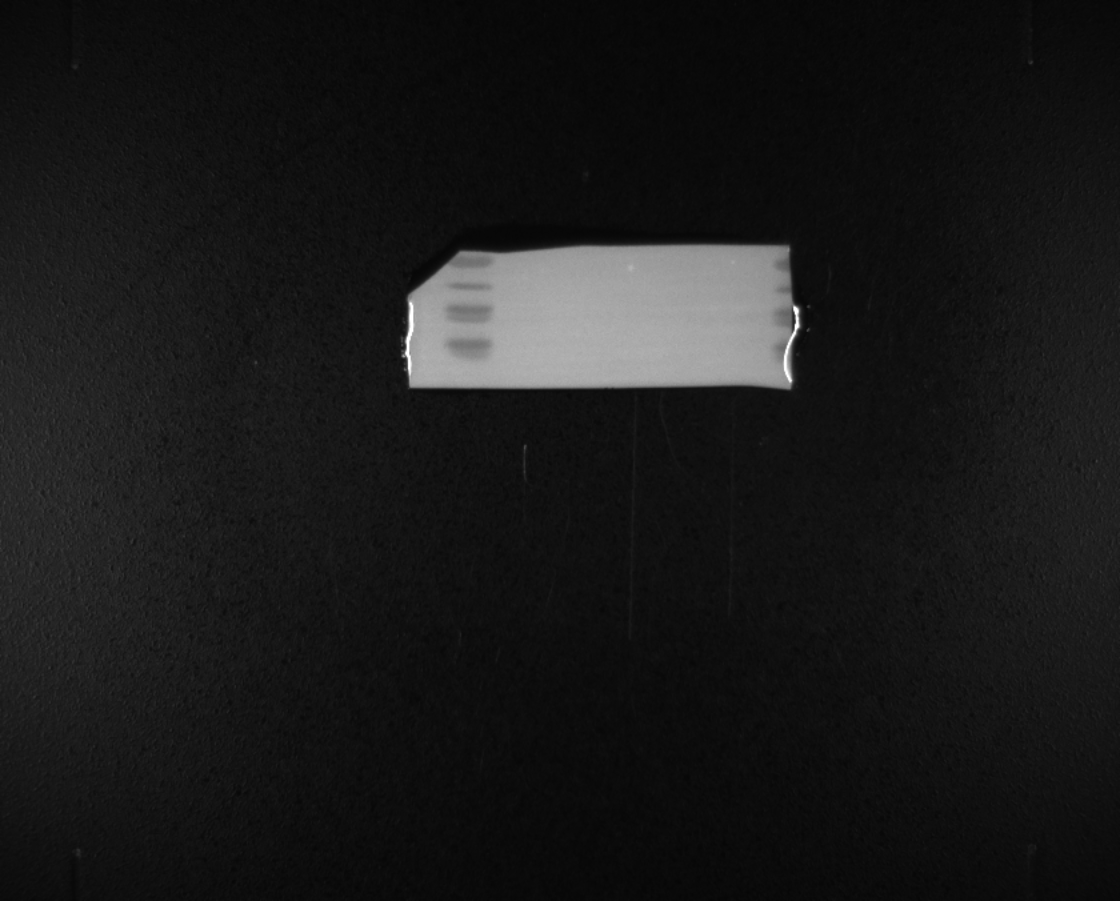

Supplement: Supplementary file 4 — Source Data [file 41467_2023_41520_MOESM4_ESM.zip › Source Data/Uncropped and Unprocessed Scans/Fig. S1c/IB GAPDH - Marker.tif]

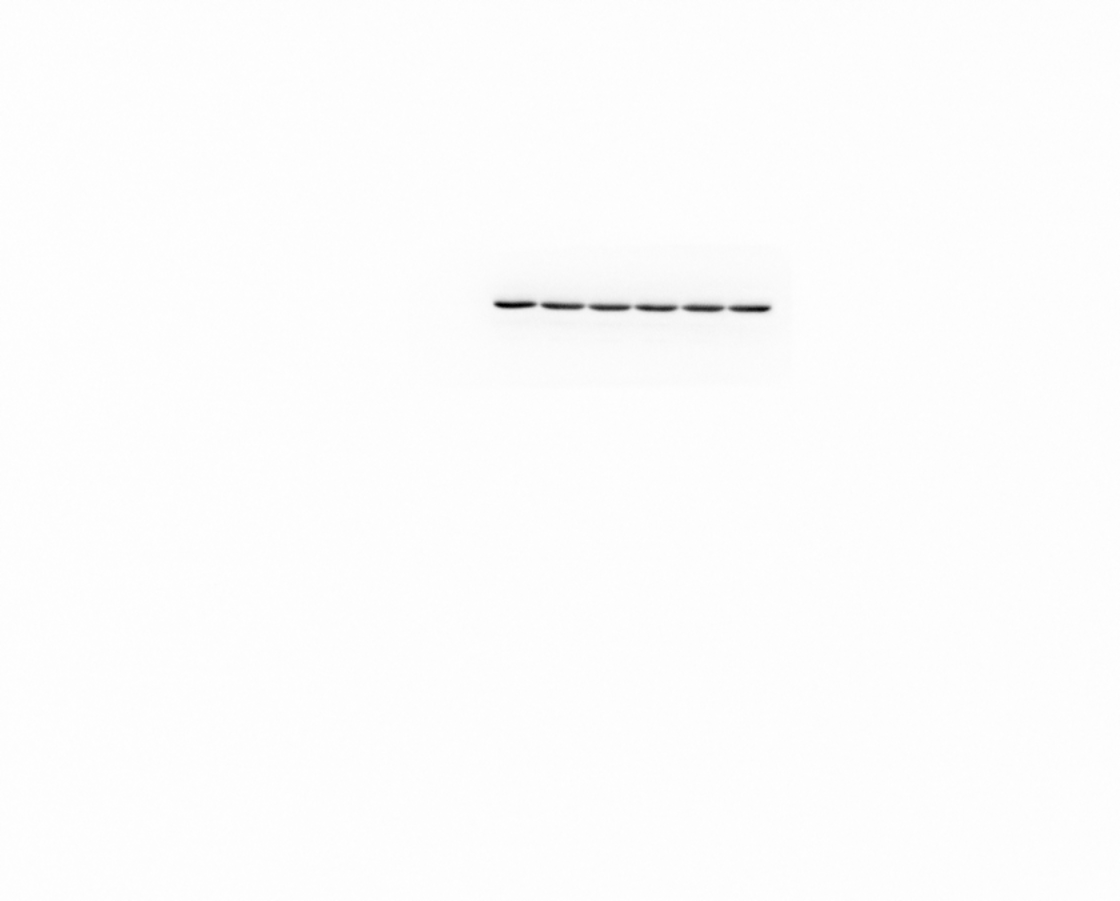

Supplement: Supplementary file 4 — Source Data [file 41467_2023_41520_MOESM4_ESM.zip › Source Data/Uncropped and Unprocessed Scans/Fig. S1c/IB GAPDH.tif]

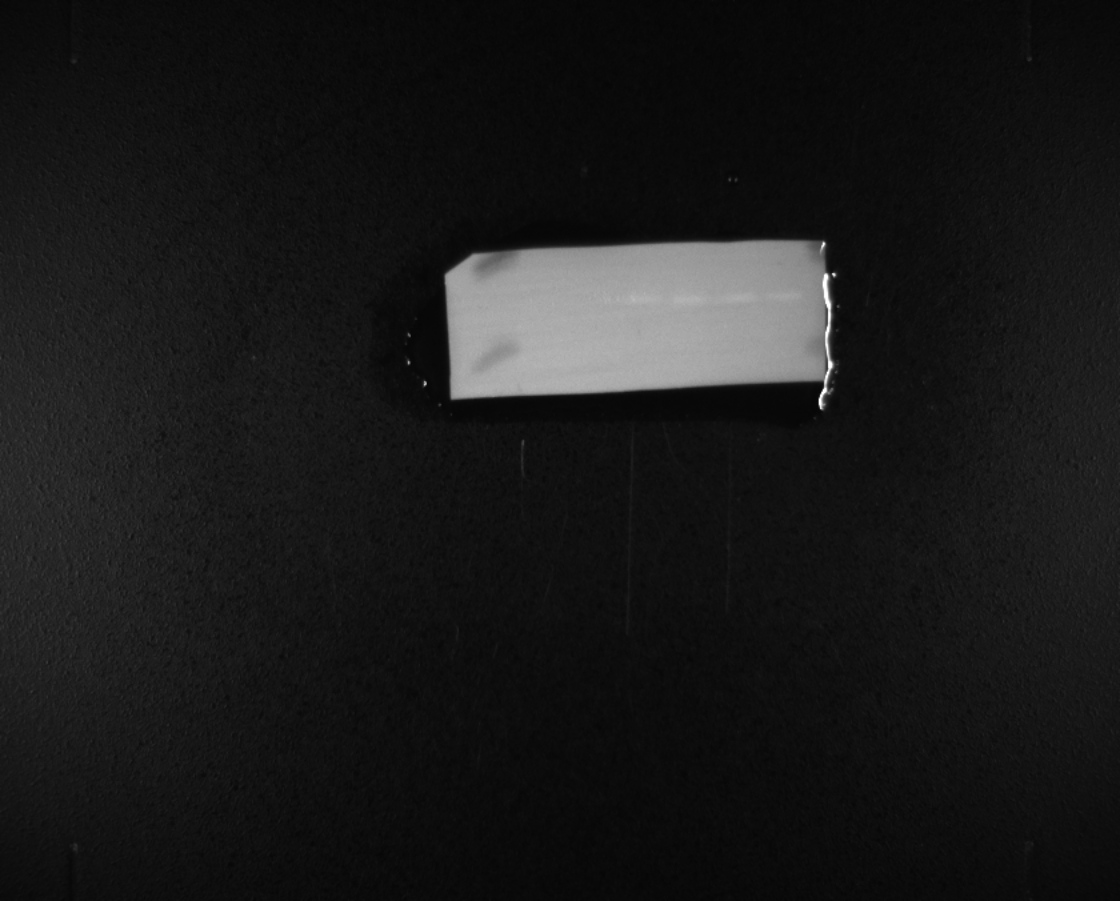

Supplement: Supplementary file 4 — Source Data [file 41467_2023_41520_MOESM4_ESM.zip › Source Data/Uncropped and Unprocessed Scans/Fig. S1c/IB HA; IB His - Marker.tif]

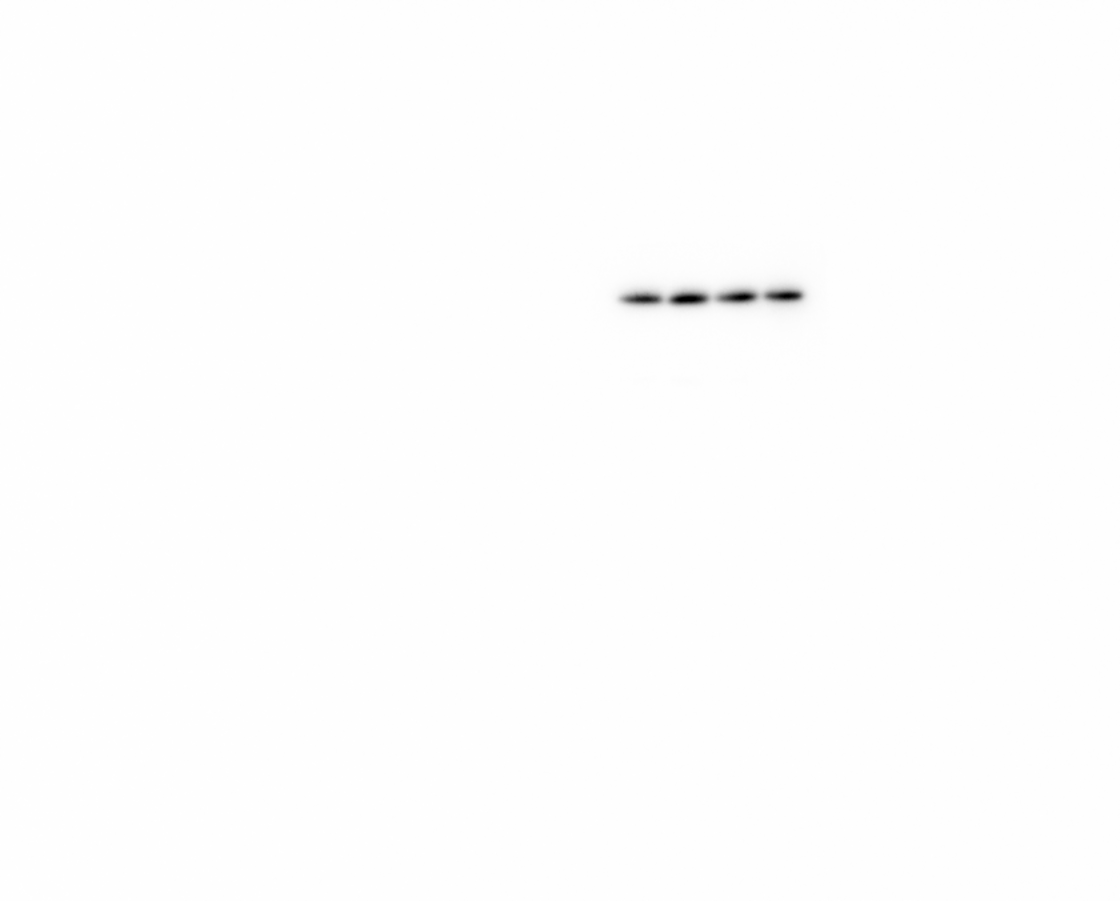

Supplement: Supplementary file 4 — Source Data [file 41467_2023_41520_MOESM4_ESM.zip › Source Data/Uncropped and Unprocessed Scans/Fig. S1c/IB HA; IB His.tif]

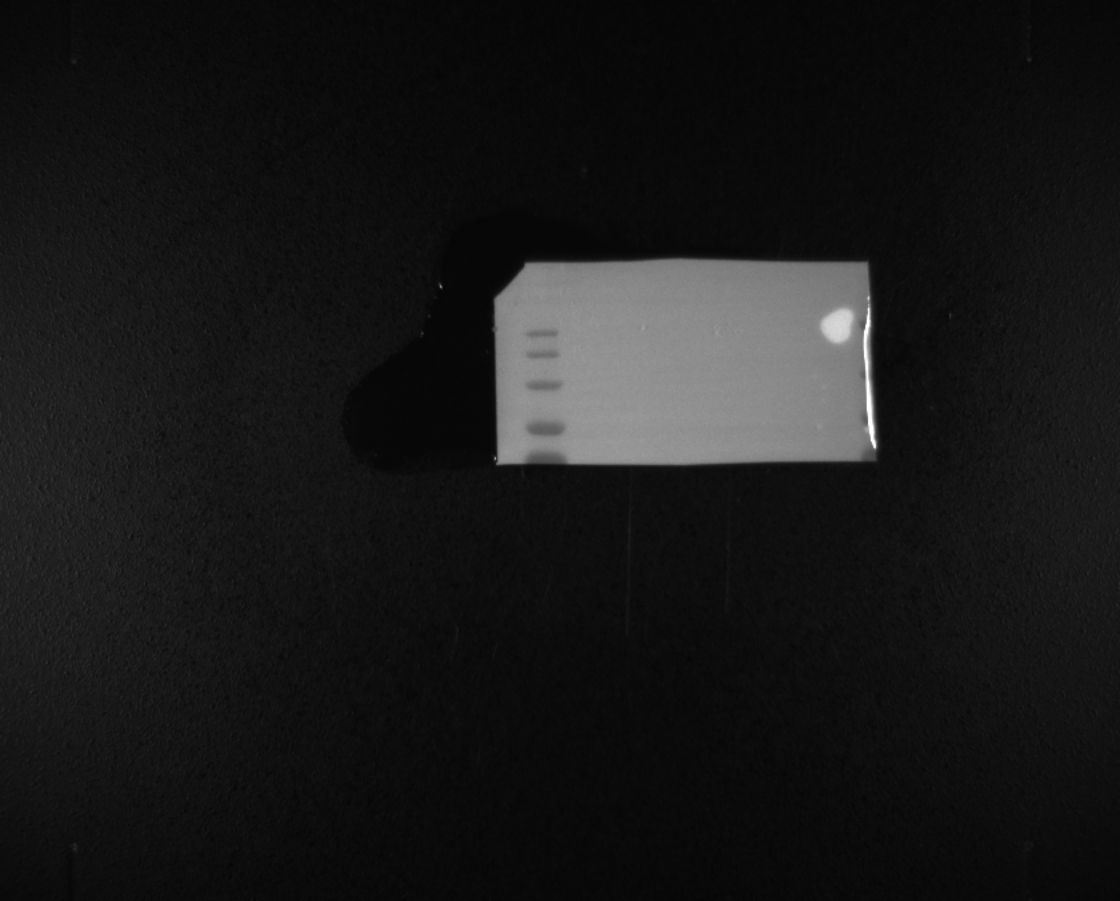

Supplement: Supplementary file 4 — Source Data [file 41467_2023_41520_MOESM4_ESM.zip › Source Data/Uncropped and Unprocessed Scans/Fig. S1c/IB SUMO1 - Marker.tif]

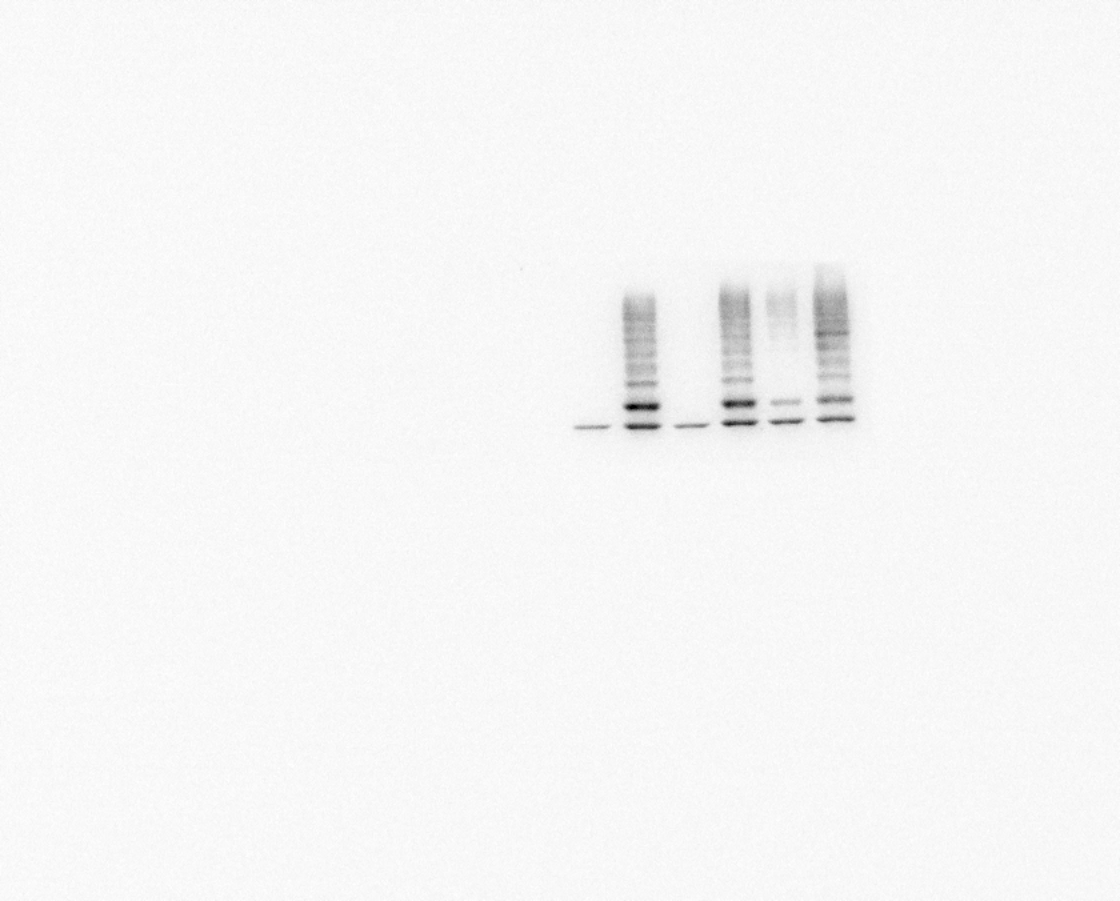

Supplement: Supplementary file 4 — Source Data [file 41467_2023_41520_MOESM4_ESM.zip › Source Data/Uncropped and Unprocessed Scans/Fig. S1c/IB SUMO1.tif]

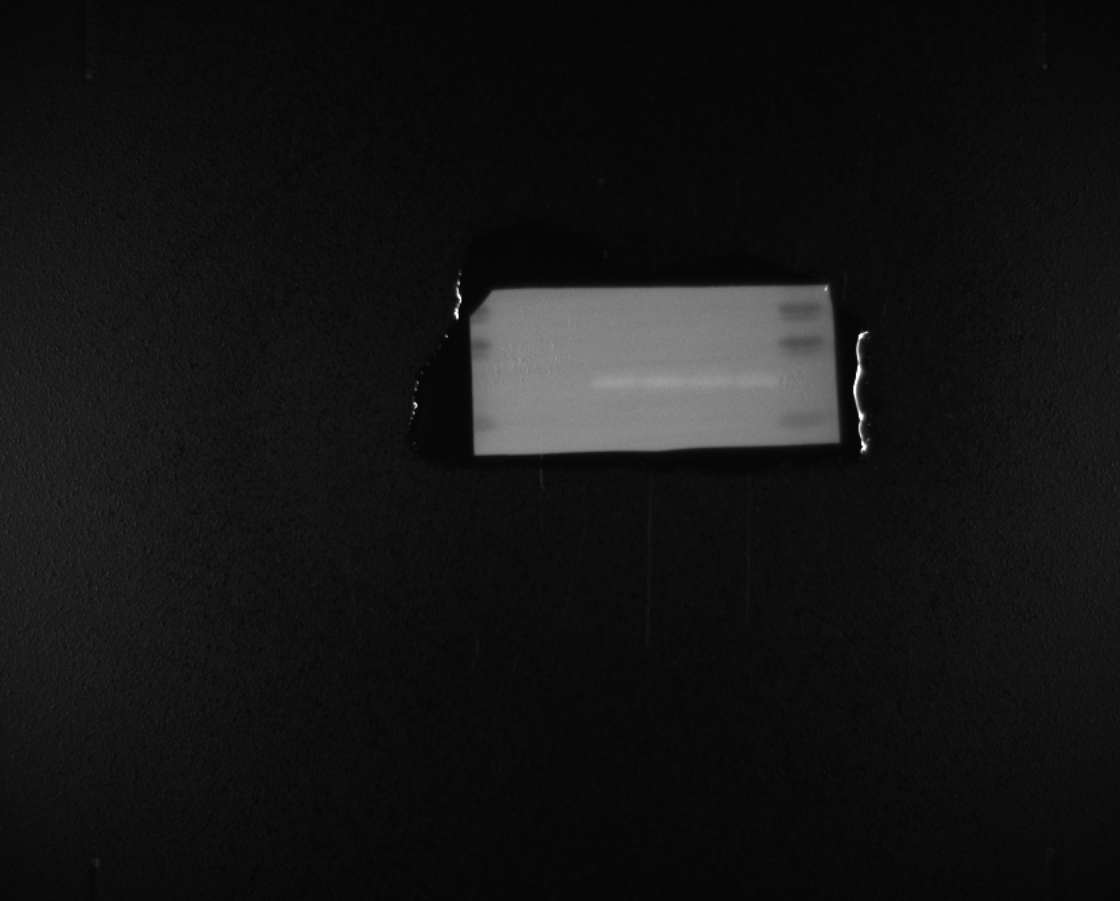

Supplement: Supplementary file 4 — Source Data [file 41467_2023_41520_MOESM4_ESM.zip › Source Data/Uncropped and Unprocessed Scans/Fig. S1c/IP HA; IB HA - Marker.tif]

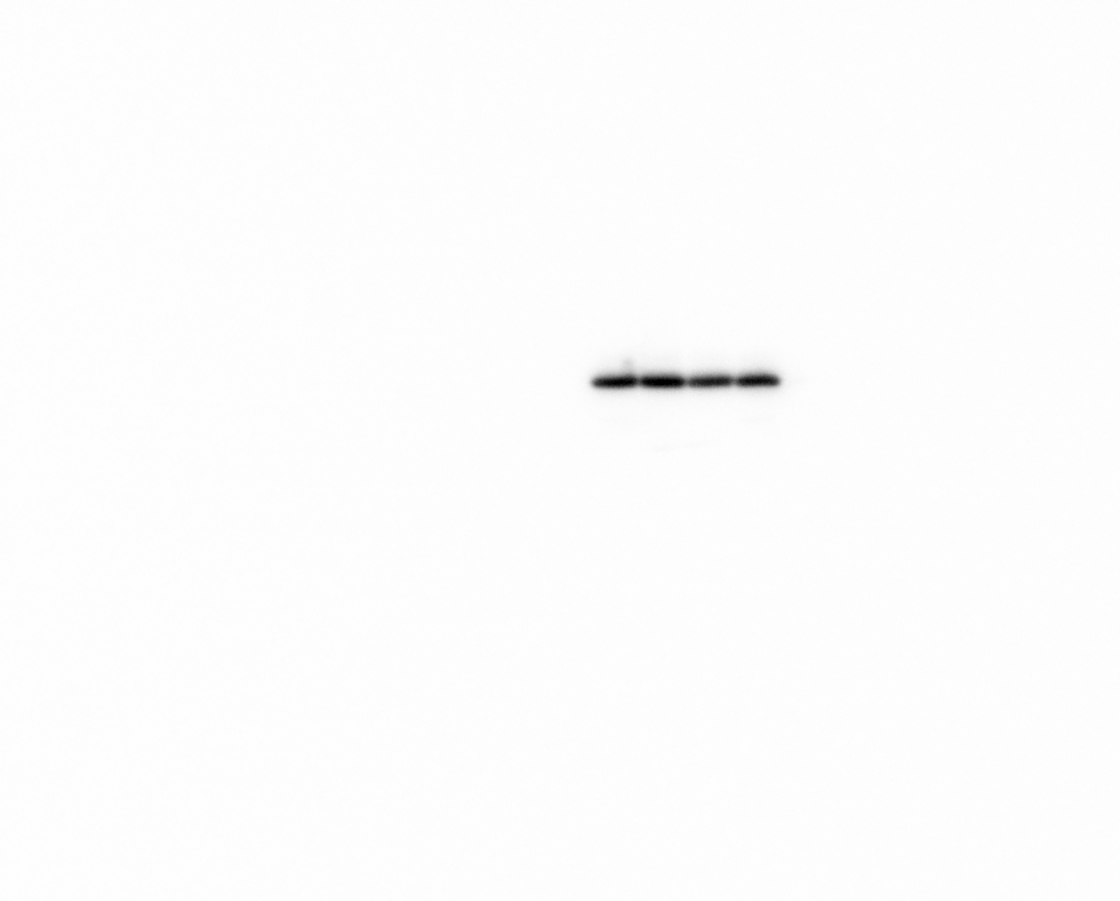

Supplement: Supplementary file 4 — Source Data [file 41467_2023_41520_MOESM4_ESM.zip › Source Data/Uncropped and Unprocessed Scans/Fig. S1c/IP HA; IB HA.tif]

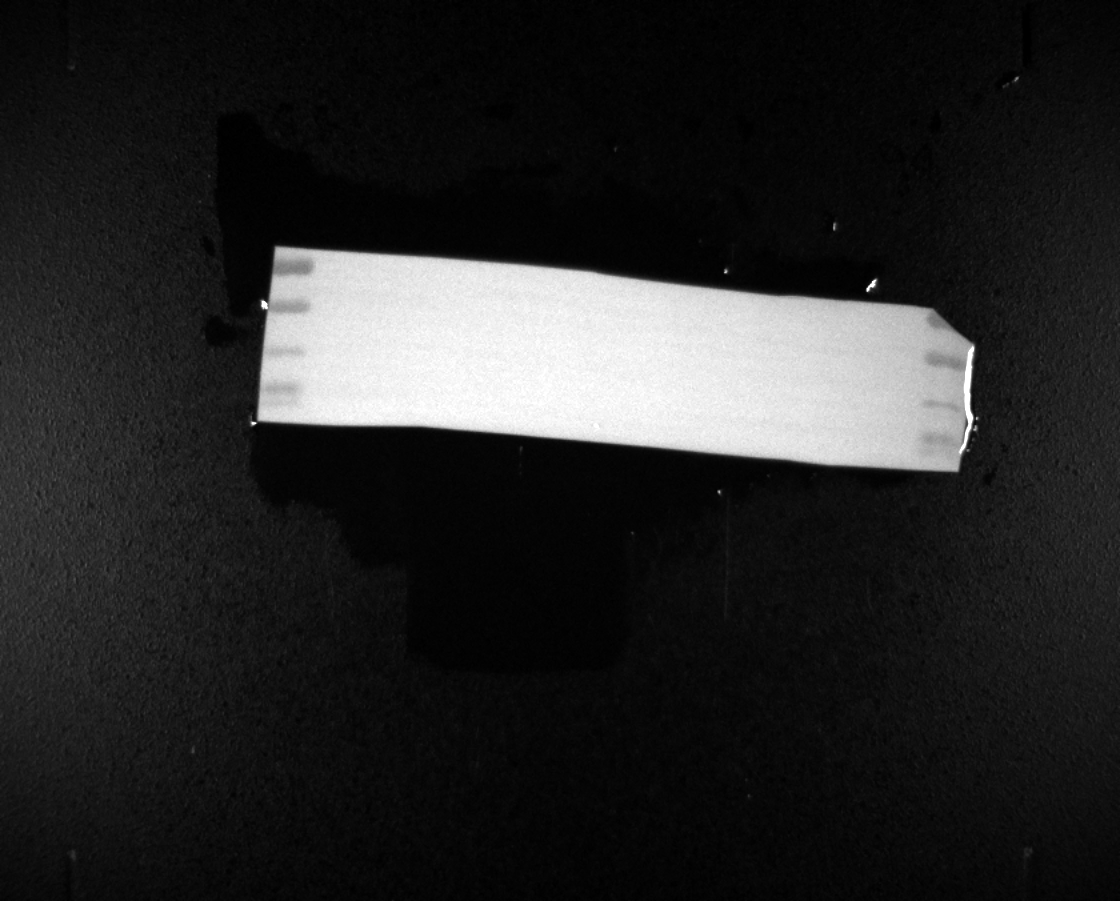

Supplement: Supplementary file 4 — Source Data [file 41467_2023_41520_MOESM4_ESM.zip › Source Data/Uncropped and Unprocessed Scans/Fig. S2b/Left/IB GAPDH - Marker.tif]

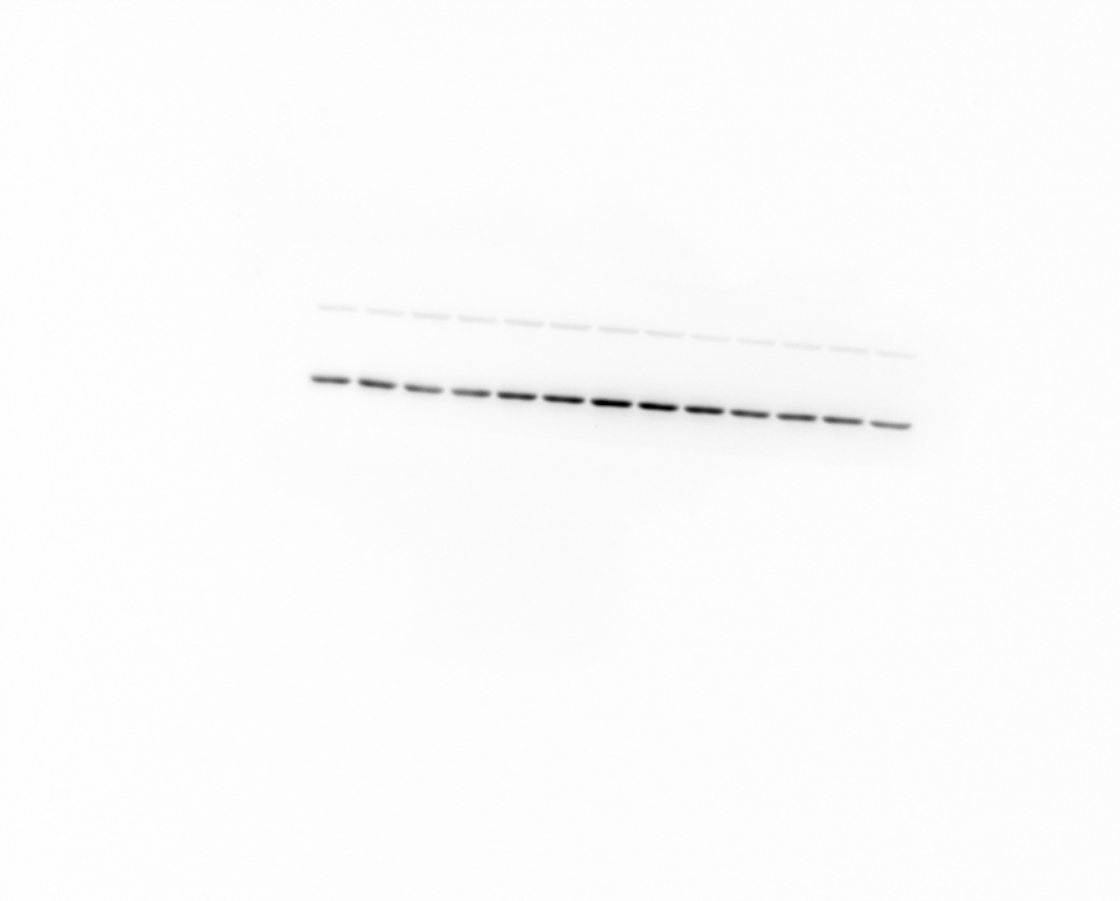

Supplement: Supplementary file 4 — Source Data [file 41467_2023_41520_MOESM4_ESM.zip › Source Data/Uncropped and Unprocessed Scans/Fig. S2b/Left/IB GAPDH.tif]

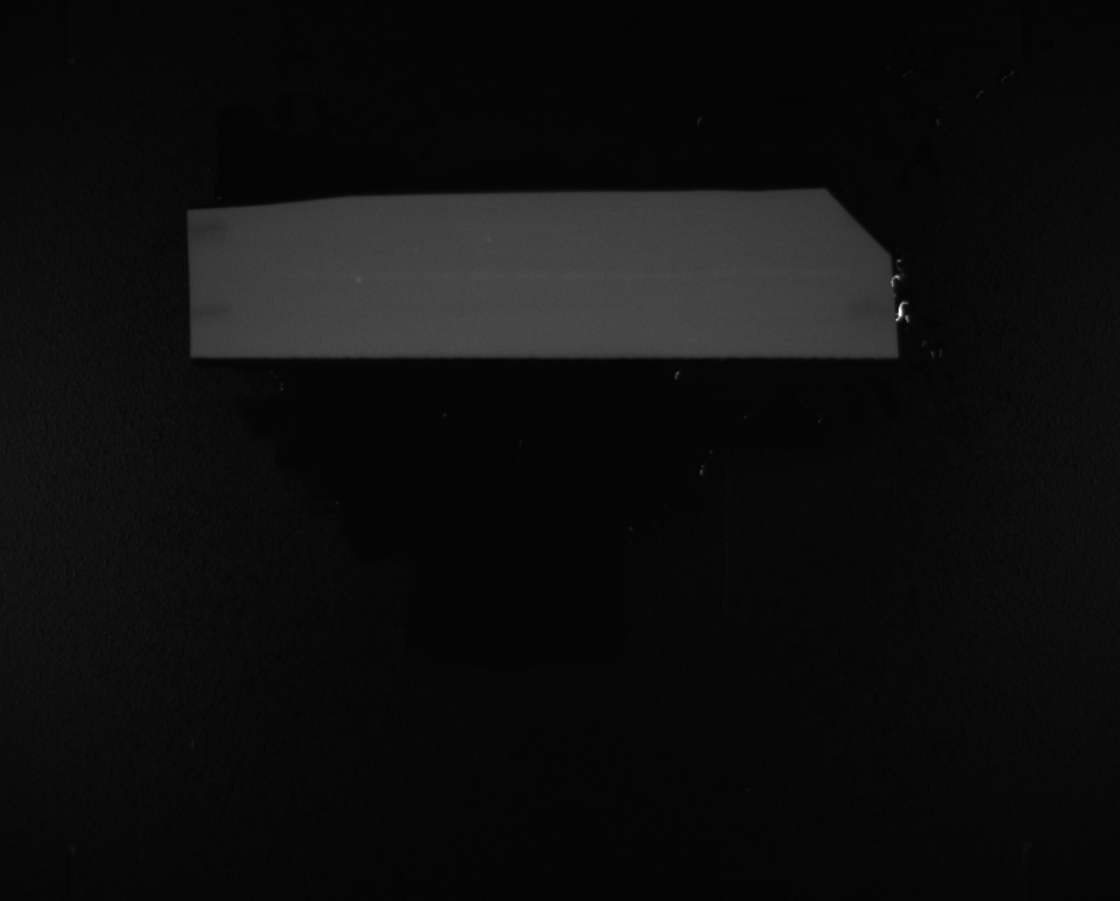

Supplement: Supplementary file 4 — Source Data [file 41467_2023_41520_MOESM4_ESM.zip › Source Data/Uncropped and Unprocessed Scans/Fig. S2b/Left/IB HA - Marker.tif]

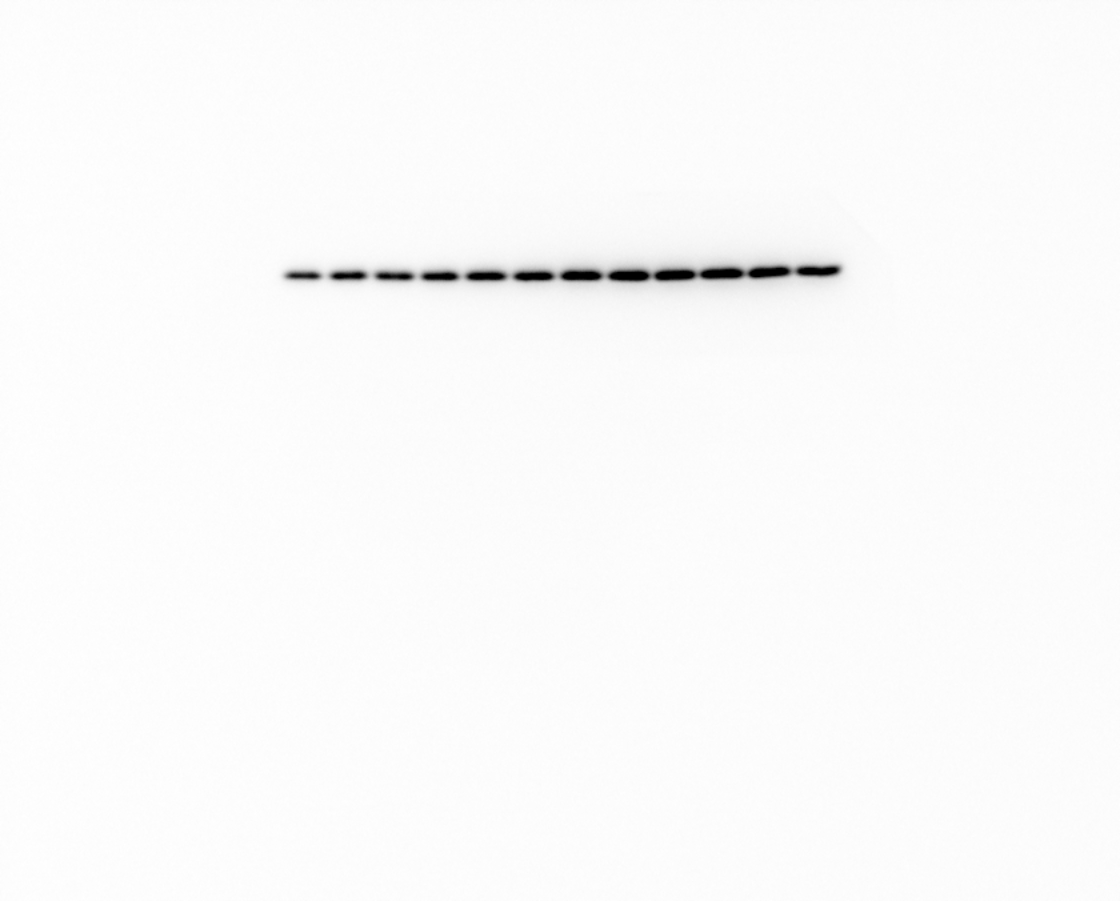

Supplement: Supplementary file 4 — Source Data [file 41467_2023_41520_MOESM4_ESM.zip › Source Data/Uncropped and Unprocessed Scans/Fig. S2b/Left/IB HA.tif]

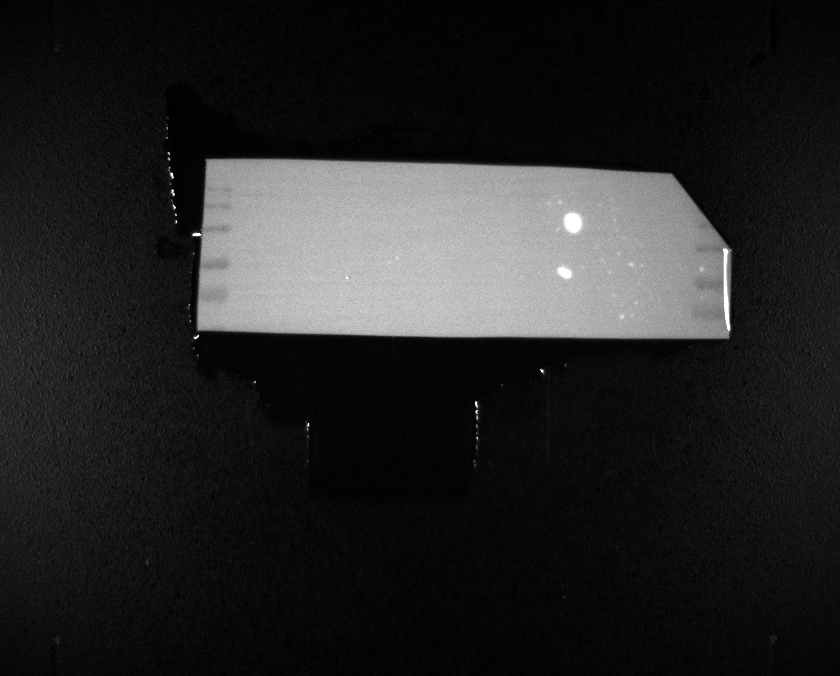

Supplement: Supplementary file 4 — Source Data [file 41467_2023_41520_MOESM4_ESM.zip › Source Data/Uncropped and Unprocessed Scans/Fig. S2b/Left/IB SUMO1 - Marker.tif]

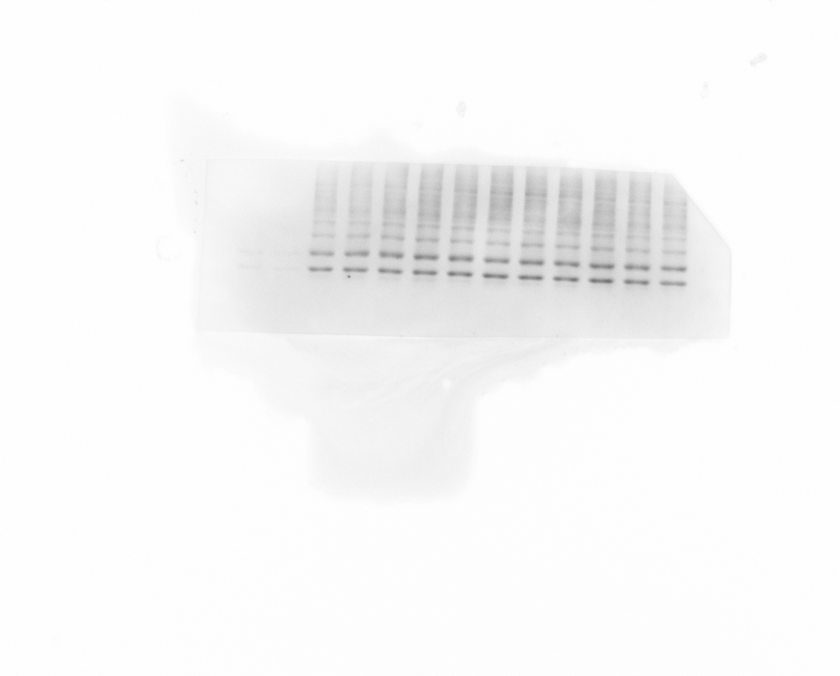

Supplement: Supplementary file 4 — Source Data [file 41467_2023_41520_MOESM4_ESM.zip › Source Data/Uncropped and Unprocessed Scans/Fig. S2b/Left/IB SUMO1.tif]

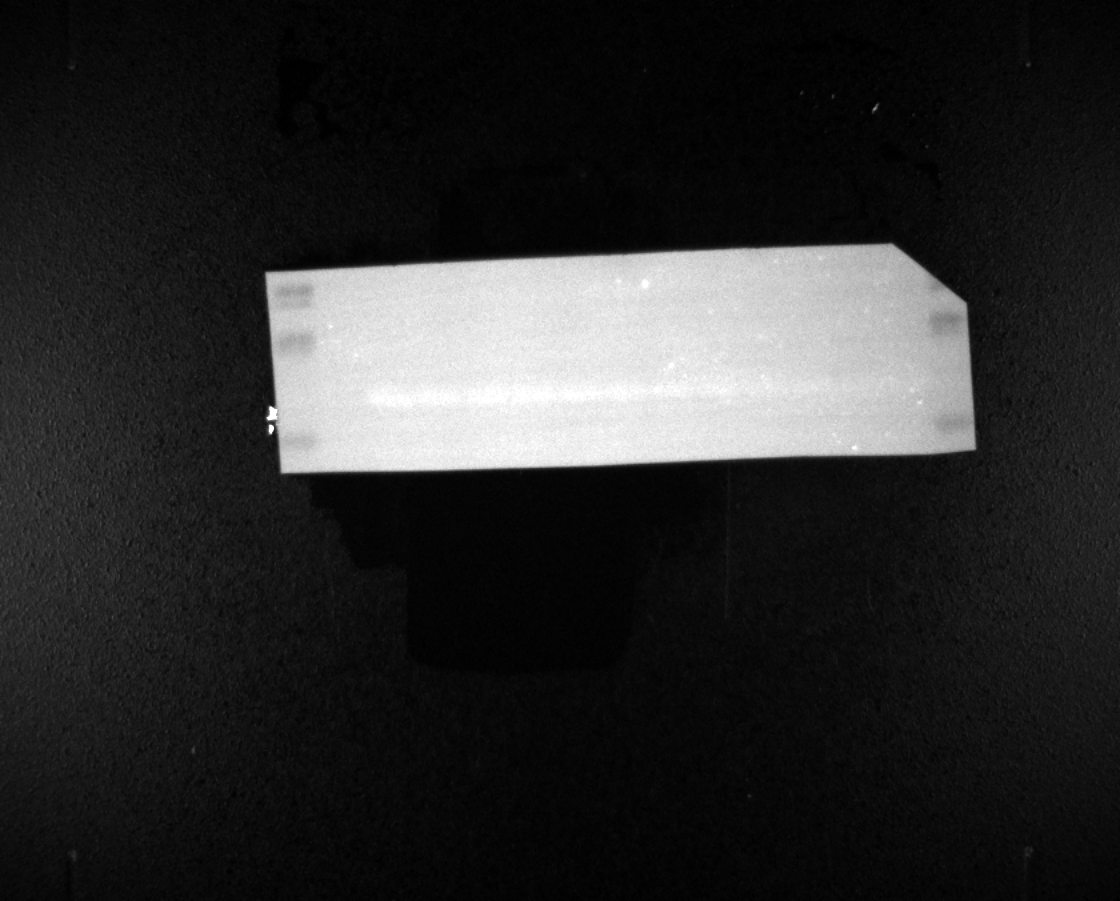

Supplement: Supplementary file 4 — Source Data [file 41467_2023_41520_MOESM4_ESM.zip › Source Data/Uncropped and Unprocessed Scans/Fig. S2b/Left/IP HA; IB HA - Marker.tif]

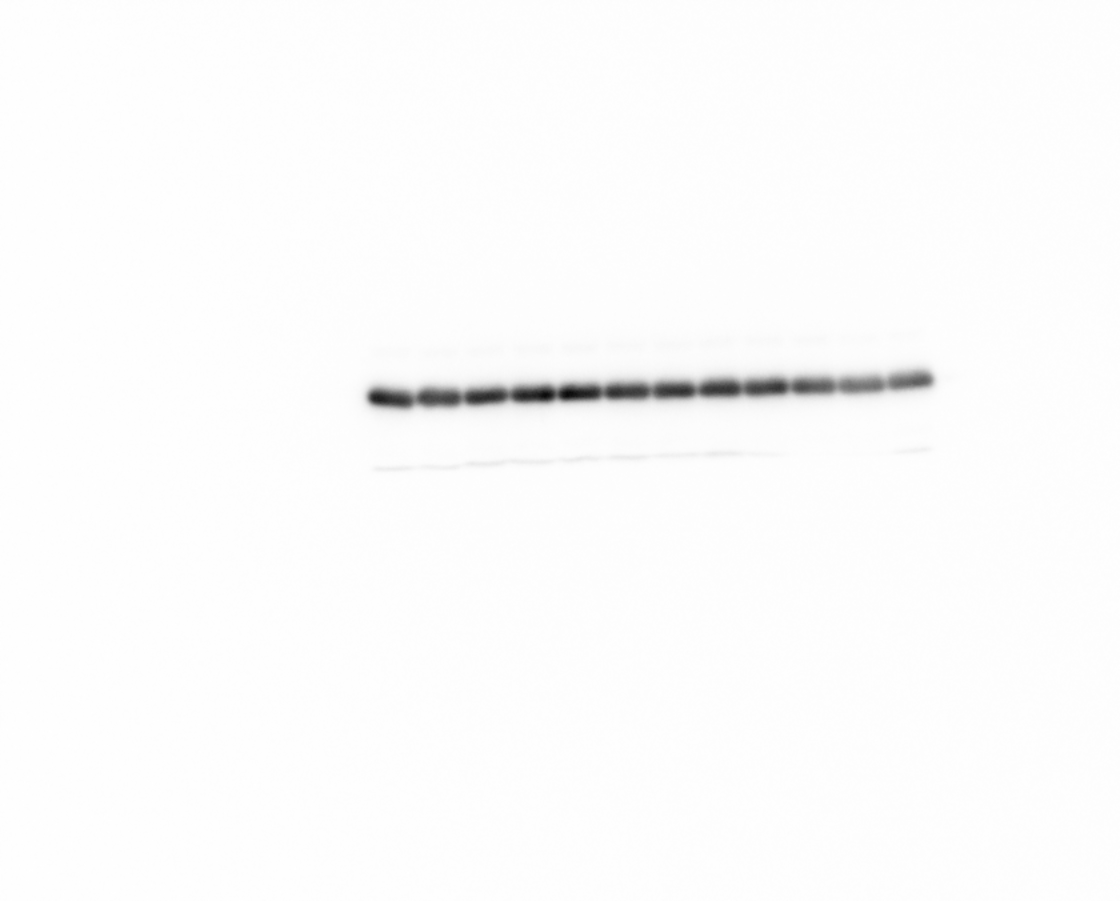

Supplement: Supplementary file 4 — Source Data [file 41467_2023_41520_MOESM4_ESM.zip › Source Data/Uncropped and Unprocessed Scans/Fig. S2b/Left/IP HA; IB HA.tif]

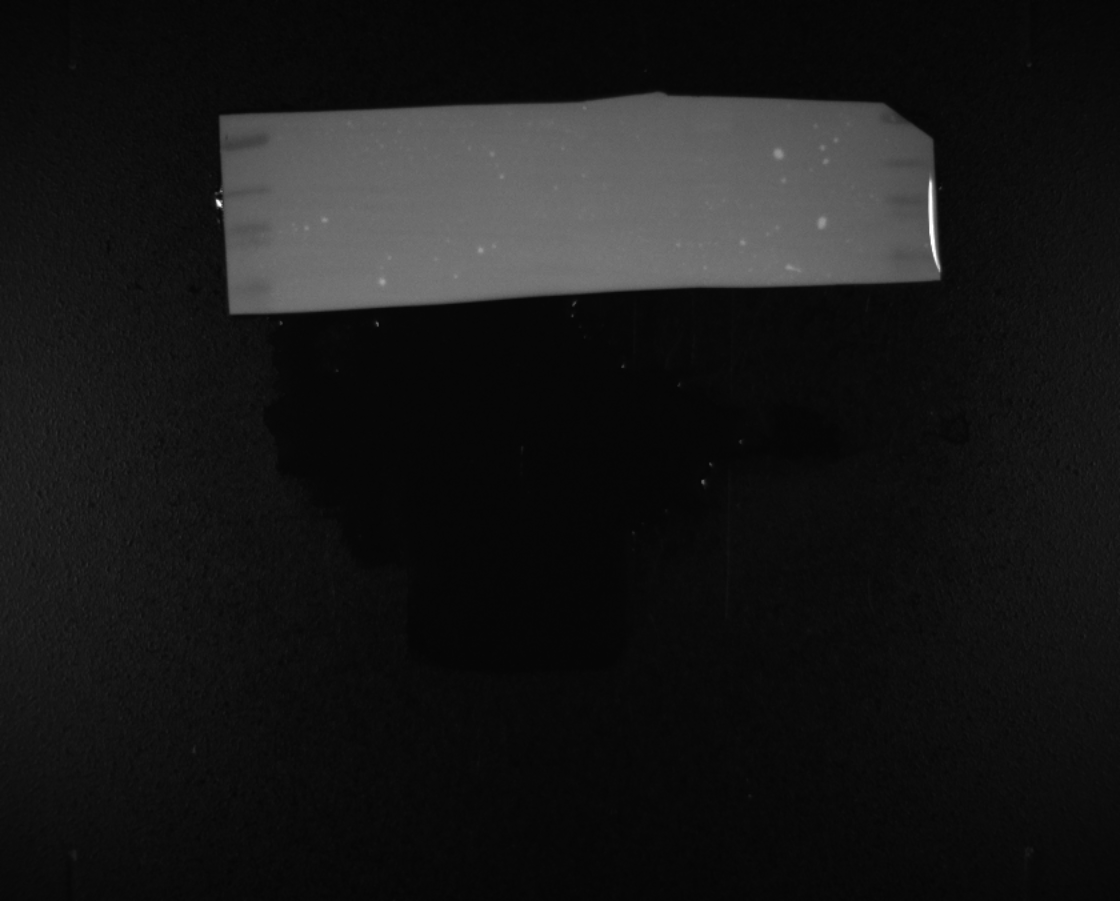

Supplement: Supplementary file 4 — Source Data [file 41467_2023_41520_MOESM4_ESM.zip › Source Data/Uncropped and Unprocessed Scans/Fig. S2b/Left/IP HA; IB SUMO1 - Marker.tif]

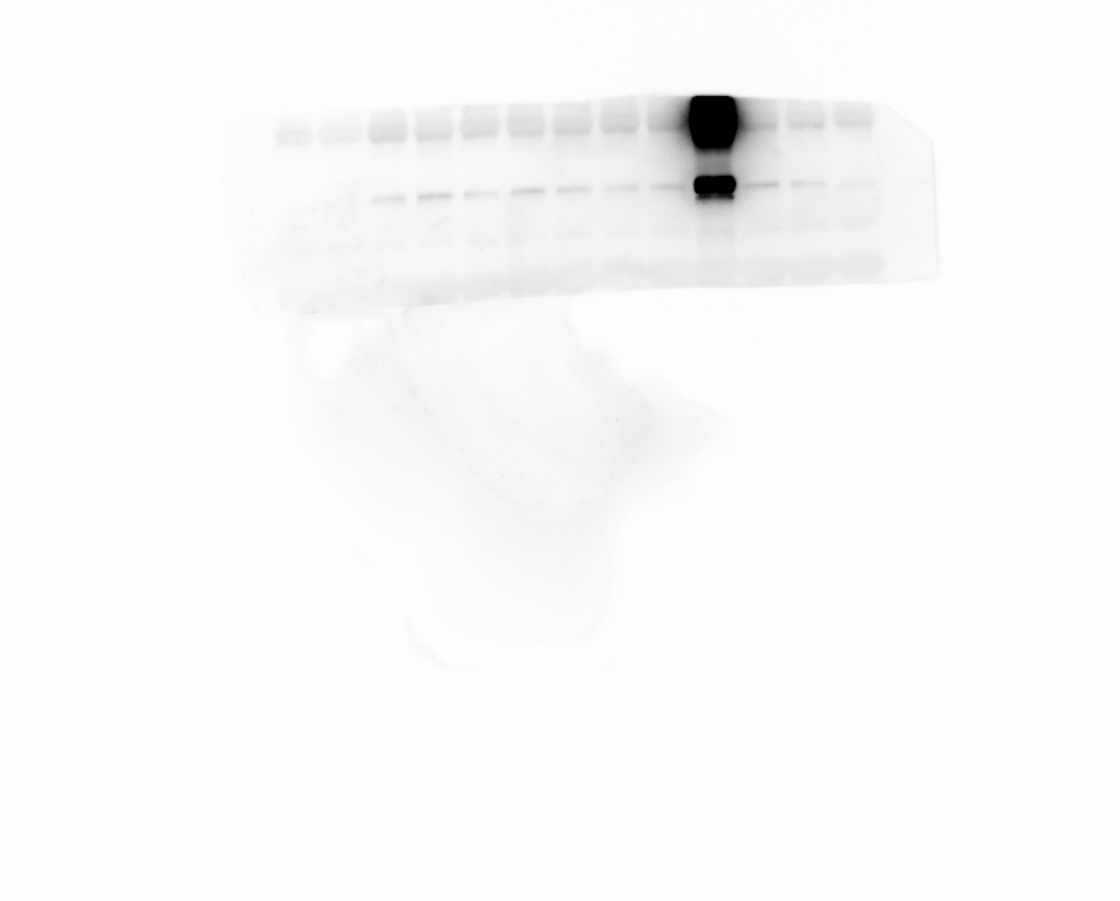

Supplement: Supplementary file 4 — Source Data [file 41467_2023_41520_MOESM4_ESM.zip › Source Data/Uncropped and Unprocessed Scans/Fig. S2b/Left/IP HA; IB SUMO1.tif]

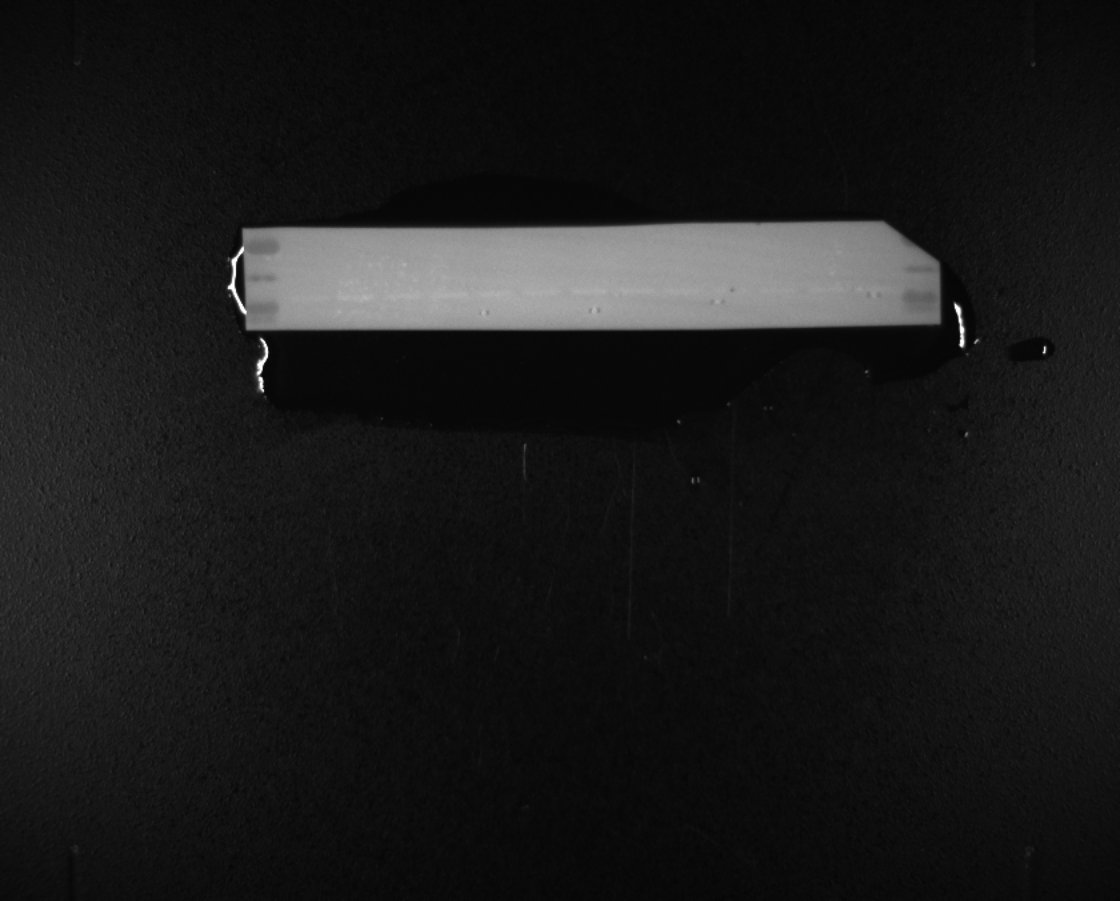

Supplement: Supplementary file 4 — Source Data [file 41467_2023_41520_MOESM4_ESM.zip › Source Data/Uncropped and Unprocessed Scans/Fig. S2b/Middle/IB GAPDH - Marker.tif]

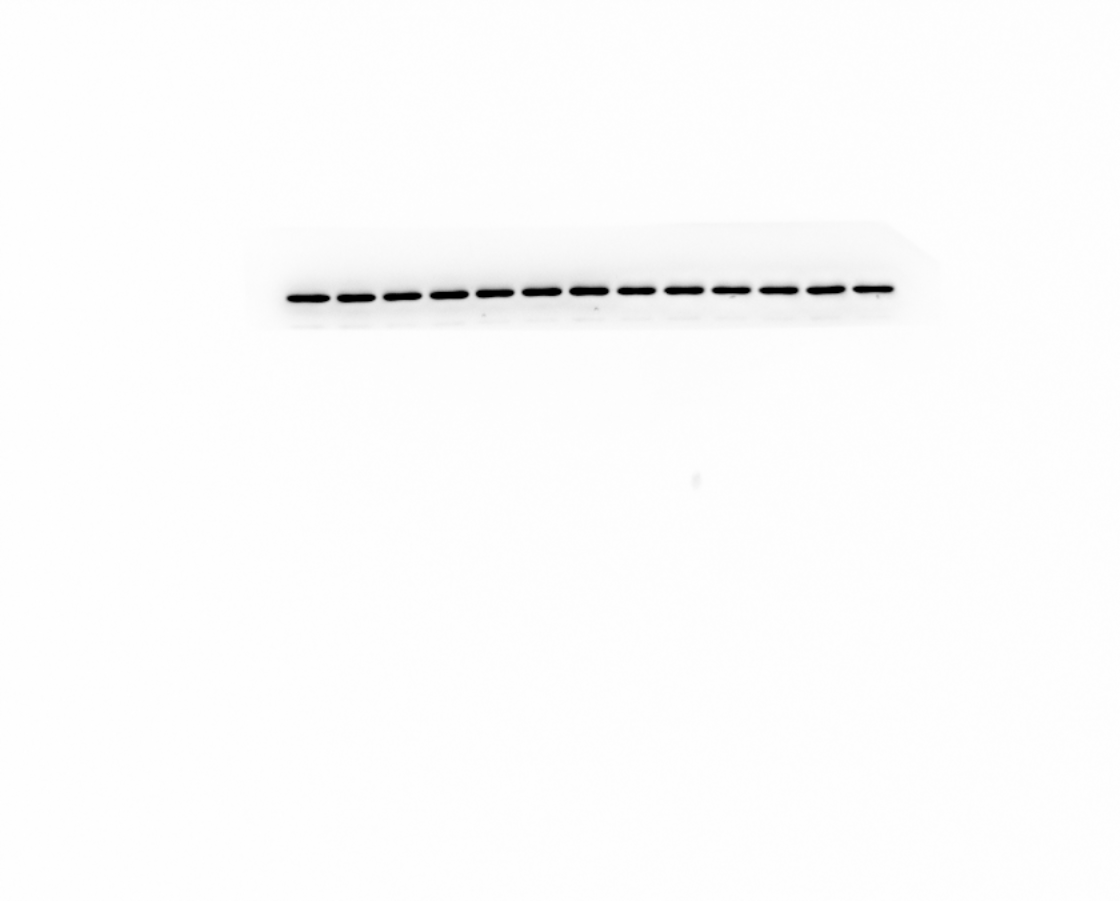

Supplement: Supplementary file 4 — Source Data [file 41467_2023_41520_MOESM4_ESM.zip › Source Data/Uncropped and Unprocessed Scans/Fig. S2b/Middle/IB GAPDH.tif]

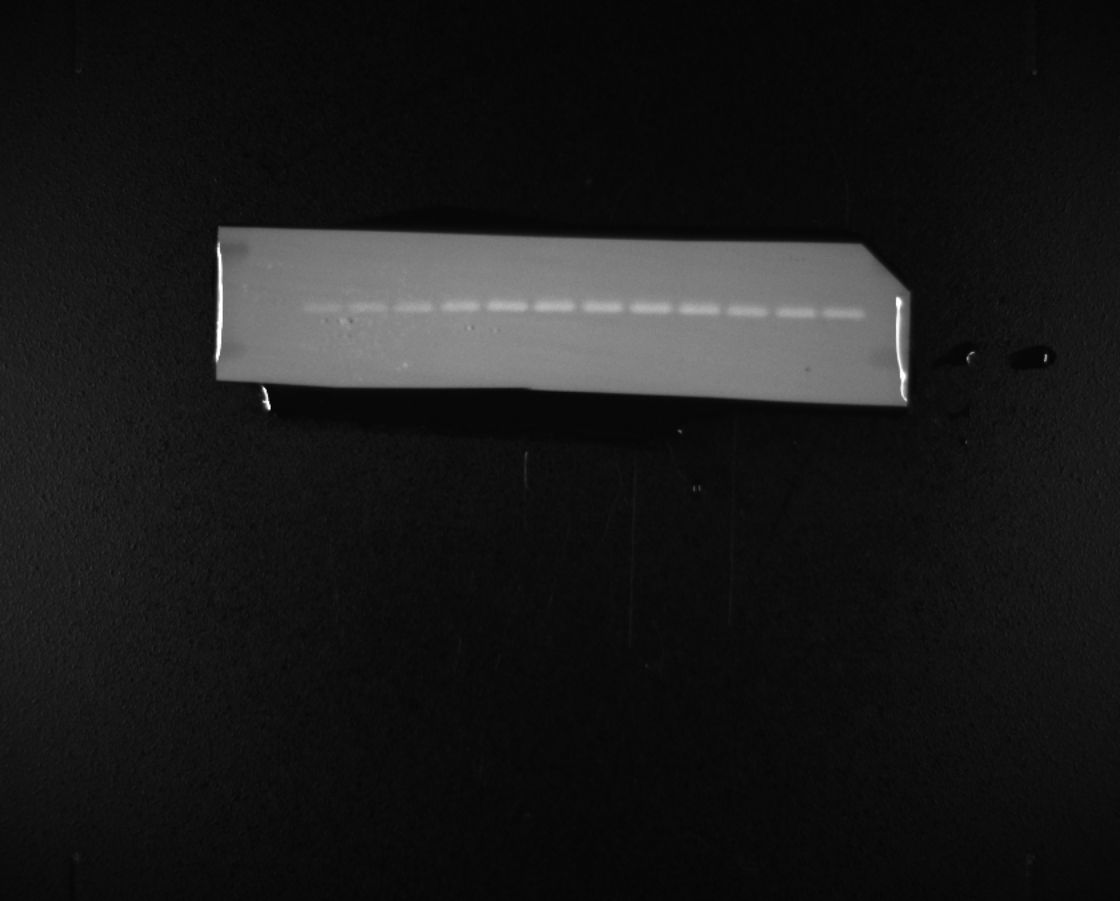

Supplement: Supplementary file 4 — Source Data [file 41467_2023_41520_MOESM4_ESM.zip › Source Data/Uncropped and Unprocessed Scans/Fig. S2b/Middle/IB HA - Marker.tif]

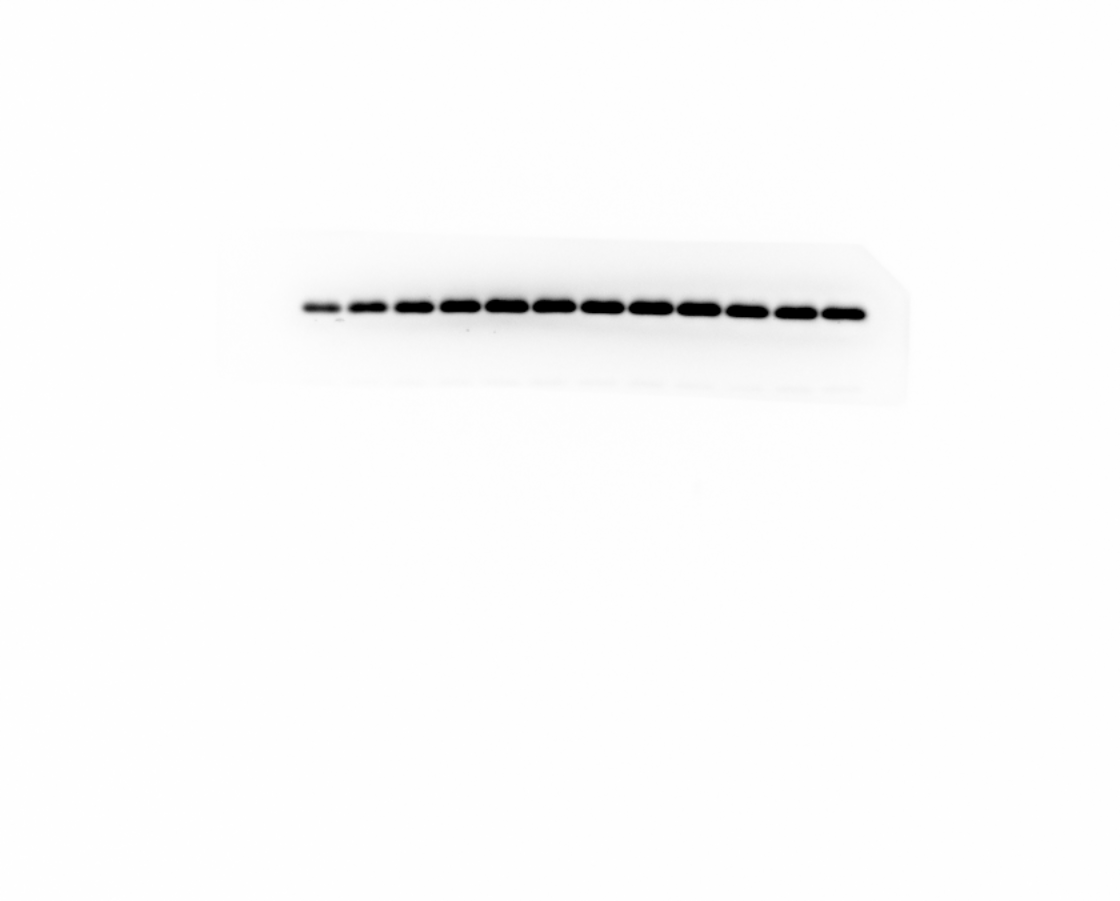

Supplement: Supplementary file 4 — Source Data [file 41467_2023_41520_MOESM4_ESM.zip › Source Data/Uncropped and Unprocessed Scans/Fig. S2b/Middle/IB HA.tif]

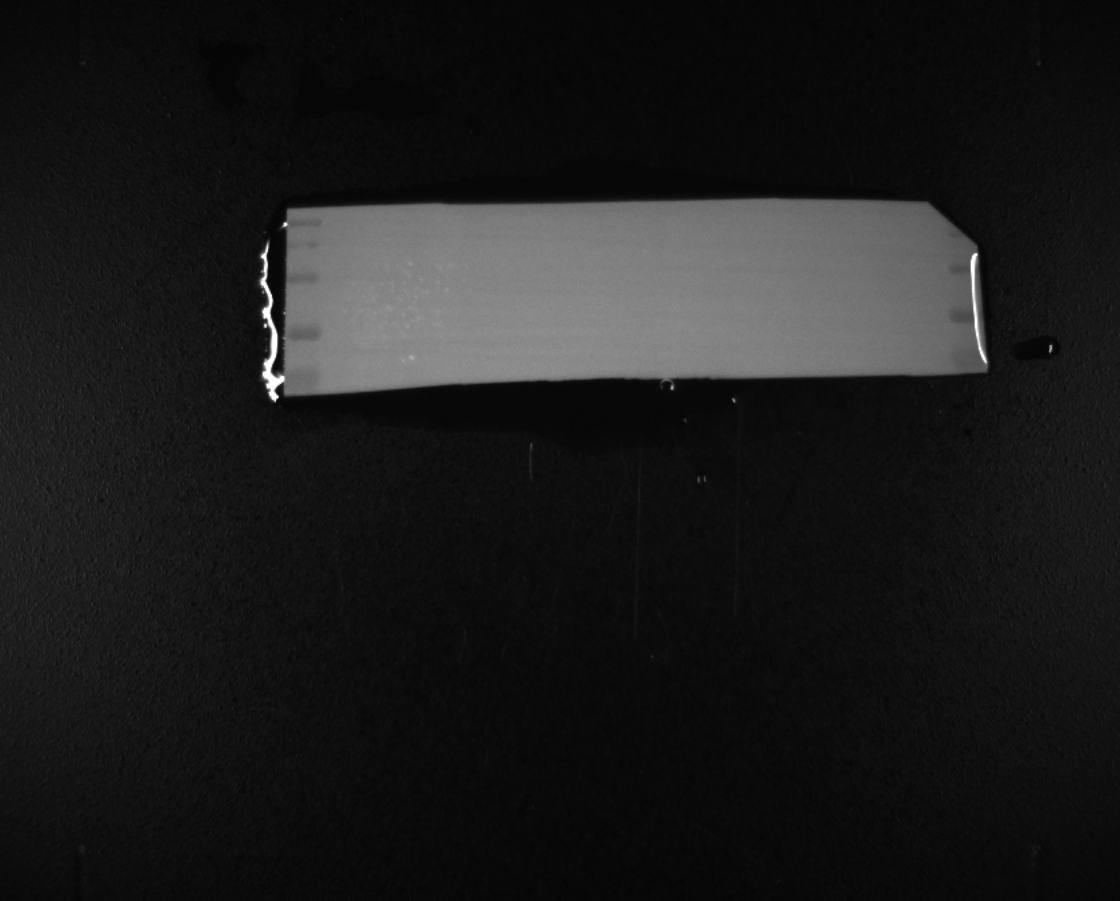

Supplement: Supplementary file 4 — Source Data [file 41467_2023_41520_MOESM4_ESM.zip › Source Data/Uncropped and Unprocessed Scans/Fig. S2b/Middle/IB SUMO1 - Marker.tif]

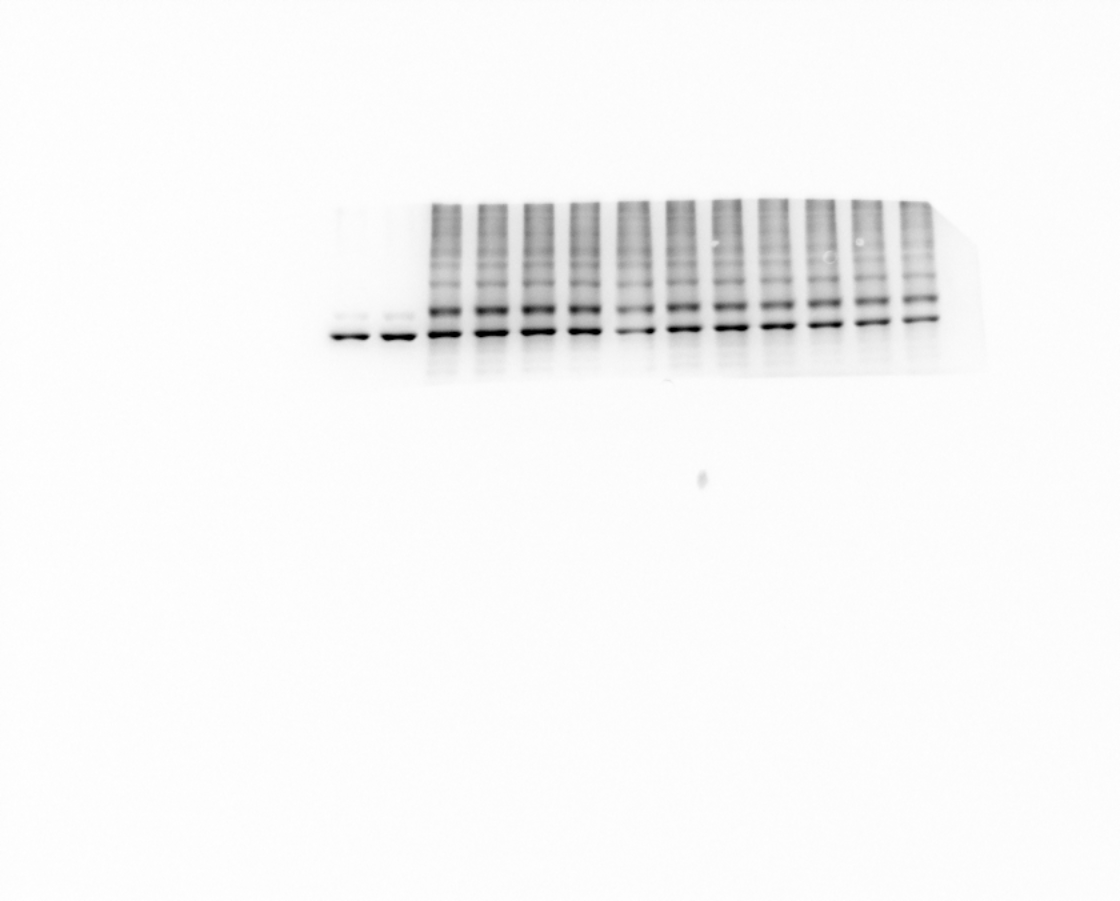

Supplement: Supplementary file 4 — Source Data [file 41467_2023_41520_MOESM4_ESM.zip › Source Data/Uncropped and Unprocessed Scans/Fig. S2b/Middle/IB SUMO1.tif]

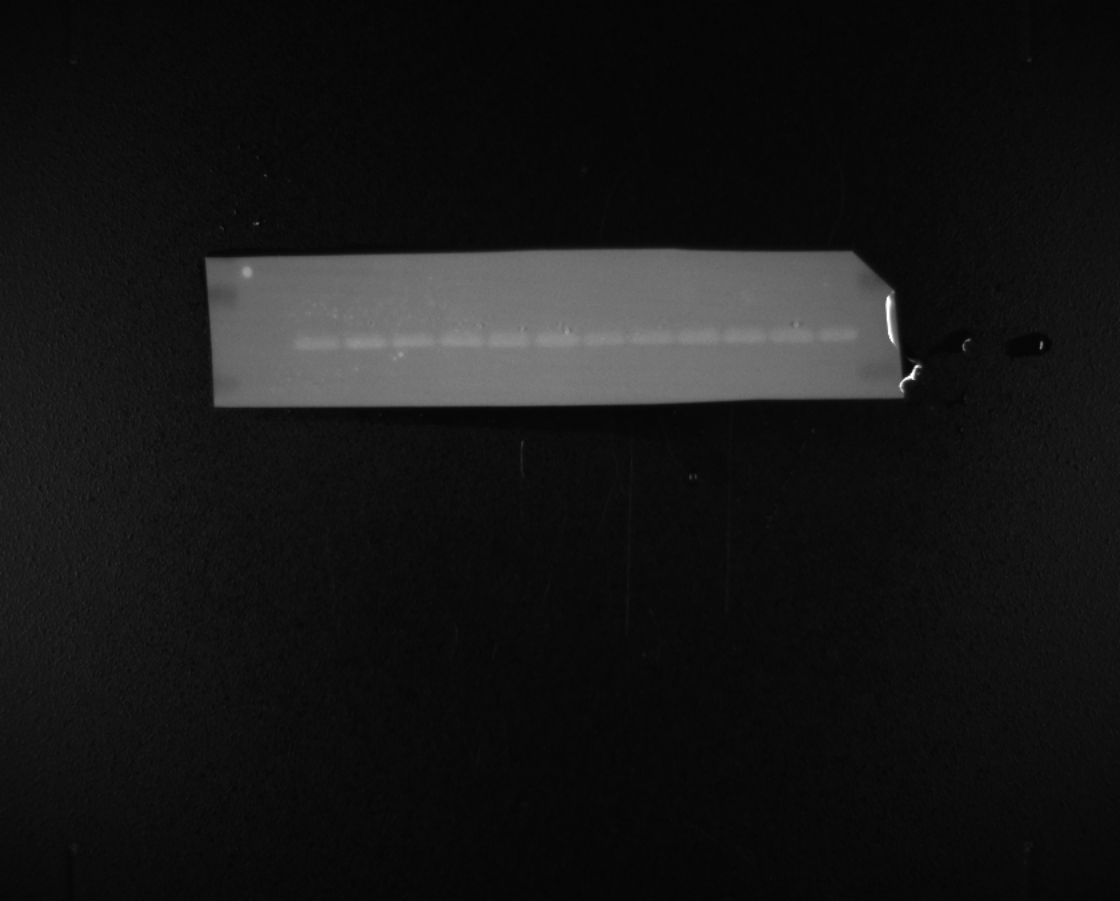

Supplement: Supplementary file 4 — Source Data [file 41467_2023_41520_MOESM4_ESM.zip › Source Data/Uncropped and Unprocessed Scans/Fig. S2b/Middle/IP HA; IB HA - Marker.tif]

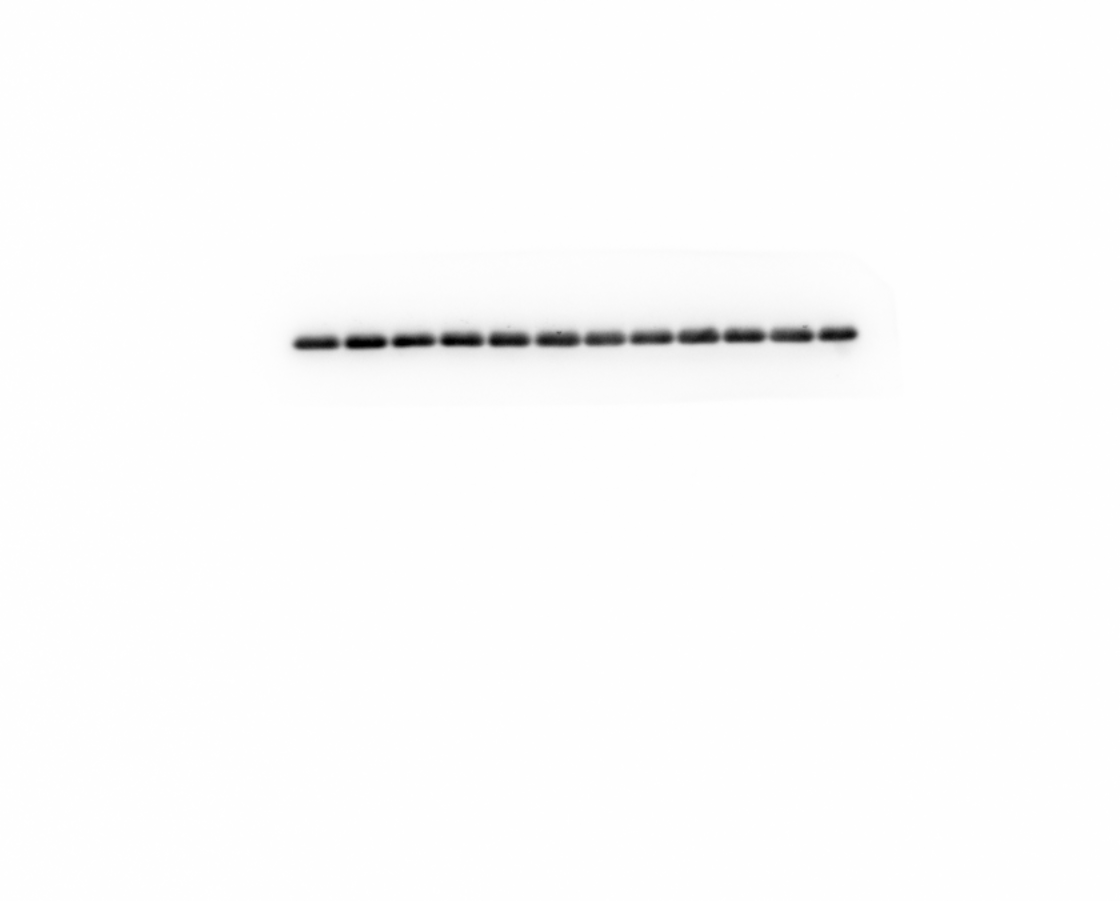

Supplement: Supplementary file 4 — Source Data [file 41467_2023_41520_MOESM4_ESM.zip › Source Data/Uncropped and Unprocessed Scans/Fig. S2b/Middle/IP HA; IB HA.tif]

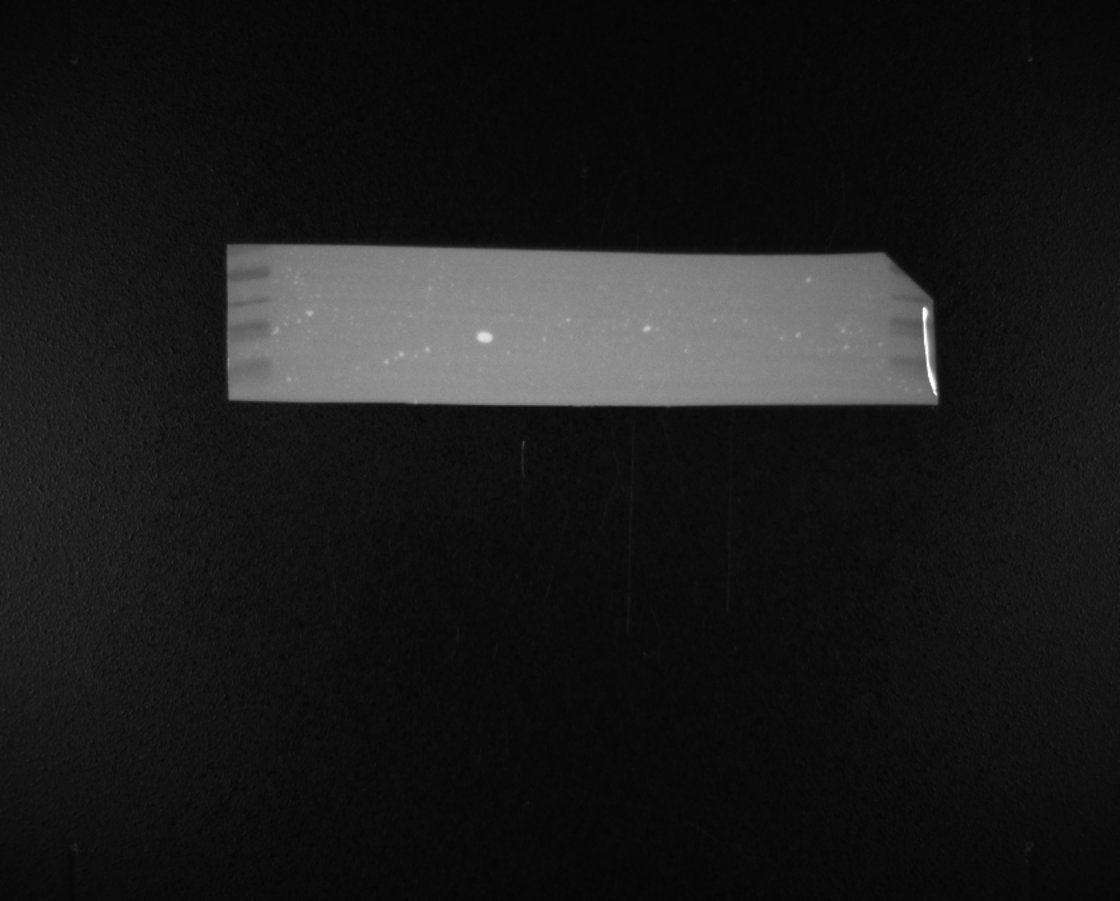

Supplement: Supplementary file 4 — Source Data [file 41467_2023_41520_MOESM4_ESM.zip › Source Data/Uncropped and Unprocessed Scans/Fig. S2b/Middle/IP SUMO1 - Marker.tif]

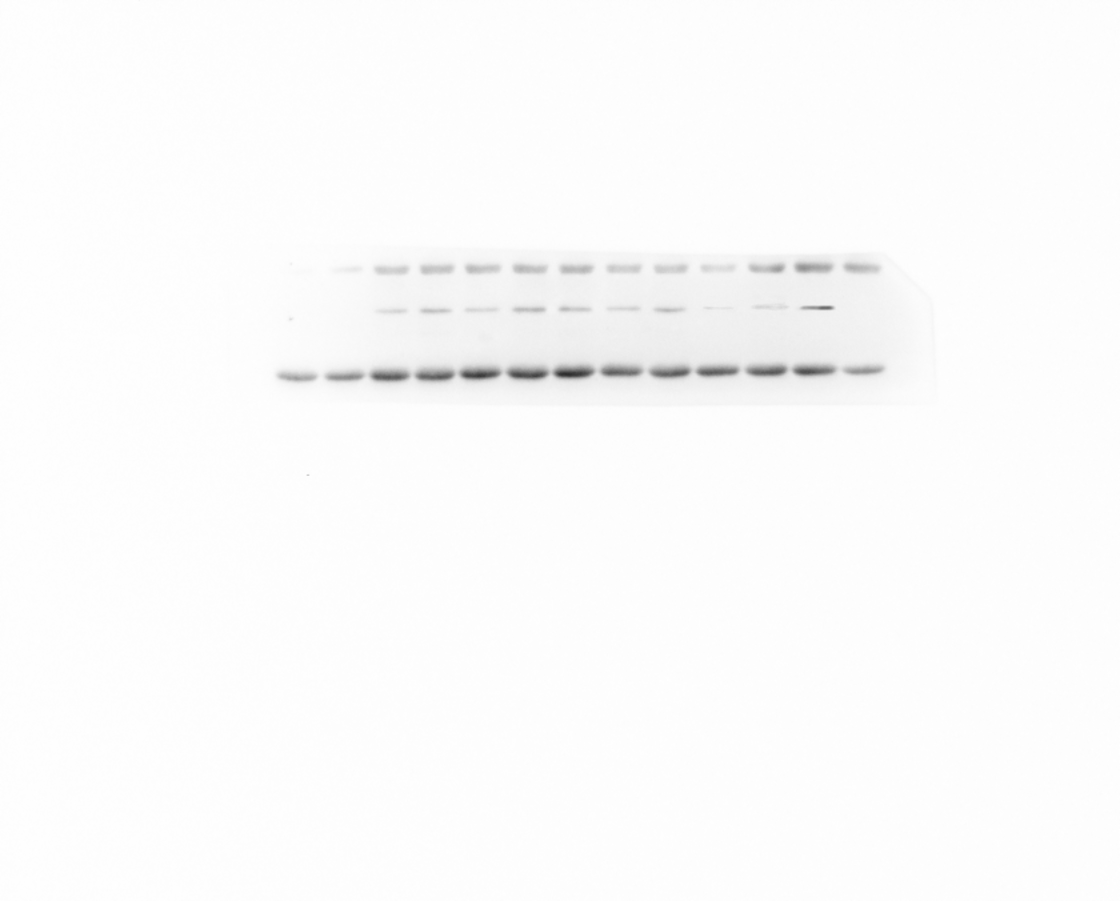

Supplement: Supplementary file 4 — Source Data [file 41467_2023_41520_MOESM4_ESM.zip › Source Data/Uncropped and Unprocessed Scans/Fig. S2b/Middle/IP SUMO1.tif]

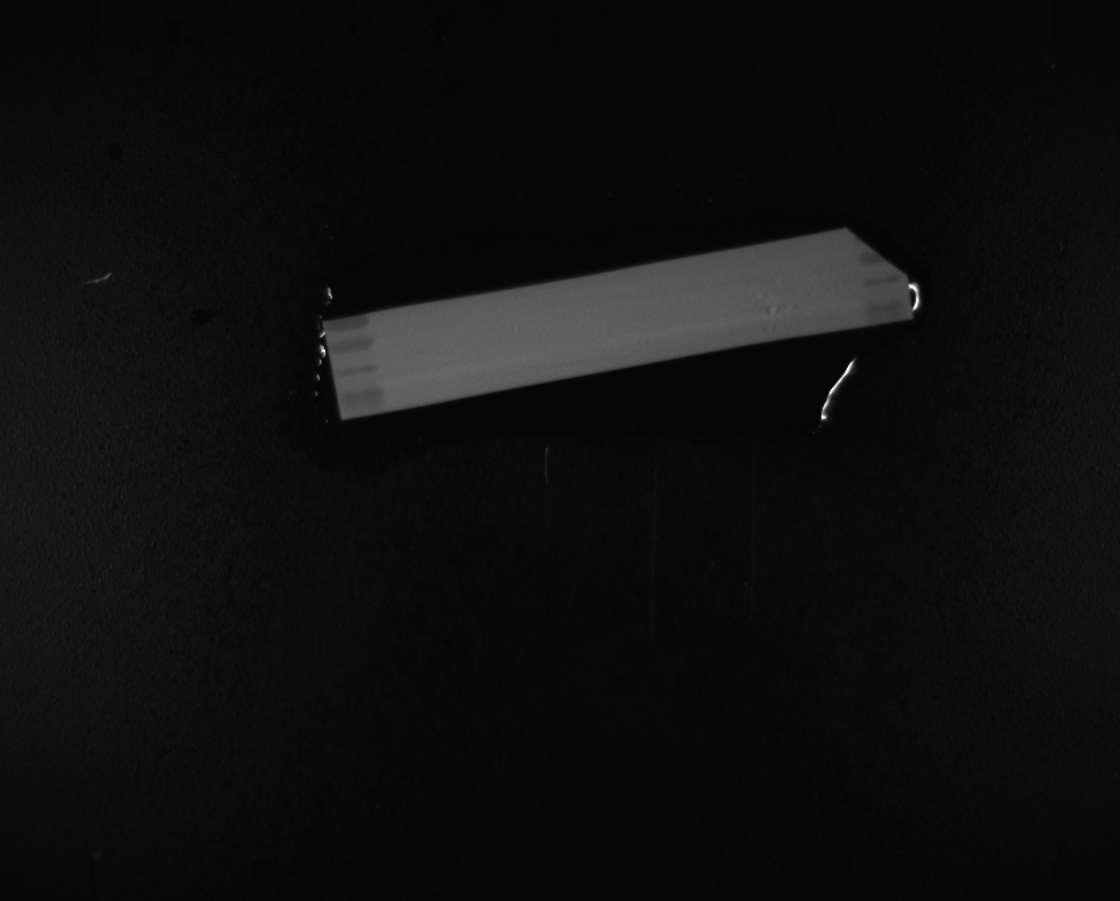

Supplement: Supplementary file 4 — Source Data [file 41467_2023_41520_MOESM4_ESM.zip › Source Data/Uncropped and Unprocessed Scans/Fig. S2b/Right/IB GAPDH - Marker.tif]

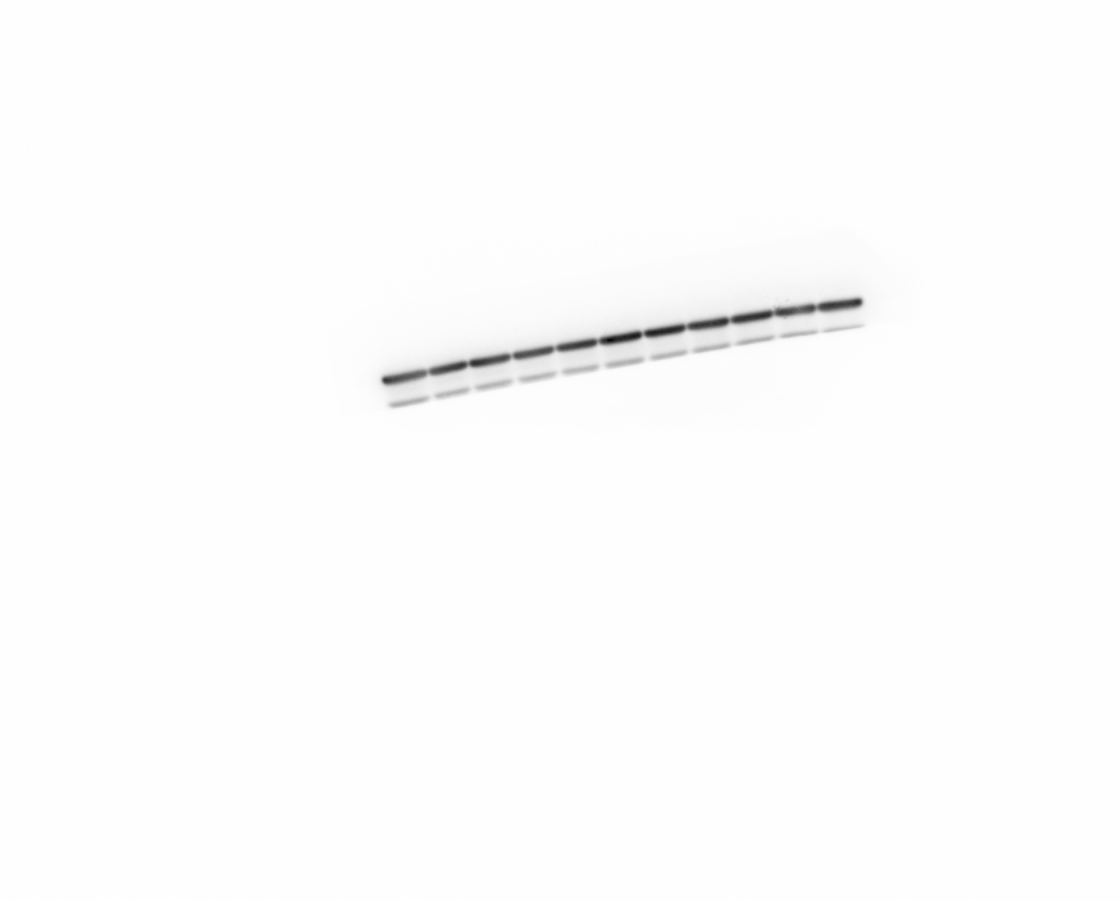

Supplement: Supplementary file 4 — Source Data [file 41467_2023_41520_MOESM4_ESM.zip › Source Data/Uncropped and Unprocessed Scans/Fig. S2b/Right/IB GAPDH.tif]

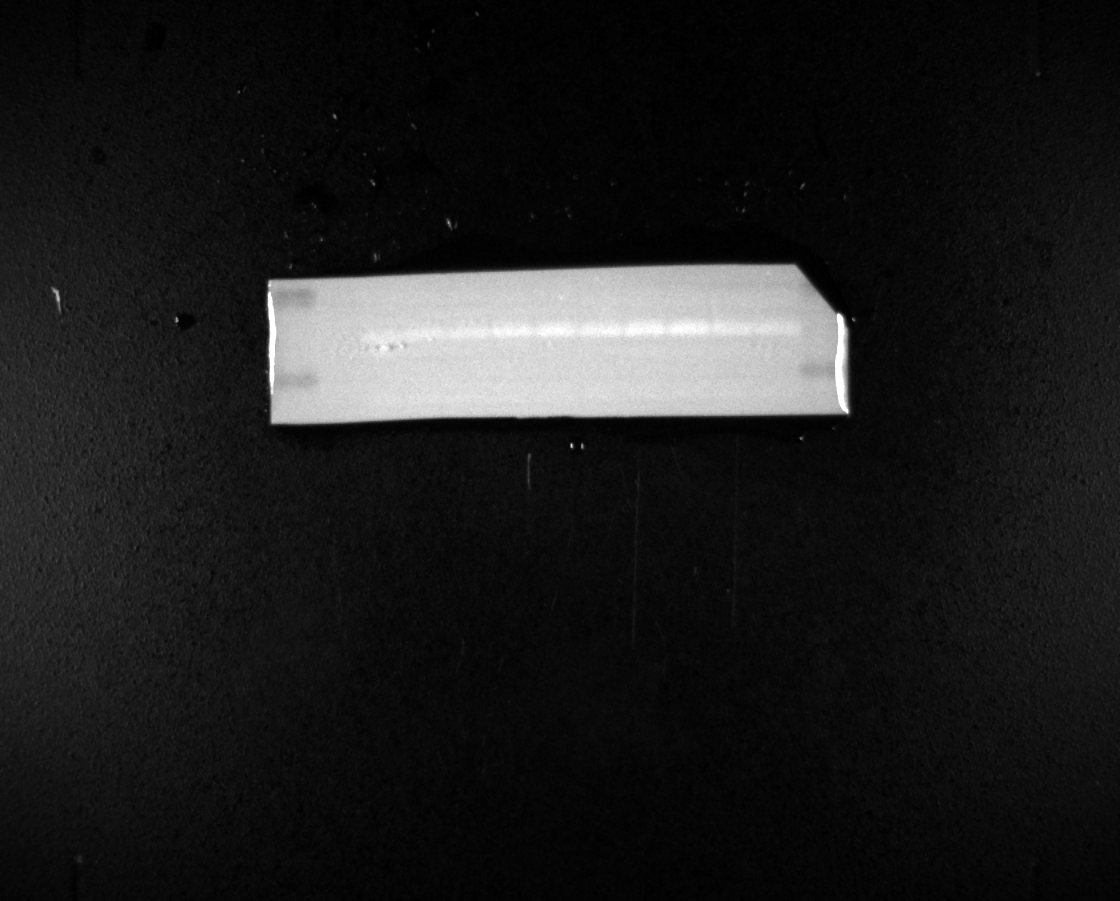

Supplement: Supplementary file 4 — Source Data [file 41467_2023_41520_MOESM4_ESM.zip › Source Data/Uncropped and Unprocessed Scans/Fig. S2b/Right/IB HA - Marker.tif]

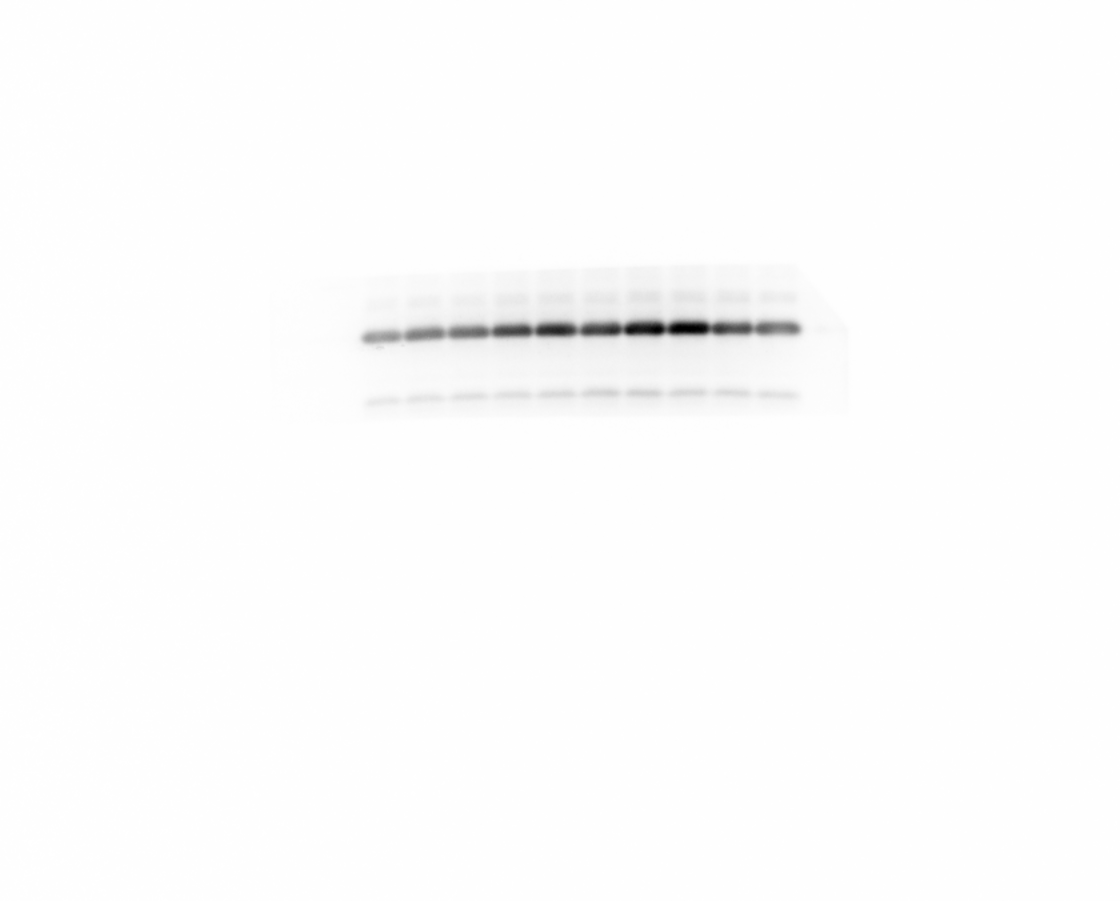

Supplement: Supplementary file 4 — Source Data [file 41467_2023_41520_MOESM4_ESM.zip › Source Data/Uncropped and Unprocessed Scans/Fig. S2b/Right/IB HA.tif]

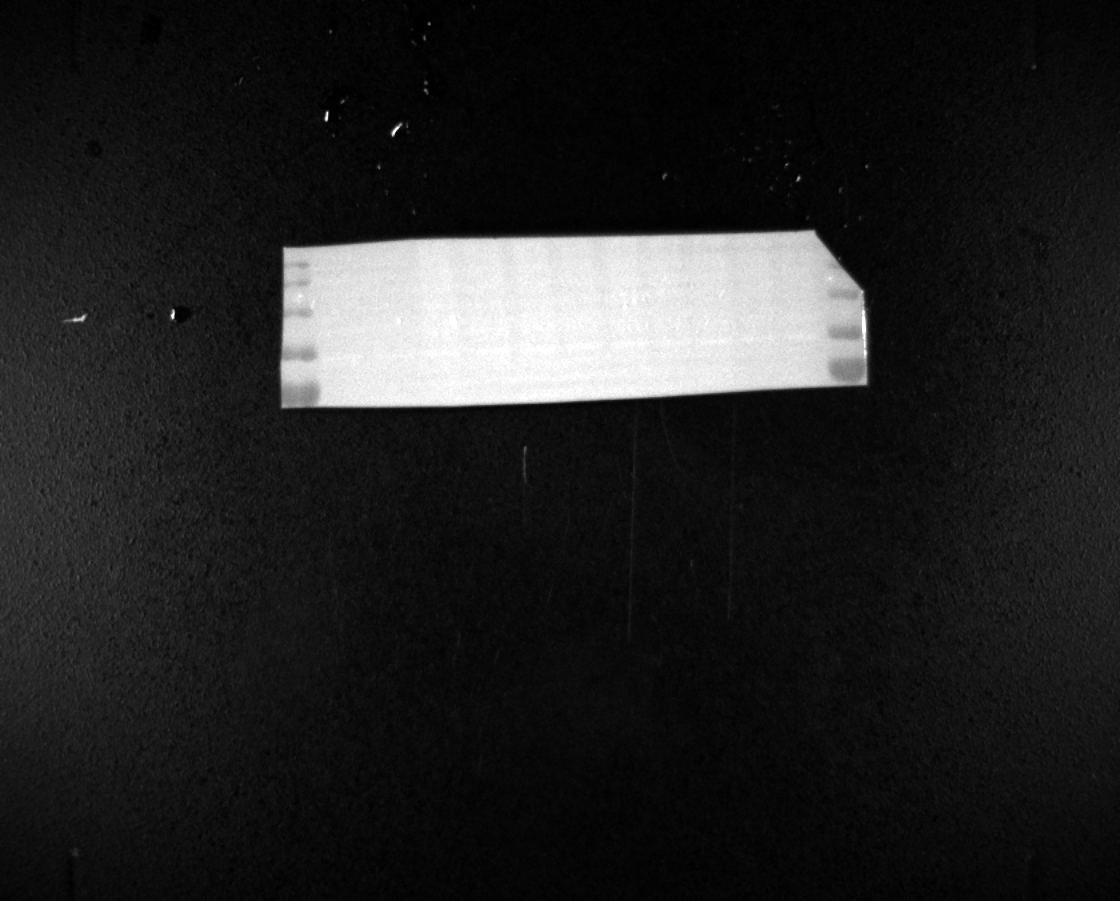

Supplement: Supplementary file 4 — Source Data [file 41467_2023_41520_MOESM4_ESM.zip › Source Data/Uncropped and Unprocessed Scans/Fig. S2b/Right/IB SUMO1 - Marker.tif]

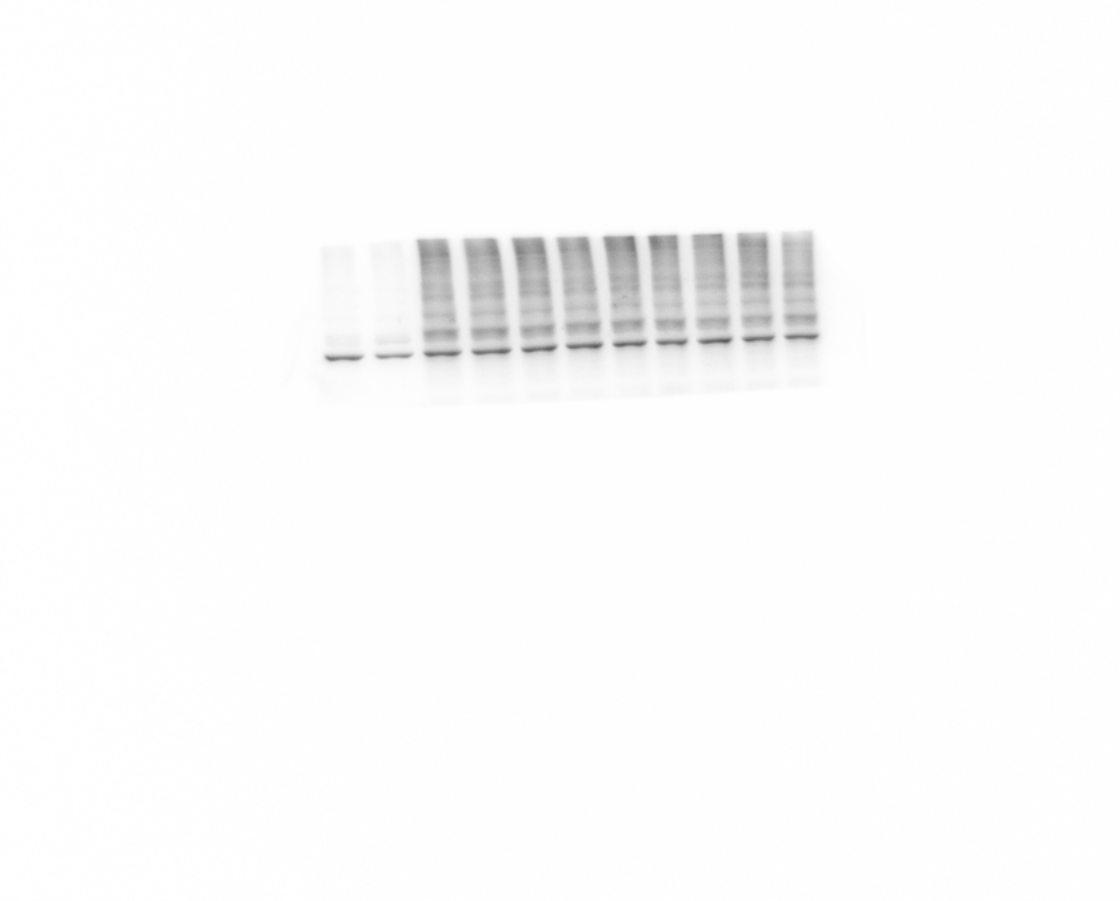

Supplement: Supplementary file 4 — Source Data [file 41467_2023_41520_MOESM4_ESM.zip › Source Data/Uncropped and Unprocessed Scans/Fig. S2b/Right/IB SUMO1.tif]

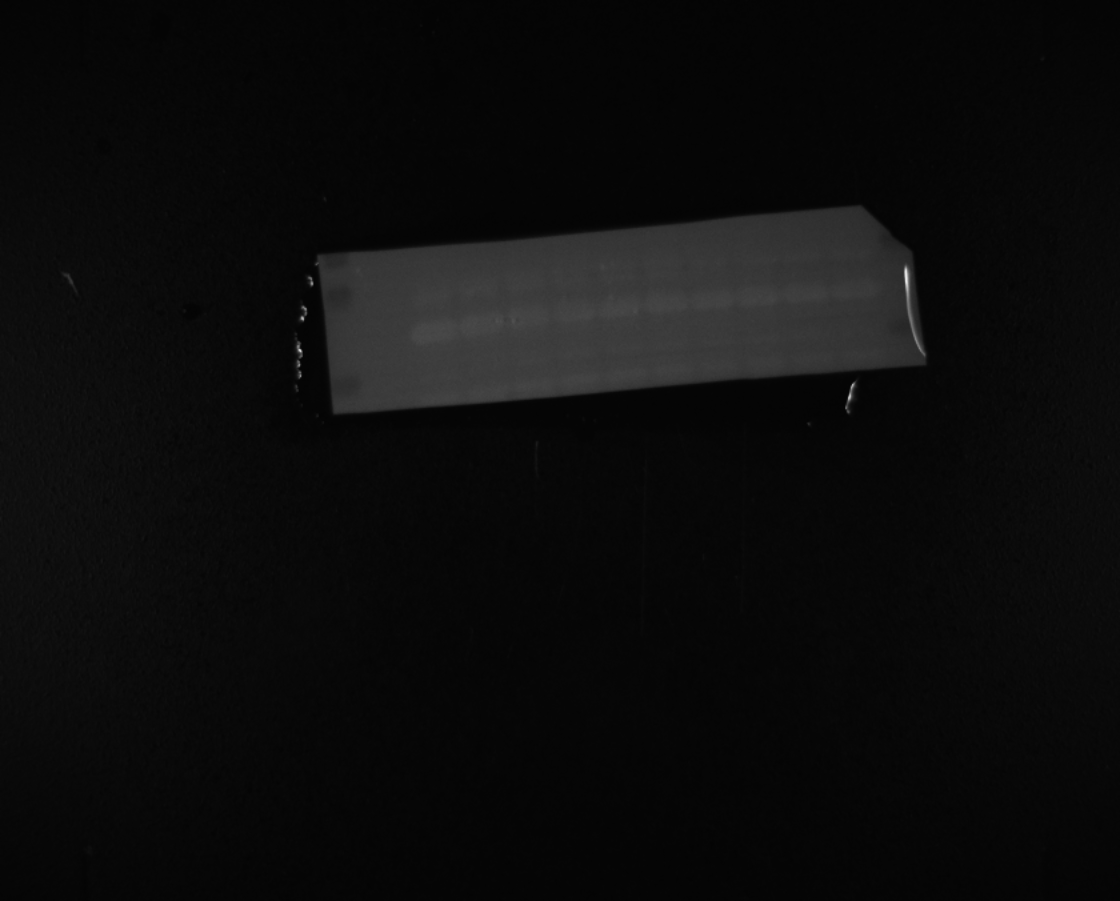

Supplement: Supplementary file 4 — Source Data [file 41467_2023_41520_MOESM4_ESM.zip › Source Data/Uncropped and Unprocessed Scans/Fig. S2b/Right/IP HA; IB HA - Marker.tif]

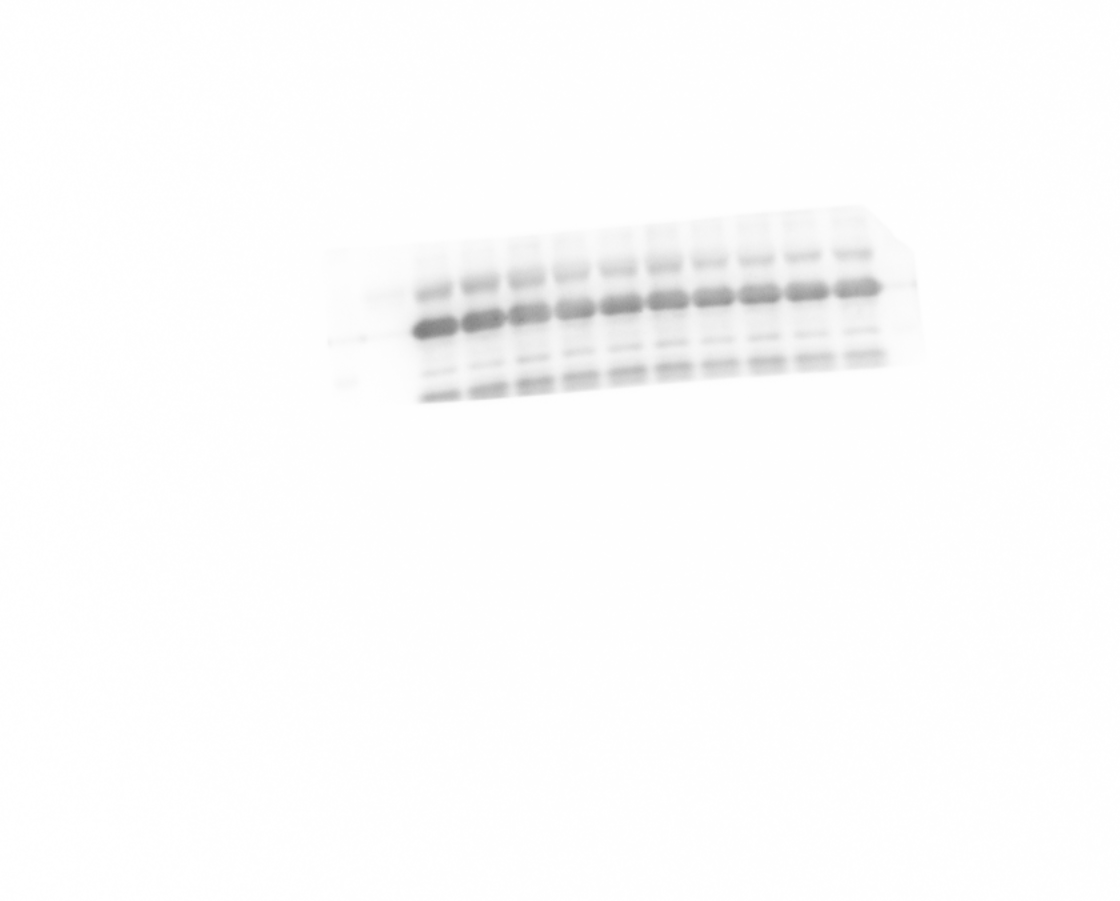

Supplement: Supplementary file 4 — Source Data [file 41467_2023_41520_MOESM4_ESM.zip › Source Data/Uncropped and Unprocessed Scans/Fig. S2b/Right/IP HA; IB HA.tif]

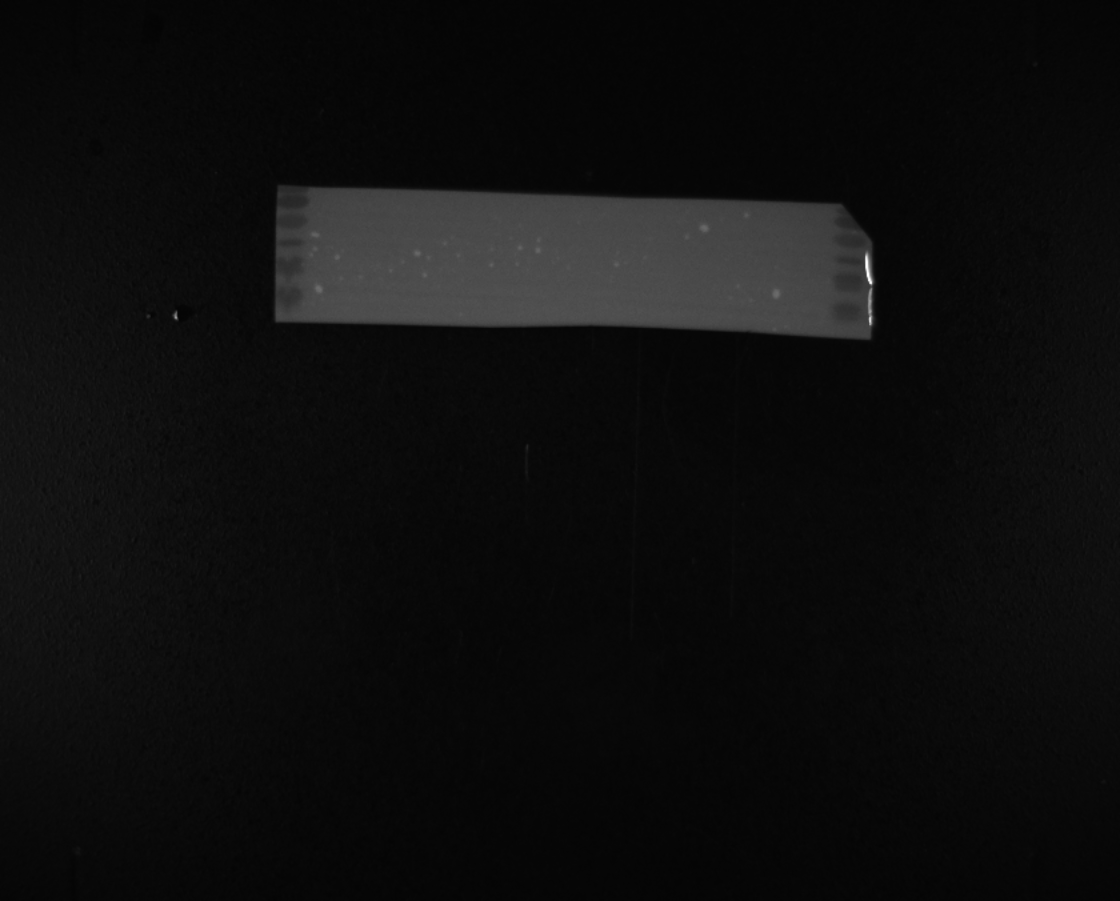

Supplement: Supplementary file 4 — Source Data [file 41467_2023_41520_MOESM4_ESM.zip › Source Data/Uncropped and Unprocessed Scans/Fig. S2b/Right/IP HA; IB SUMO1 - Marker.tif]

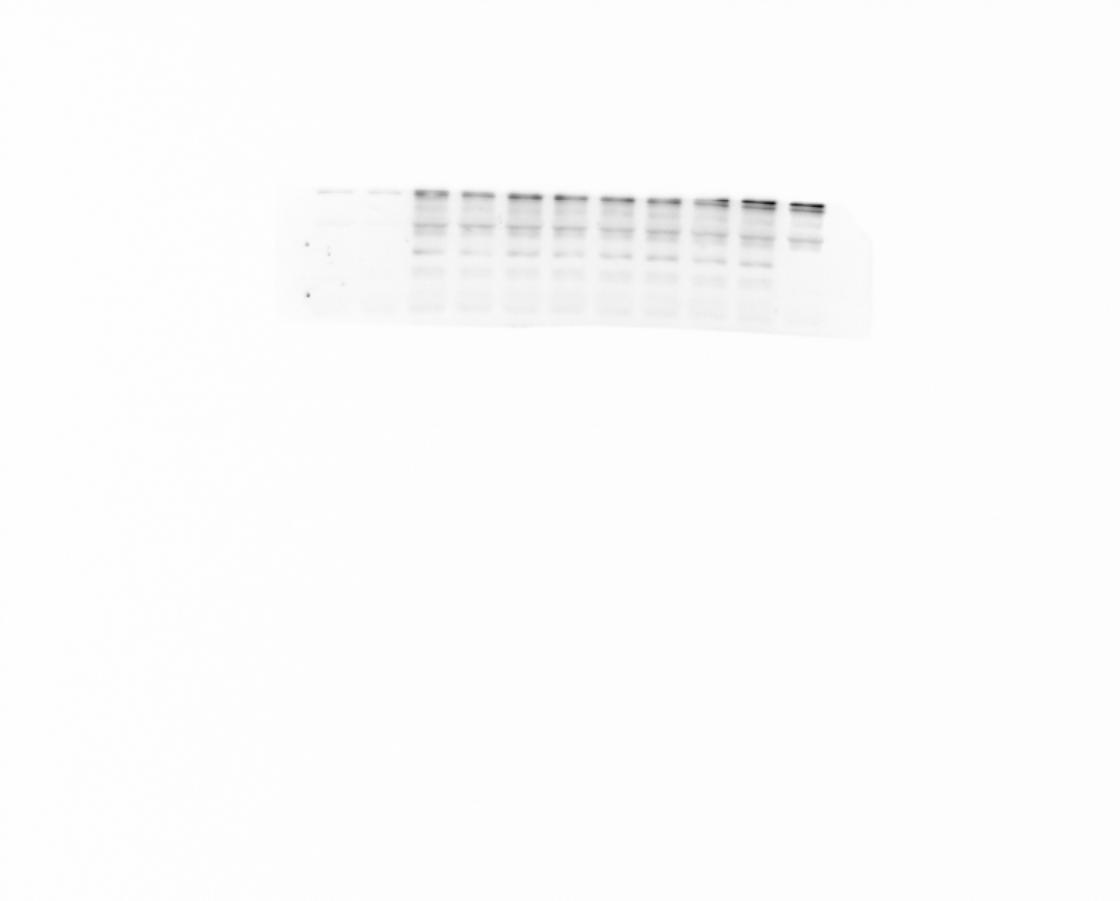

Supplement: Supplementary file 4 — Source Data [file 41467_2023_41520_MOESM4_ESM.zip › Source Data/Uncropped and Unprocessed Scans/Fig. S2b/Right/IP HA; IB SUMO1.tif]

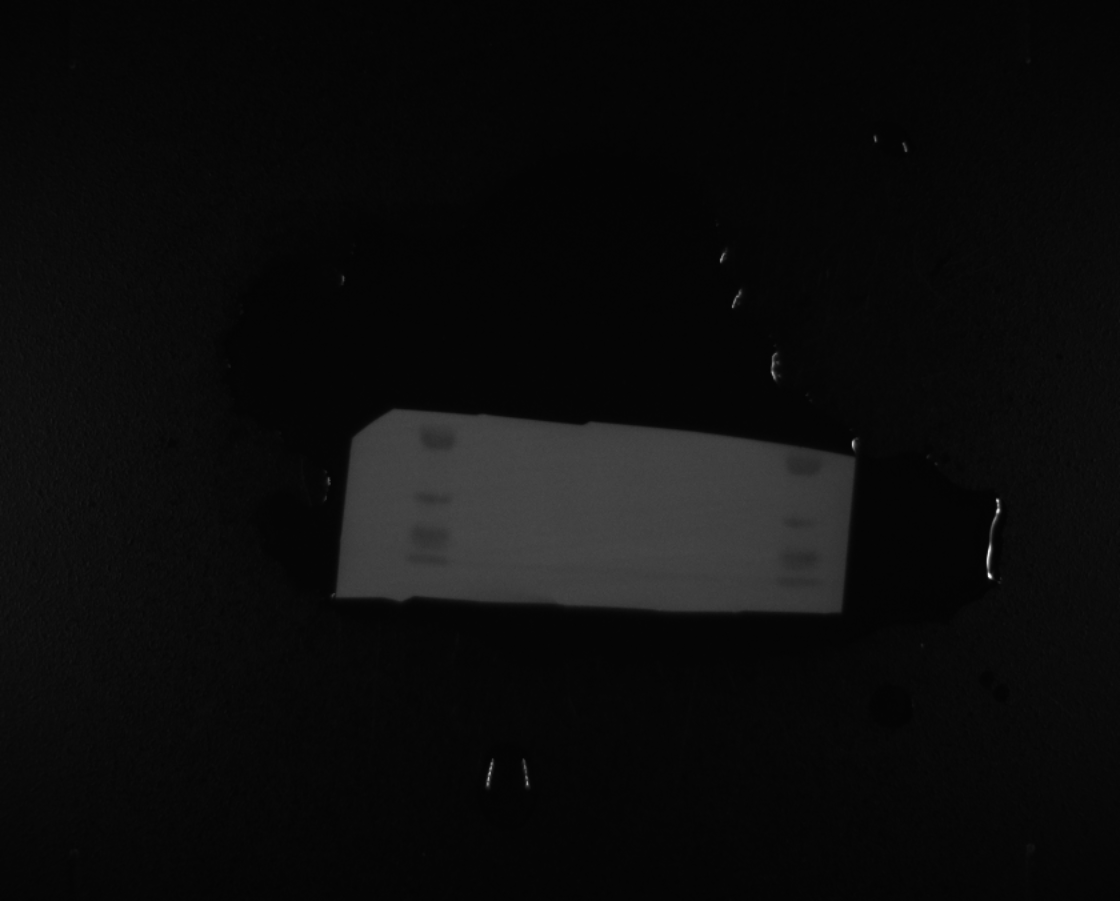

Supplement: Supplementary file 4 — Source Data [file 41467_2023_41520_MOESM4_ESM.zip › Source Data/Uncropped and Unprocessed Scans/Fig. S3a/IB GAPDH - Marker.tif]

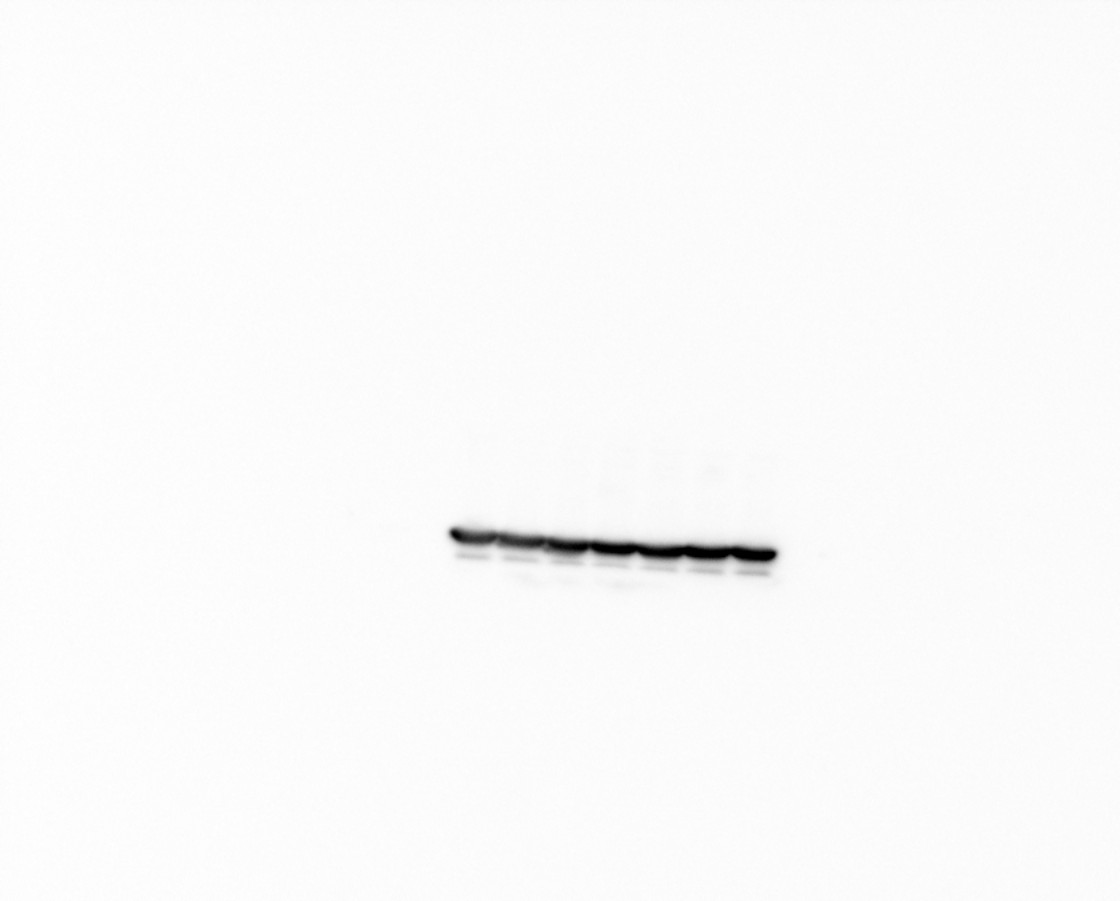

Supplement: Supplementary file 4 — Source Data [file 41467_2023_41520_MOESM4_ESM.zip › Source Data/Uncropped and Unprocessed Scans/Fig. S3a/IB GAPDH.tif]

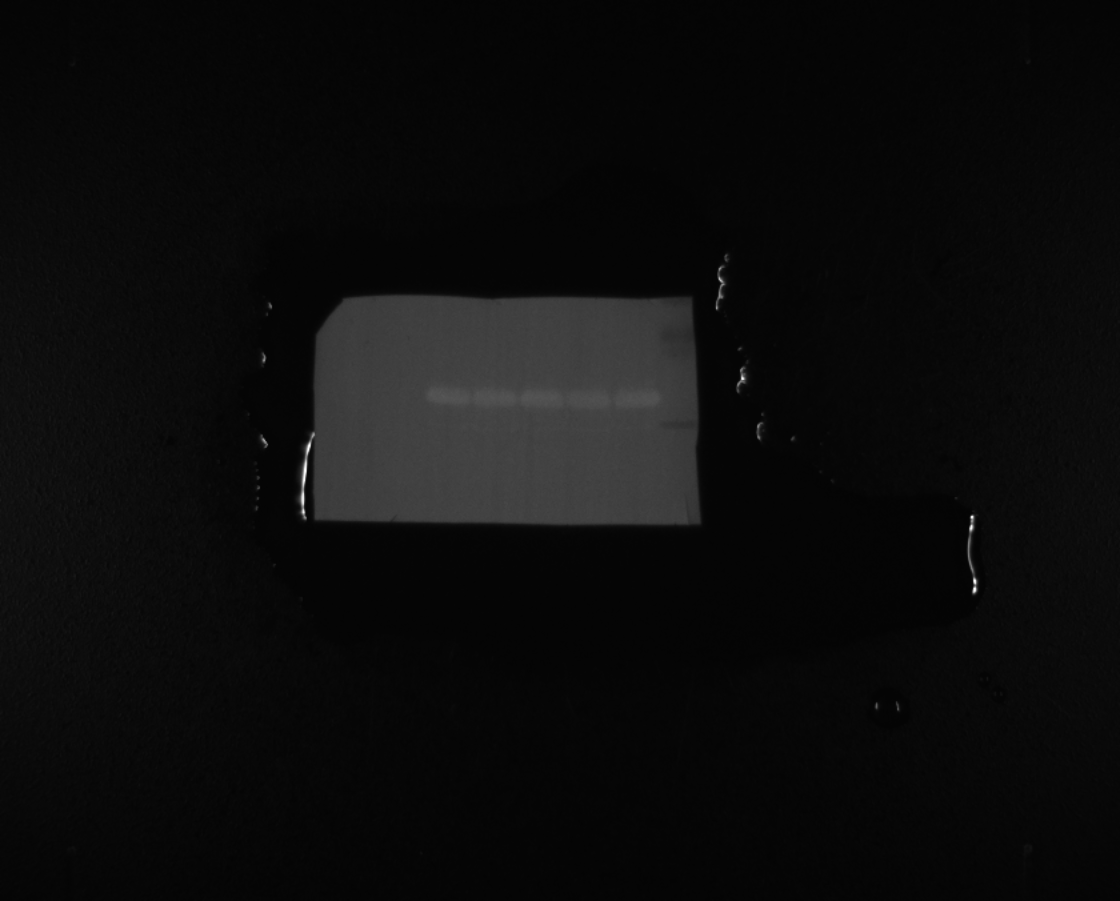

Supplement: Supplementary file 4 — Source Data [file 41467_2023_41520_MOESM4_ESM.zip › Source Data/Uncropped and Unprocessed Scans/Fig. S3a/IB HA - Marker.tif]

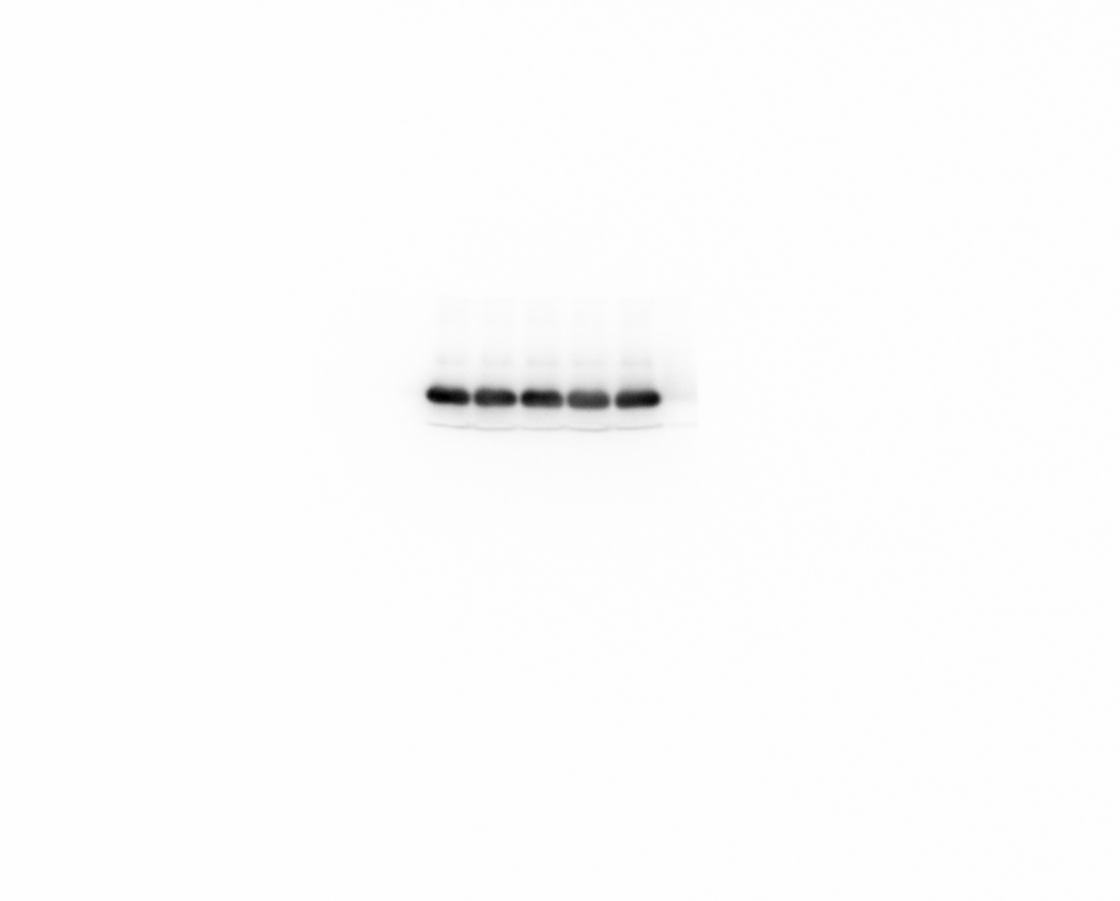

Supplement: Supplementary file 4 — Source Data [file 41467_2023_41520_MOESM4_ESM.zip › Source Data/Uncropped and Unprocessed Scans/Fig. S3a/IB HA.tif]

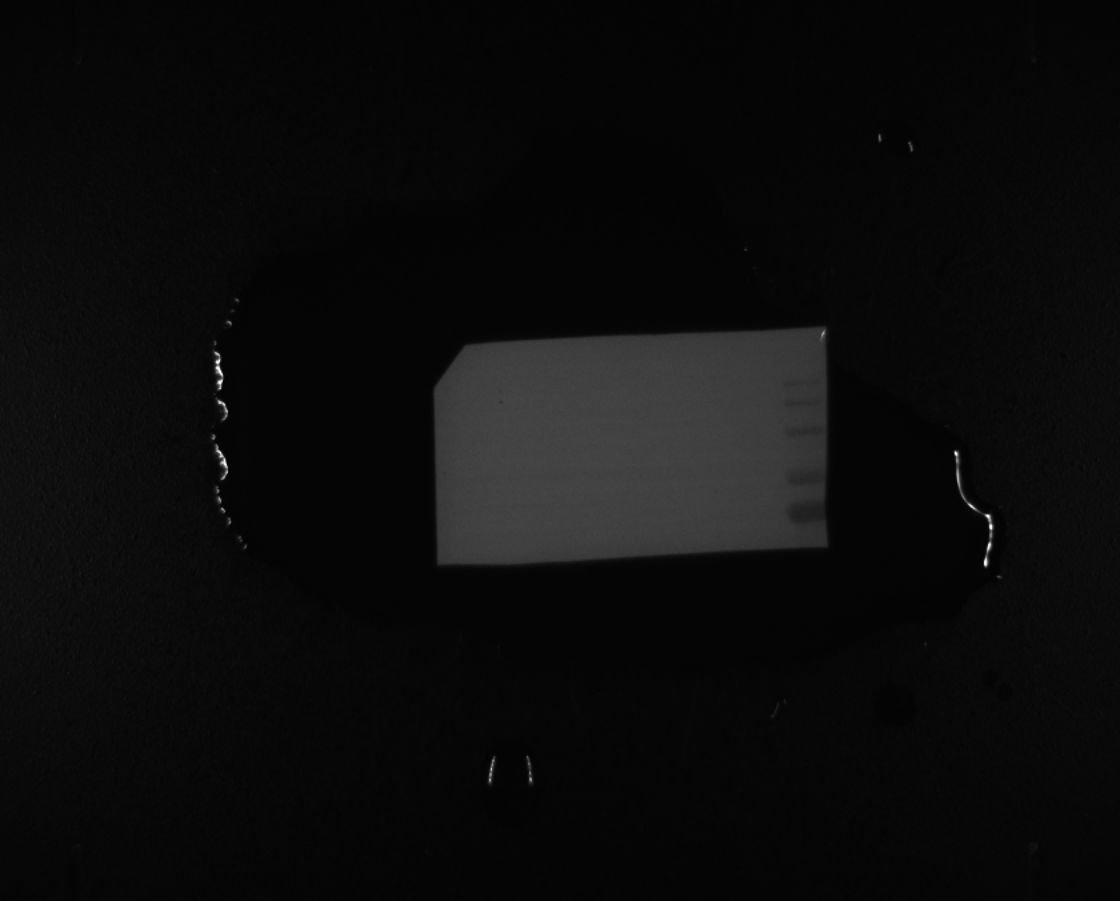

Supplement: Supplementary file 4 — Source Data [file 41467_2023_41520_MOESM4_ESM.zip › Source Data/Uncropped and Unprocessed Scans/Fig. S3a/IB SUMO1 - Marker.tif]

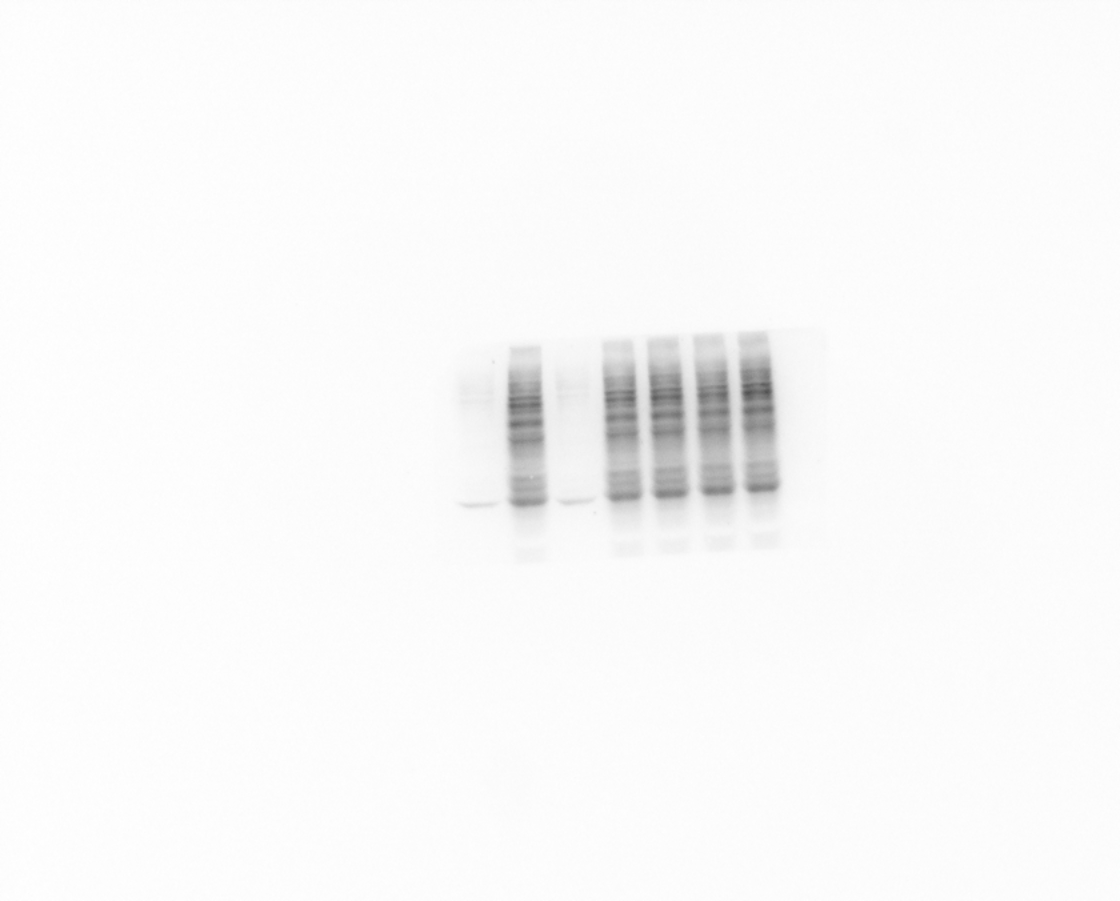

Supplement: Supplementary file 4 — Source Data [file 41467_2023_41520_MOESM4_ESM.zip › Source Data/Uncropped and Unprocessed Scans/Fig. S3a/IB SUMO1.tif]

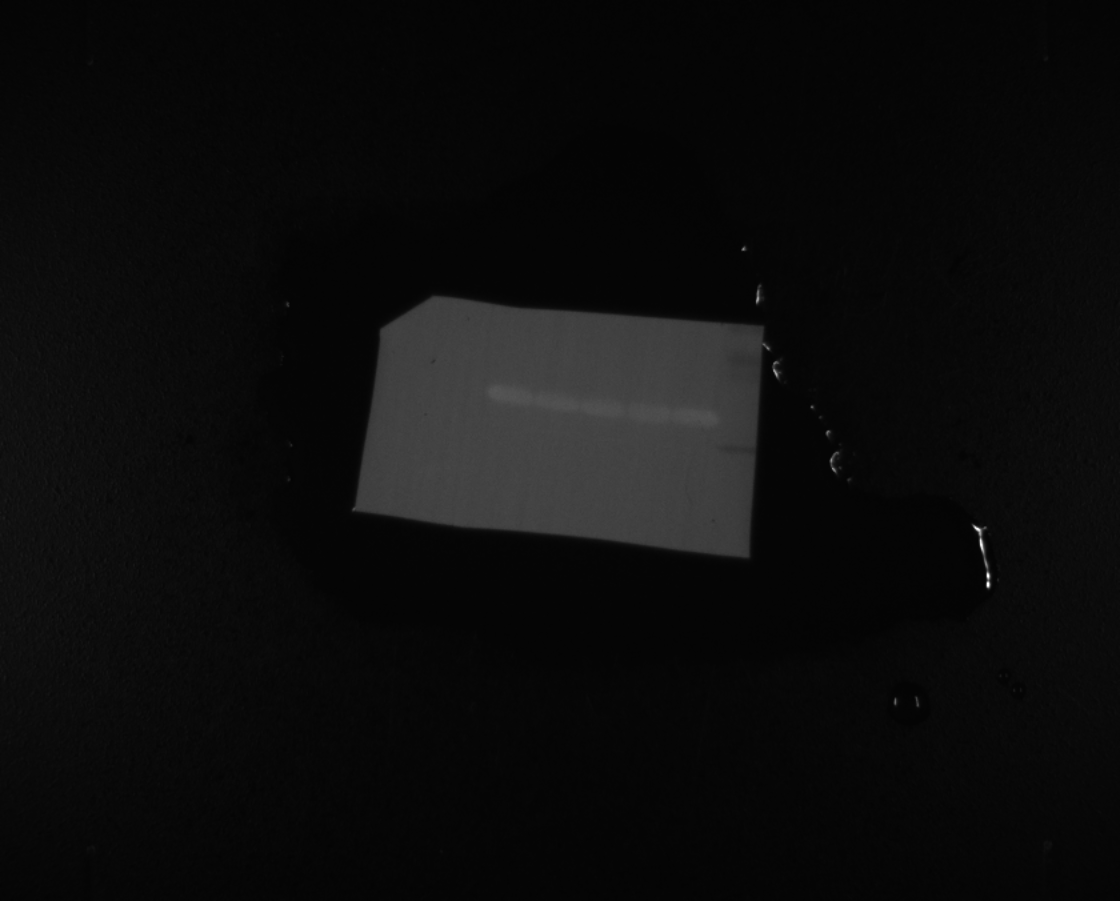

Supplement: Supplementary file 4 — Source Data [file 41467_2023_41520_MOESM4_ESM.zip › Source Data/Uncropped and Unprocessed Scans/Fig. S3a/IP HA; IB HA - Marker.tif]

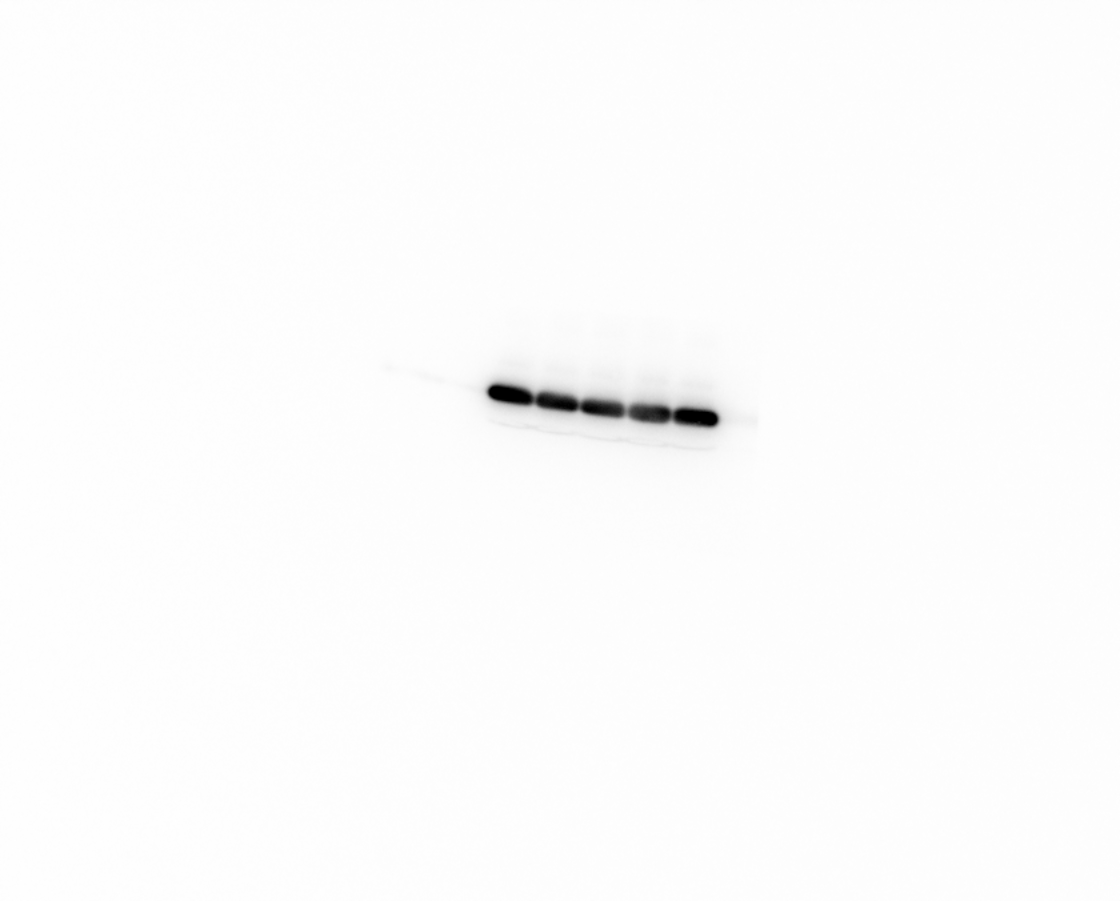

Supplement: Supplementary file 4 — Source Data [file 41467_2023_41520_MOESM4_ESM.zip › Source Data/Uncropped and Unprocessed Scans/Fig. S3a/IP HA; IB HA.tif]

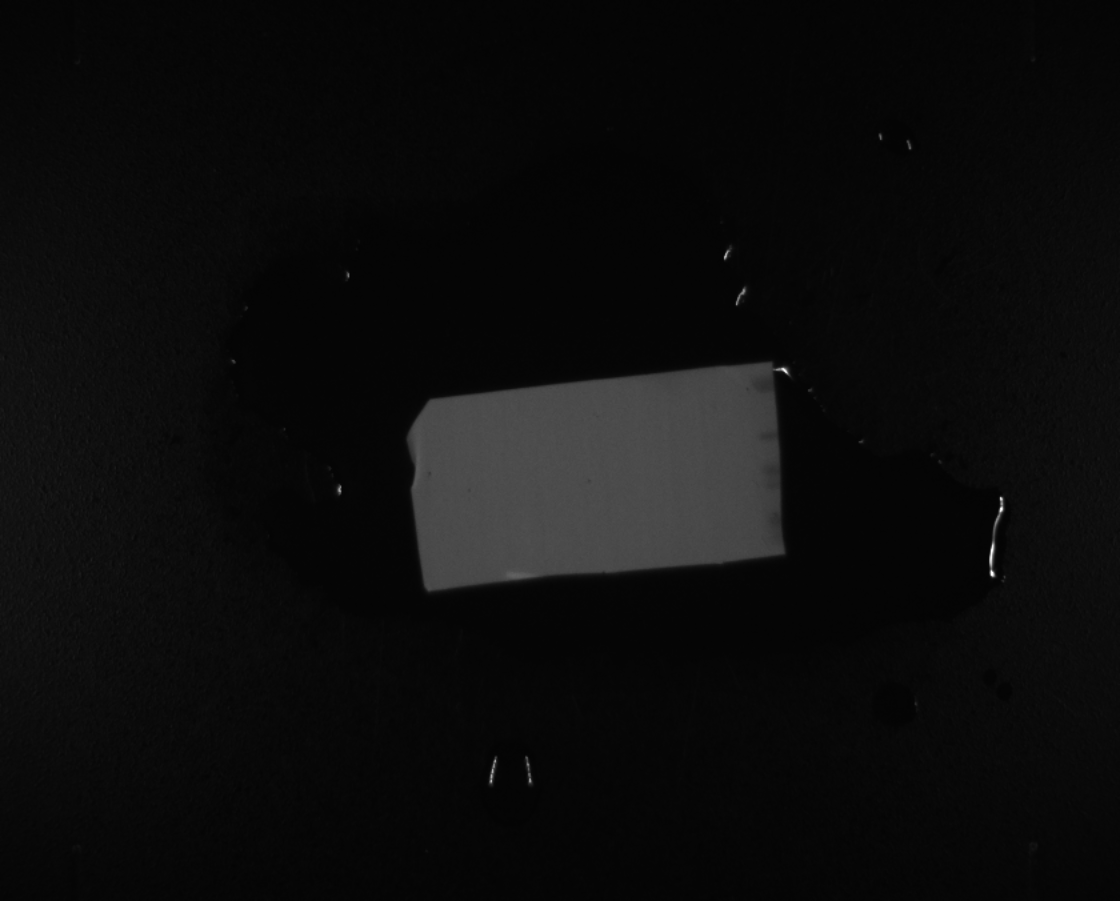

Supplement: Supplementary file 4 — Source Data [file 41467_2023_41520_MOESM4_ESM.zip › Source Data/Uncropped and Unprocessed Scans/Fig. S3a/IP HA; IB SUMO1 - Marker.tif]

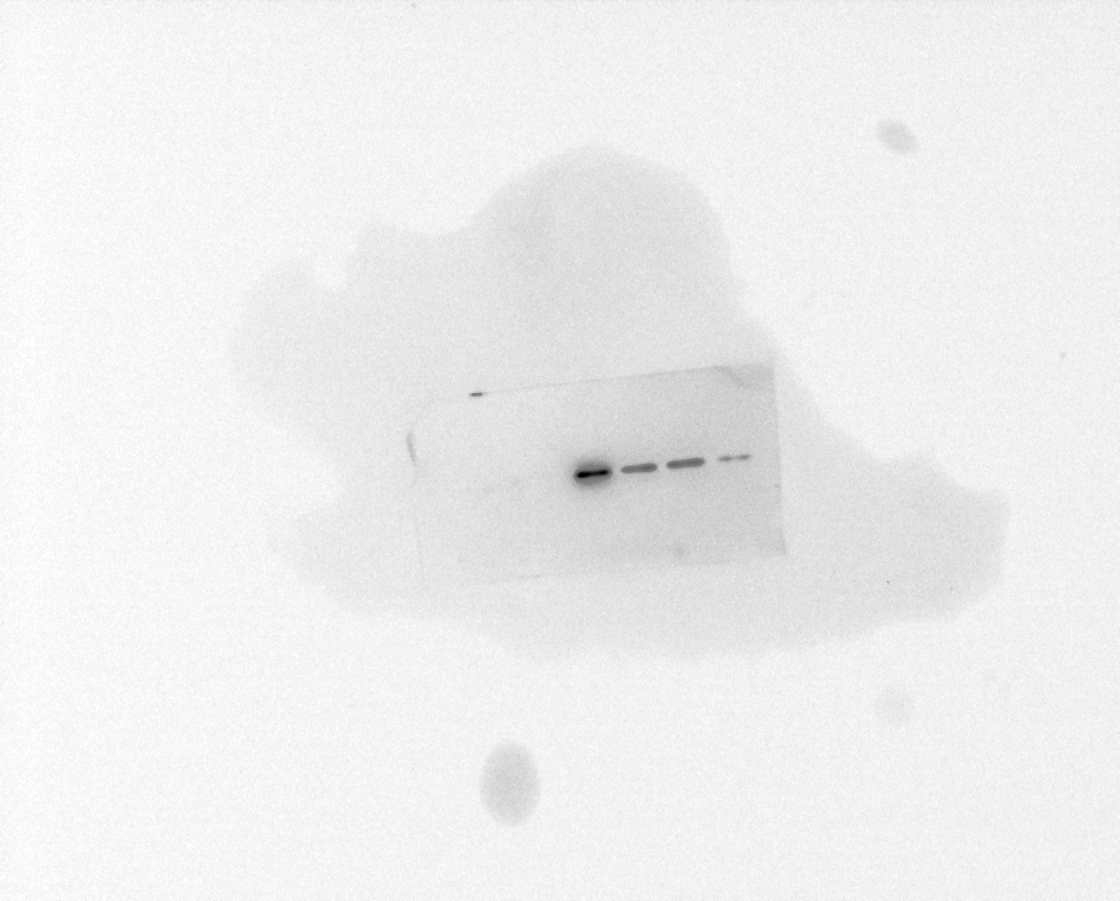

Supplement: Supplementary file 4 — Source Data [file 41467_2023_41520_MOESM4_ESM.zip › Source Data/Uncropped and Unprocessed Scans/Fig. S3a/IP HA; IB SUMO1.tif]

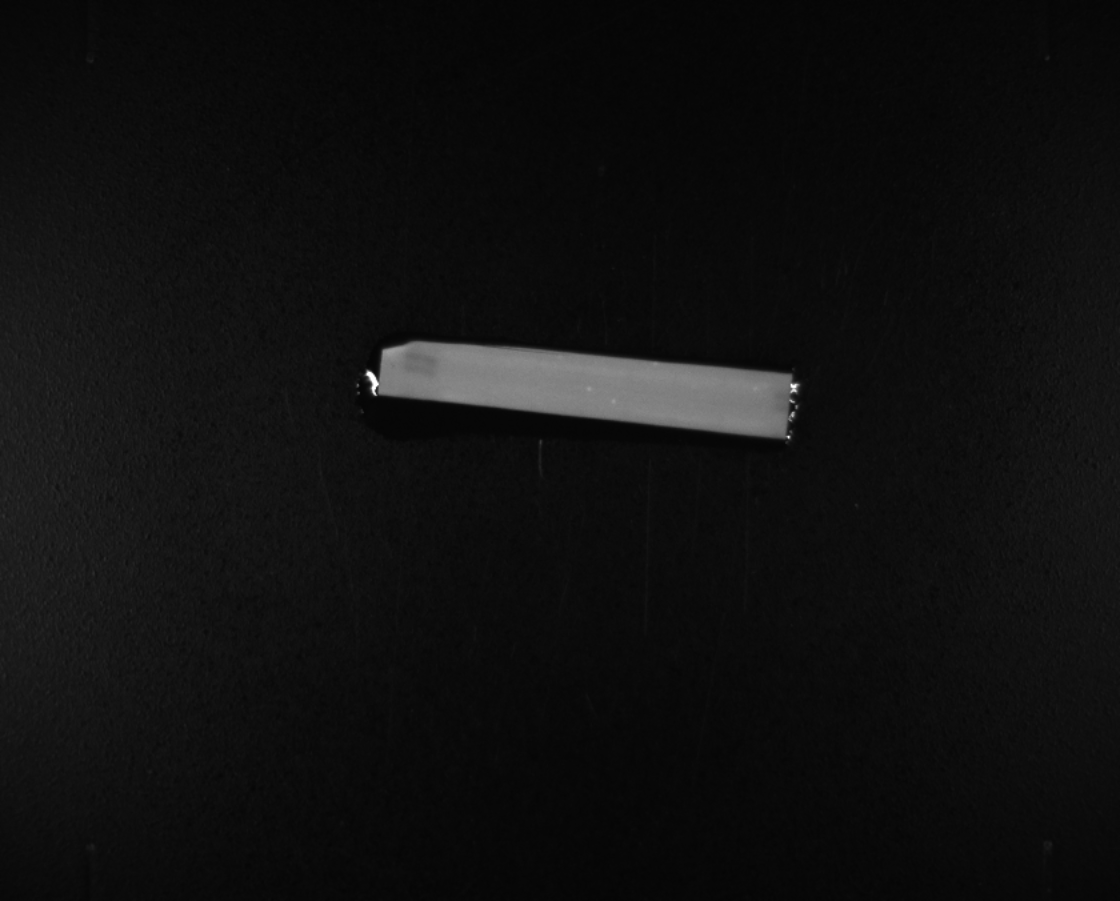

Supplement: Supplementary file 4 — Source Data [file 41467_2023_41520_MOESM4_ESM.zip › Source Data/Uncropped and Unprocessed Scans/Fig. S3b/IB GAPDH - Marker.tif]
