# Supplementary figures and images for: N-terminal α-amino SUMOylation of cofilin-1 is critical for its regulation of actin depolymerization (part 1 of 2)
Source: Nat Commun. 2023 Sep 14;14:5688. doi: 10.1038/s41467-023-41520-2 (PMC10502023; doi:10.1038/s41467-023-41520-2)

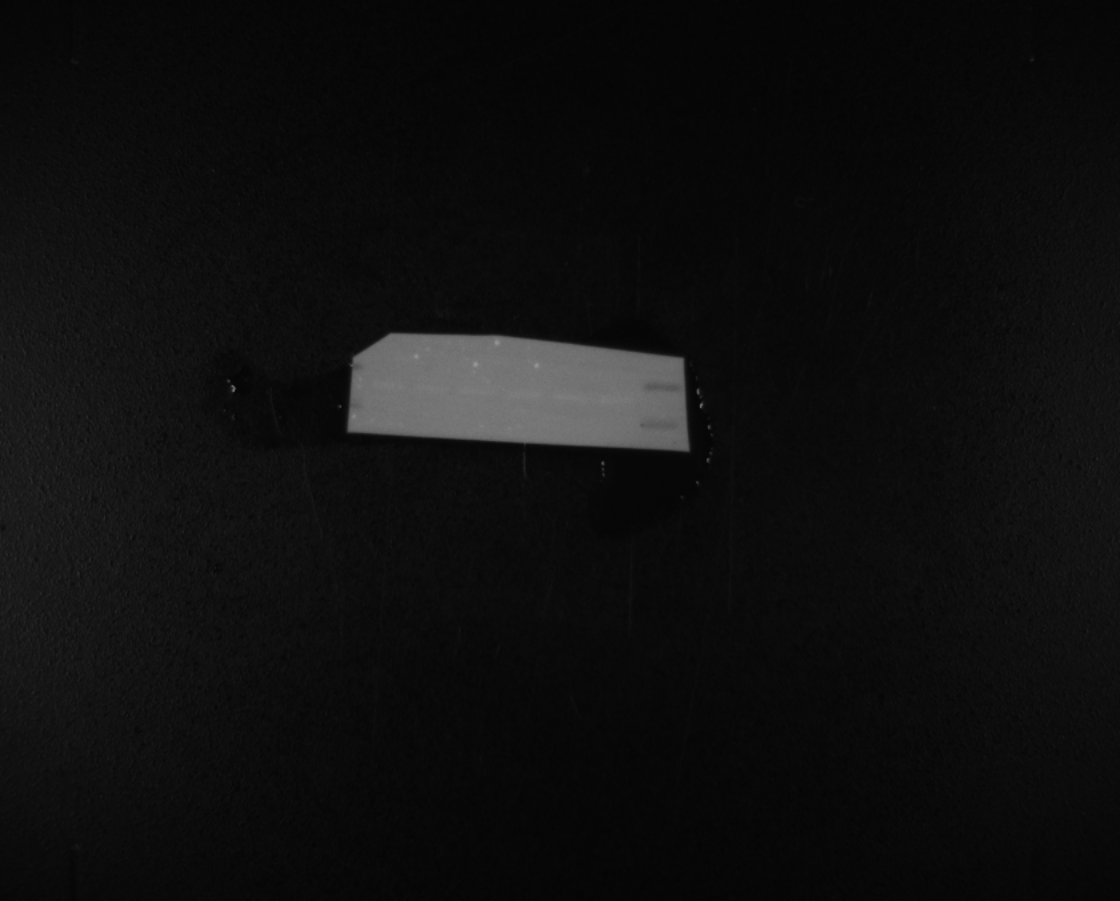

Supplement: Supplementary file 4 — Source Data [file 41467_2023_41520_MOESM4_ESM.zip › Source Data/Uncropped and Unprocessed Scans/Fig. 1a/IB GAPDH - Marker.tif]

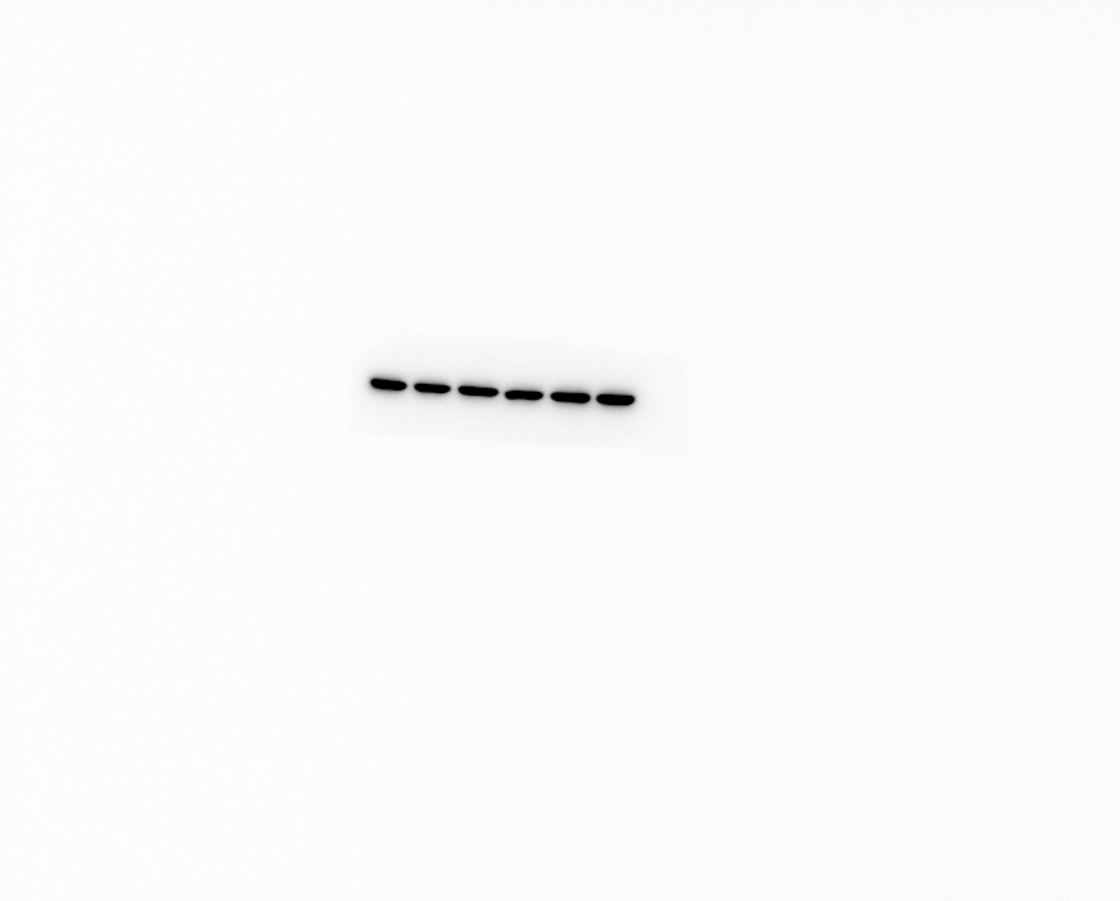

Supplement: Supplementary file 4 — Source Data [file 41467_2023_41520_MOESM4_ESM.zip › Source Data/Uncropped and Unprocessed Scans/Fig. 1a/IB GAPDH.tif]

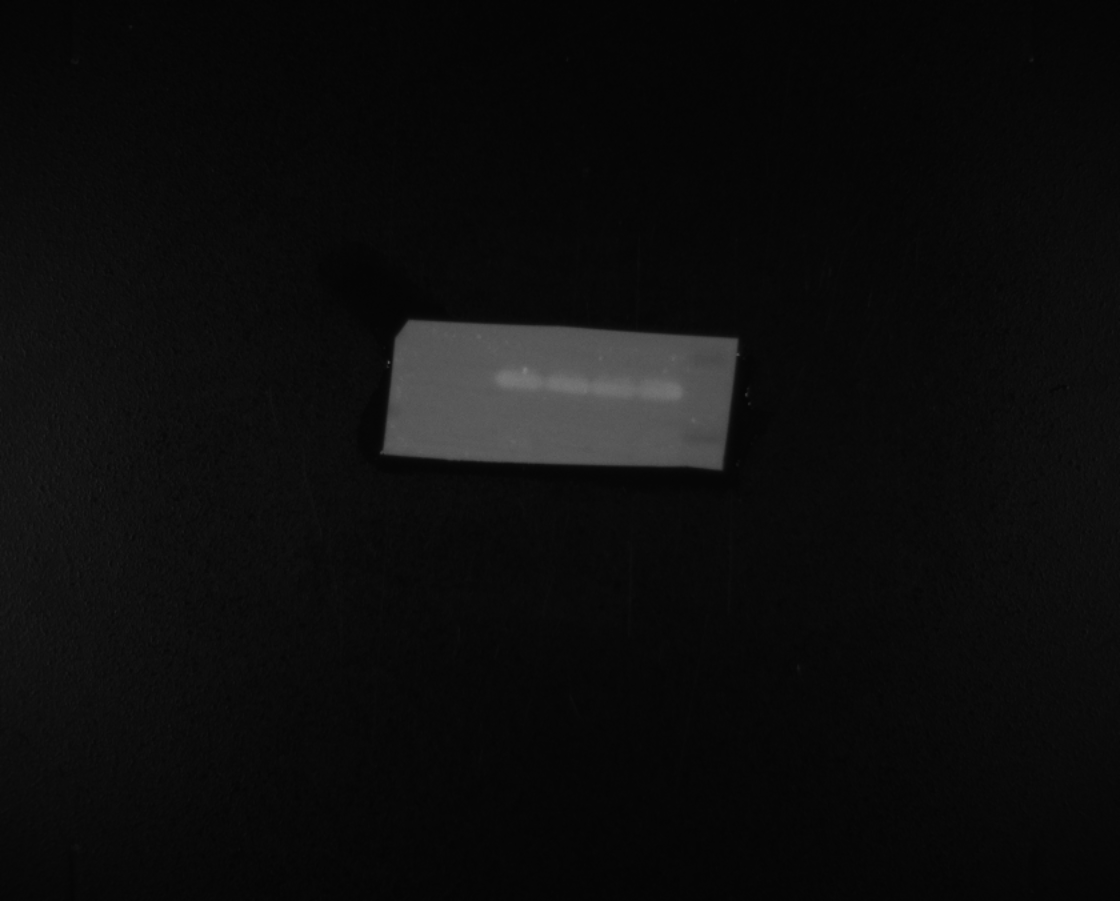

Supplement: Supplementary file 4 — Source Data [file 41467_2023_41520_MOESM4_ESM.zip › Source Data/Uncropped and Unprocessed Scans/Fig. 1a/IB HA - Marker.tif]

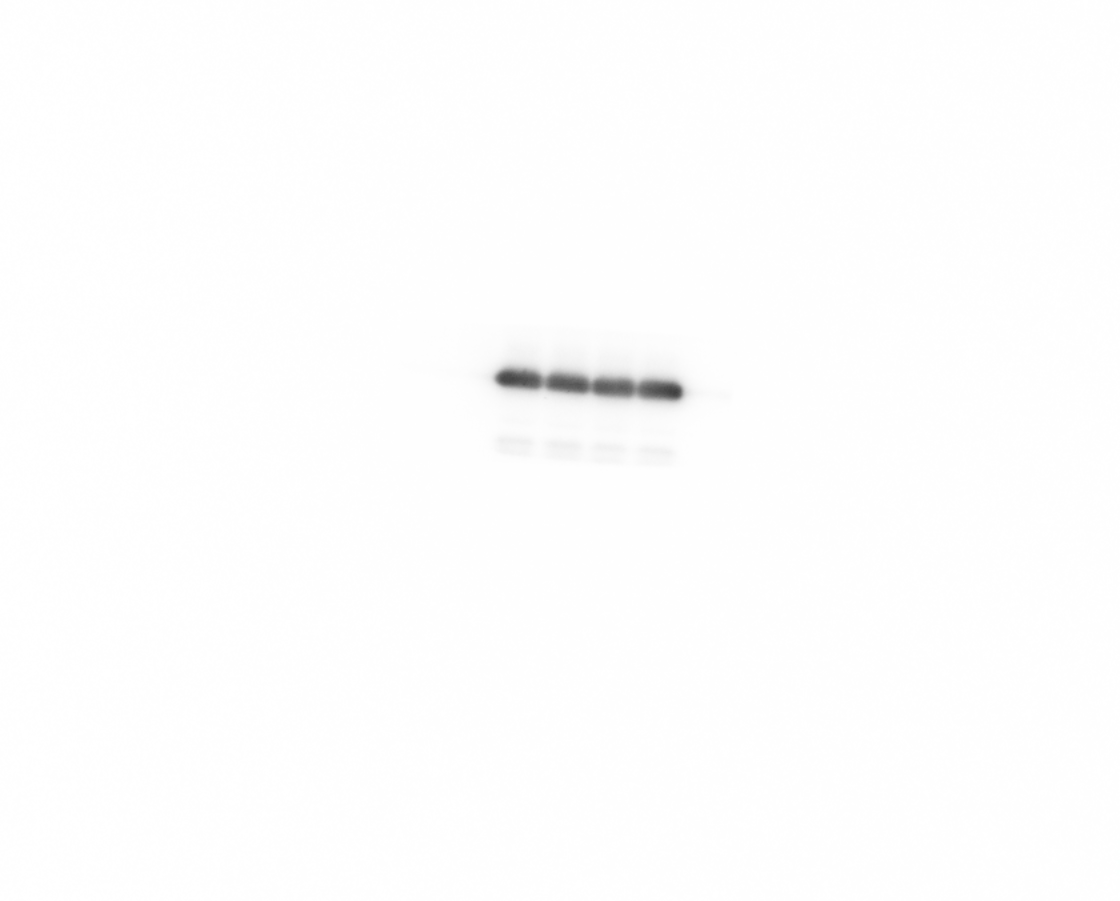

Supplement: Supplementary file 4 — Source Data [file 41467_2023_41520_MOESM4_ESM.zip › Source Data/Uncropped and Unprocessed Scans/Fig. 1a/IB HA.tif]

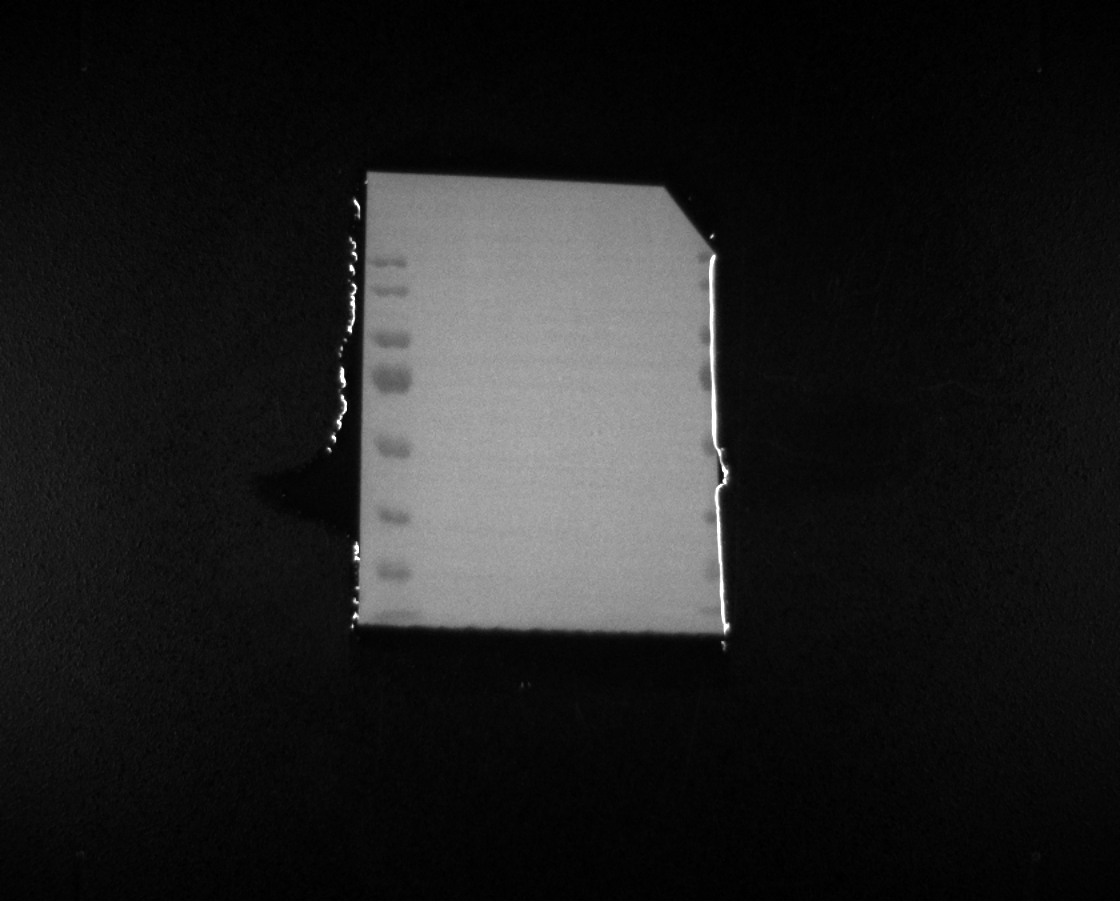

Supplement: Supplementary file 4 — Source Data [file 41467_2023_41520_MOESM4_ESM.zip › Source Data/Uncropped and Unprocessed Scans/Fig. 1a/IB SUMO1 - Marker.tif]

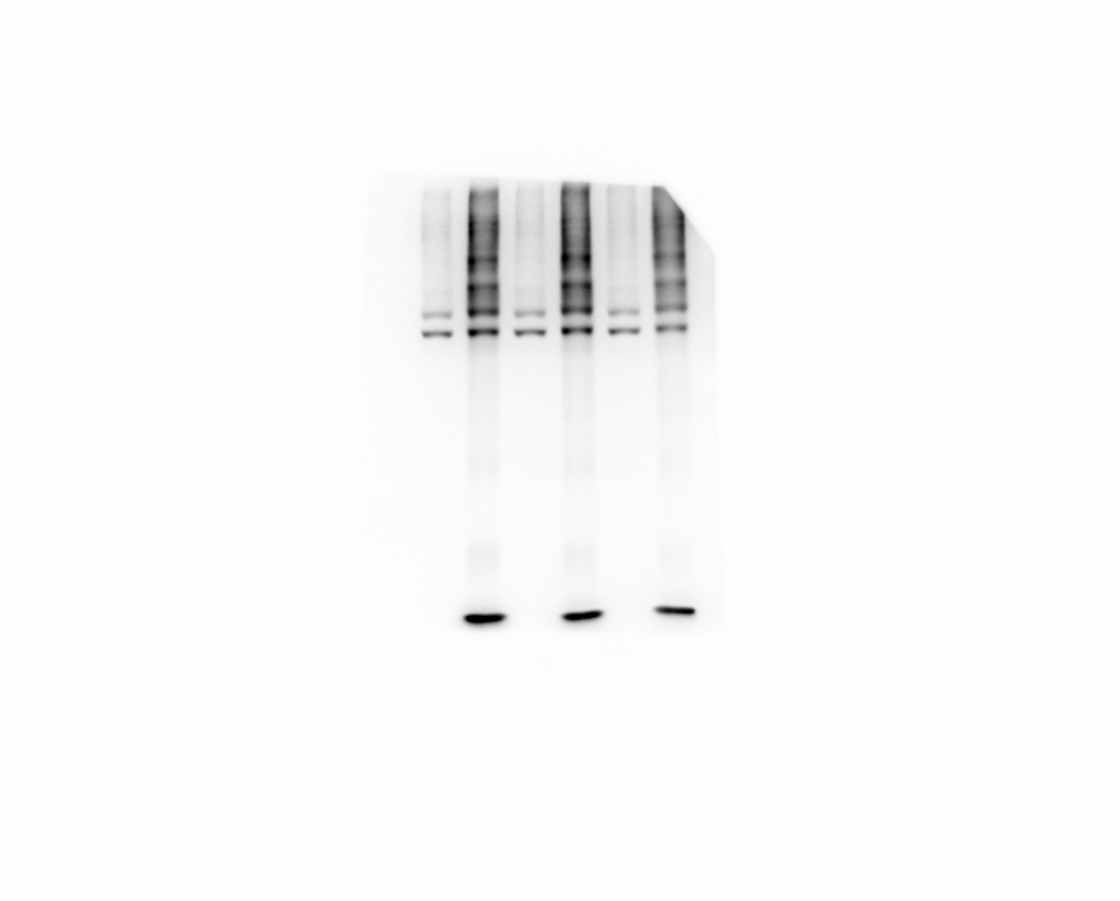

Supplement: Supplementary file 4 — Source Data [file 41467_2023_41520_MOESM4_ESM.zip › Source Data/Uncropped and Unprocessed Scans/Fig. 1a/IB SUMO1.tif]

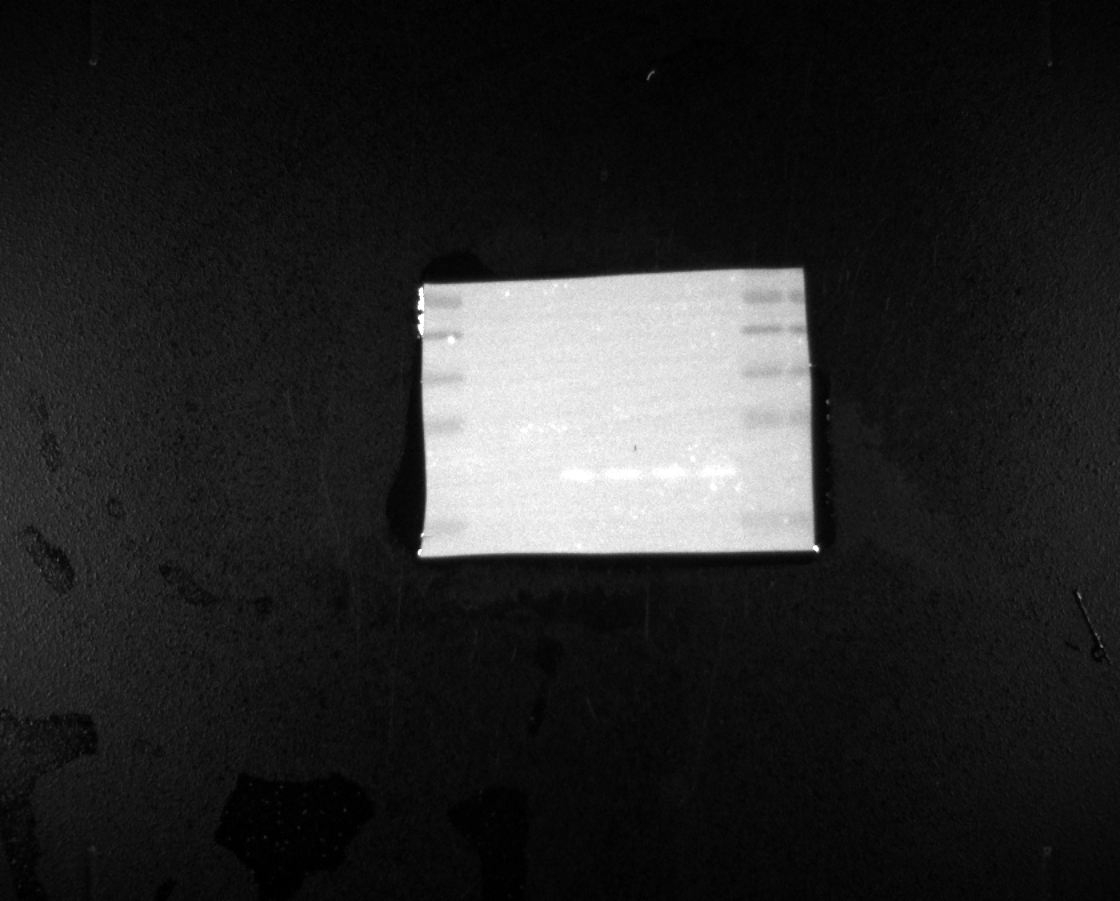

Supplement: Supplementary file 4 — Source Data [file 41467_2023_41520_MOESM4_ESM.zip › Source Data/Uncropped and Unprocessed Scans/Fig. 1a/IP HA; IB HA - Marker.tif]

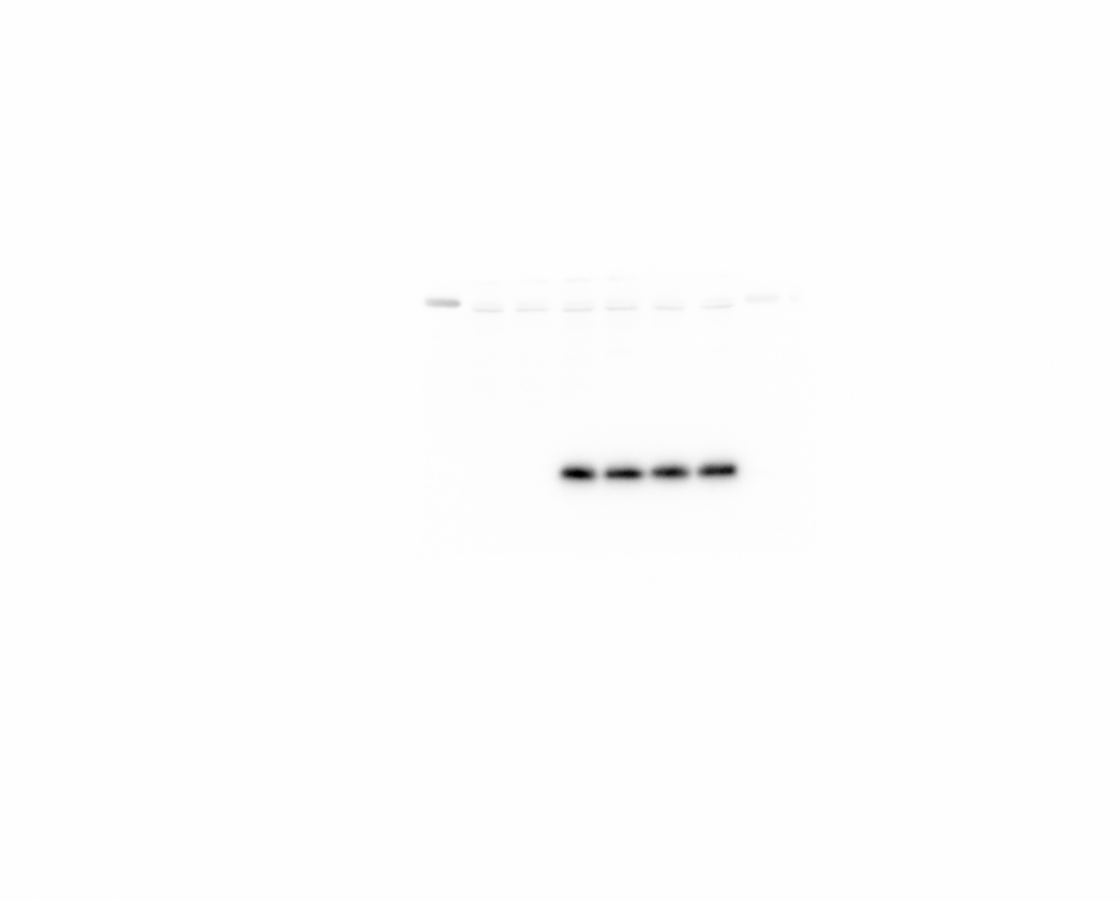

Supplement: Supplementary file 4 — Source Data [file 41467_2023_41520_MOESM4_ESM.zip › Source Data/Uncropped and Unprocessed Scans/Fig. 1a/IP HA; IB HA.tif]

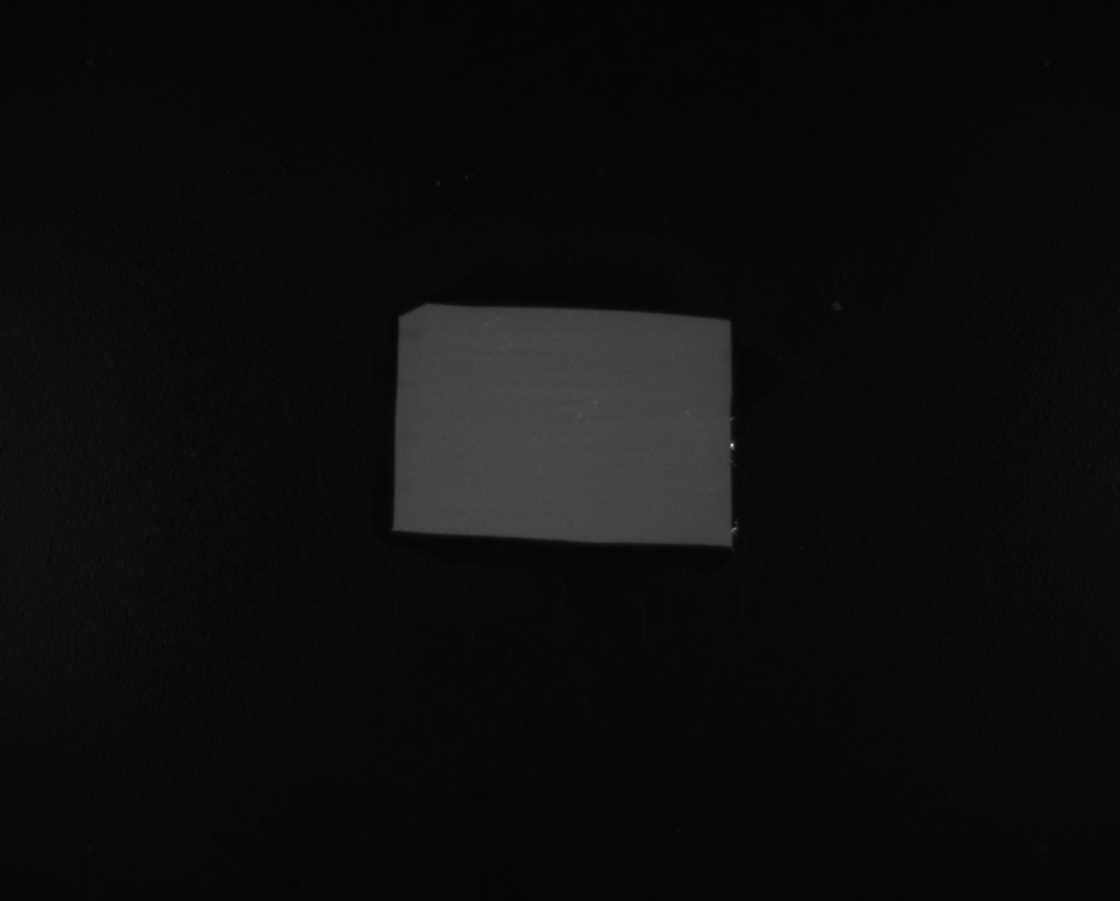

Supplement: Supplementary file 4 — Source Data [file 41467_2023_41520_MOESM4_ESM.zip › Source Data/Uncropped and Unprocessed Scans/Fig. 1a/IP HA; IB SUMO1 - Marker.tif]

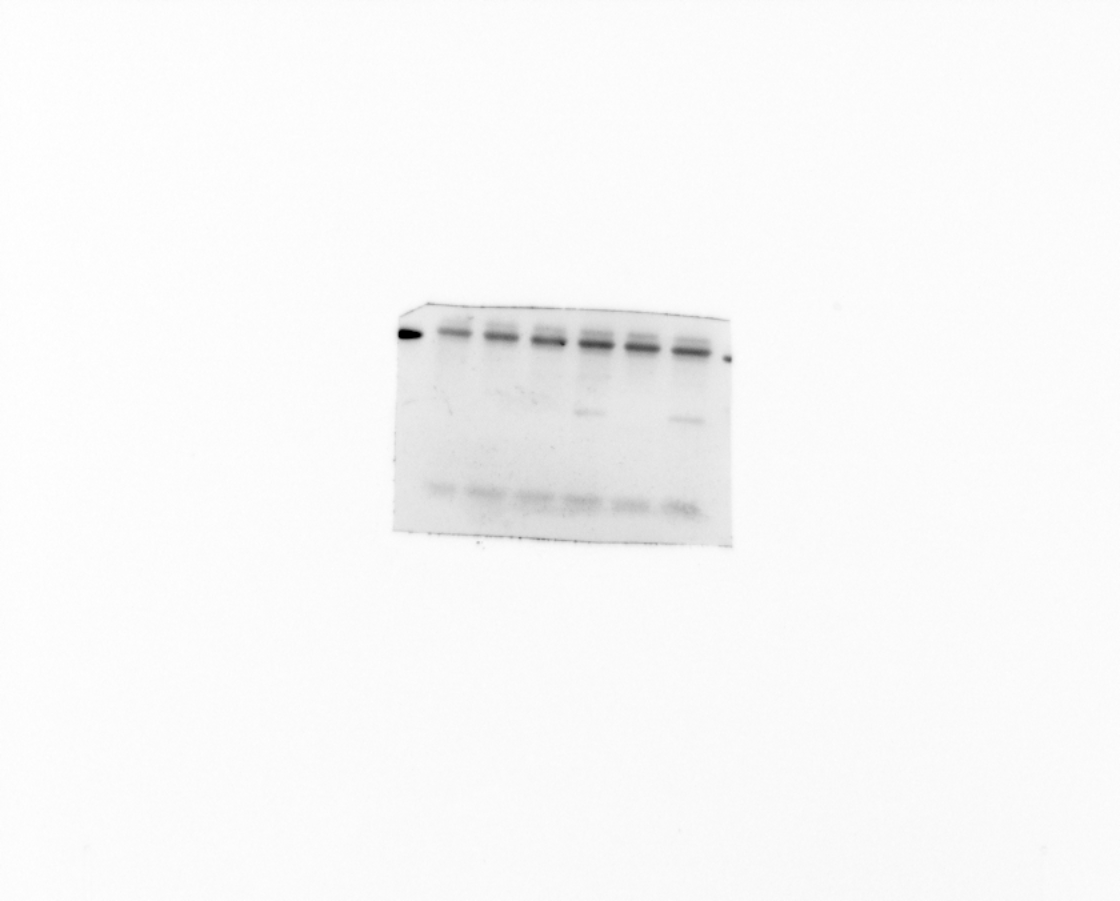

Supplement: Supplementary file 4 — Source Data [file 41467_2023_41520_MOESM4_ESM.zip › Source Data/Uncropped and Unprocessed Scans/Fig. 1a/IP HA; IB SUMO1.tif]

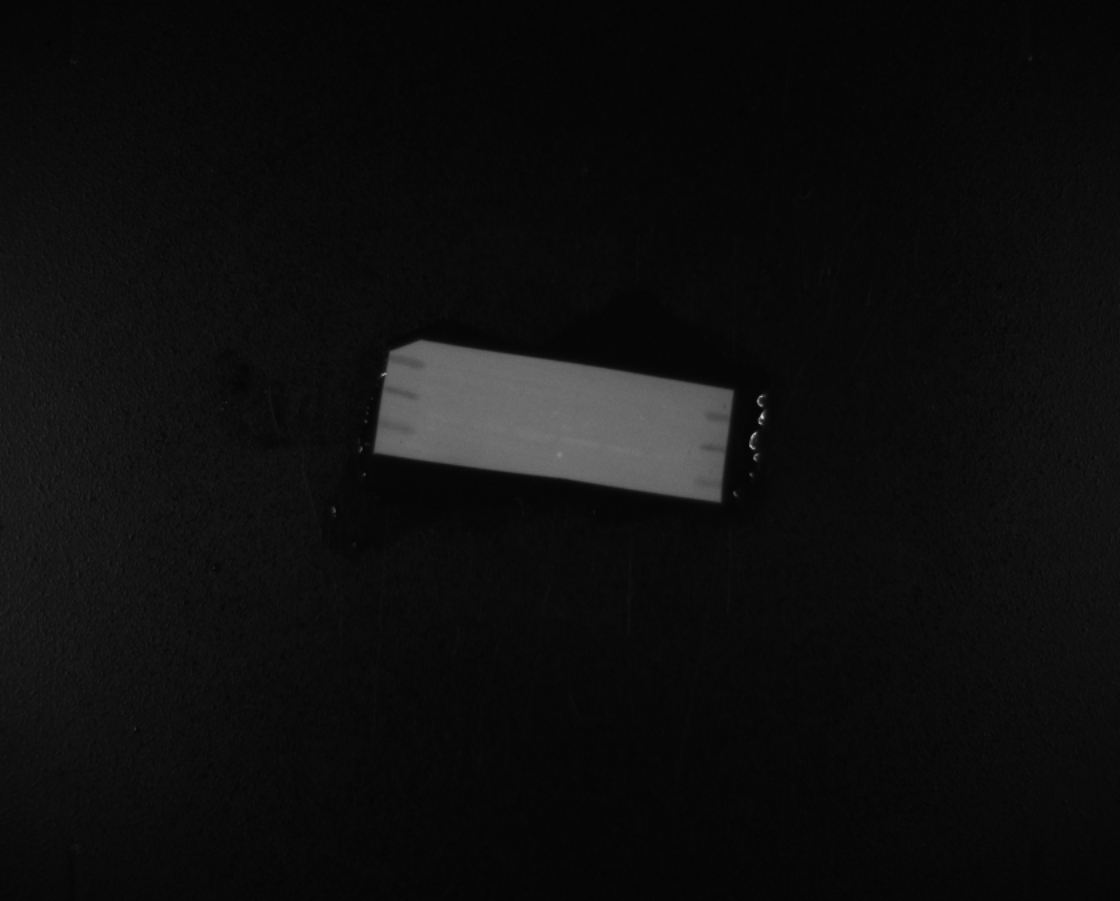

Supplement: Supplementary file 4 — Source Data [file 41467_2023_41520_MOESM4_ESM.zip › Source Data/Uncropped and Unprocessed Scans/Fig. 1b/IB GAPDH - Marker.tif]

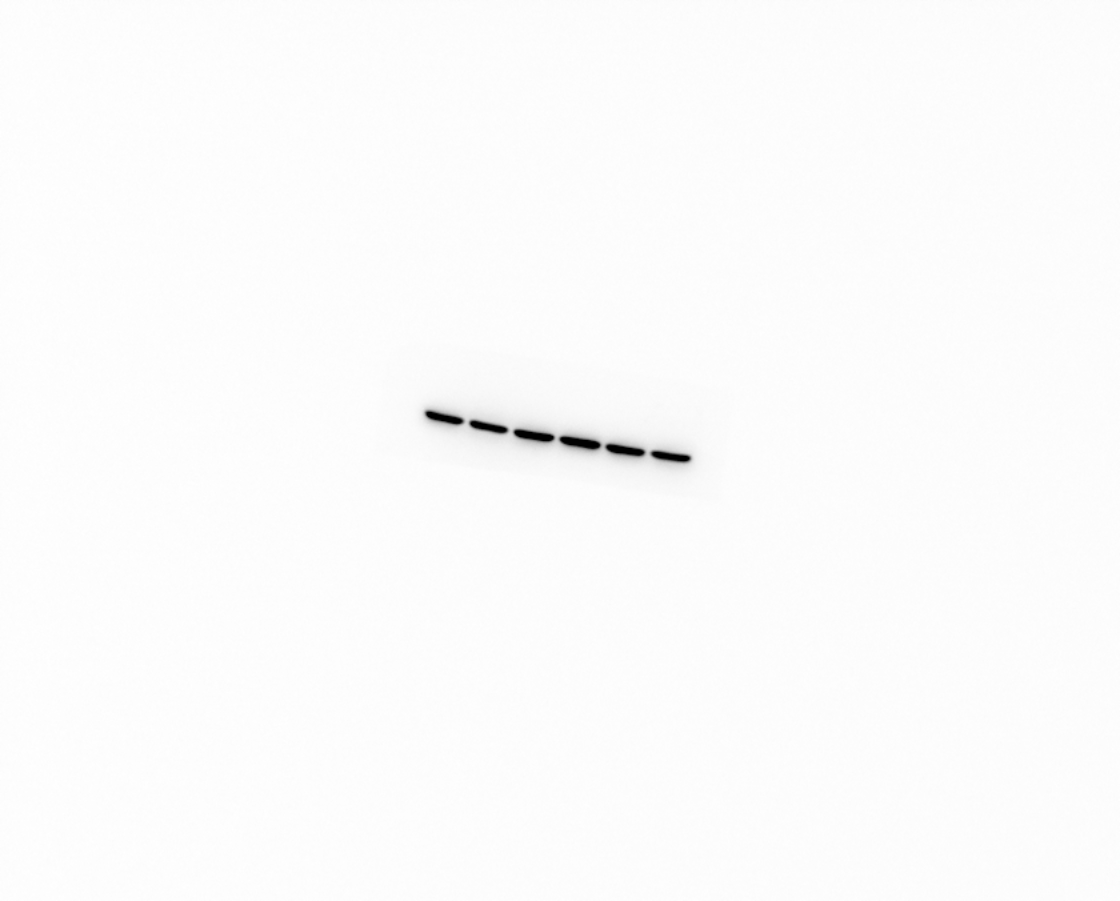

Supplement: Supplementary file 4 — Source Data [file 41467_2023_41520_MOESM4_ESM.zip › Source Data/Uncropped and Unprocessed Scans/Fig. 1b/IB GAPDH.tif]

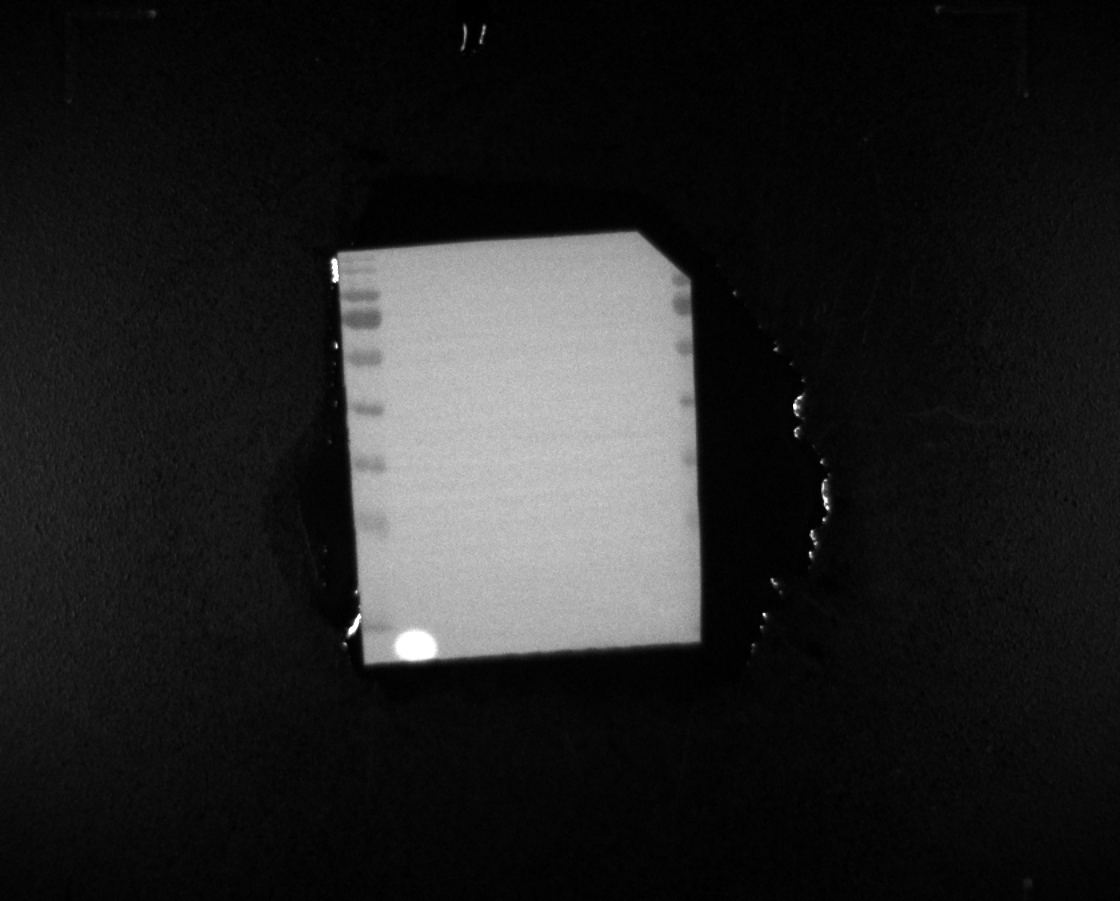

Supplement: Supplementary file 4 — Source Data [file 41467_2023_41520_MOESM4_ESM.zip › Source Data/Uncropped and Unprocessed Scans/Fig. 1b/IB HA - Marker.tif]

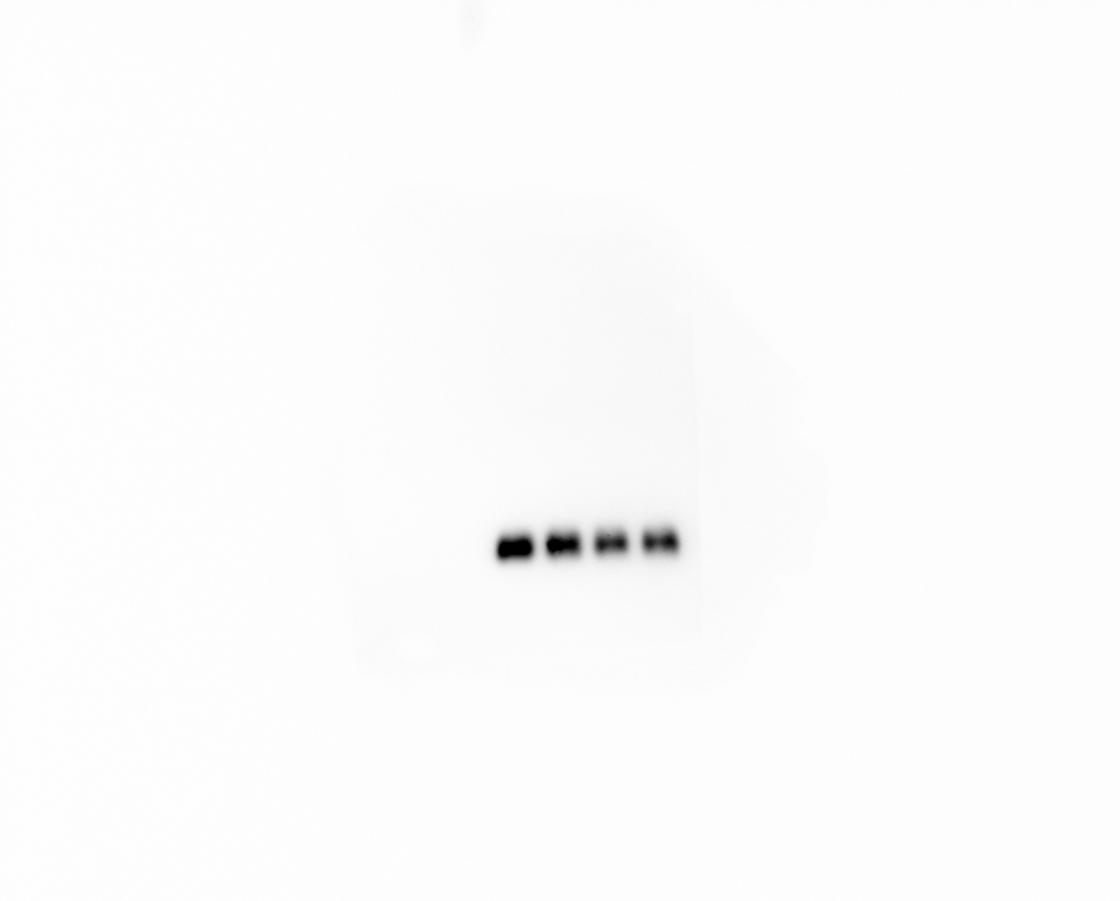

Supplement: Supplementary file 4 — Source Data [file 41467_2023_41520_MOESM4_ESM.zip › Source Data/Uncropped and Unprocessed Scans/Fig. 1b/IB HA.tif]

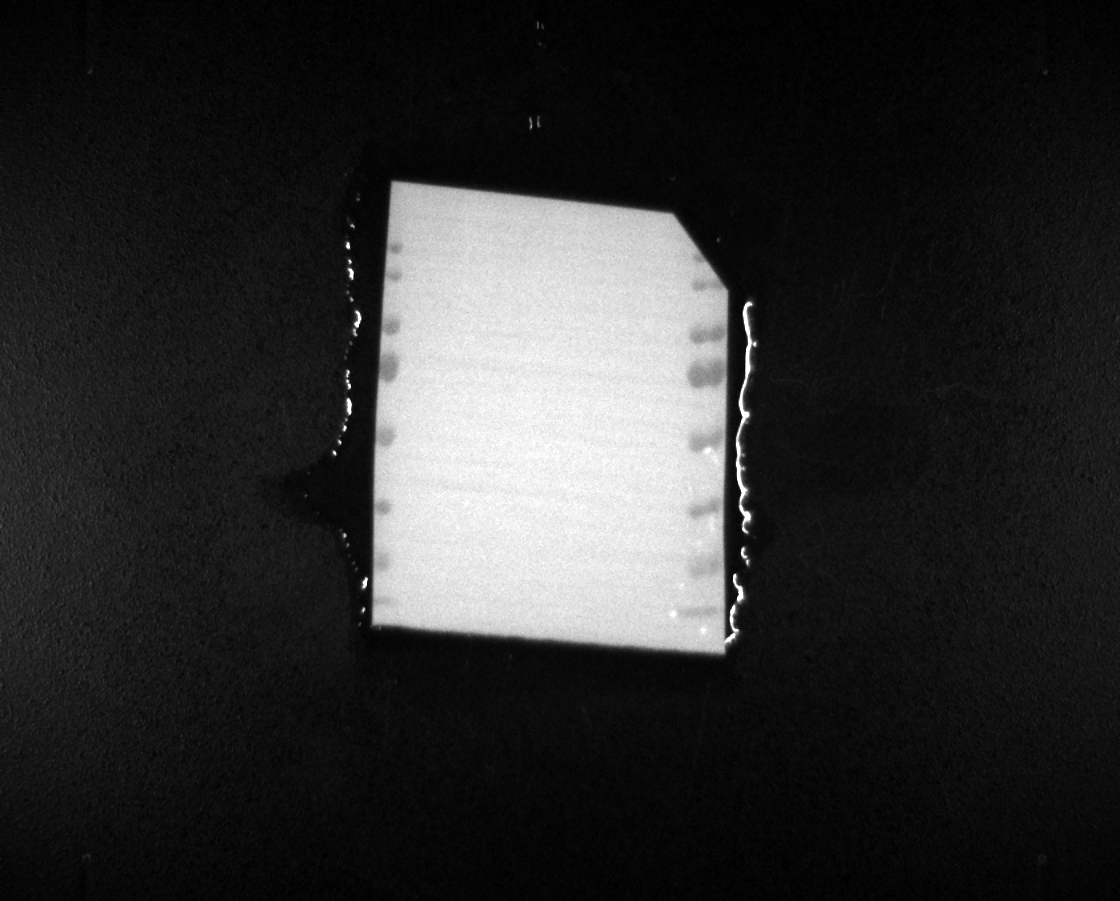

Supplement: Supplementary file 4 — Source Data [file 41467_2023_41520_MOESM4_ESM.zip › Source Data/Uncropped and Unprocessed Scans/Fig. 1b/IB SUMO1 - Marker.tif]

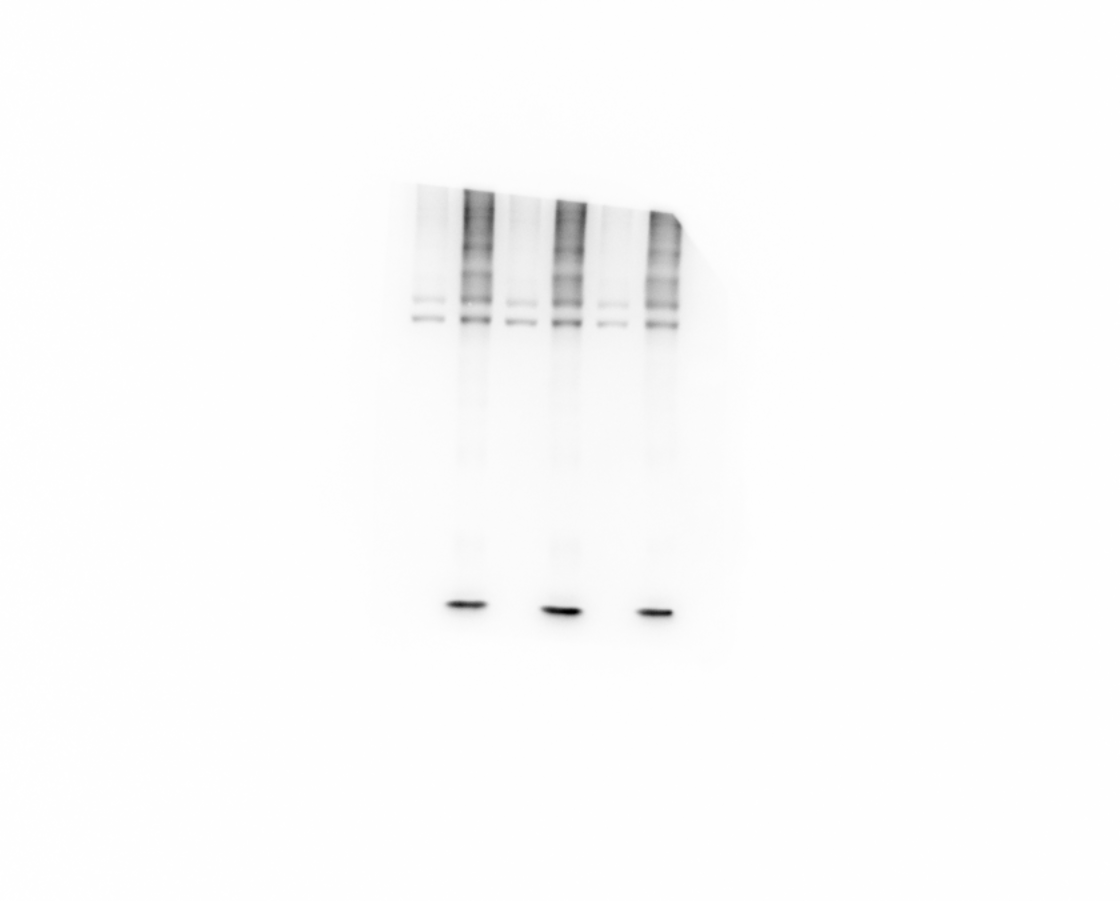

Supplement: Supplementary file 4 — Source Data [file 41467_2023_41520_MOESM4_ESM.zip › Source Data/Uncropped and Unprocessed Scans/Fig. 1b/IB SUMO1.tif]

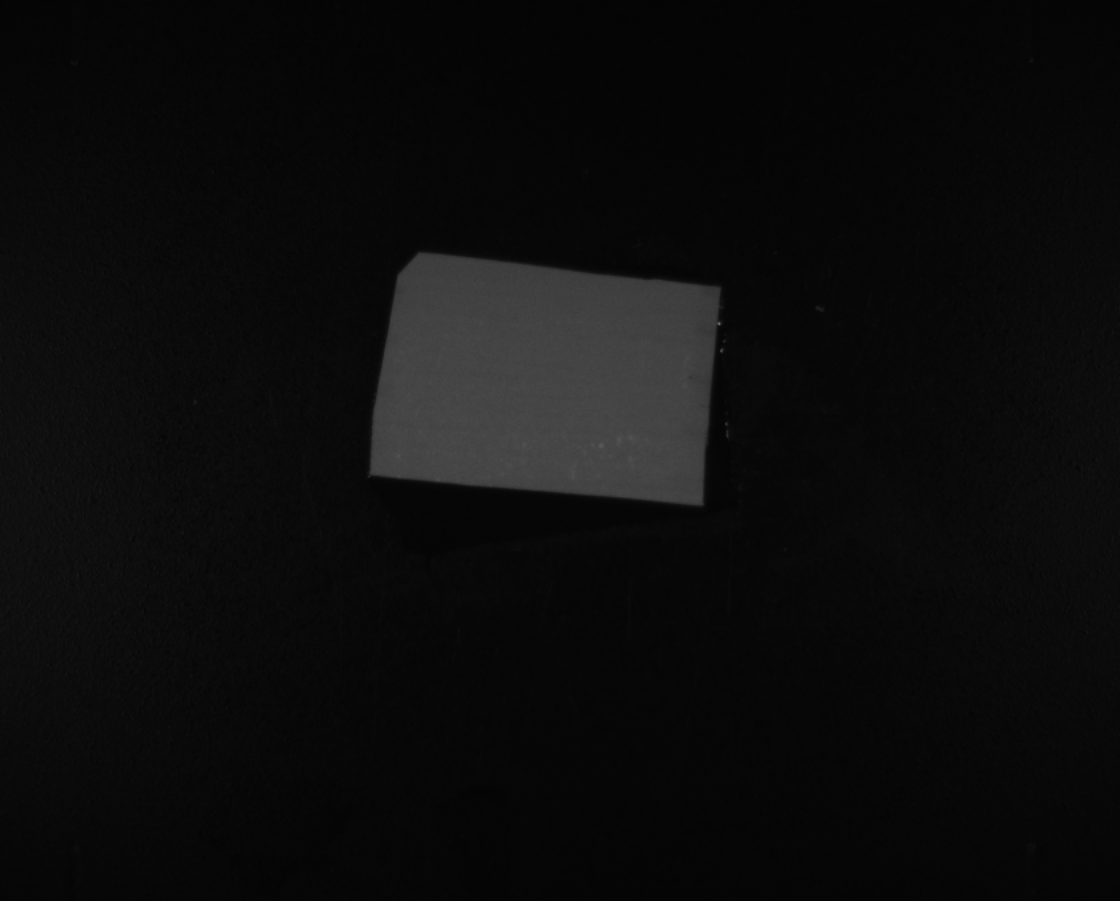

Supplement: Supplementary file 4 — Source Data [file 41467_2023_41520_MOESM4_ESM.zip › Source Data/Uncropped and Unprocessed Scans/Fig. 1b/IP Flag; IB HA - Marker.tif]

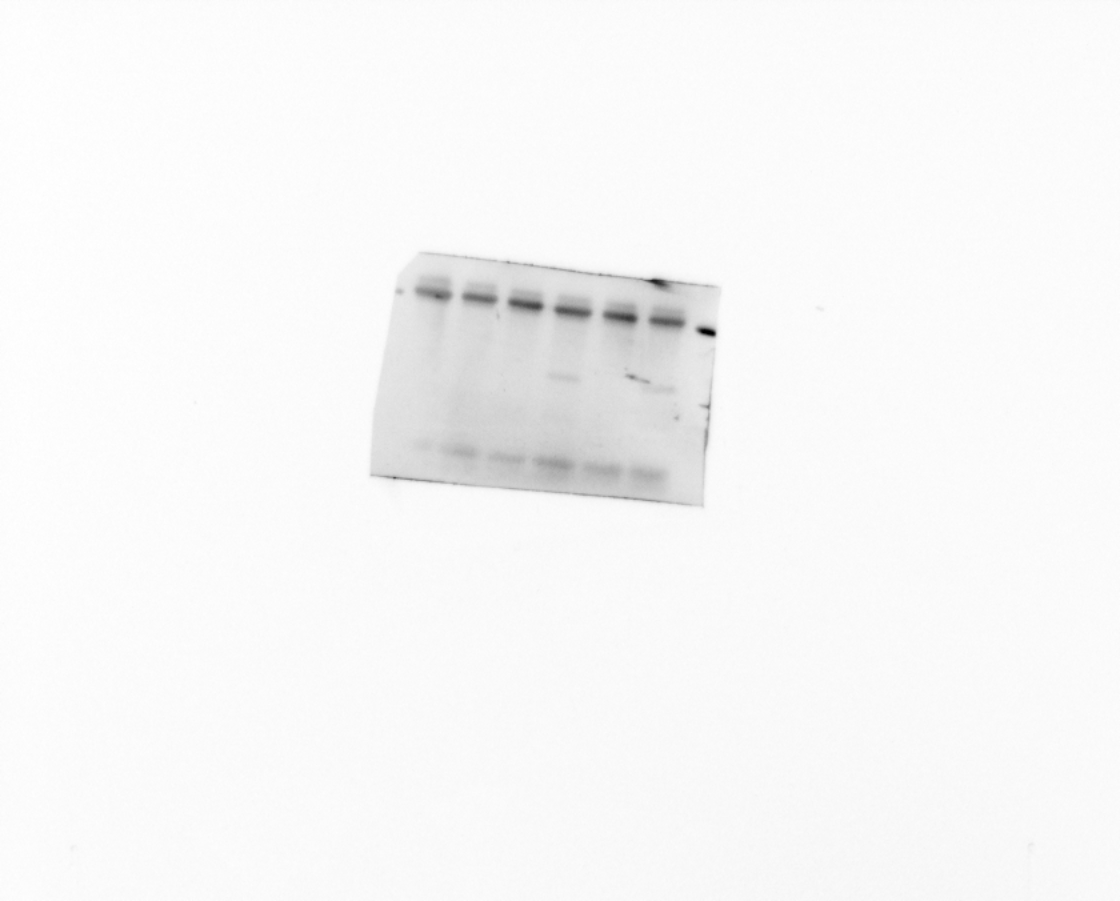

Supplement: Supplementary file 4 — Source Data [file 41467_2023_41520_MOESM4_ESM.zip › Source Data/Uncropped and Unprocessed Scans/Fig. 1b/IP Flag; IB HA.tif]

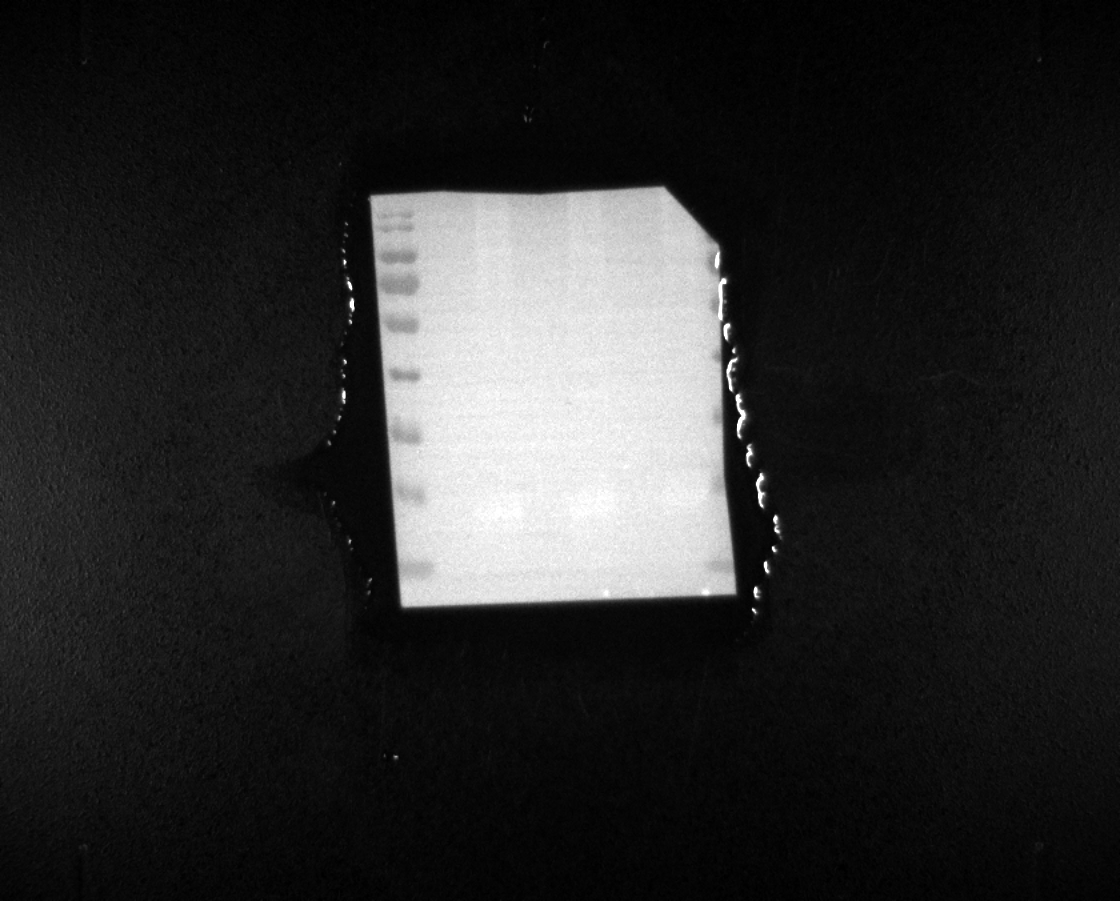

Supplement: Supplementary file 4 — Source Data [file 41467_2023_41520_MOESM4_ESM.zip › Source Data/Uncropped and Unprocessed Scans/Fig. 1b/IP Flag; IB SUMO1 - Marker.tif]

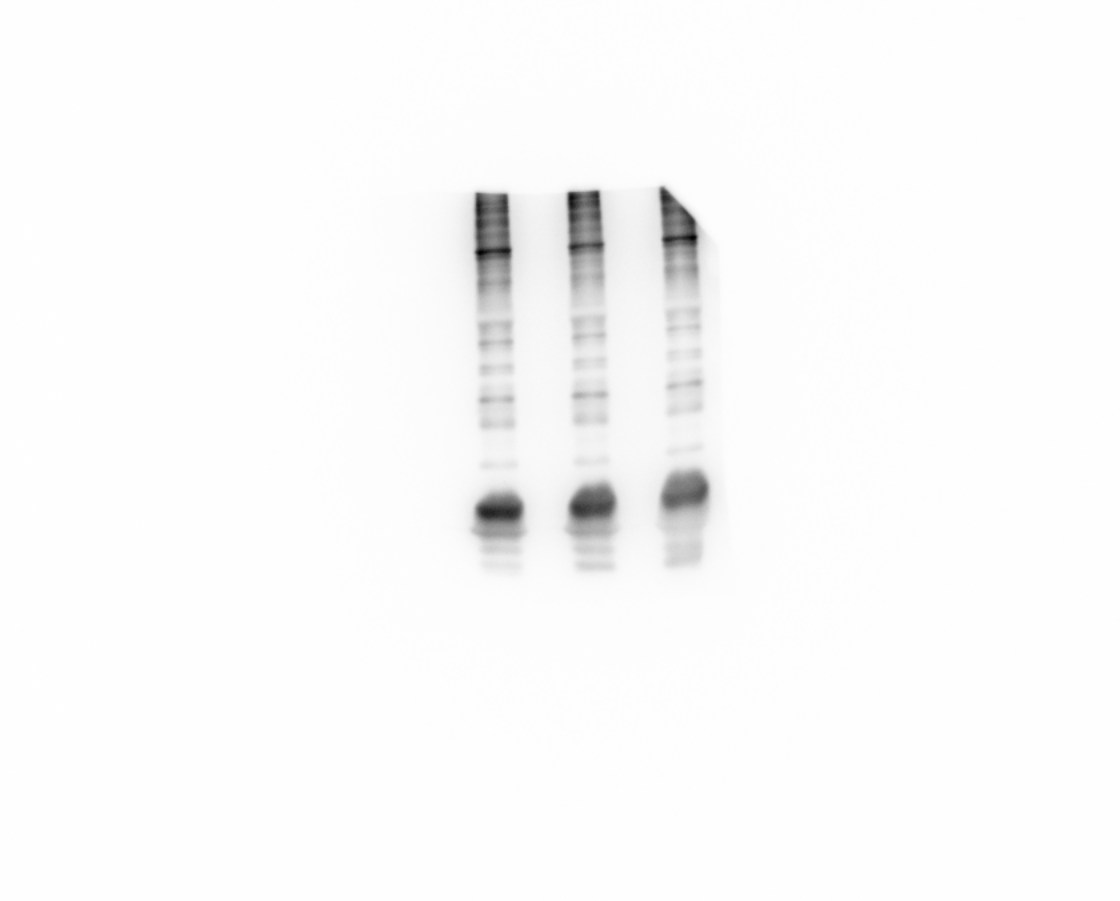

Supplement: Supplementary file 4 — Source Data [file 41467_2023_41520_MOESM4_ESM.zip › Source Data/Uncropped and Unprocessed Scans/Fig. 1b/IP Flag; IB SUMO1.tif]

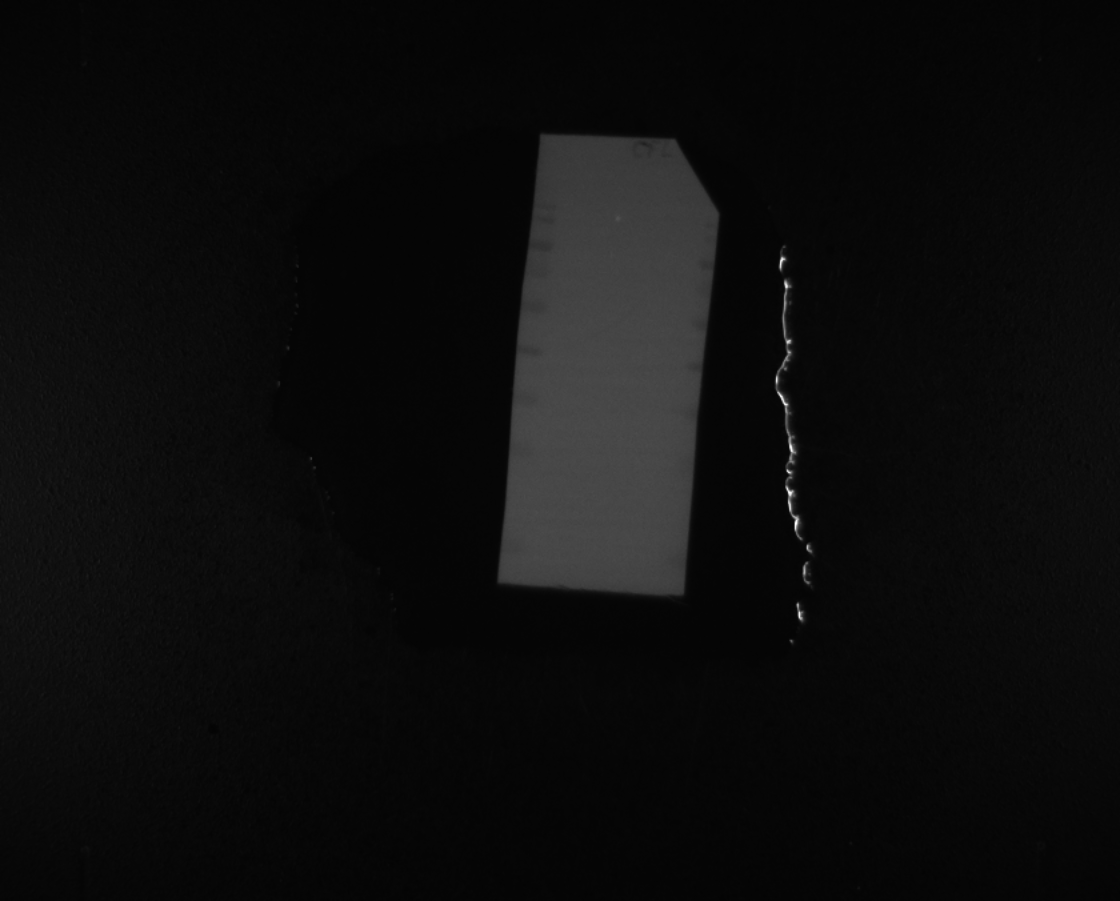

Supplement: Supplementary file 4 — Source Data [file 41467_2023_41520_MOESM4_ESM.zip › Source Data/Uncropped and Unprocessed Scans/Fig. 1c/IB CFL1 - Marker.tif]

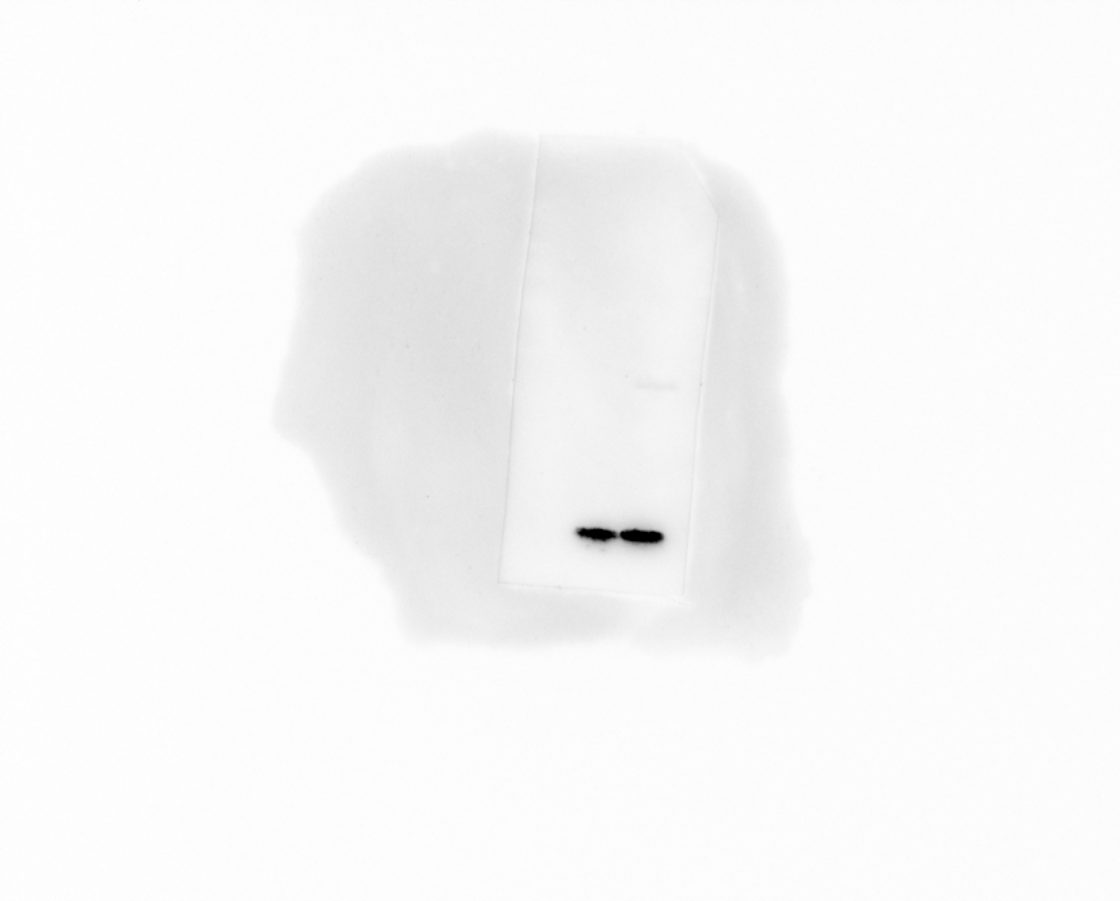

Supplement: Supplementary file 4 — Source Data [file 41467_2023_41520_MOESM4_ESM.zip › Source Data/Uncropped and Unprocessed Scans/Fig. 1c/IB CFL1.tif]

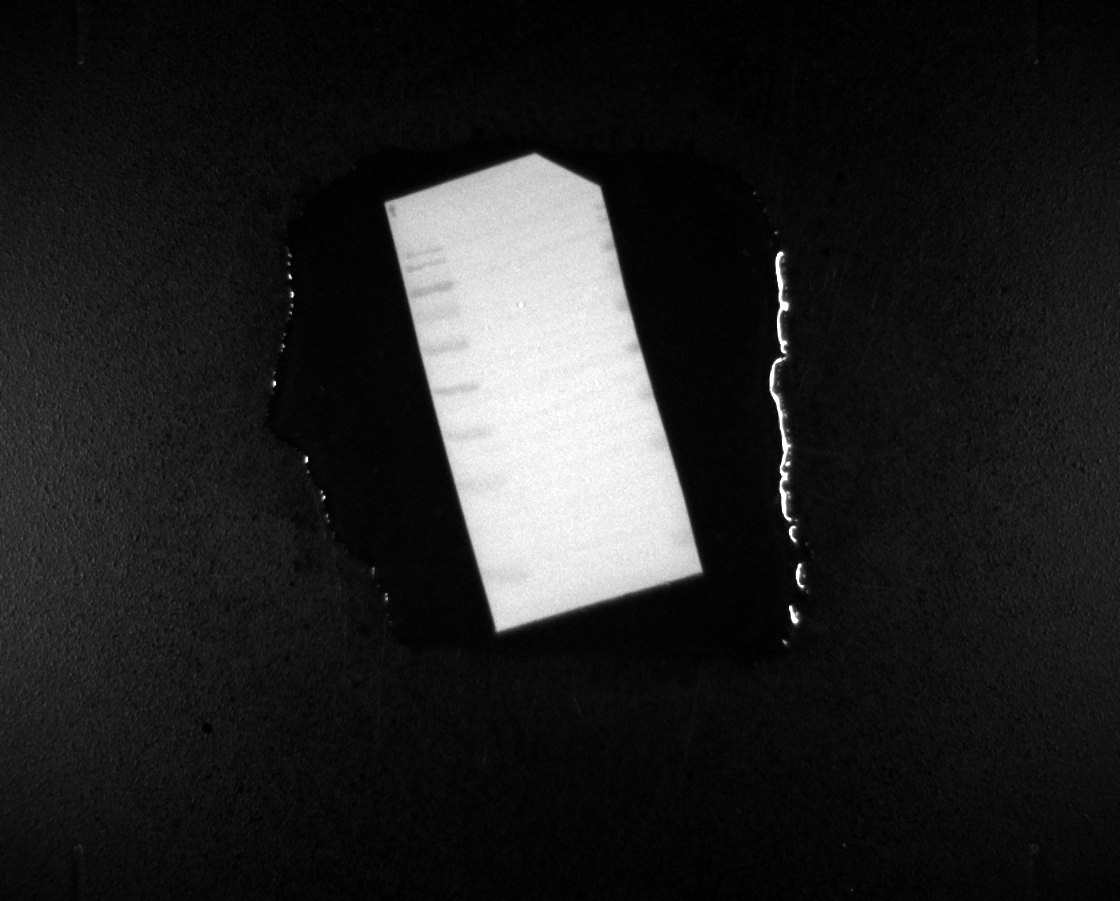

Supplement: Supplementary file 4 — Source Data [file 41467_2023_41520_MOESM4_ESM.zip › Source Data/Uncropped and Unprocessed Scans/Fig. 1c/IB SUMO1 - Marker.tif]

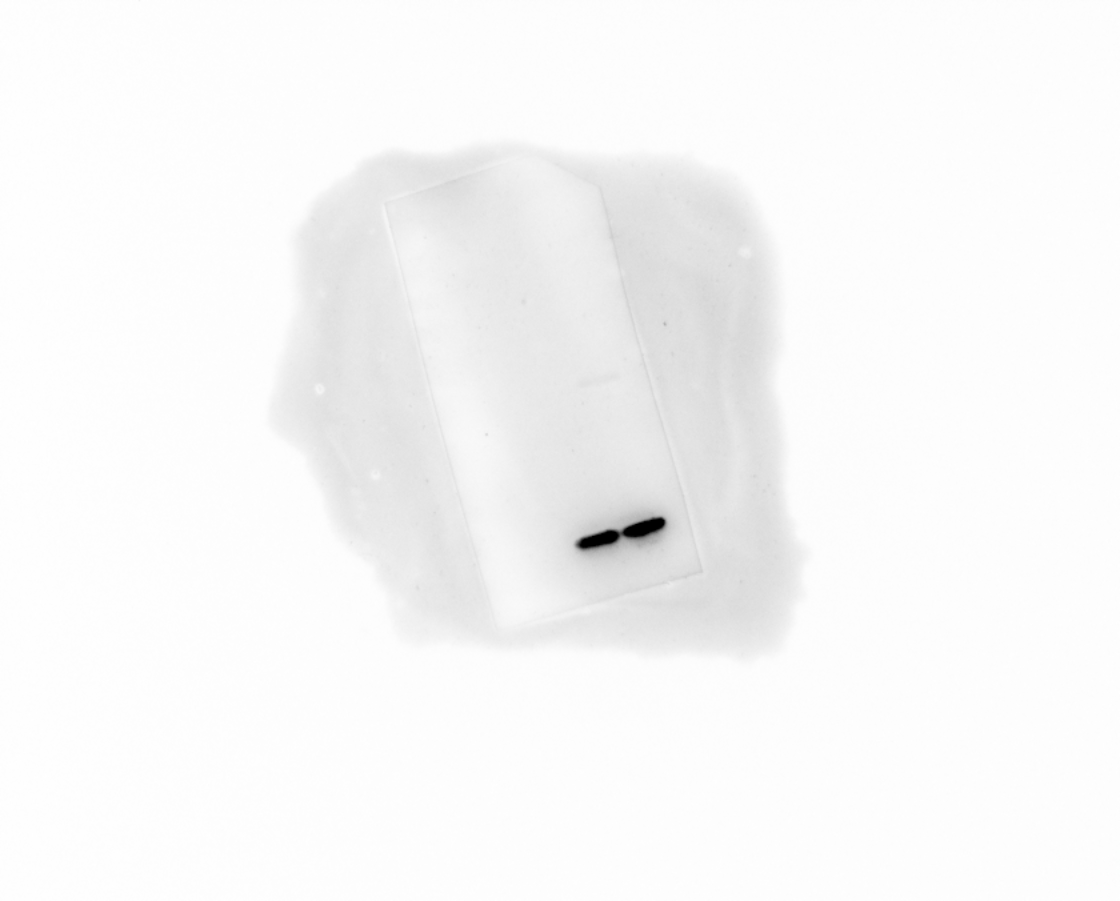

Supplement: Supplementary file 4 — Source Data [file 41467_2023_41520_MOESM4_ESM.zip › Source Data/Uncropped and Unprocessed Scans/Fig. 1c/IB SUMO1.tif]

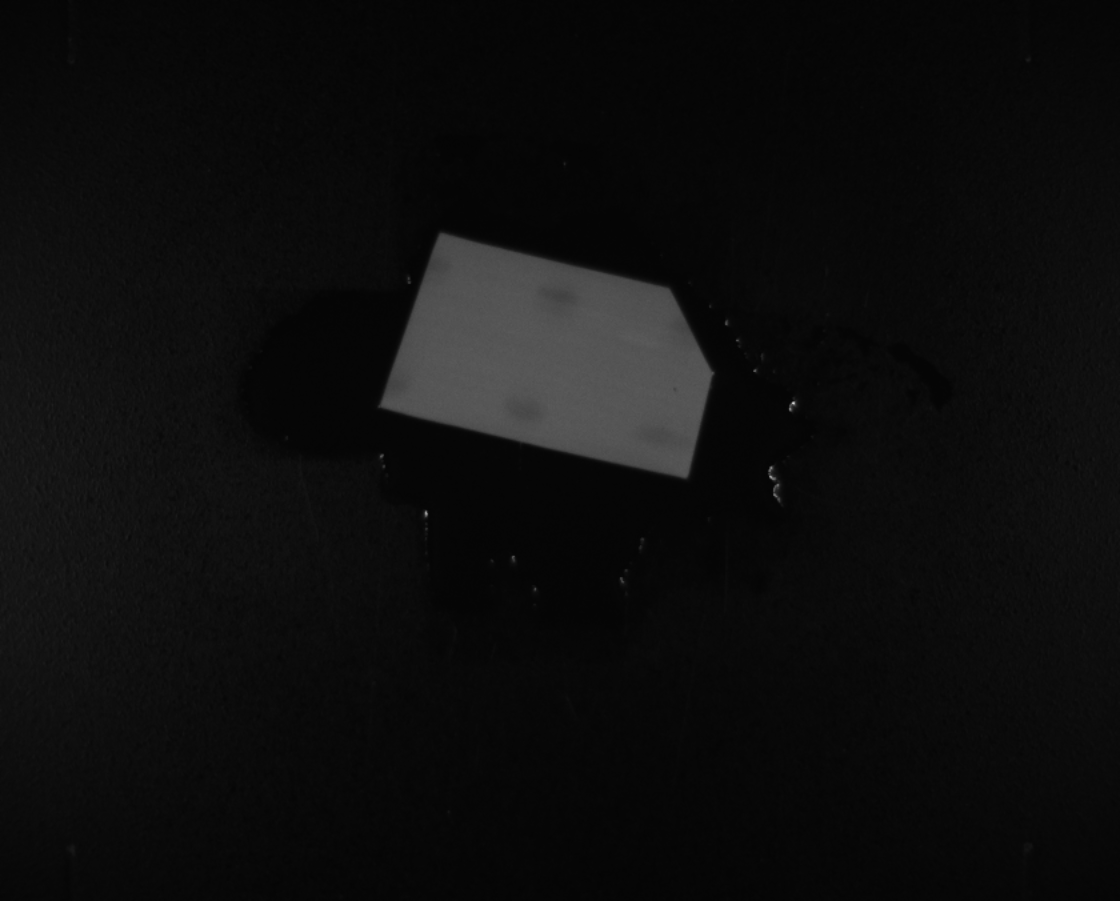

Supplement: Supplementary file 4 — Source Data [file 41467_2023_41520_MOESM4_ESM.zip › Source Data/Uncropped and Unprocessed Scans/Fig. 1d/(Left) IB CFL1(rabbit) - Marker.tif]

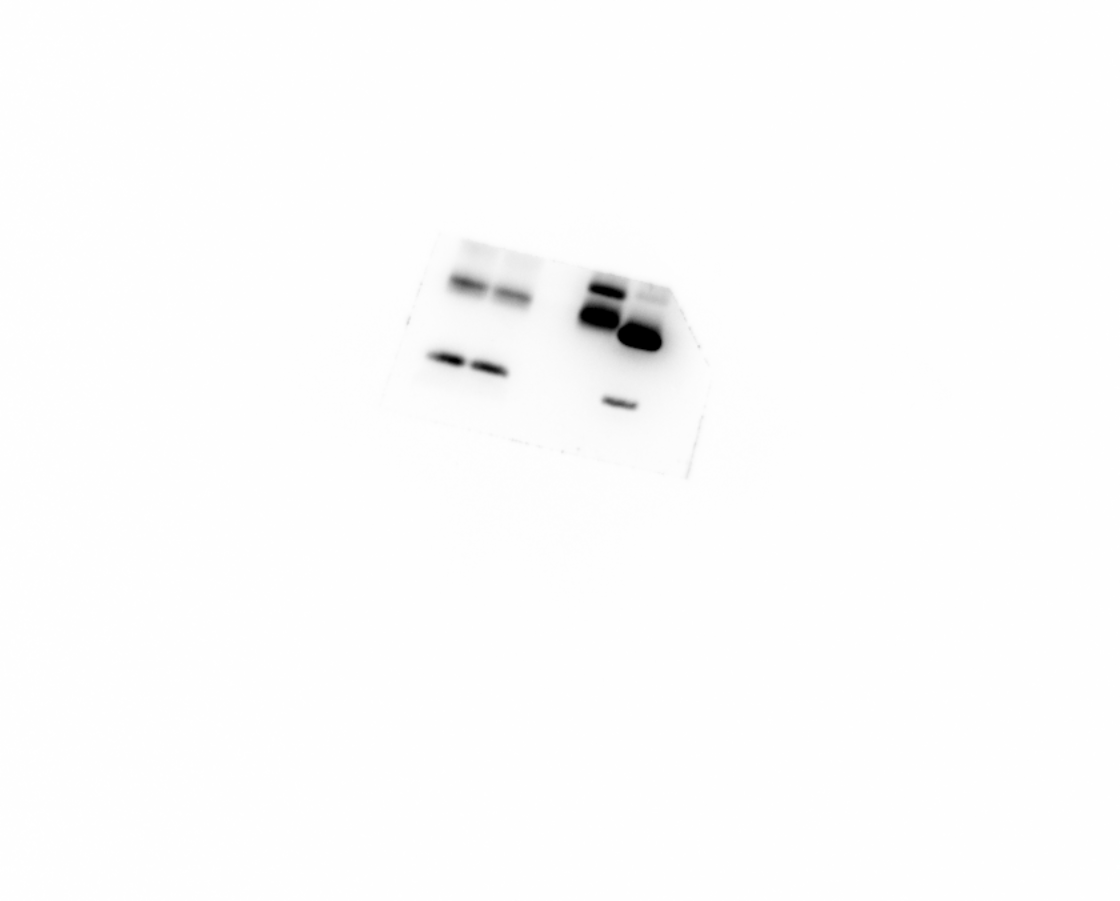

Supplement: Supplementary file 4 — Source Data [file 41467_2023_41520_MOESM4_ESM.zip › Source Data/Uncropped and Unprocessed Scans/Fig. 1d/(Left) IB CFL1(rabbit).tif]

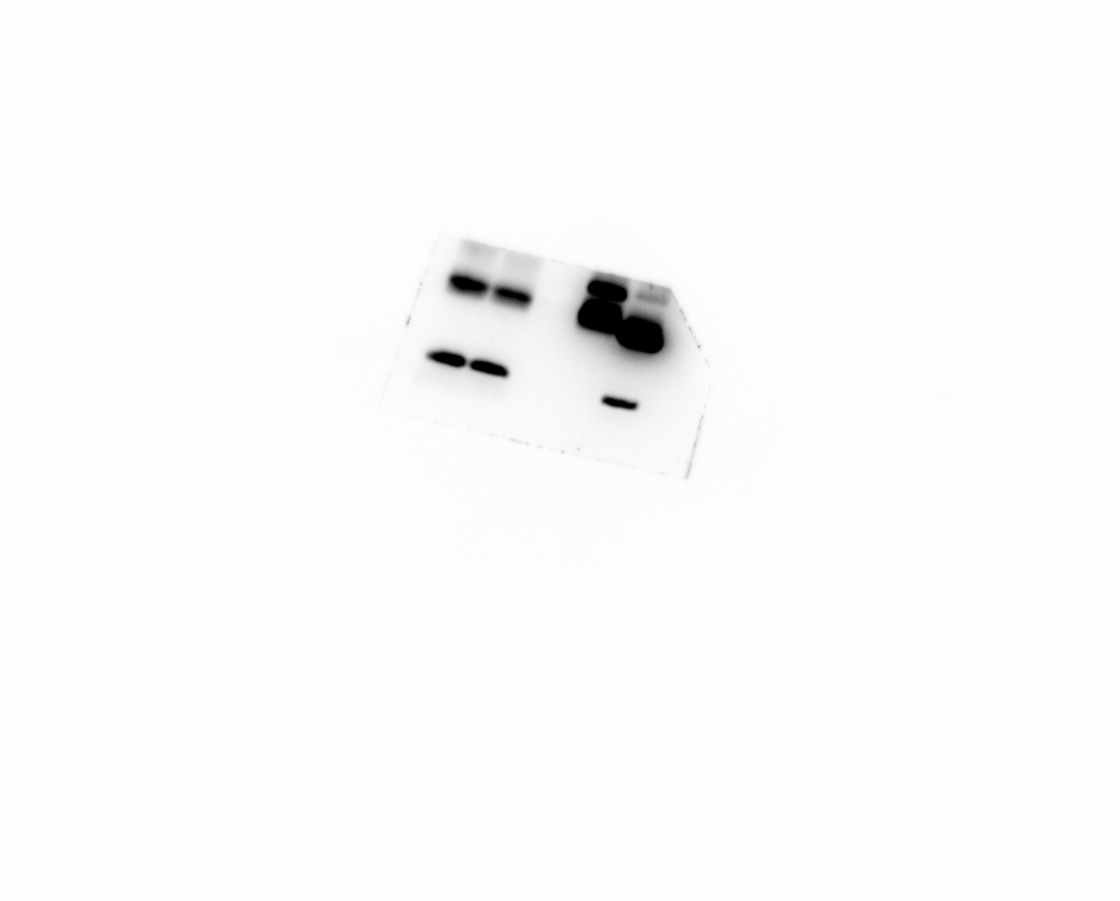

Supplement: Supplementary file 4 — Source Data [file 41467_2023_41520_MOESM4_ESM.zip › Source Data/Uncropped and Unprocessed Scans/Fig. 1d/(Right) IP CFL1(mouse); IB CFL1(rabbit).tif]

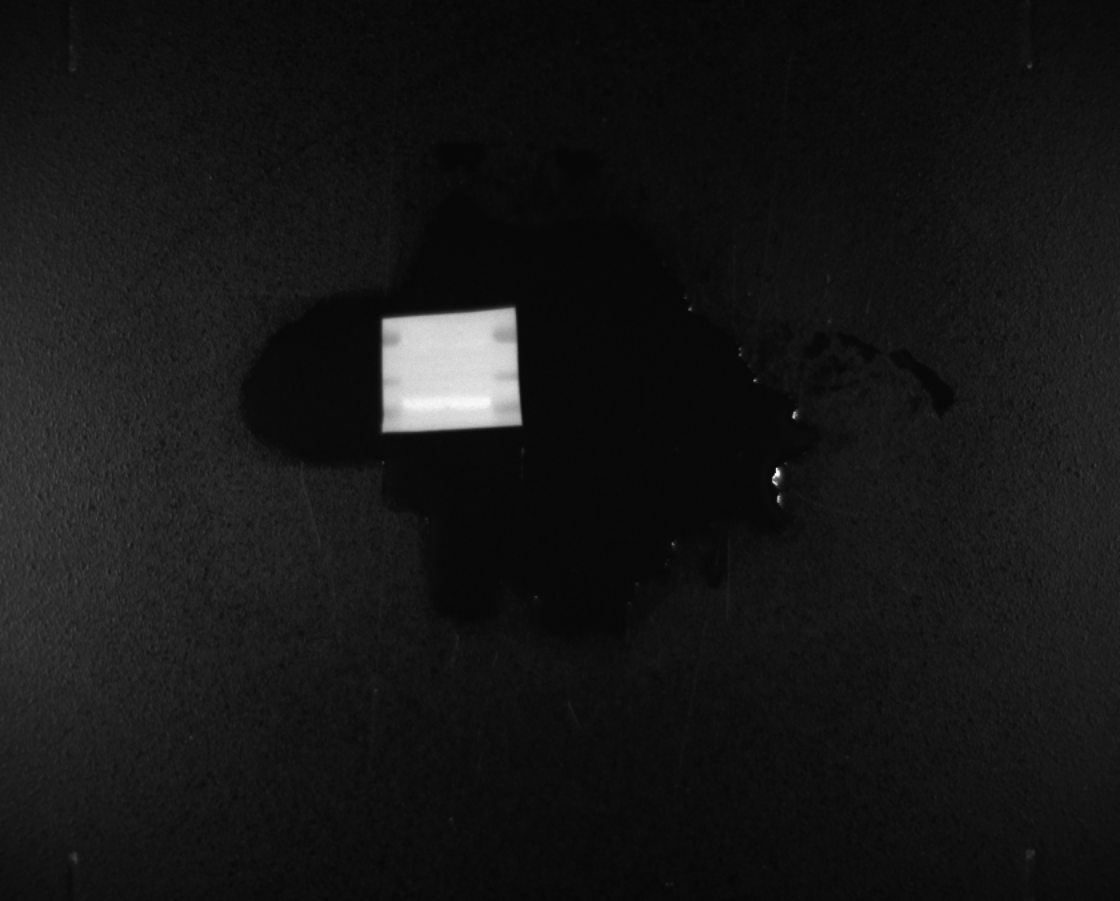

Supplement: Supplementary file 4 — Source Data [file 41467_2023_41520_MOESM4_ESM.zip › Source Data/Uncropped and Unprocessed Scans/Fig. 1d/IB GAPDH - Marker.tif]

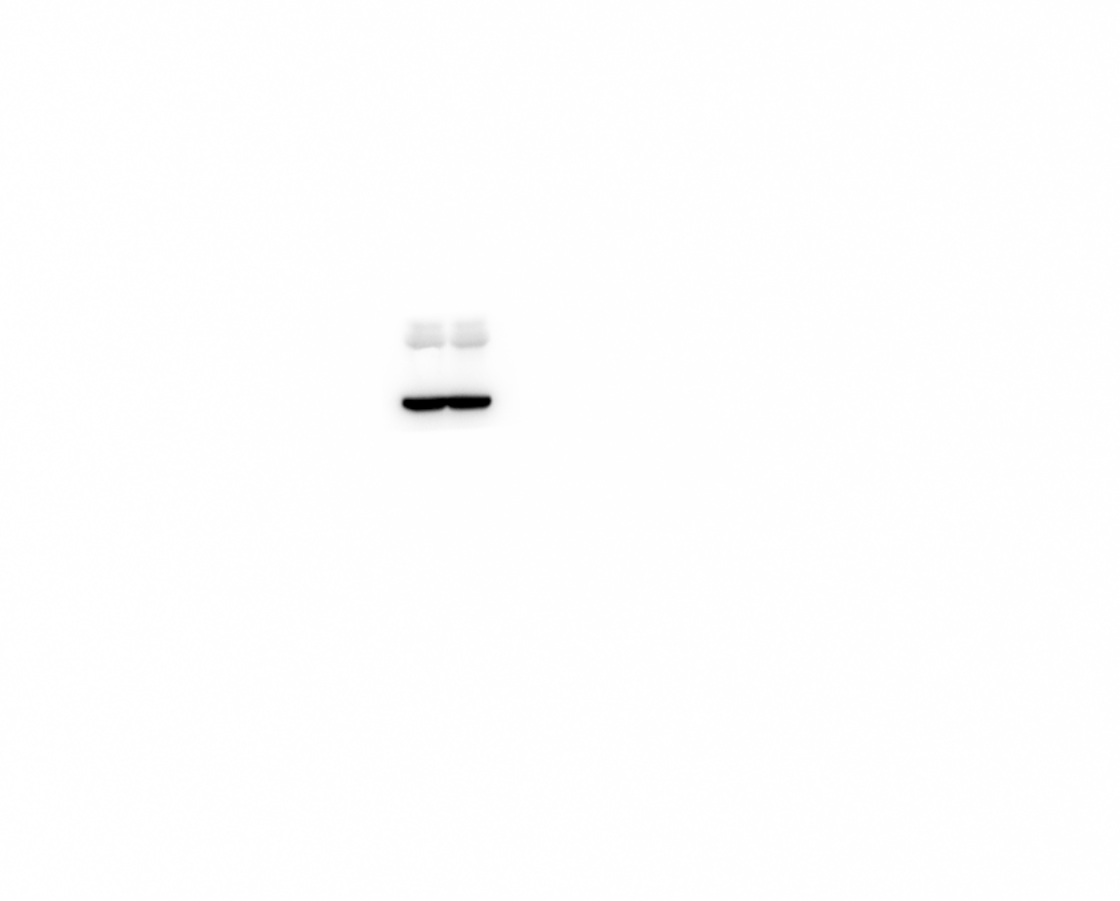

Supplement: Supplementary file 4 — Source Data [file 41467_2023_41520_MOESM4_ESM.zip › Source Data/Uncropped and Unprocessed Scans/Fig. 1d/IB GAPDH.tif]

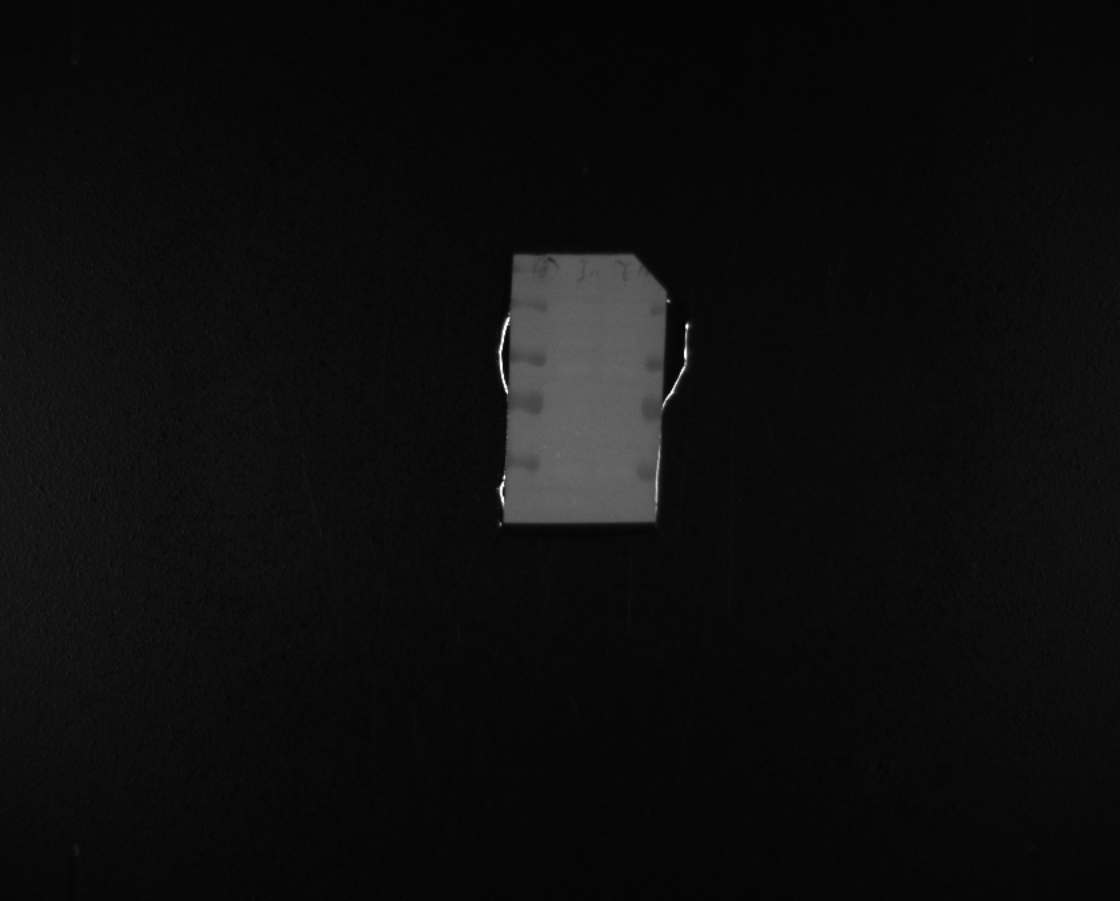

Supplement: Supplementary file 4 — Source Data [file 41467_2023_41520_MOESM4_ESM.zip › Source Data/Uncropped and Unprocessed Scans/Fig. 1d/IB SUMO1 - Marker.tif]

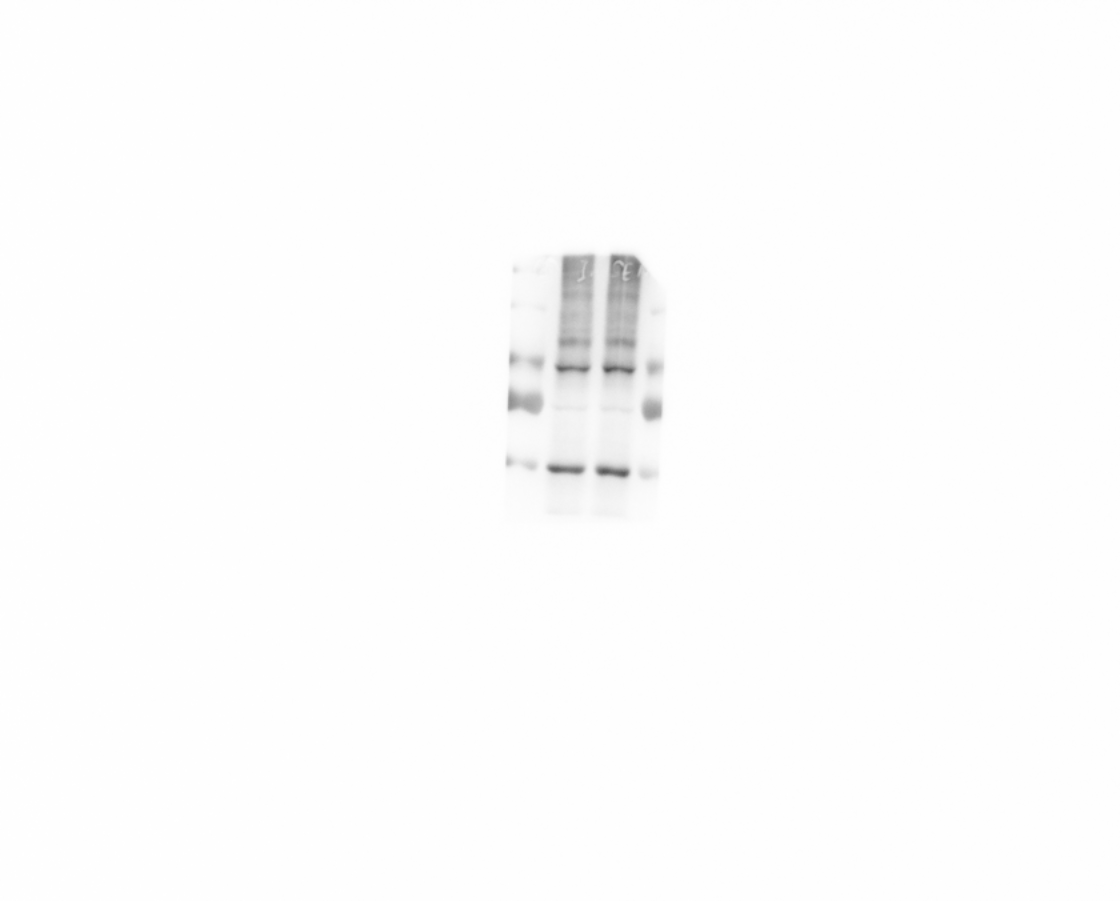

Supplement: Supplementary file 4 — Source Data [file 41467_2023_41520_MOESM4_ESM.zip › Source Data/Uncropped and Unprocessed Scans/Fig. 1d/IB SUMO1.tif]

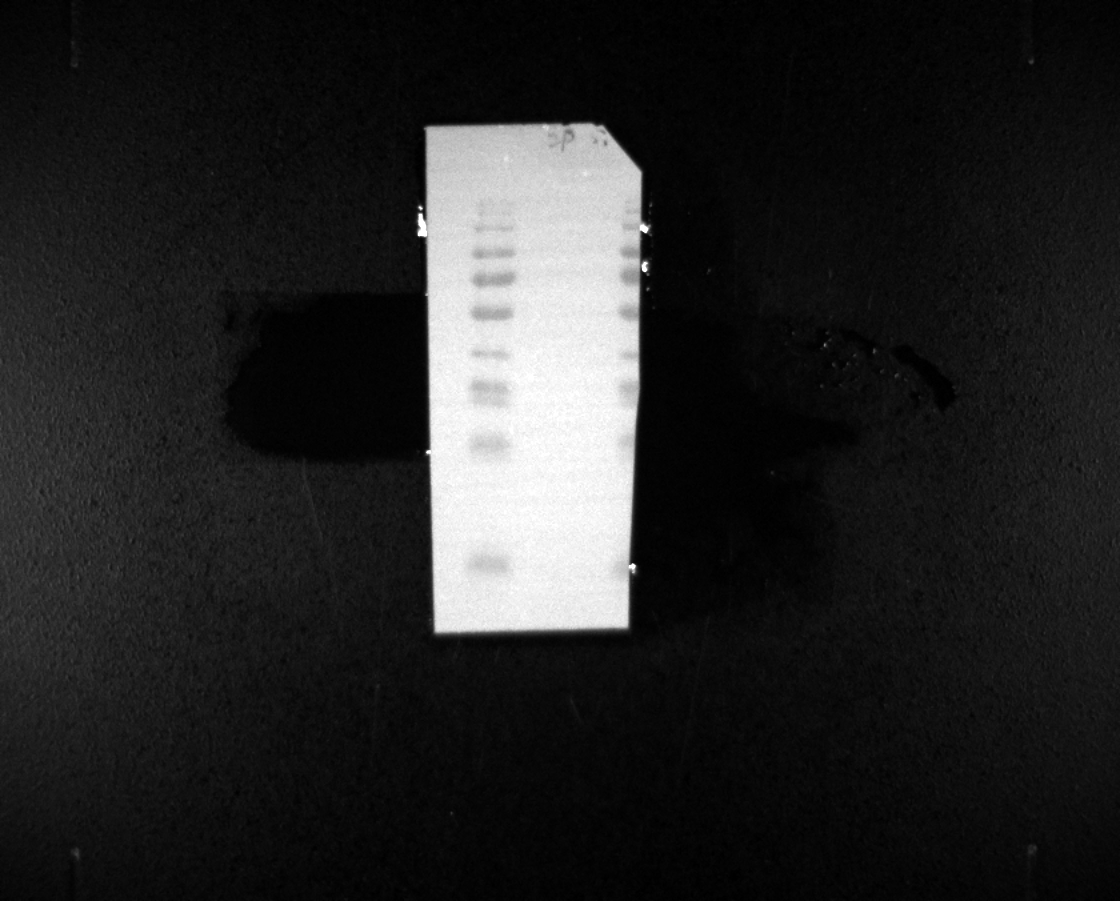

Supplement: Supplementary file 4 — Source Data [file 41467_2023_41520_MOESM4_ESM.zip › Source Data/Uncropped and Unprocessed Scans/Fig. 1d/IP CFL1(mouse); IB SUMO1 - Marker.tif]

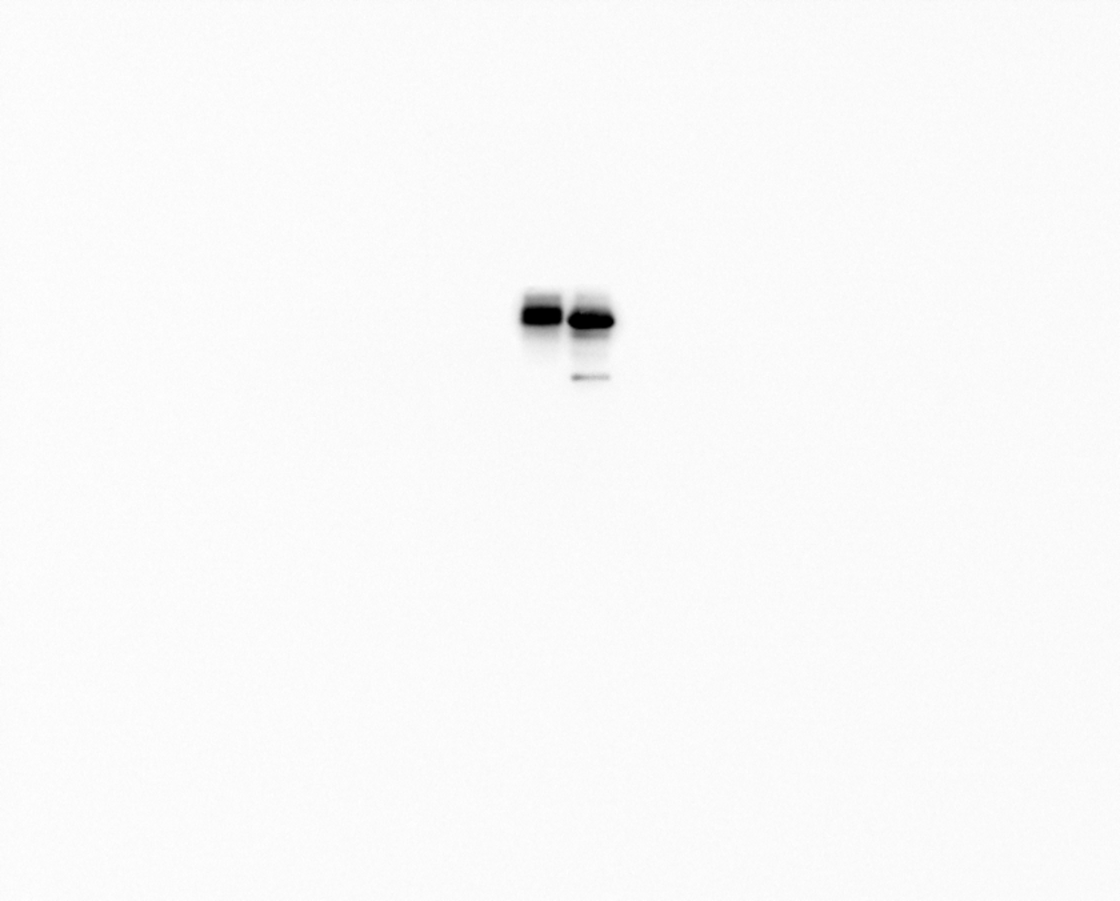

Supplement: Supplementary file 4 — Source Data [file 41467_2023_41520_MOESM4_ESM.zip › Source Data/Uncropped and Unprocessed Scans/Fig. 1d/IP CFL1(mouse); IB SUMO1.tif]

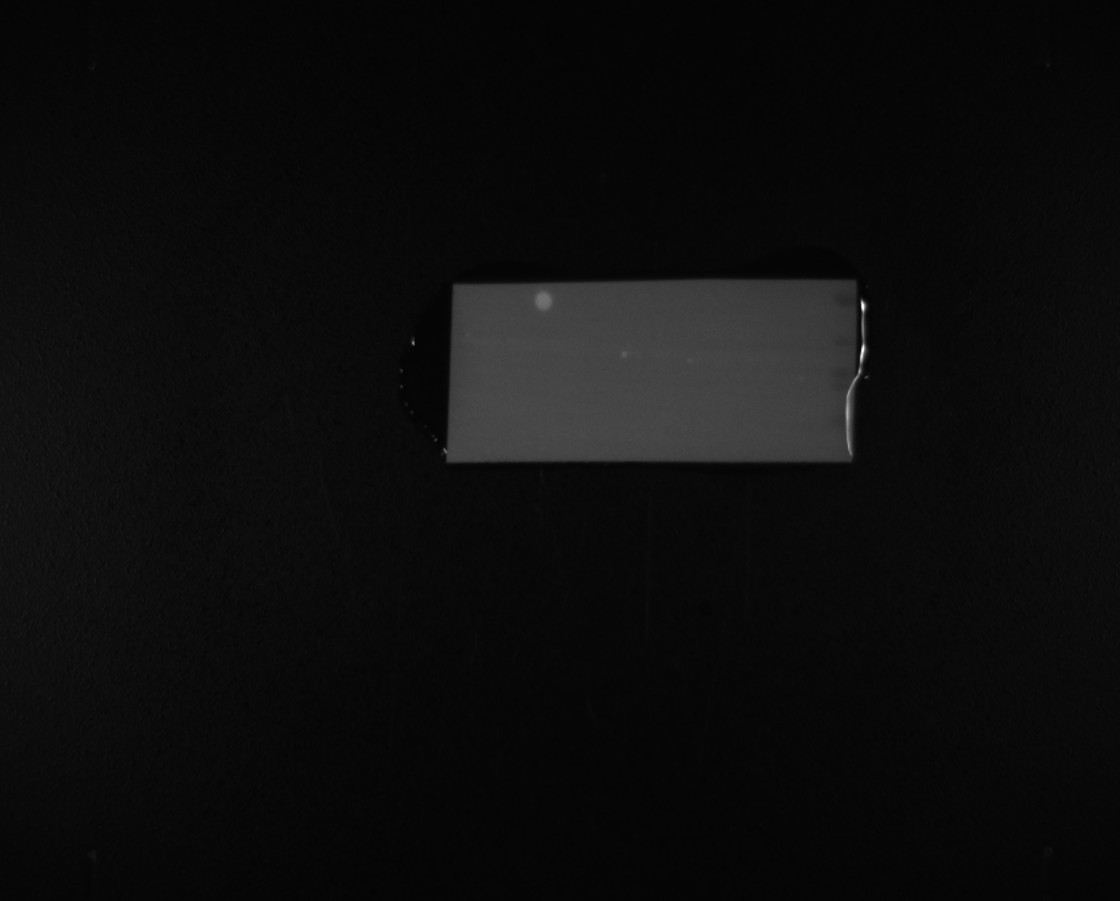

Supplement: Supplementary file 4 — Source Data [file 41467_2023_41520_MOESM4_ESM.zip › Source Data/Uncropped and Unprocessed Scans/Fig. 2c/IB GAPDH (Left 5 columns) - Marker.tif]

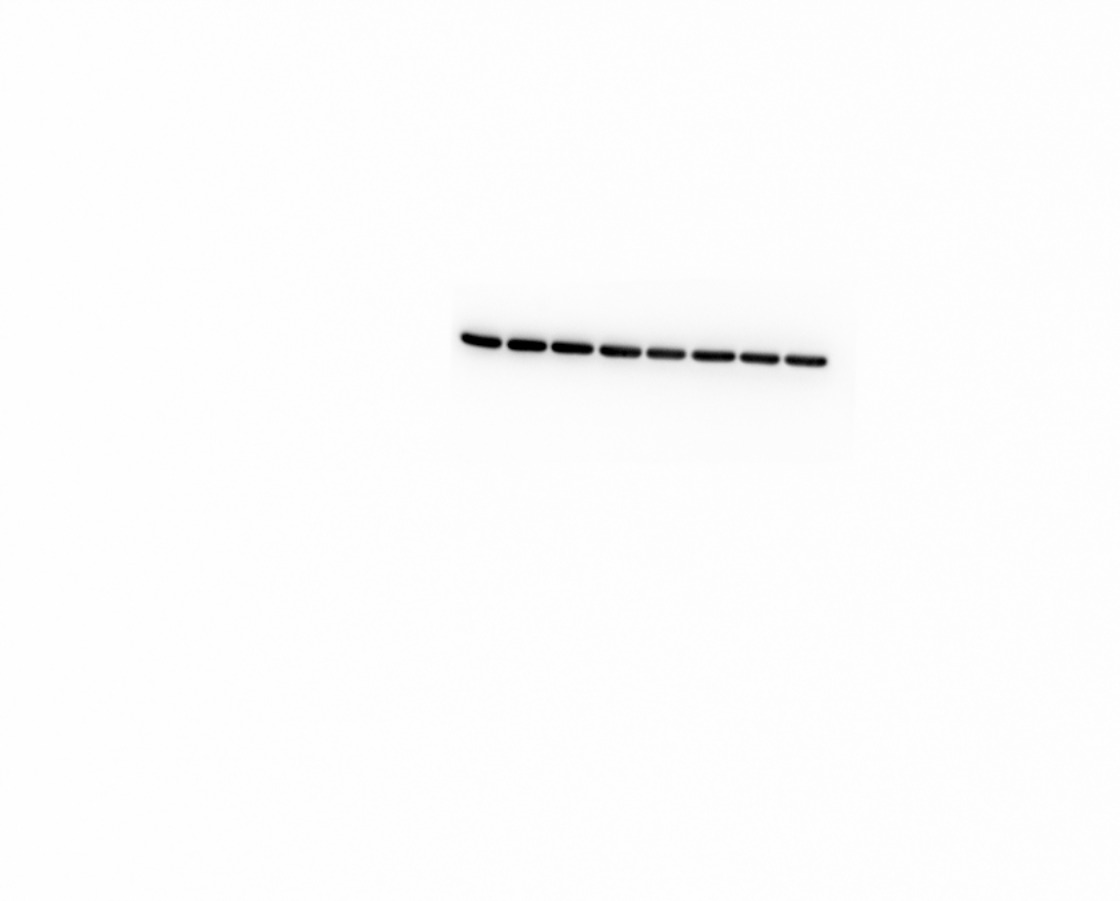

Supplement: Supplementary file 4 — Source Data [file 41467_2023_41520_MOESM4_ESM.zip › Source Data/Uncropped and Unprocessed Scans/Fig. 2c/IB GAPDH (Left 5 columns).tif]

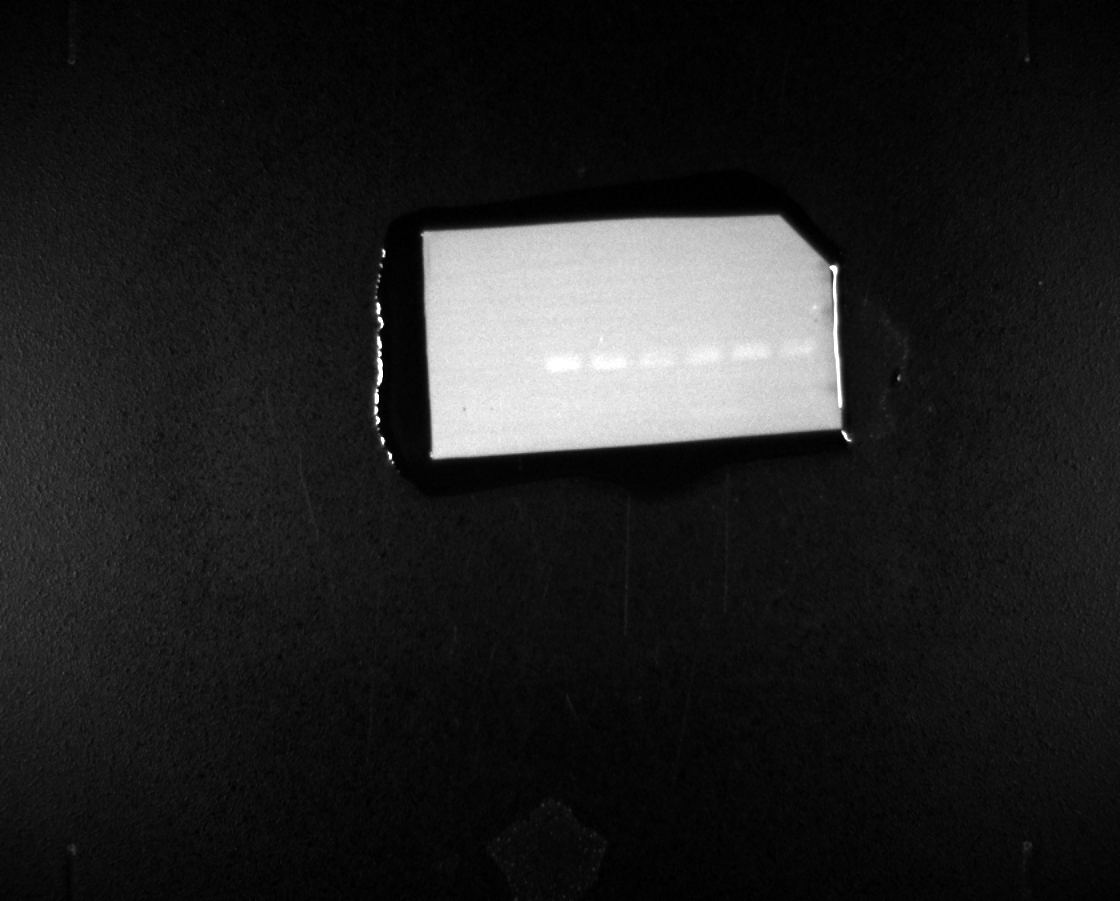

Supplement: Supplementary file 4 — Source Data [file 41467_2023_41520_MOESM4_ESM.zip › Source Data/Uncropped and Unprocessed Scans/Fig. 2c/IB HA (Left 5 columns) - Marker.tif]

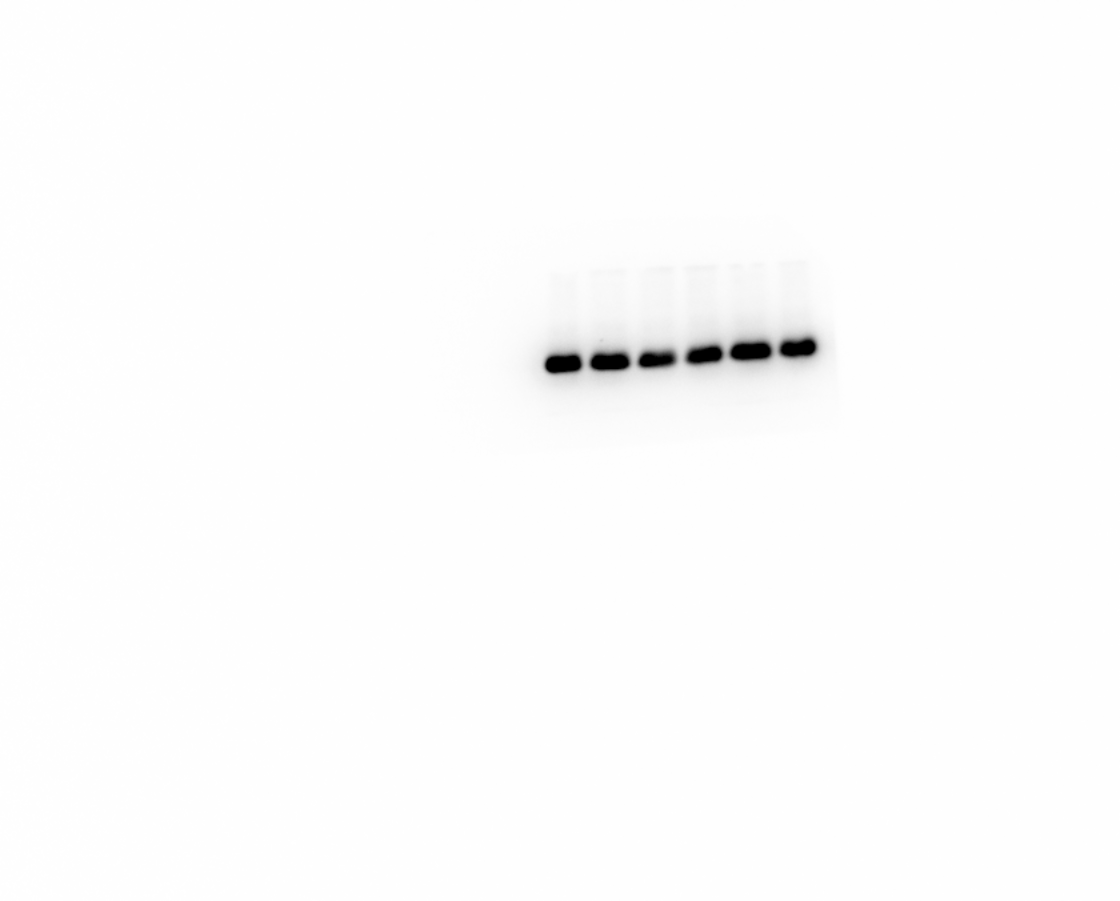

Supplement: Supplementary file 4 — Source Data [file 41467_2023_41520_MOESM4_ESM.zip › Source Data/Uncropped and Unprocessed Scans/Fig. 2c/IB HA (Left 5 columns).tif]

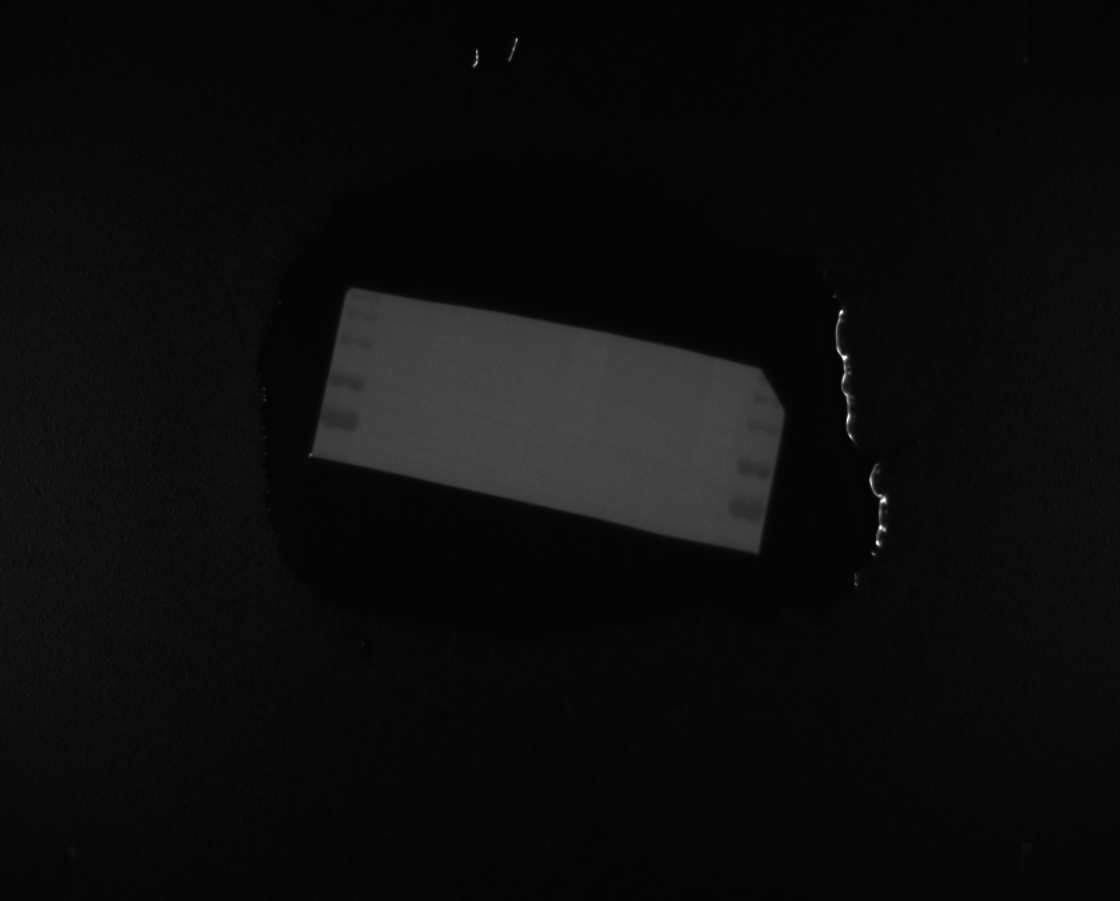

Supplement: Supplementary file 4 — Source Data [file 41467_2023_41520_MOESM4_ESM.zip › Source Data/Uncropped and Unprocessed Scans/Fig. 2c/IB SUMO1 (Left 5 columns) - Marker.tif]

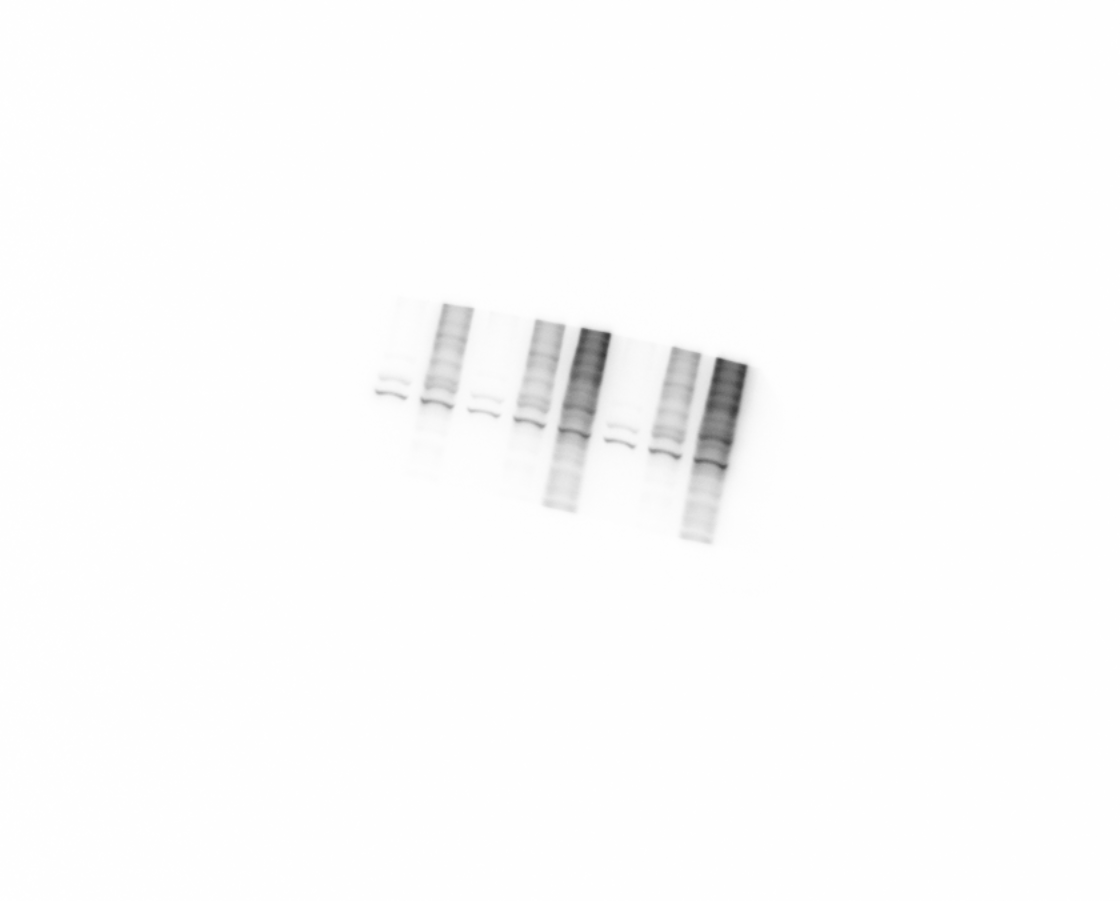

Supplement: Supplementary file 4 — Source Data [file 41467_2023_41520_MOESM4_ESM.zip › Source Data/Uncropped and Unprocessed Scans/Fig. 2c/IB SUMO1 (Left 5 columns).tif]

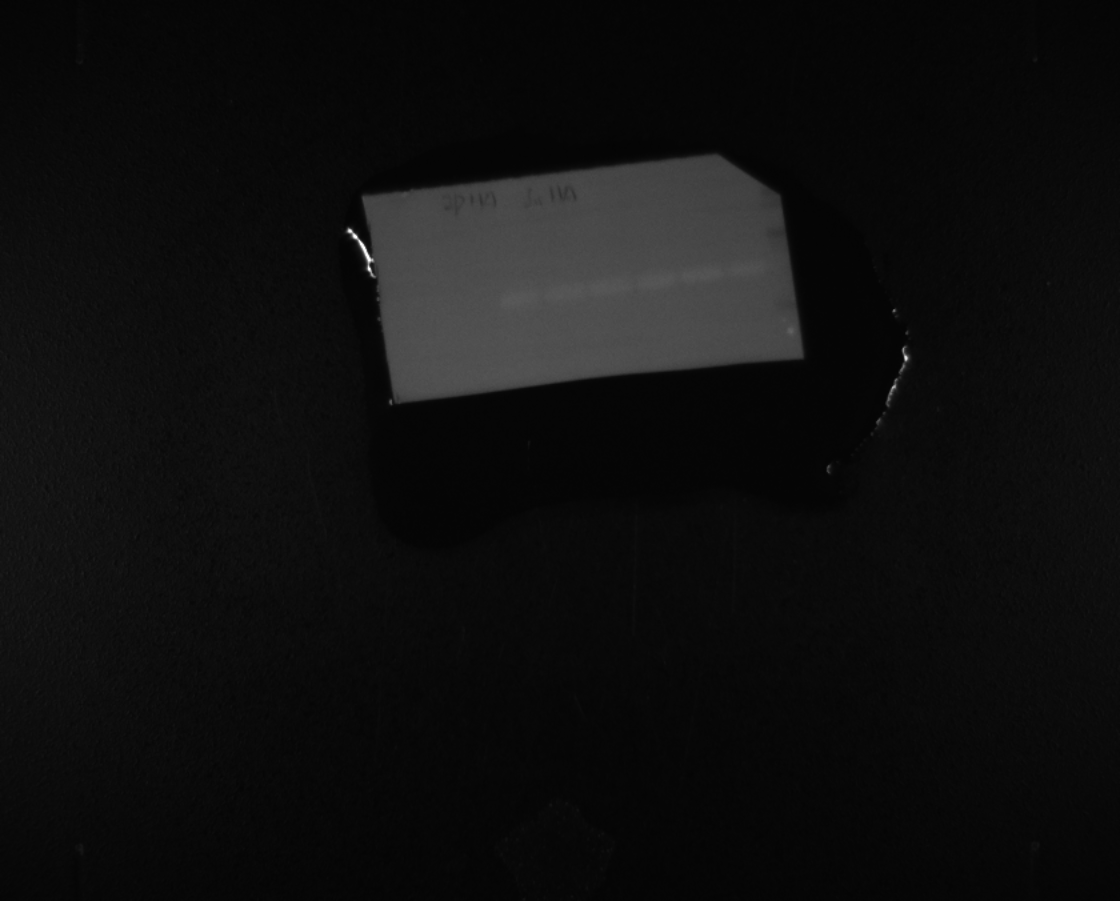

Supplement: Supplementary file 4 — Source Data [file 41467_2023_41520_MOESM4_ESM.zip › Source Data/Uncropped and Unprocessed Scans/Fig. 2c/IP HA; IB HA (Left 5 columns) - Marker.tif]

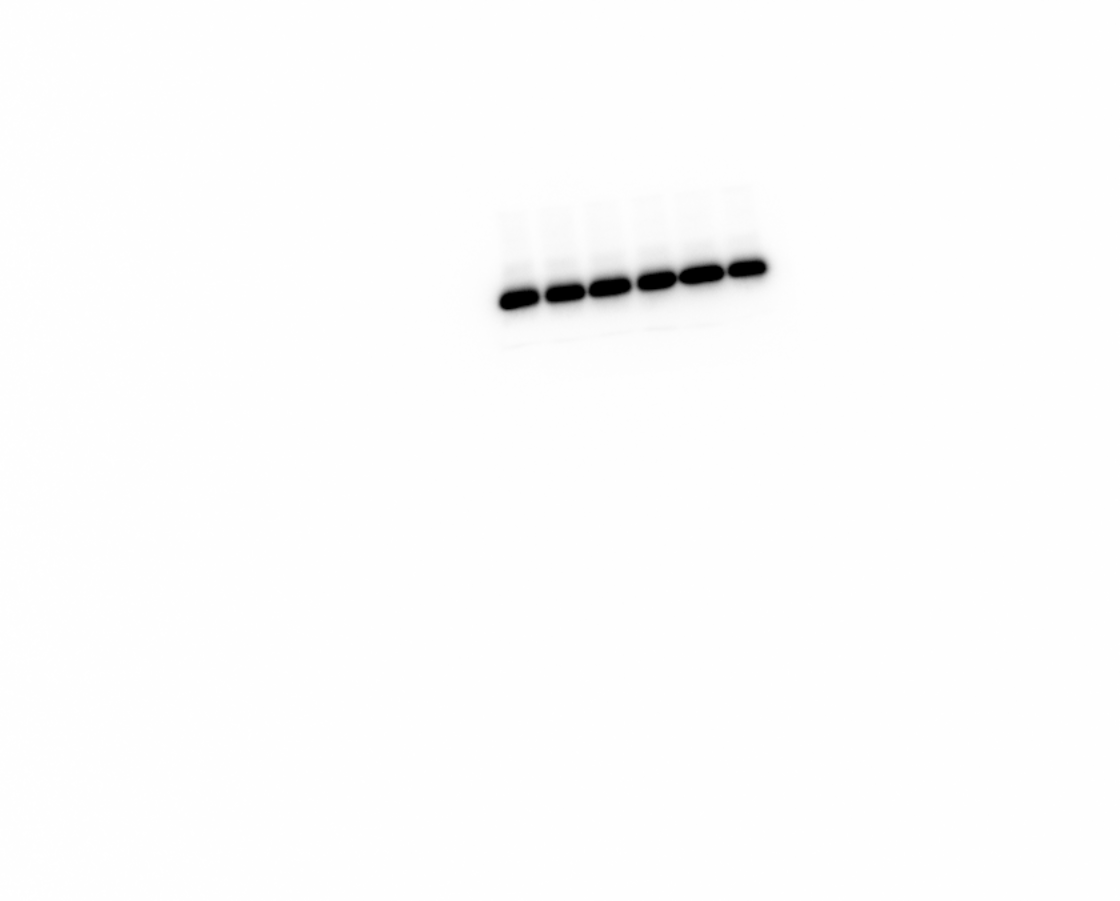

Supplement: Supplementary file 4 — Source Data [file 41467_2023_41520_MOESM4_ESM.zip › Source Data/Uncropped and Unprocessed Scans/Fig. 2c/IP HA; IB HA (Left 5 columns).tif]

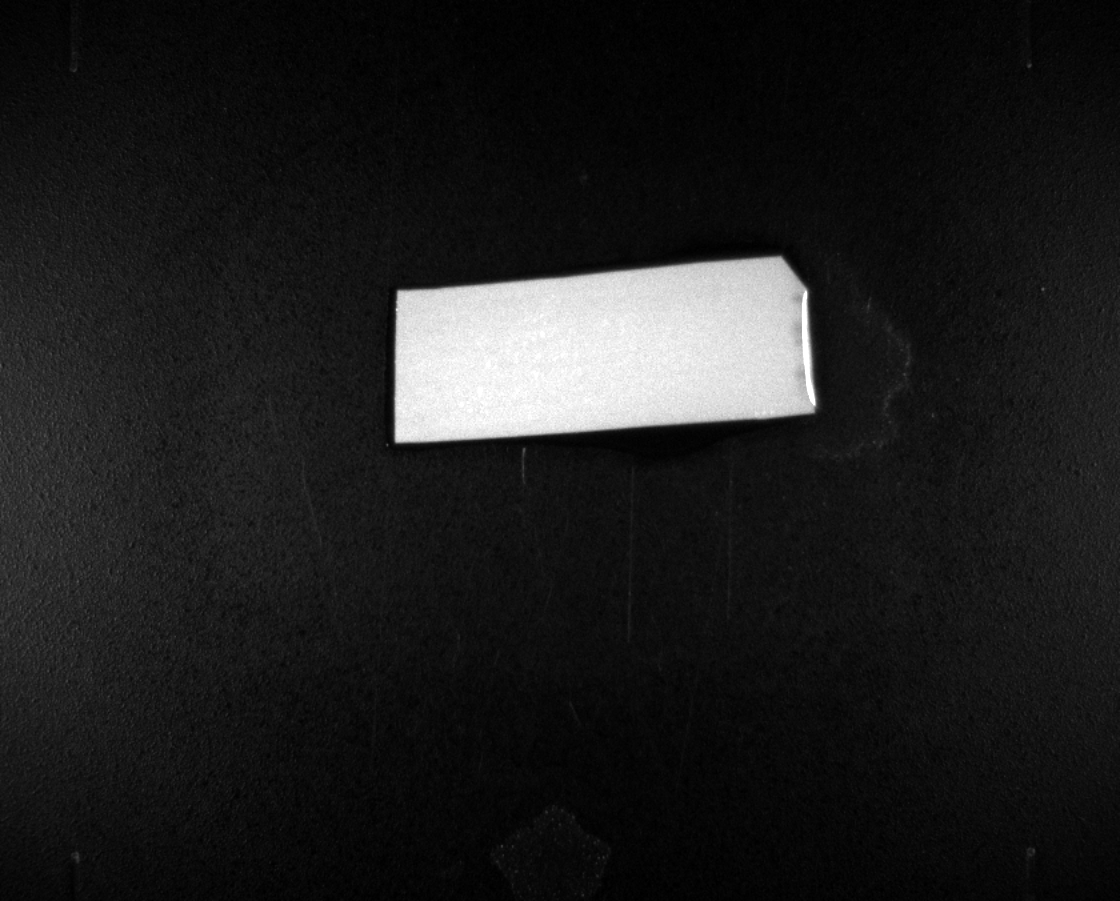

Supplement: Supplementary file 4 — Source Data [file 41467_2023_41520_MOESM4_ESM.zip › Source Data/Uncropped and Unprocessed Scans/Fig. 2c/IP HA; IB SUMO1 (Left 5 columns) - Marker.tif]

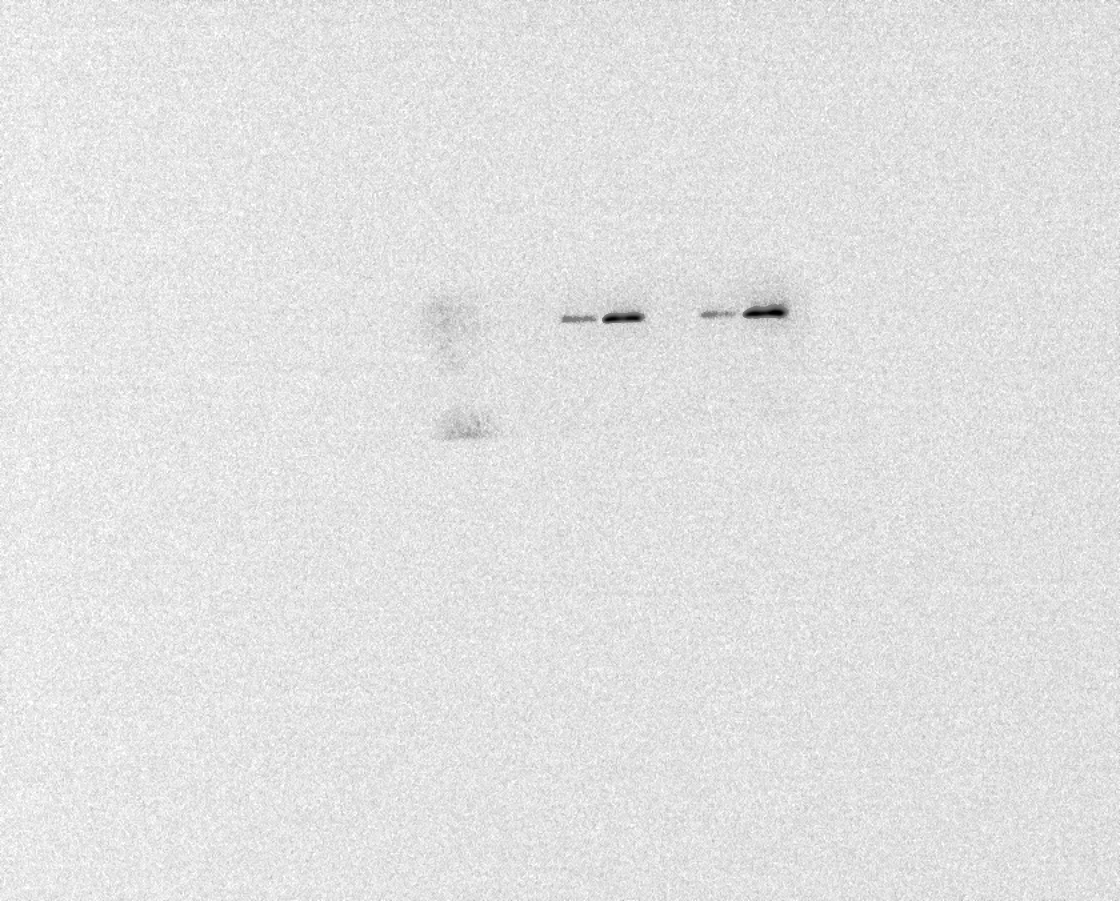

Supplement: Supplementary file 4 — Source Data [file 41467_2023_41520_MOESM4_ESM.zip › Source Data/Uncropped and Unprocessed Scans/Fig. 2c/IP HA; IB SUMO1 (Left 5 columns).tif]

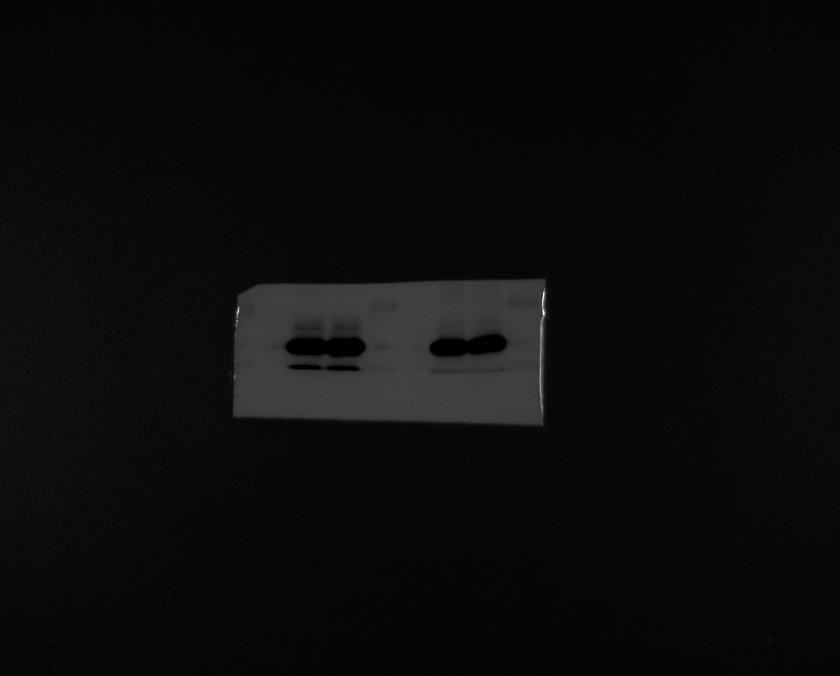

Supplement: Supplementary file 4 — Source Data [file 41467_2023_41520_MOESM4_ESM.zip › Source Data/Uncropped and Unprocessed Scans/Fig. 2d/(Left) IP HA; IB HA - Marker.tif]

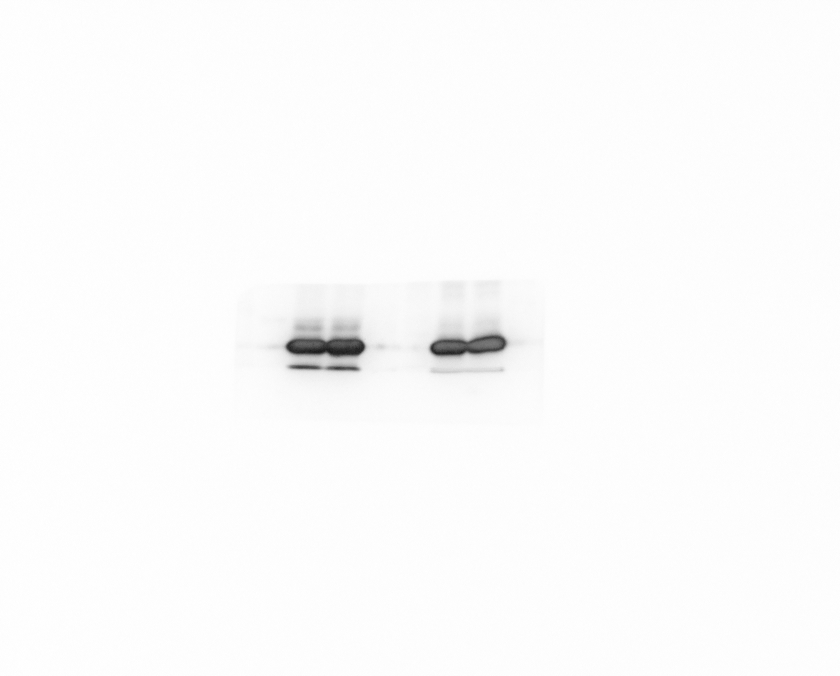

Supplement: Supplementary file 4 — Source Data [file 41467_2023_41520_MOESM4_ESM.zip › Source Data/Uncropped and Unprocessed Scans/Fig. 2d/(Left) IP HA; IB HA.tif]

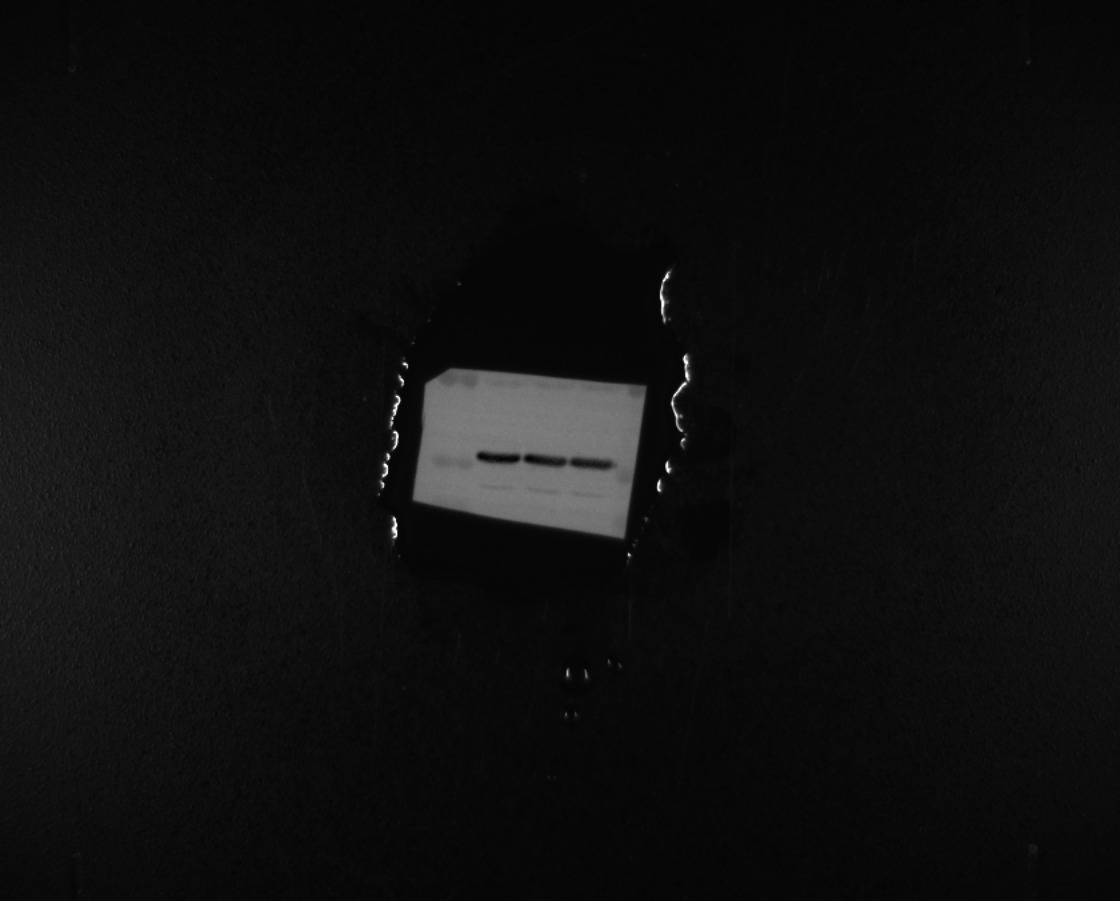

Supplement: Supplementary file 4 — Source Data [file 41467_2023_41520_MOESM4_ESM.zip › Source Data/Uncropped and Unprocessed Scans/Fig. 2d/IB GAPDH - Marker.tif]

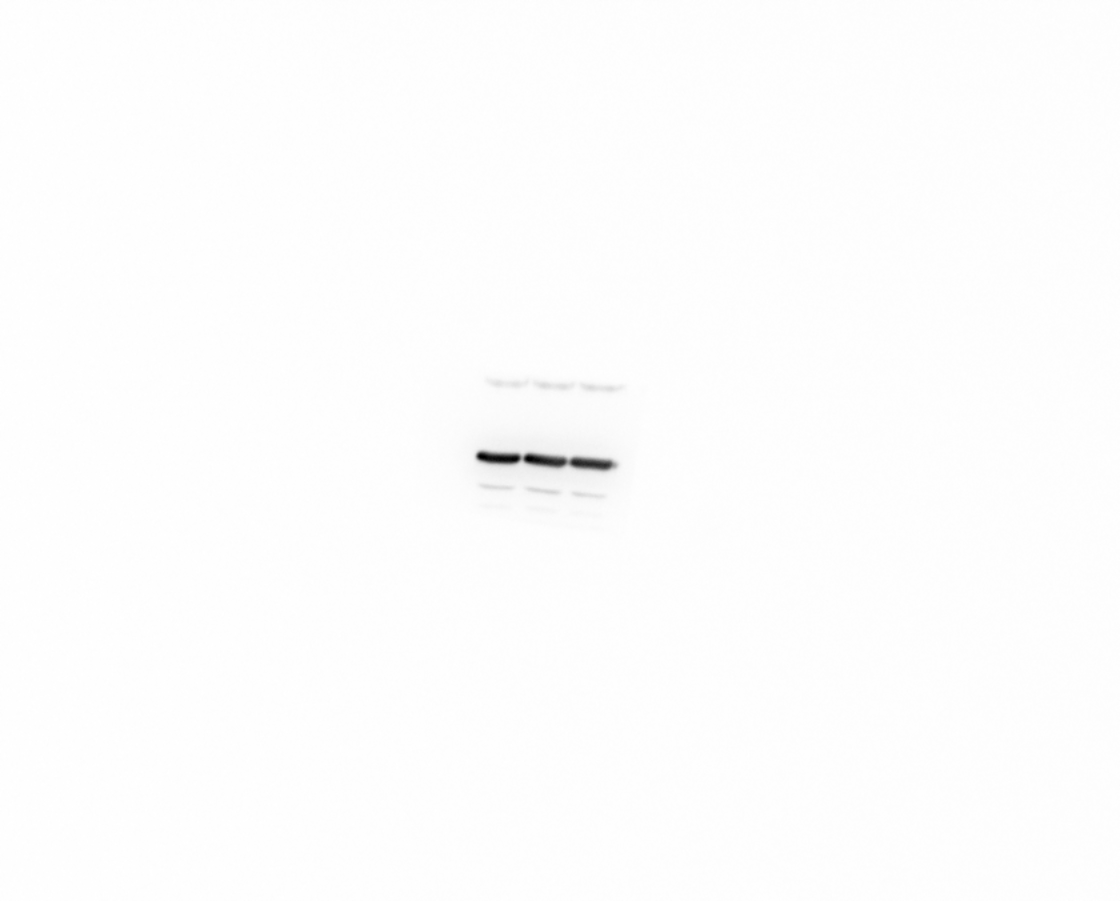

Supplement: Supplementary file 4 — Source Data [file 41467_2023_41520_MOESM4_ESM.zip › Source Data/Uncropped and Unprocessed Scans/Fig. 2d/IB GAPDH.tif]

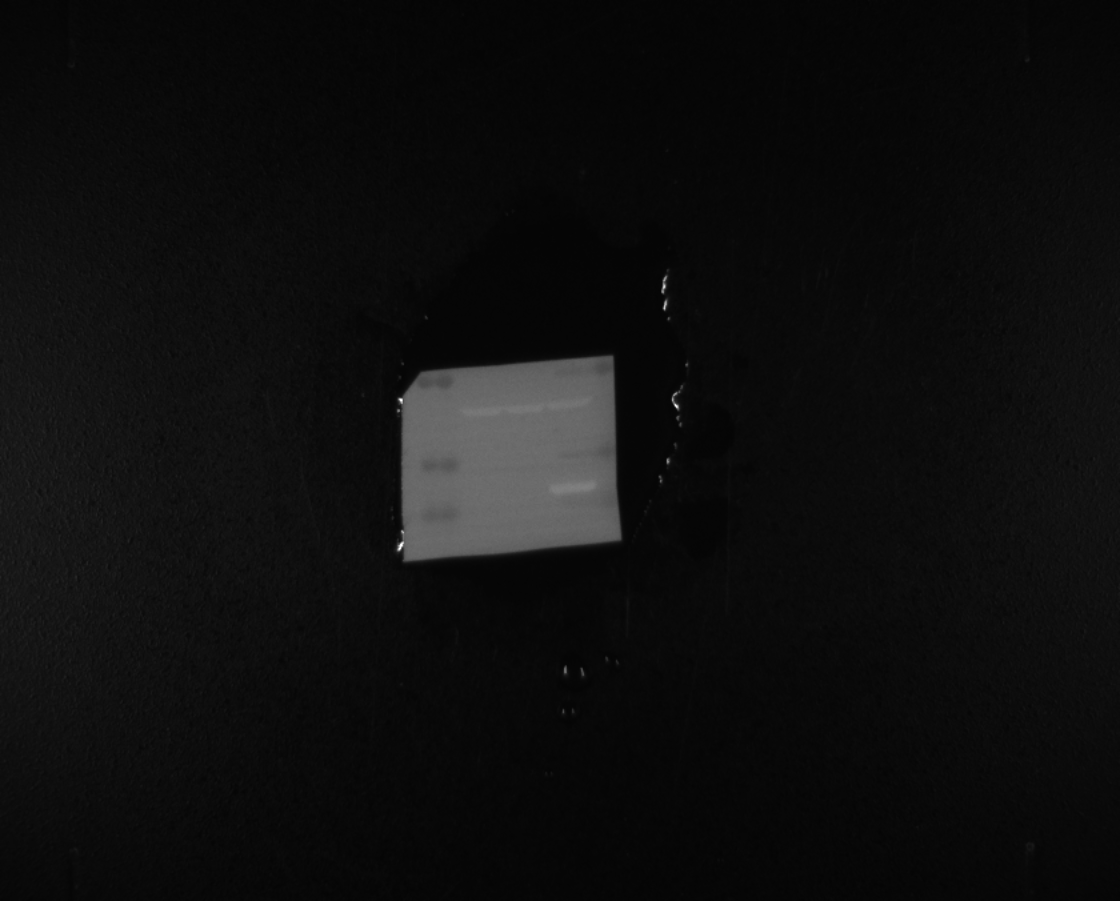

Supplement: Supplementary file 4 — Source Data [file 41467_2023_41520_MOESM4_ESM.zip › Source Data/Uncropped and Unprocessed Scans/Fig. 2d/IB Myc - Marker.tif]

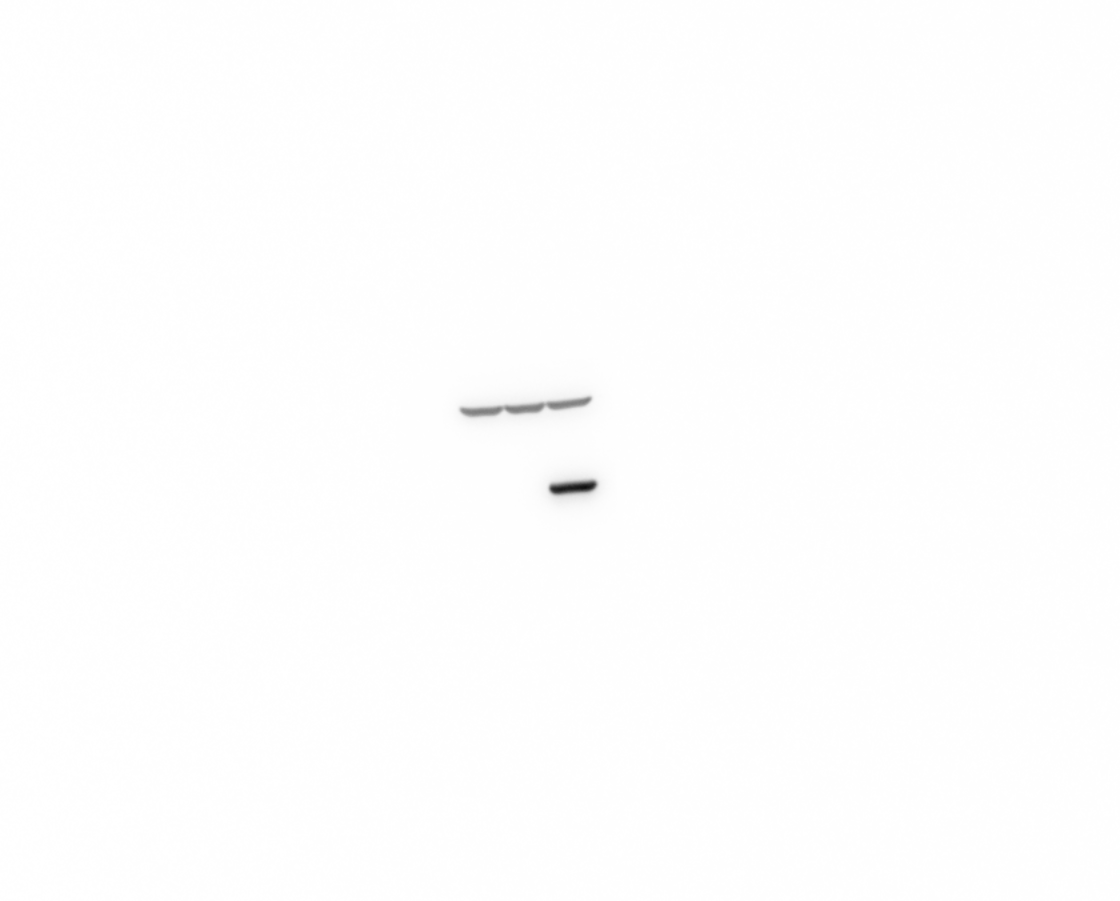

Supplement: Supplementary file 4 — Source Data [file 41467_2023_41520_MOESM4_ESM.zip › Source Data/Uncropped and Unprocessed Scans/Fig. 2d/IB Myc.tif]

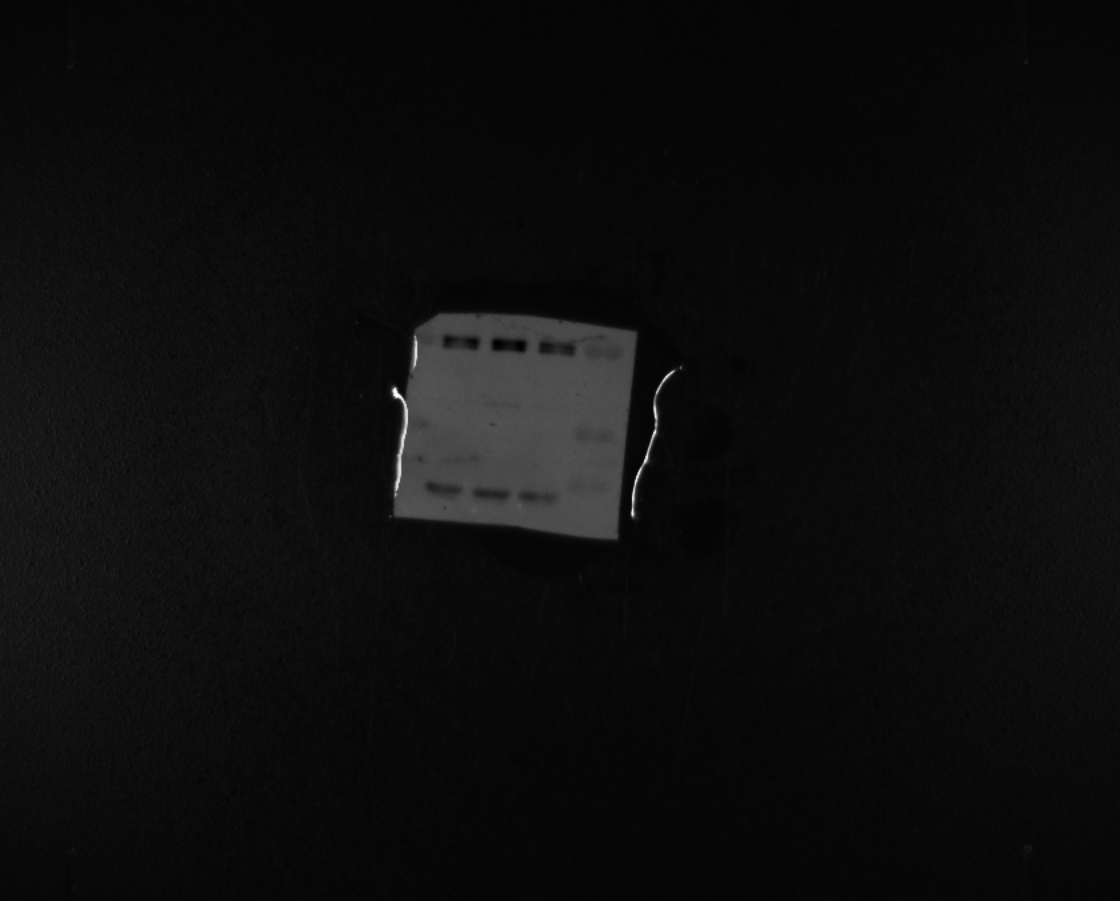

Supplement: Supplementary file 4 — Source Data [file 41467_2023_41520_MOESM4_ESM.zip › Source Data/Uncropped and Unprocessed Scans/Fig. 2d/IP HA; IB SUMO1 - Marker.tif]

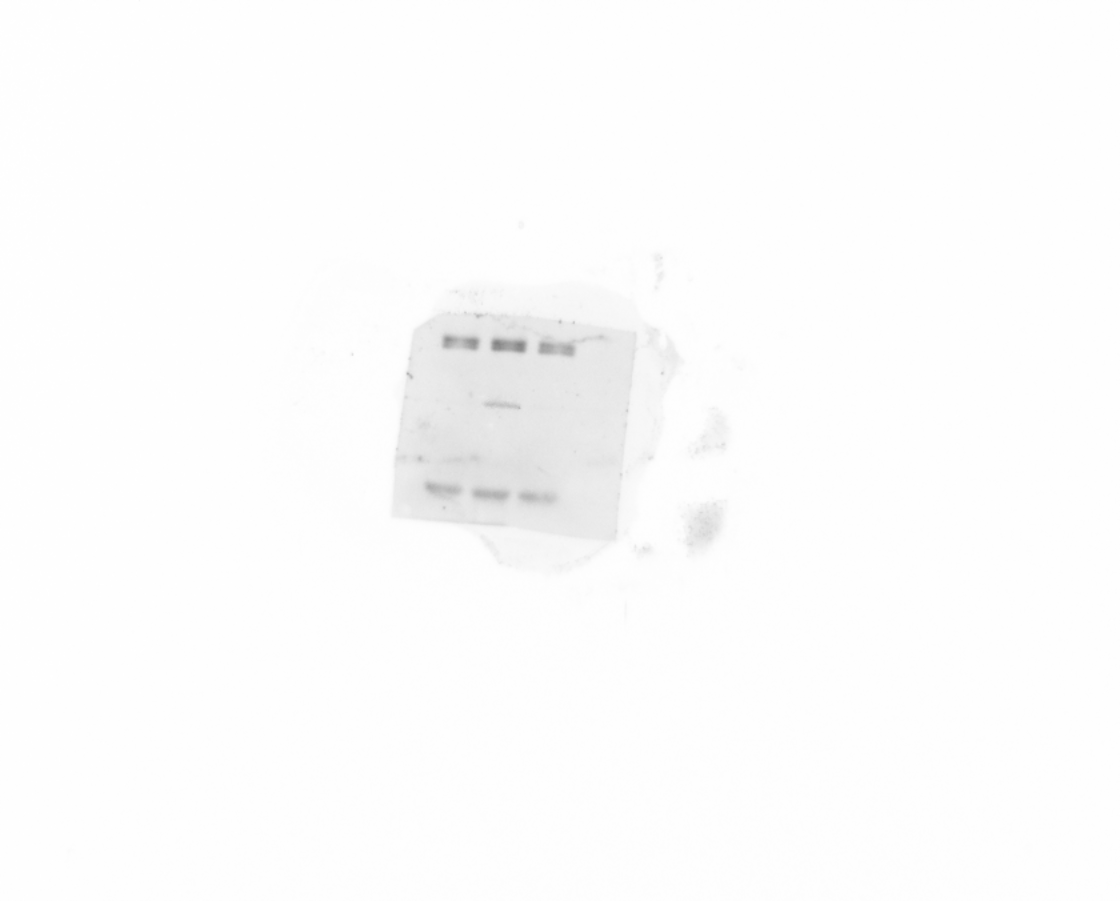

Supplement: Supplementary file 4 — Source Data [file 41467_2023_41520_MOESM4_ESM.zip › Source Data/Uncropped and Unprocessed Scans/Fig. 2d/IP HA; IB SUMO1.tif]

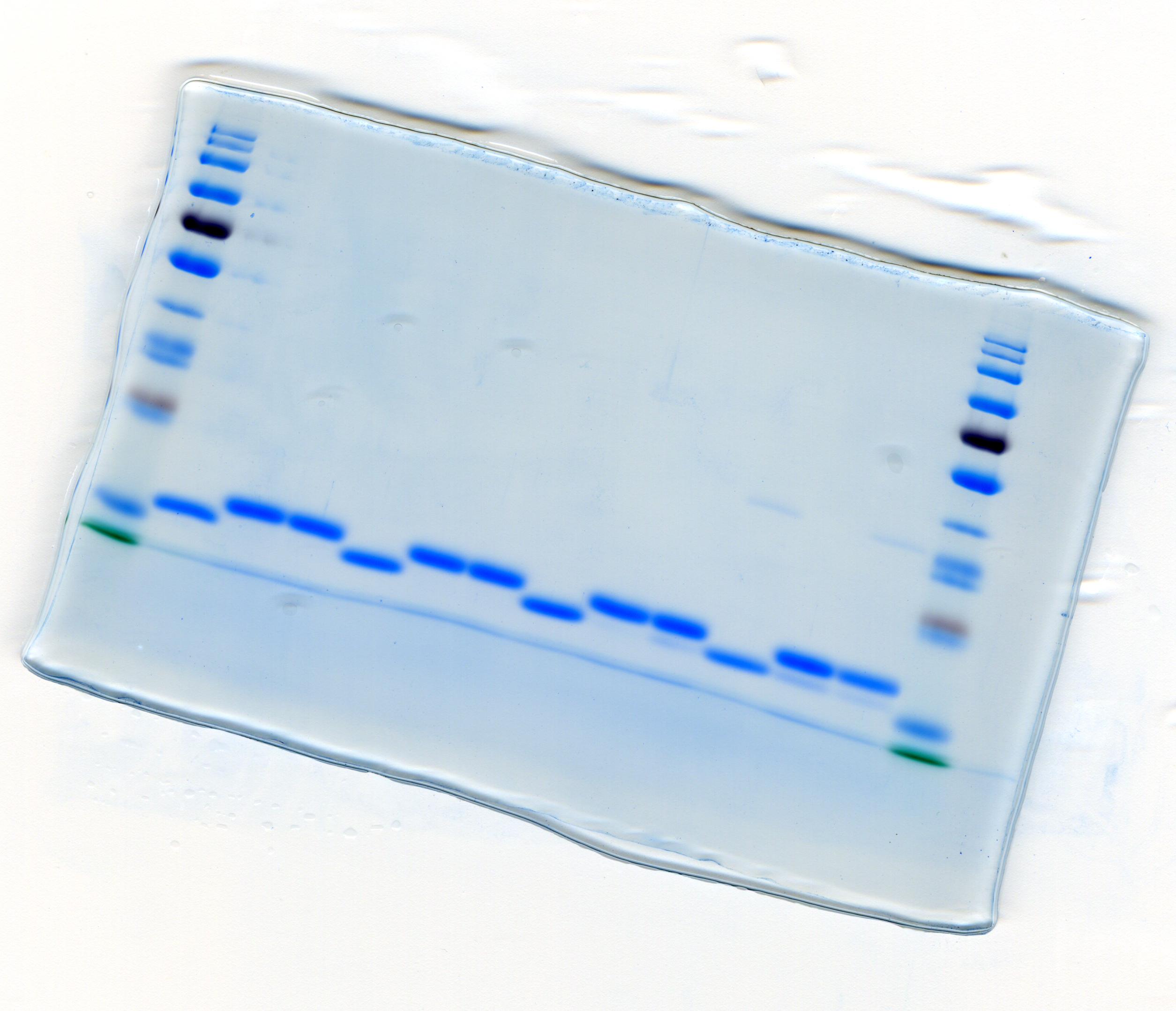

Supplement: Supplementary file 4 — Source Data [file 41467_2023_41520_MOESM4_ESM.zip › Source Data/Uncropped and Unprocessed Scans/Fig. 2f/CBB.tif]

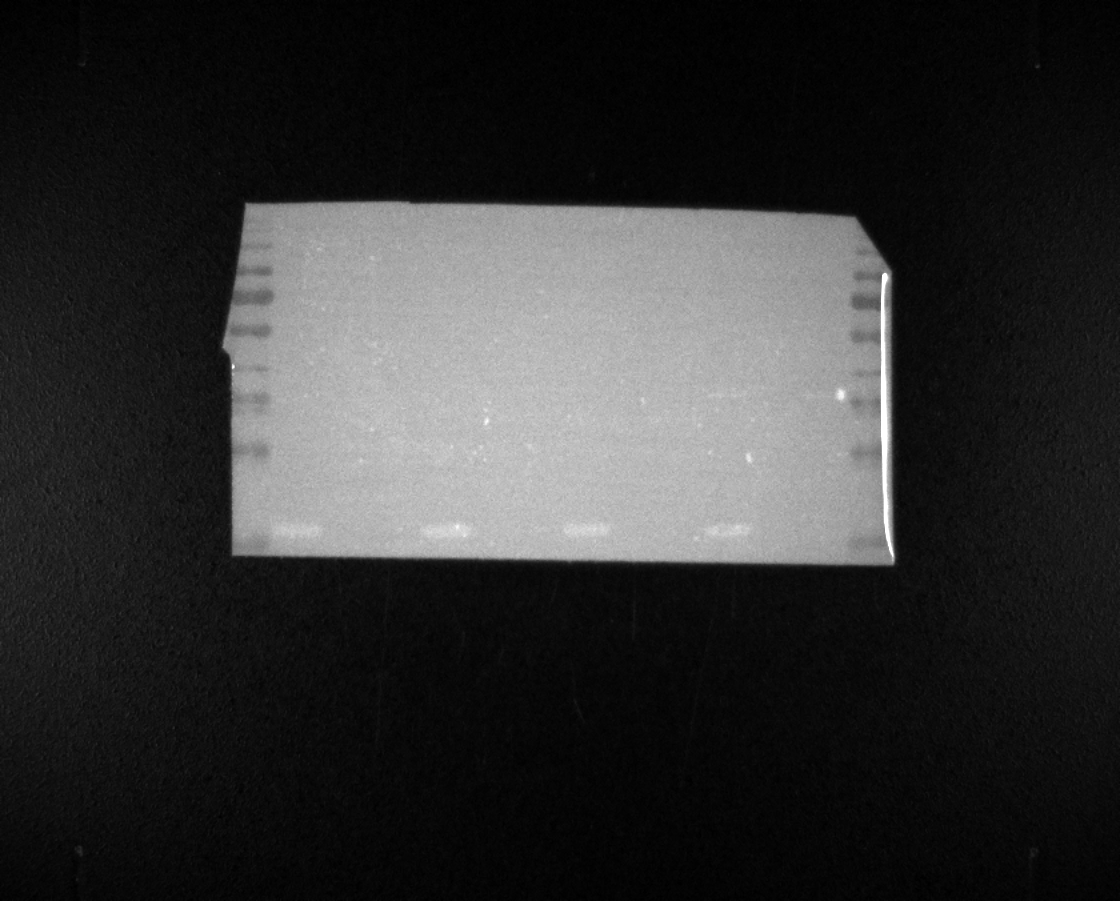

Supplement: Supplementary file 4 — Source Data [file 41467_2023_41520_MOESM4_ESM.zip › Source Data/Uncropped and Unprocessed Scans/Fig. 2f/IB CFL1 - Marker.tif]

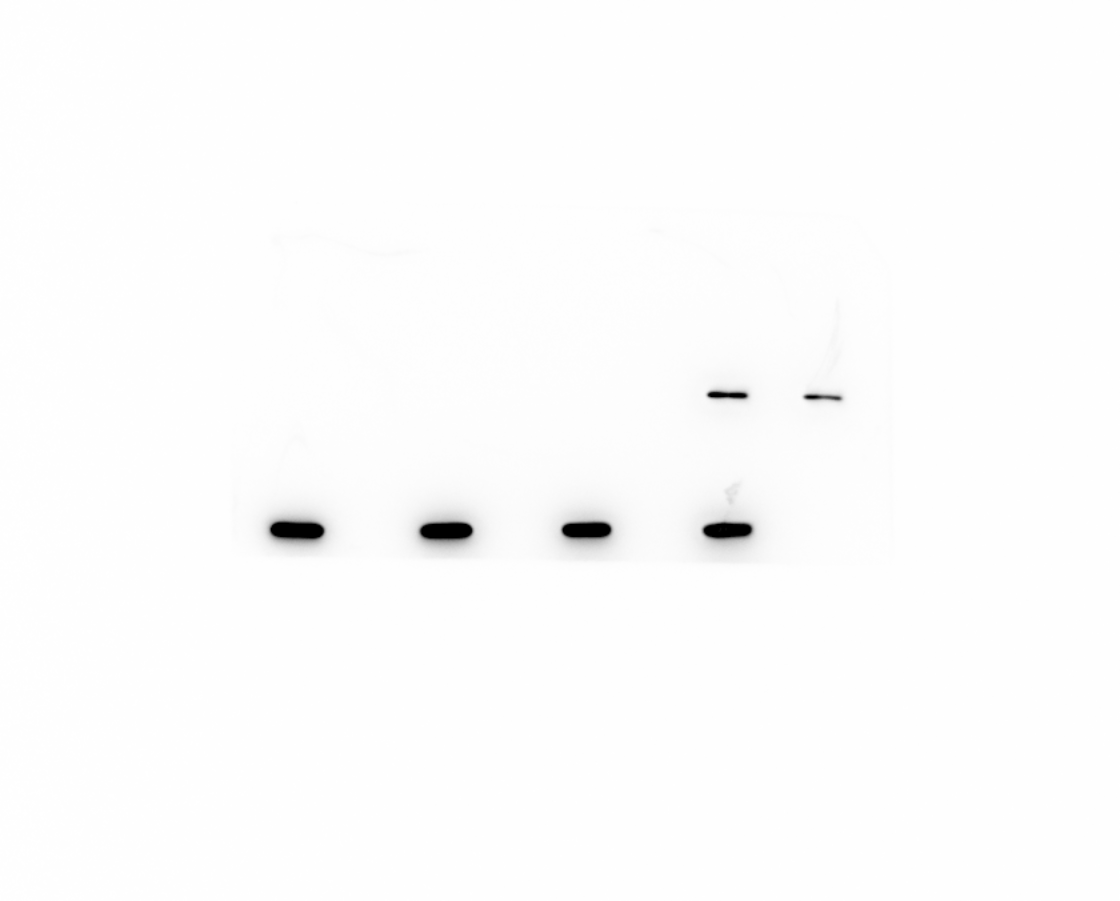

Supplement: Supplementary file 4 — Source Data [file 41467_2023_41520_MOESM4_ESM.zip › Source Data/Uncropped and Unprocessed Scans/Fig. 2f/IB CFL1.tif]

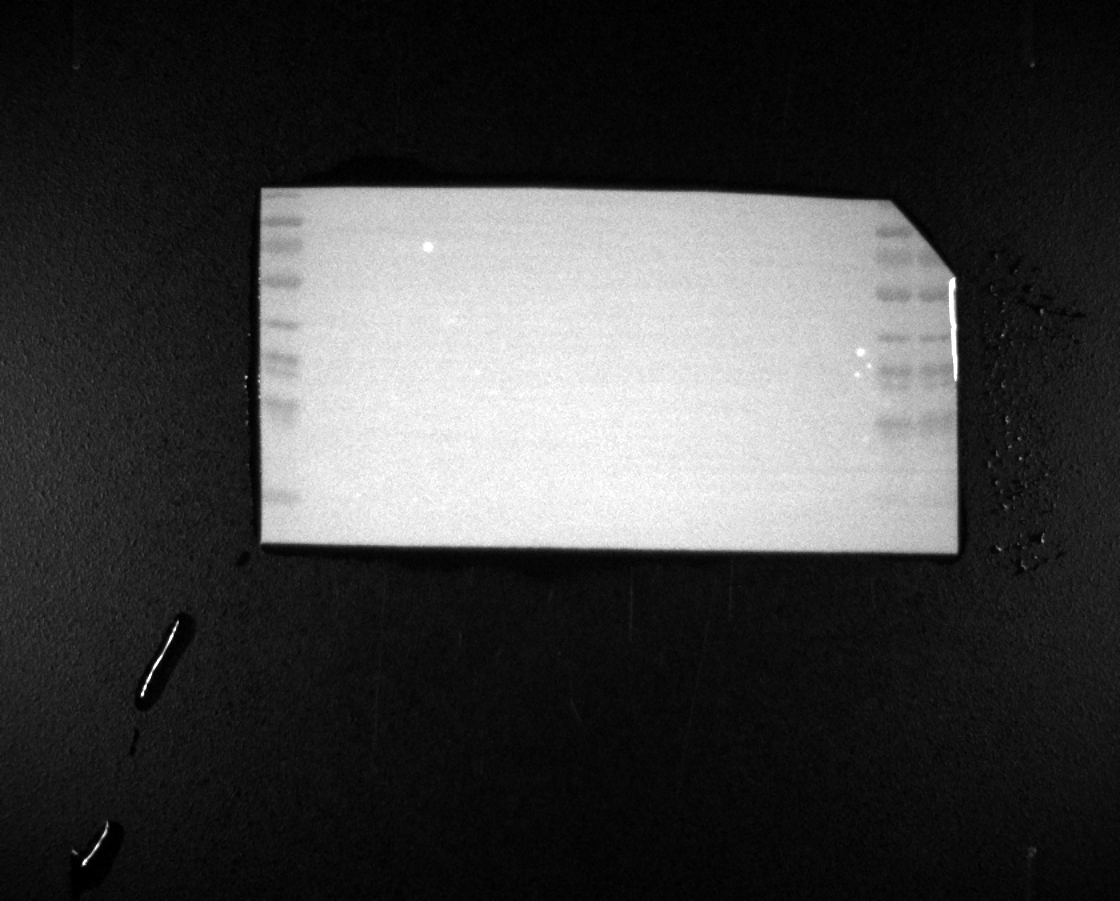

Supplement: Supplementary file 4 — Source Data [file 41467_2023_41520_MOESM4_ESM.zip › Source Data/Uncropped and Unprocessed Scans/Fig. 2f/IB SUMO1 - Marker.tif]

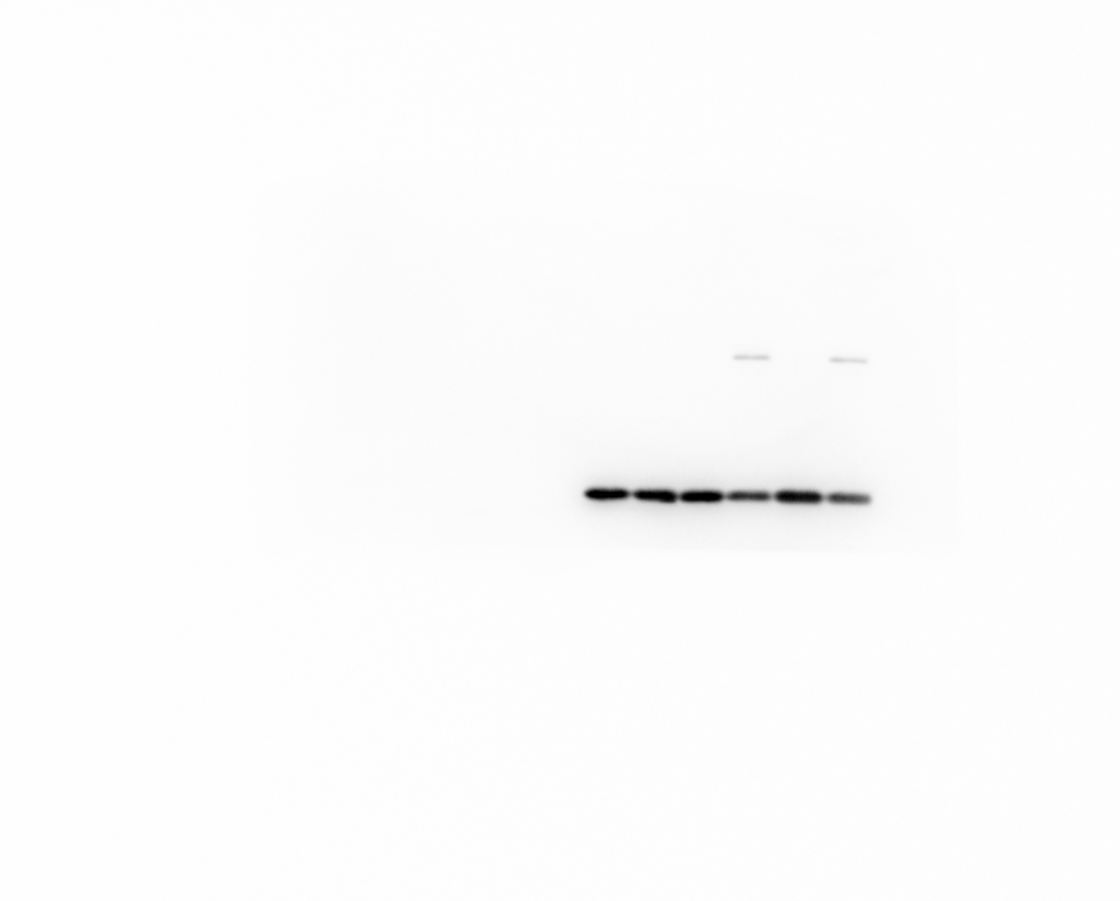

Supplement: Supplementary file 4 — Source Data [file 41467_2023_41520_MOESM4_ESM.zip › Source Data/Uncropped and Unprocessed Scans/Fig. 2f/IB SUMO1.tif]

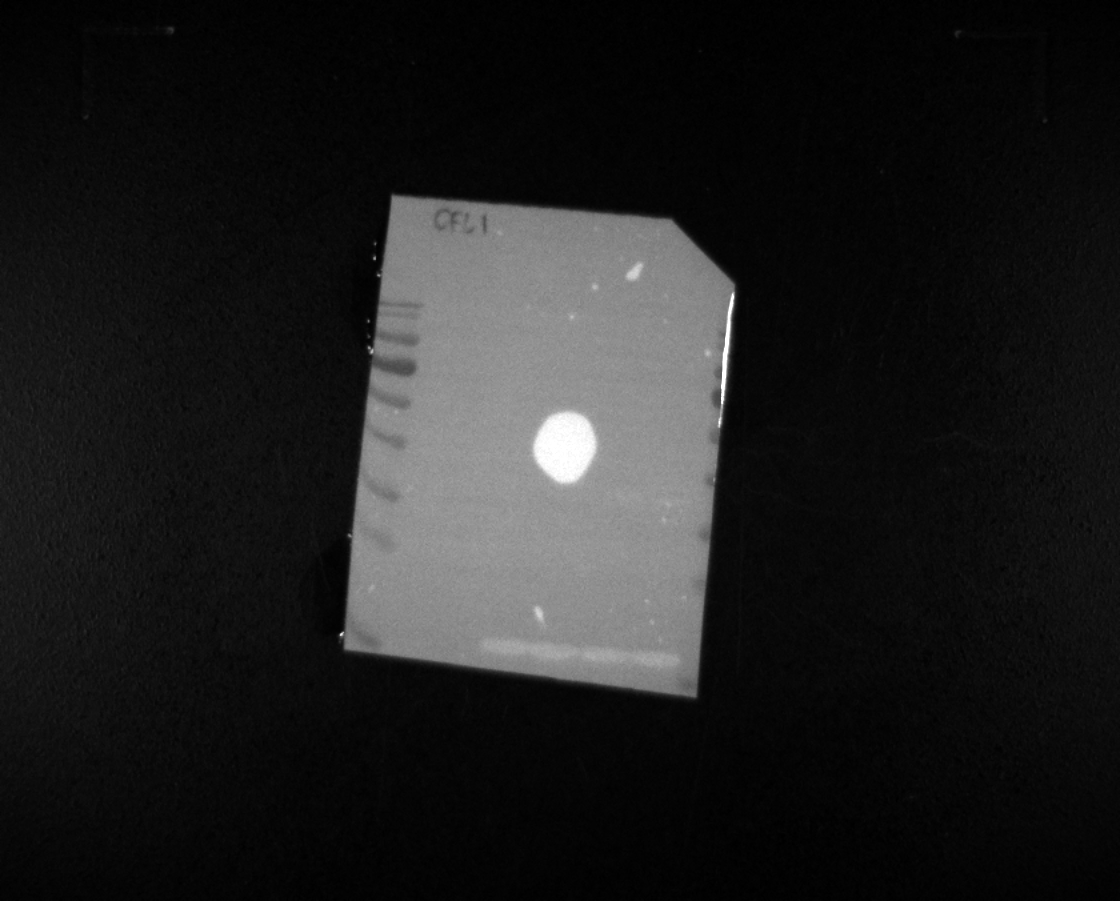

Supplement: Supplementary file 4 — Source Data [file 41467_2023_41520_MOESM4_ESM.zip › Source Data/Uncropped and Unprocessed Scans/Fig. 2g/IB CFL1 - Marker.tif]

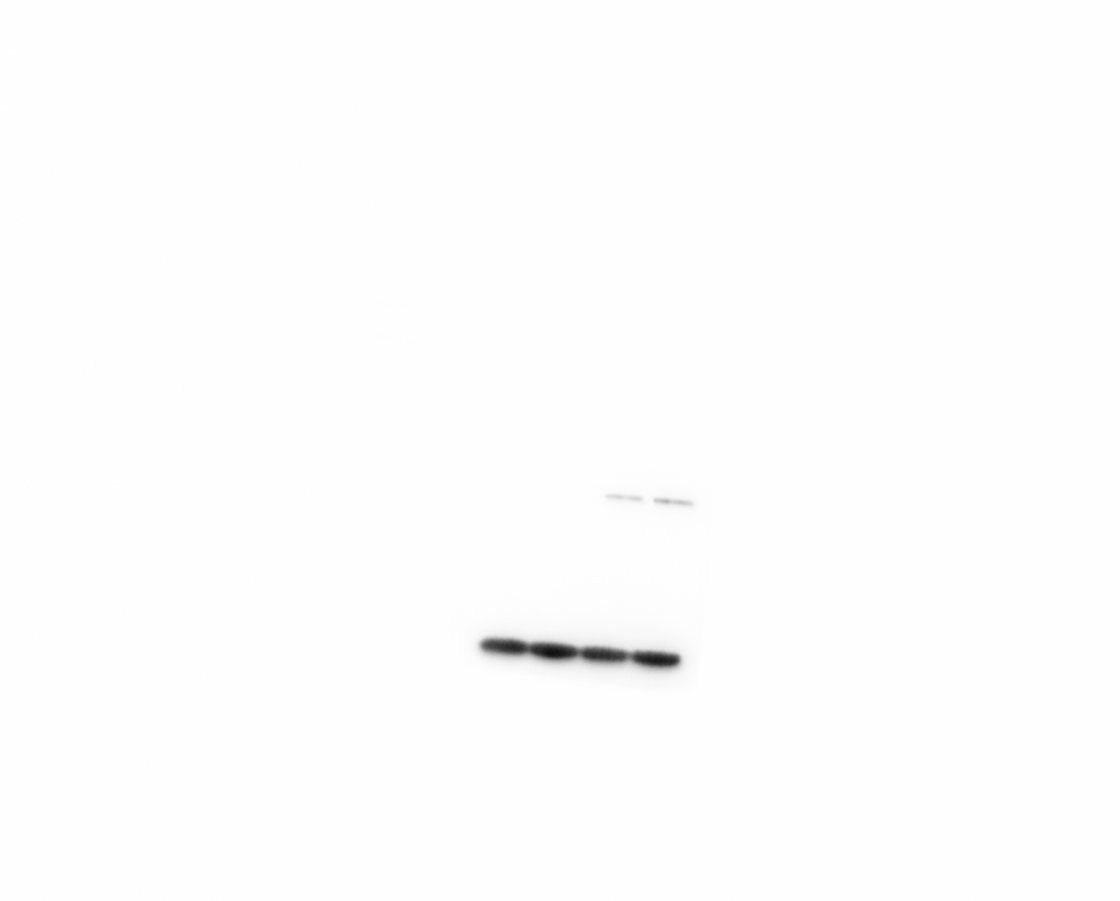

Supplement: Supplementary file 4 — Source Data [file 41467_2023_41520_MOESM4_ESM.zip › Source Data/Uncropped and Unprocessed Scans/Fig. 2g/IB CFL1.tif]

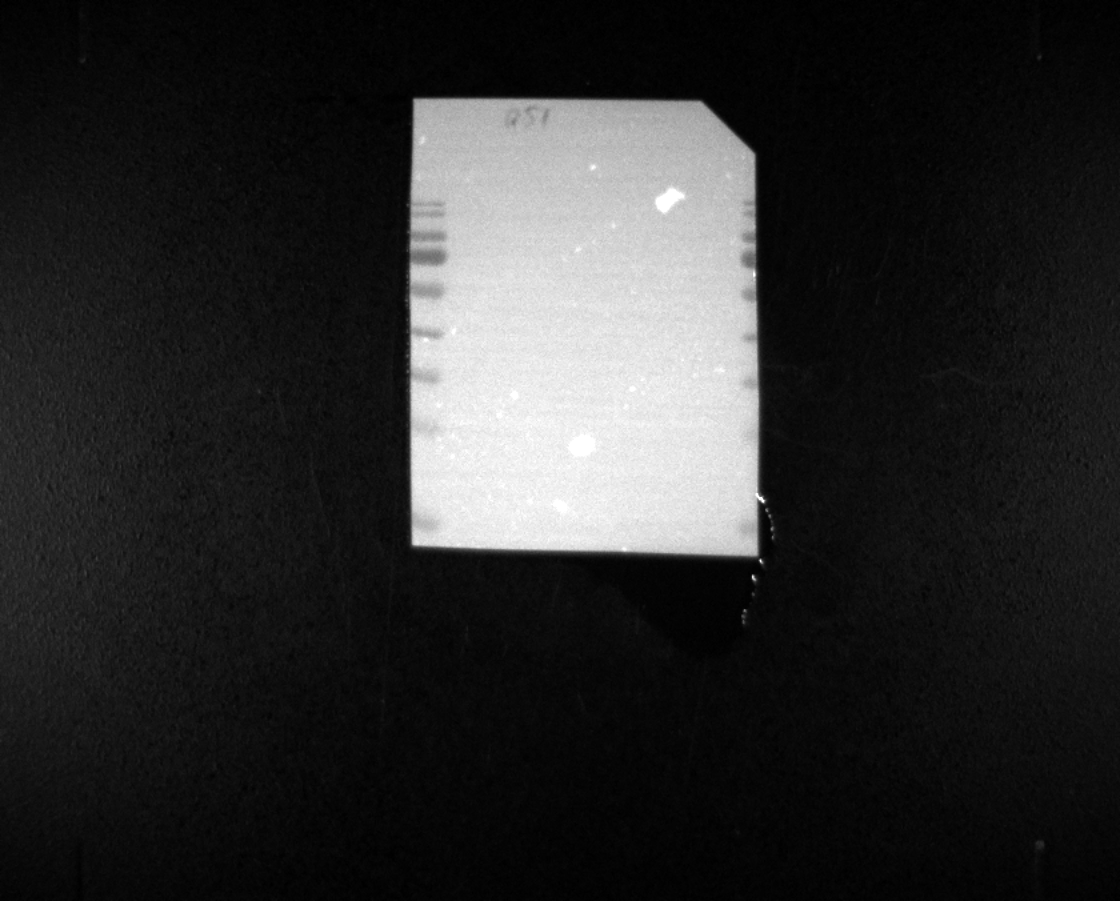

Supplement: Supplementary file 4 — Source Data [file 41467_2023_41520_MOESM4_ESM.zip › Source Data/Uncropped and Unprocessed Scans/Fig. 2g/IB GST - Marker.tif]

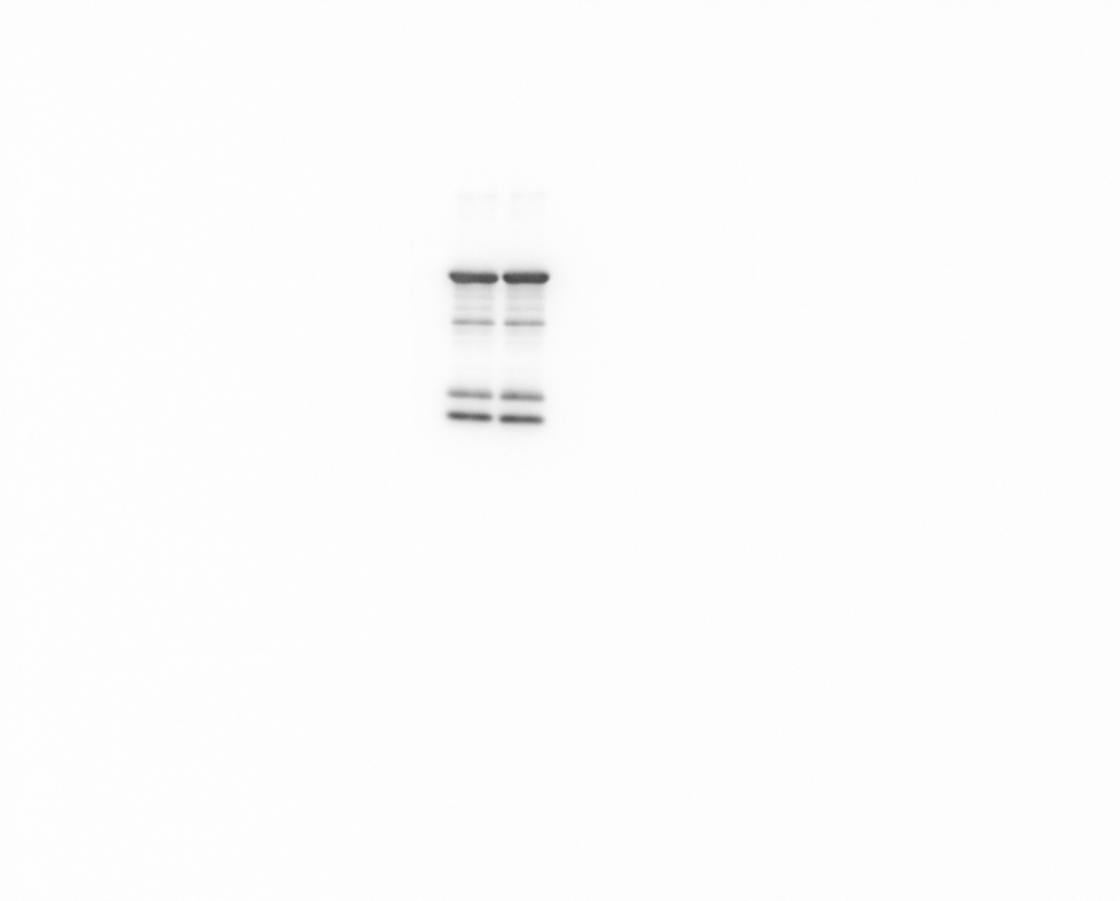

Supplement: Supplementary file 4 — Source Data [file 41467_2023_41520_MOESM4_ESM.zip › Source Data/Uncropped and Unprocessed Scans/Fig. 2g/IB GST.tif]

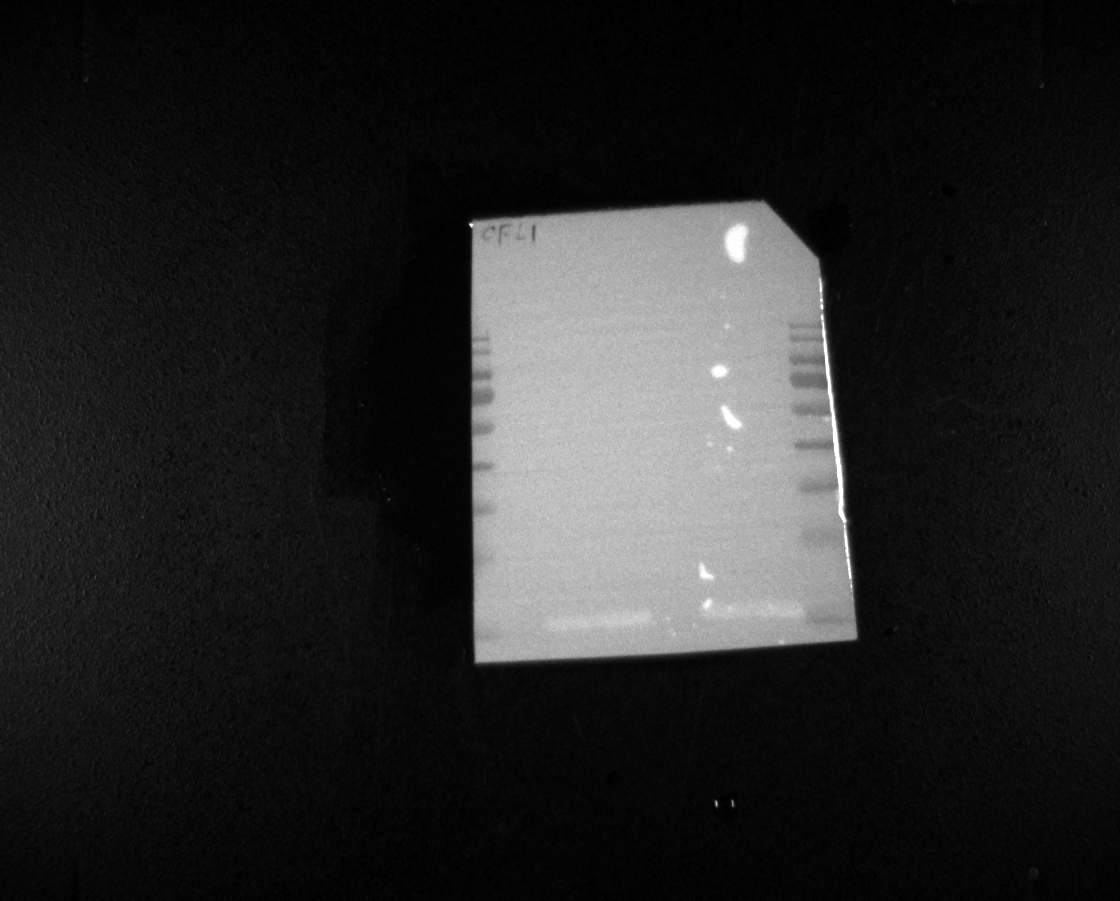

Supplement: Supplementary file 4 — Source Data [file 41467_2023_41520_MOESM4_ESM.zip › Source Data/Uncropped and Unprocessed Scans/Fig. 2g/IB SUMO1 - Marker.tif]

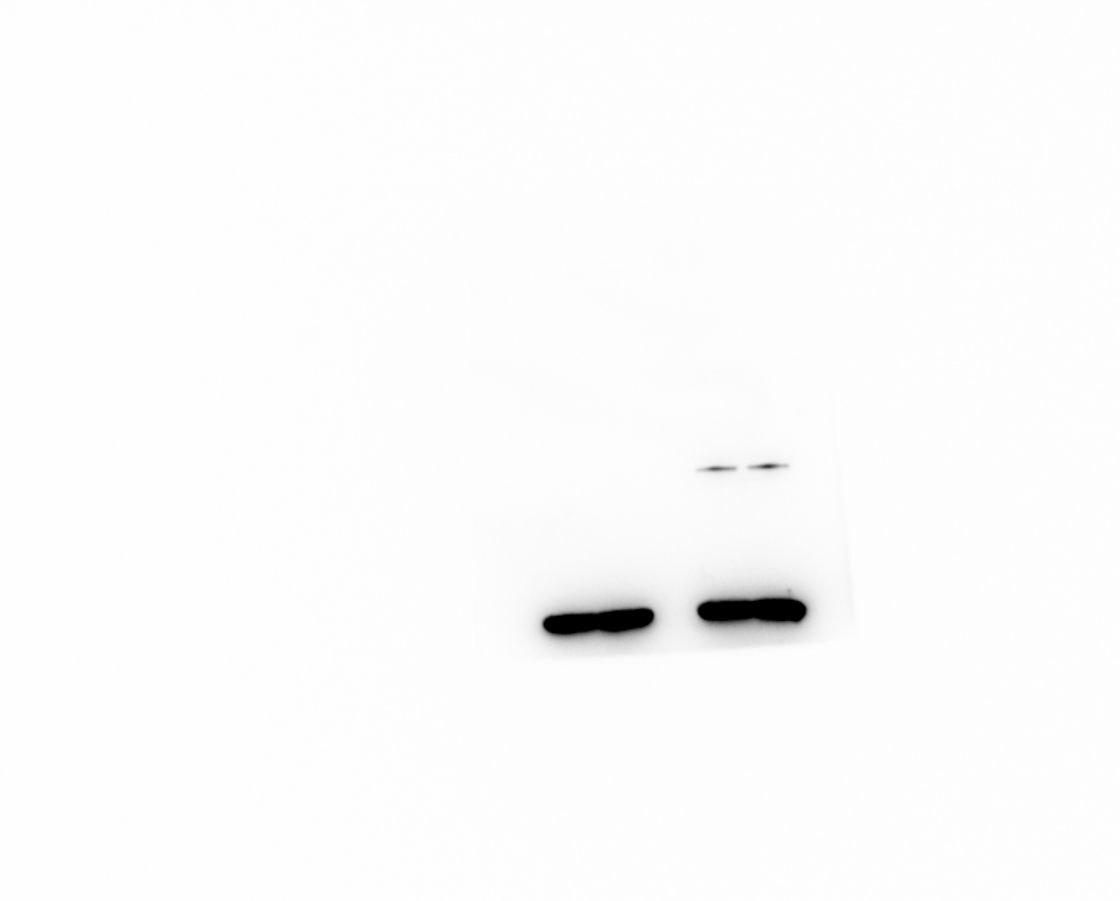

Supplement: Supplementary file 4 — Source Data [file 41467_2023_41520_MOESM4_ESM.zip › Source Data/Uncropped and Unprocessed Scans/Fig. 2g/IB SUMO1.tif]

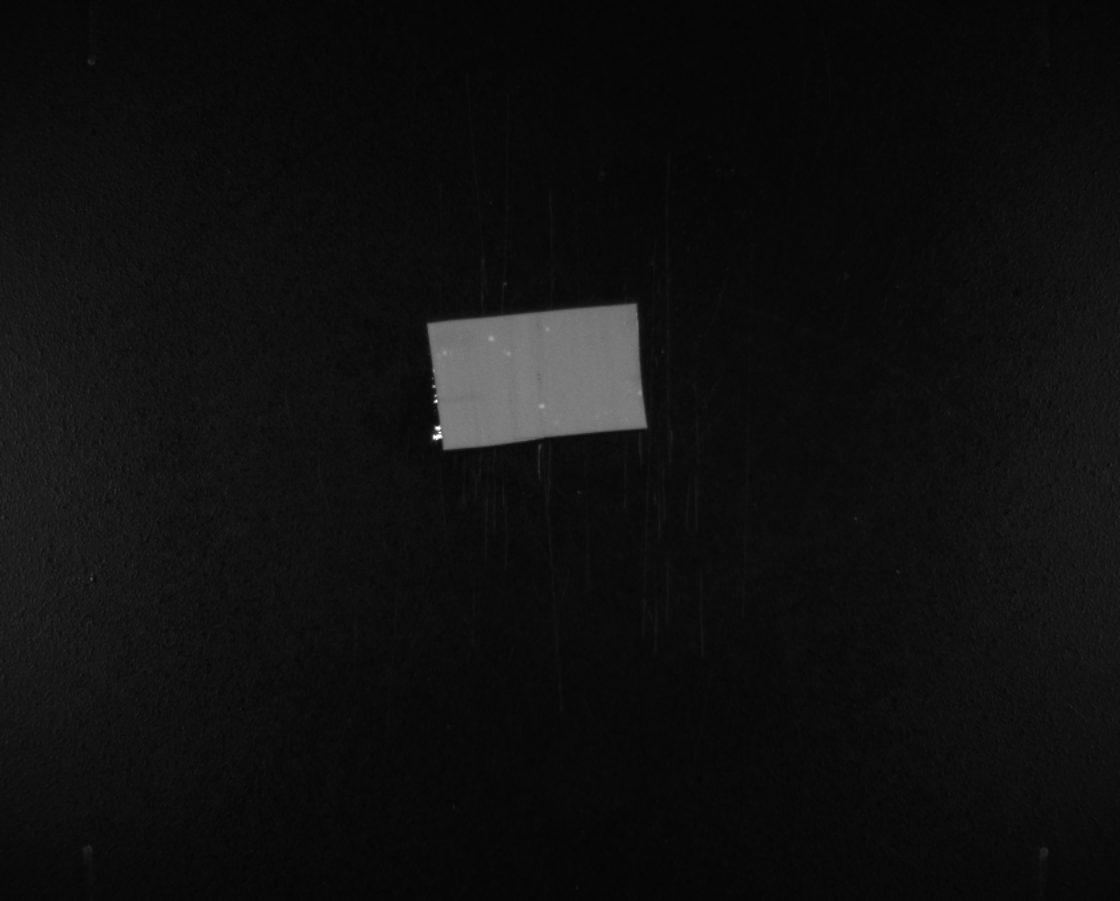

Supplement: Supplementary file 4 — Source Data [file 41467_2023_41520_MOESM4_ESM.zip › Source Data/Uncropped and Unprocessed Scans/Fig. 3a/IB CFL1 (rabbit) - Marker.tif]

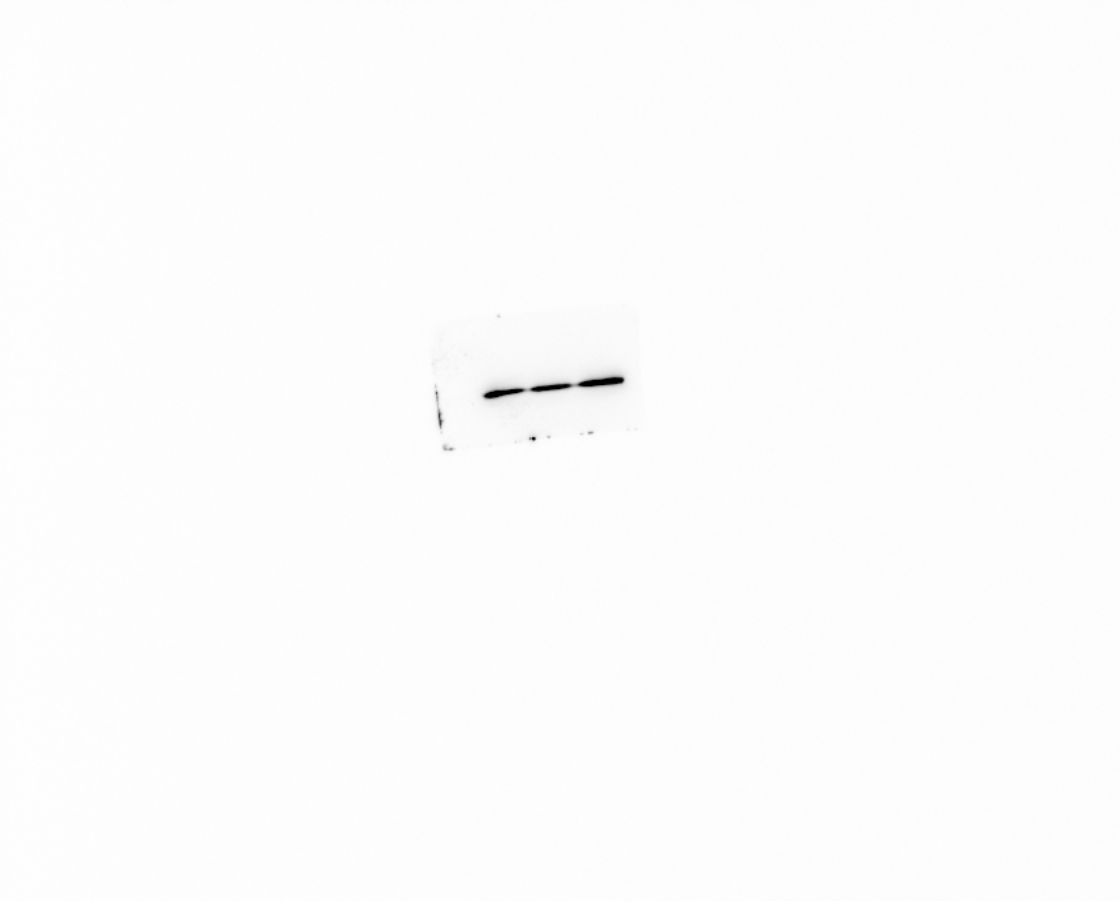

Supplement: Supplementary file 4 — Source Data [file 41467_2023_41520_MOESM4_ESM.zip › Source Data/Uncropped and Unprocessed Scans/Fig. 3a/IB CFL1 (rabbit).tif]

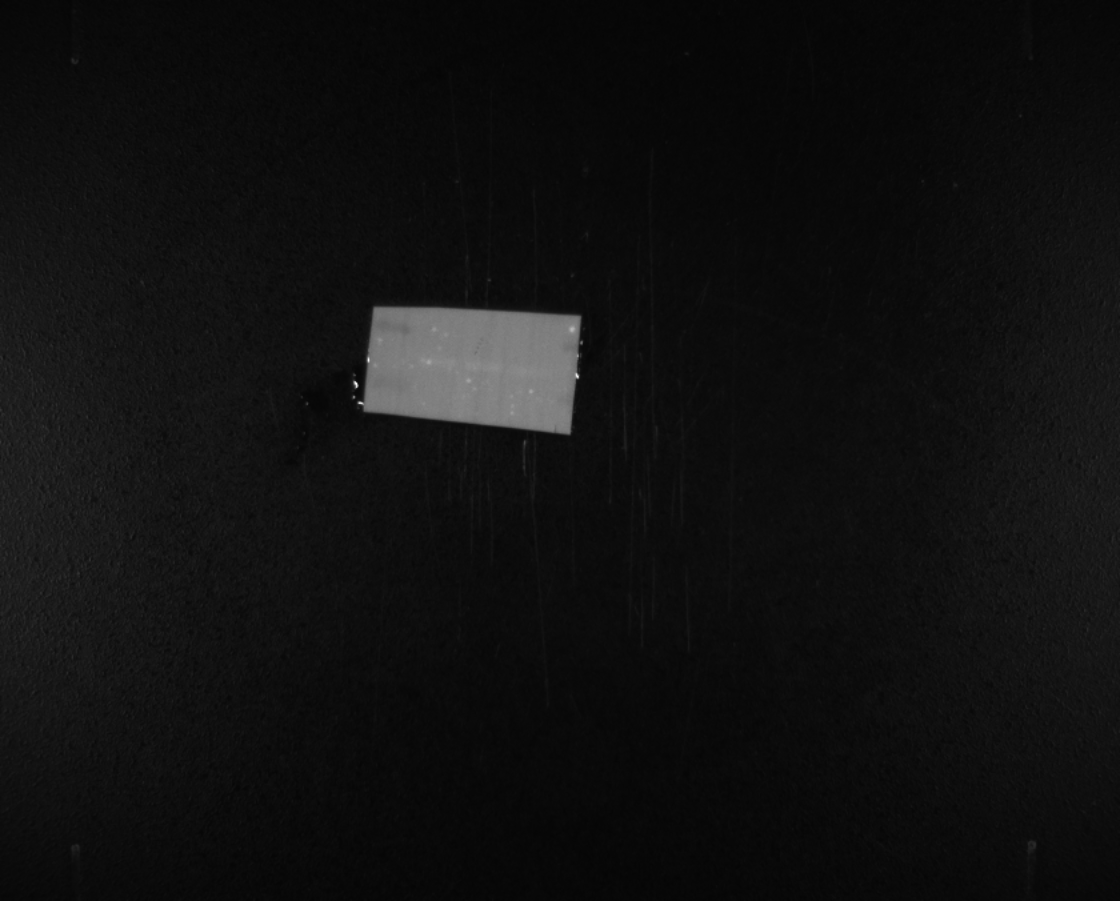

Supplement: Supplementary file 4 — Source Data [file 41467_2023_41520_MOESM4_ESM.zip › Source Data/Uncropped and Unprocessed Scans/Fig. 3a/IB GAPDH - Marker.tif]

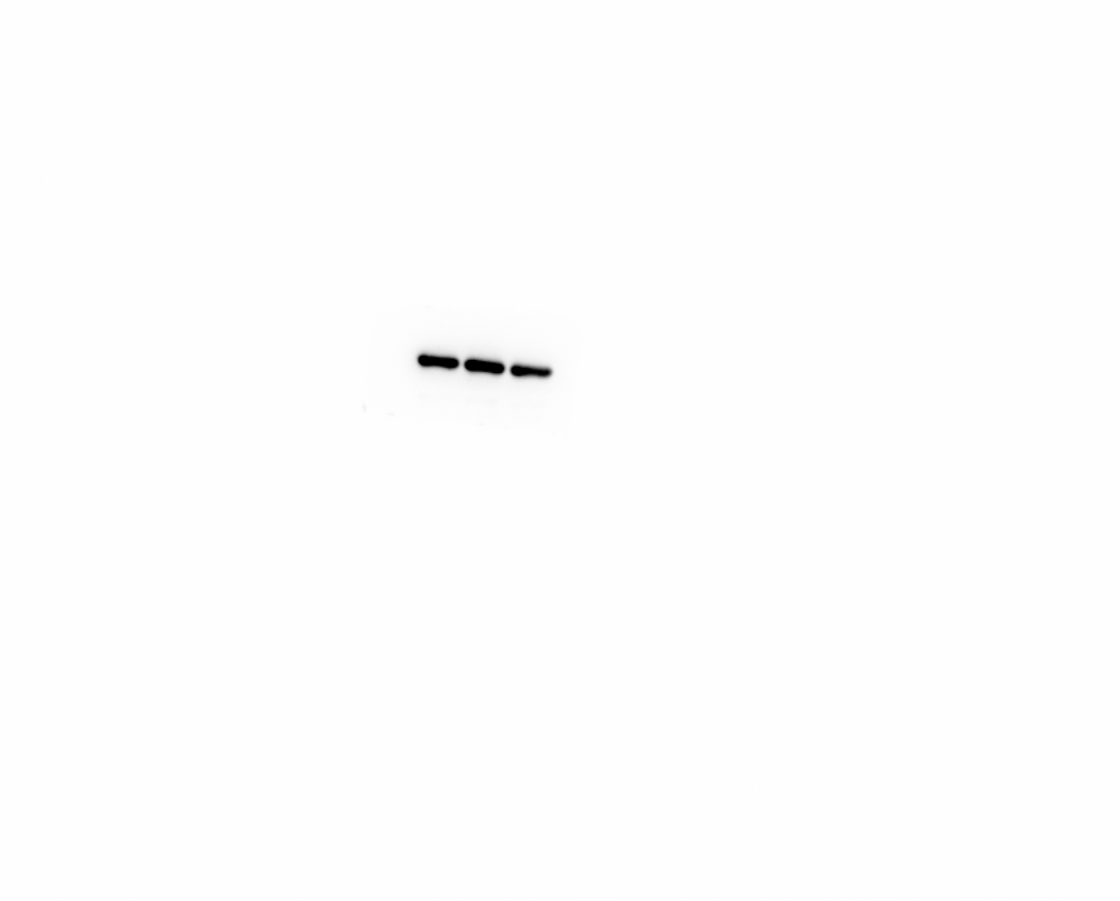

Supplement: Supplementary file 4 — Source Data [file 41467_2023_41520_MOESM4_ESM.zip › Source Data/Uncropped and Unprocessed Scans/Fig. 3a/IB GAPDH.tif]

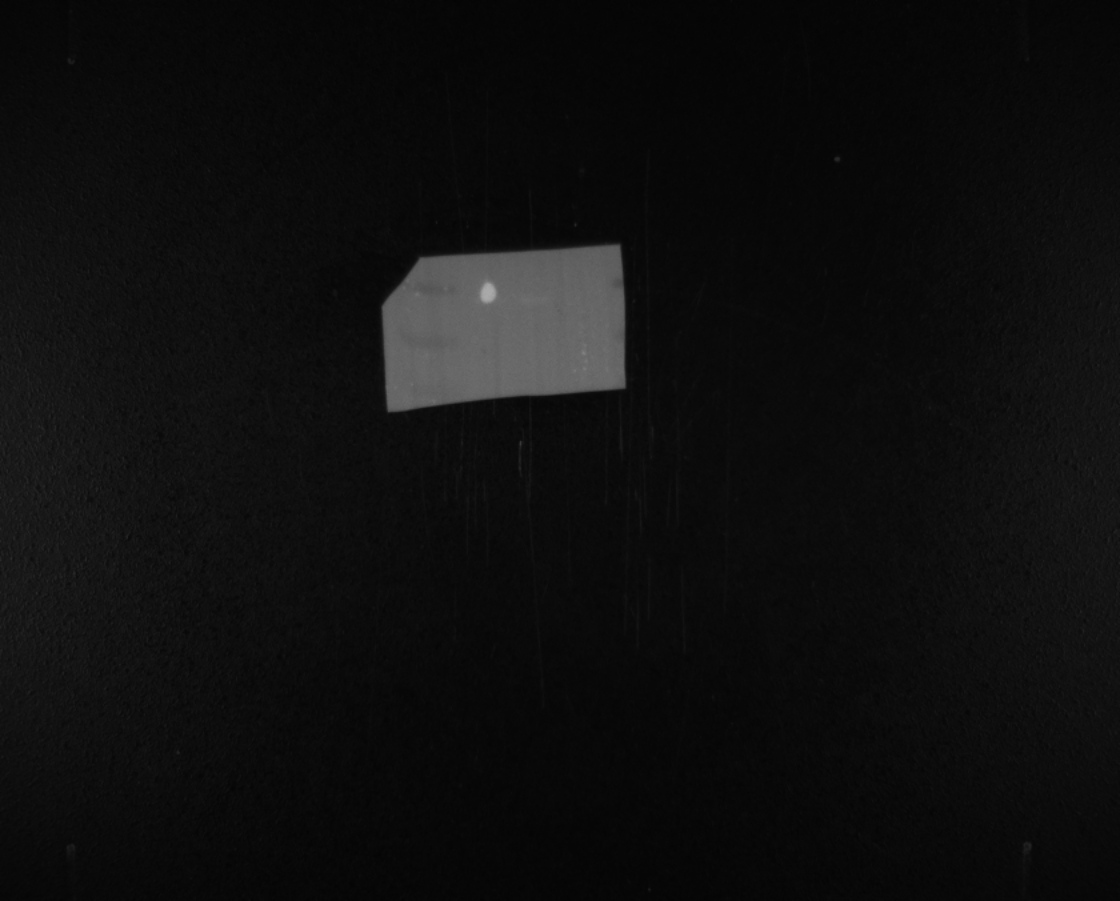

Supplement: Supplementary file 4 — Source Data [file 41467_2023_41520_MOESM4_ESM.zip › Source Data/Uncropped and Unprocessed Scans/Fig. 3a/IB SAE1 - Marker.tif]

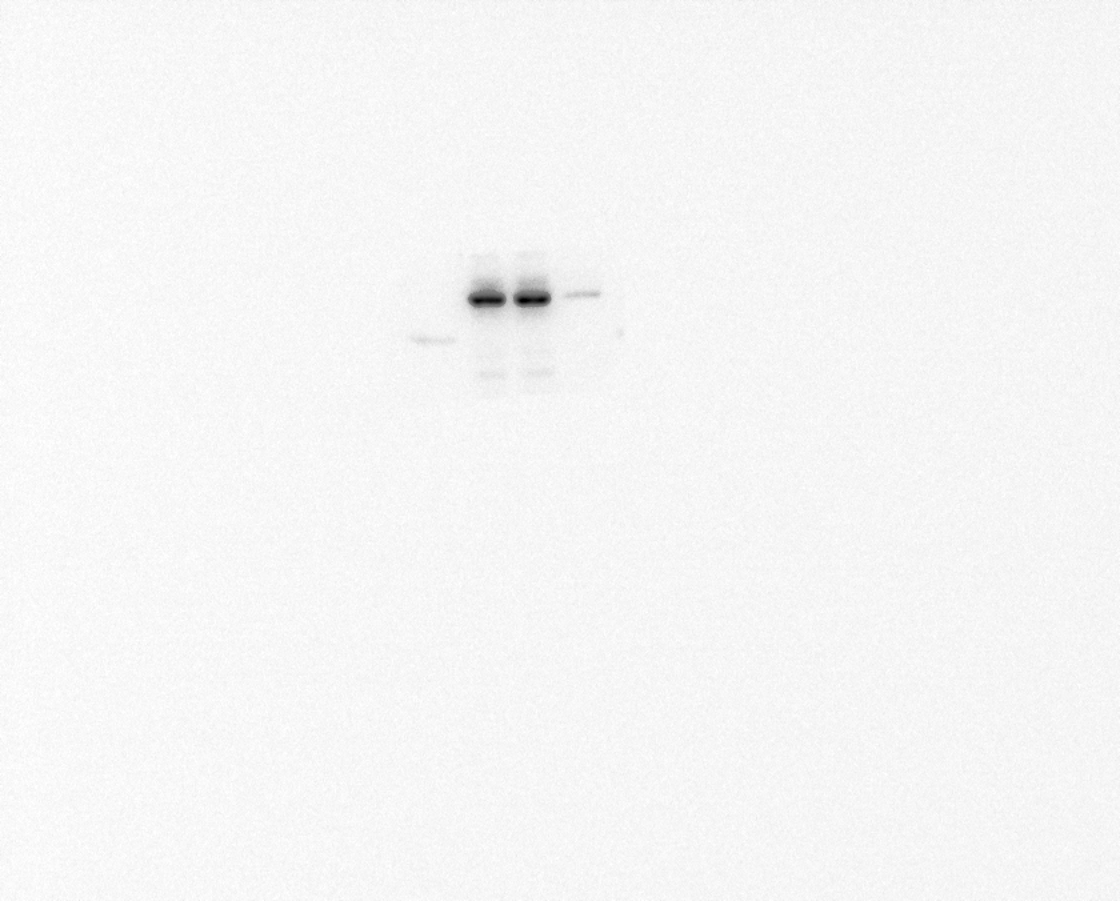

Supplement: Supplementary file 4 — Source Data [file 41467_2023_41520_MOESM4_ESM.zip › Source Data/Uncropped and Unprocessed Scans/Fig. 3a/IB SAE1.tif]

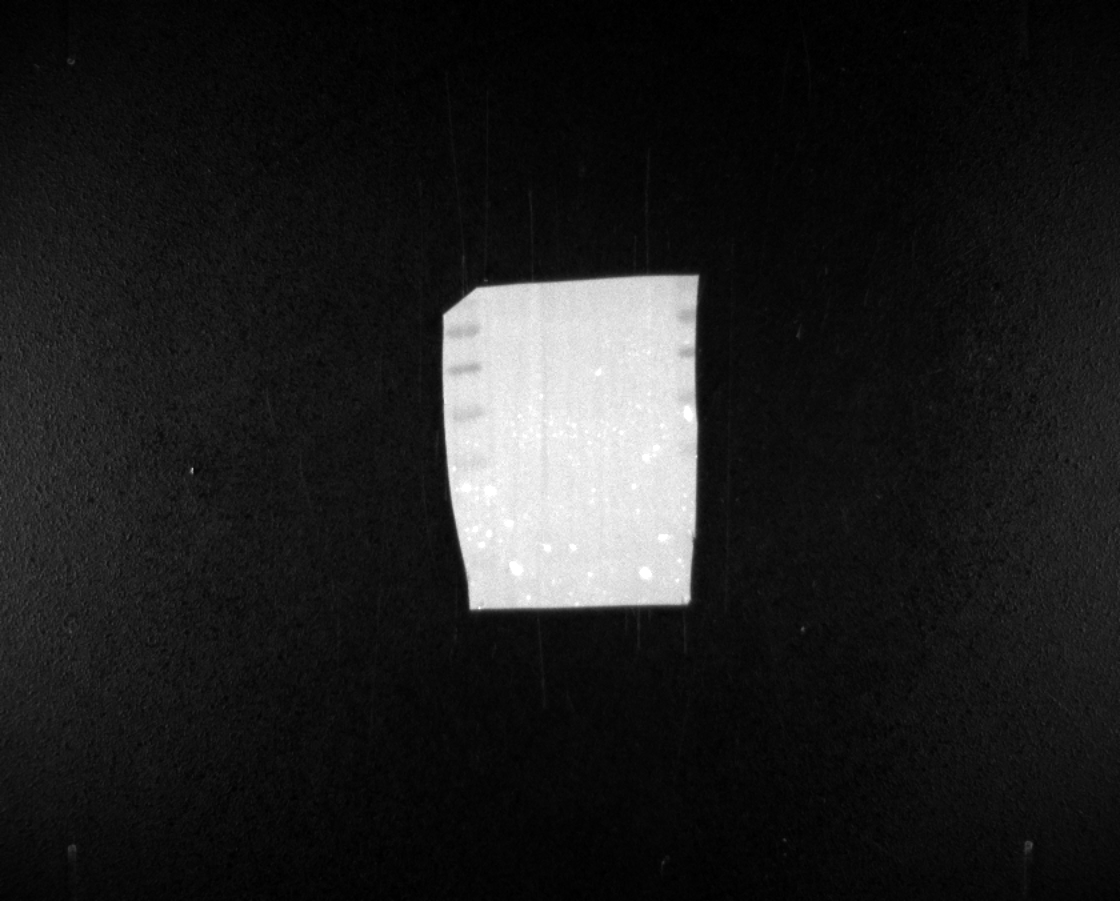

Supplement: Supplementary file 4 — Source Data [file 41467_2023_41520_MOESM4_ESM.zip › Source Data/Uncropped and Unprocessed Scans/Fig. 3a/IP CFL1 (mouse); IB CFL1 (rabbit) - Marker.tif]

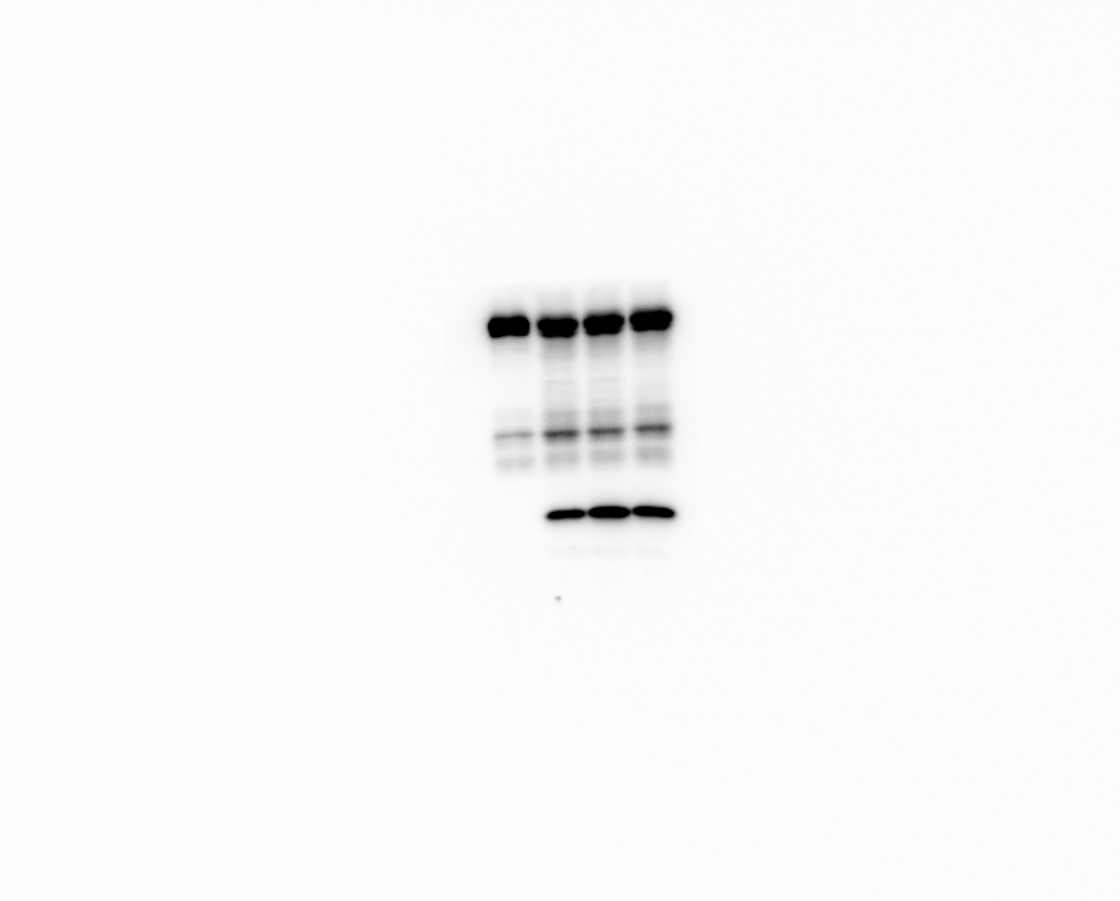

Supplement: Supplementary file 4 — Source Data [file 41467_2023_41520_MOESM4_ESM.zip › Source Data/Uncropped and Unprocessed Scans/Fig. 3a/IP CFL1 (mouse); IB CFL1 (rabbit).tif]

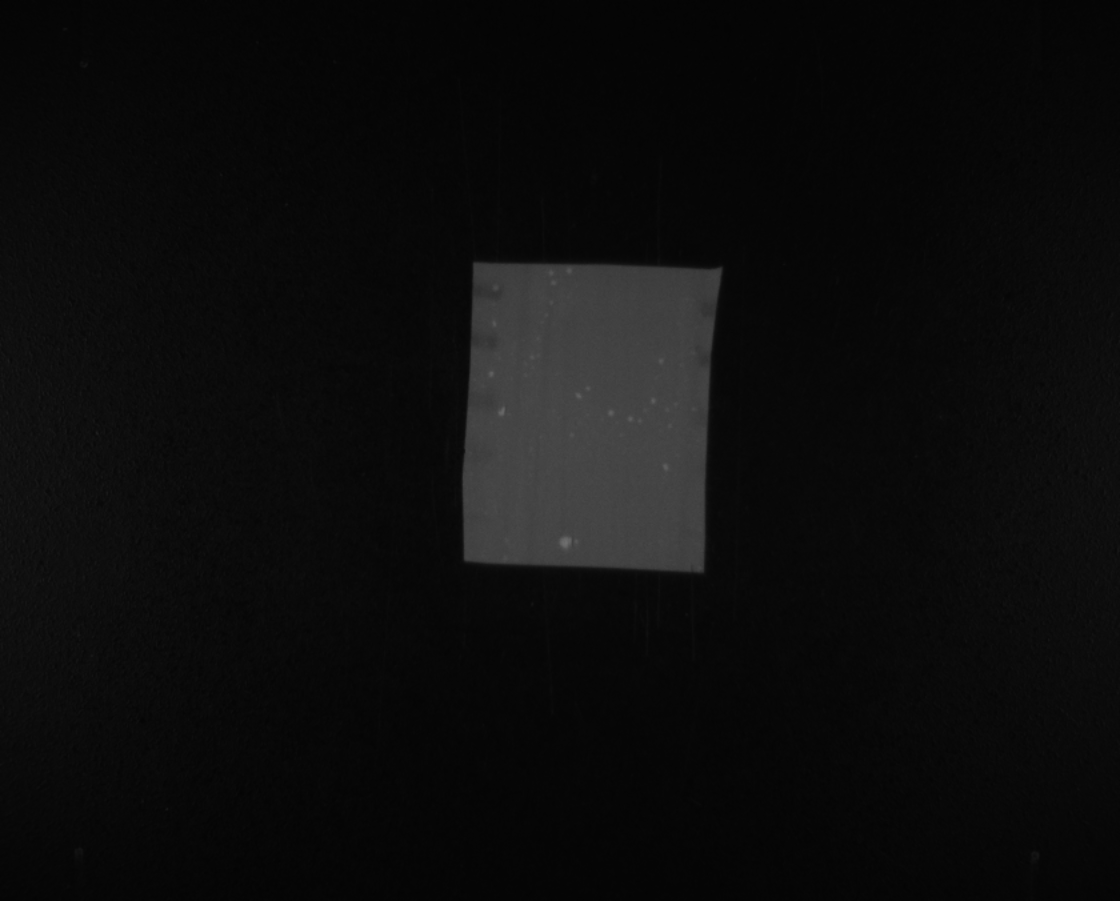

Supplement: Supplementary file 4 — Source Data [file 41467_2023_41520_MOESM4_ESM.zip › Source Data/Uncropped and Unprocessed Scans/Fig. 3a/IP CFL1 (mouse); IB SUMO1 (rabbit) - Marker.tif]

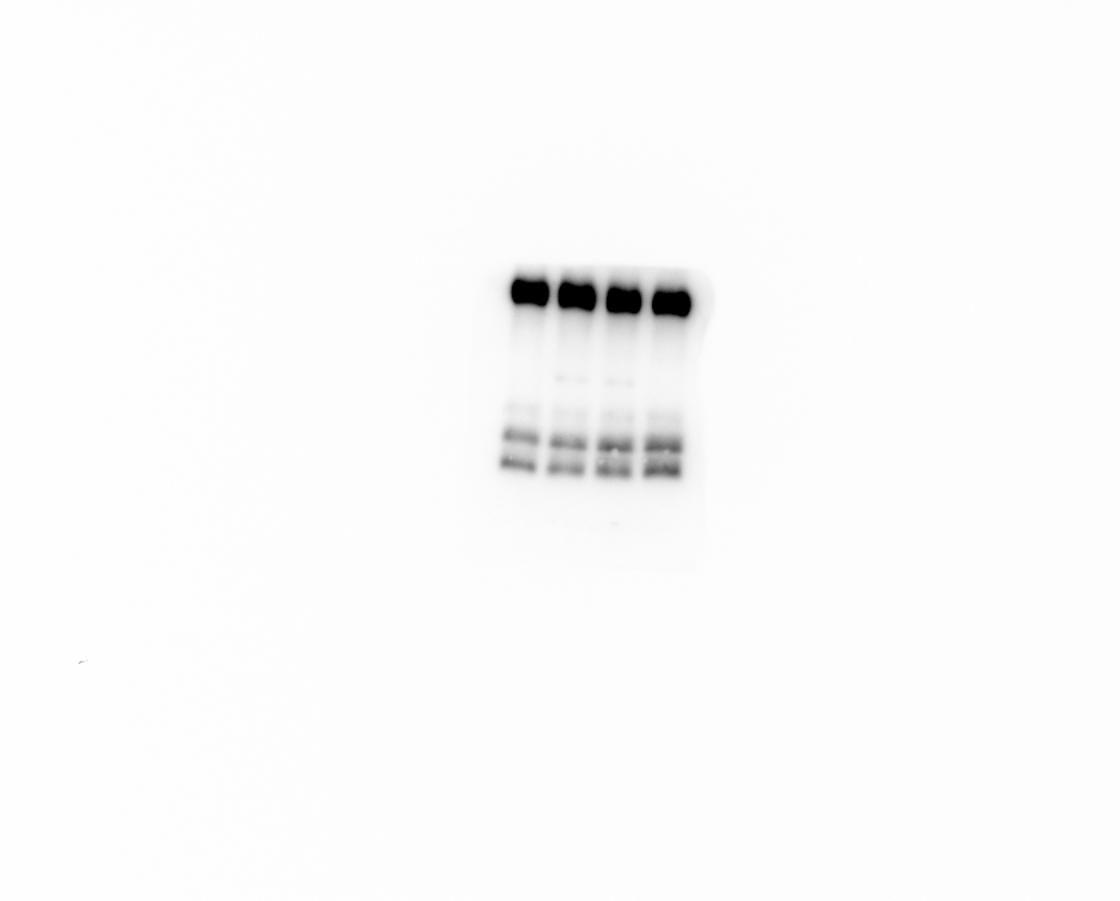

Supplement: Supplementary file 4 — Source Data [file 41467_2023_41520_MOESM4_ESM.zip › Source Data/Uncropped and Unprocessed Scans/Fig. 3a/IP CFL1 (mouse); IB SUMO1 (rabbit).tif]

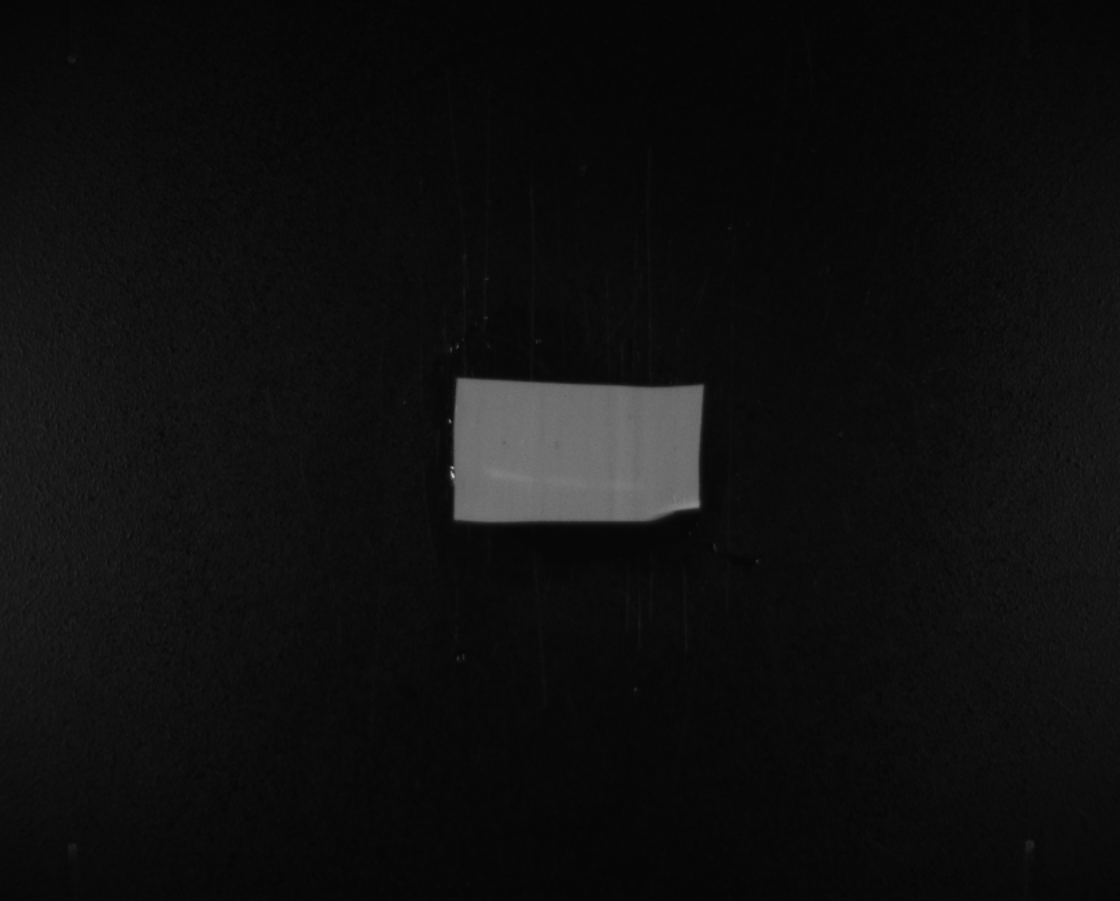

Supplement: Supplementary file 4 — Source Data [file 41467_2023_41520_MOESM4_ESM.zip › Source Data/Uncropped and Unprocessed Scans/Fig. 3b/IB CFL1 (rabbit) - Marker.tif]

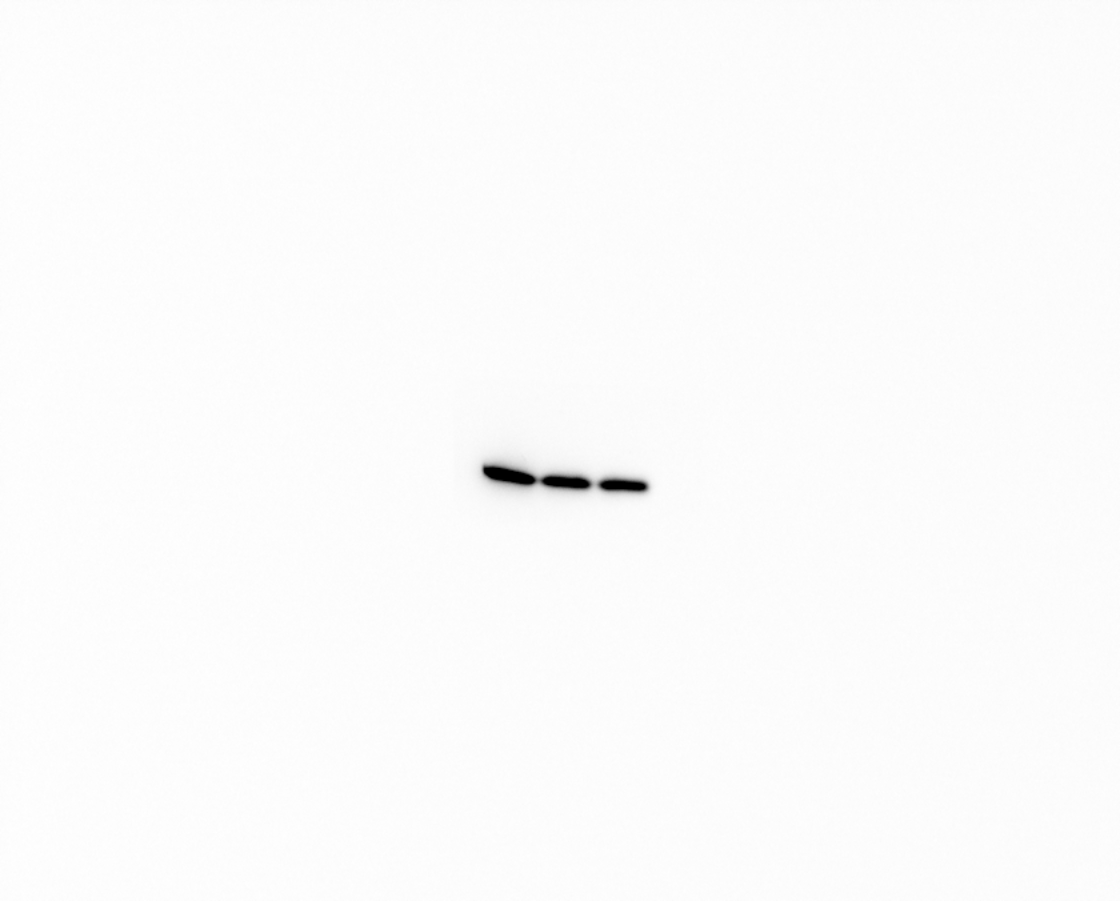

Supplement: Supplementary file 4 — Source Data [file 41467_2023_41520_MOESM4_ESM.zip › Source Data/Uncropped and Unprocessed Scans/Fig. 3b/IB CFL1 (rabbit).tif]

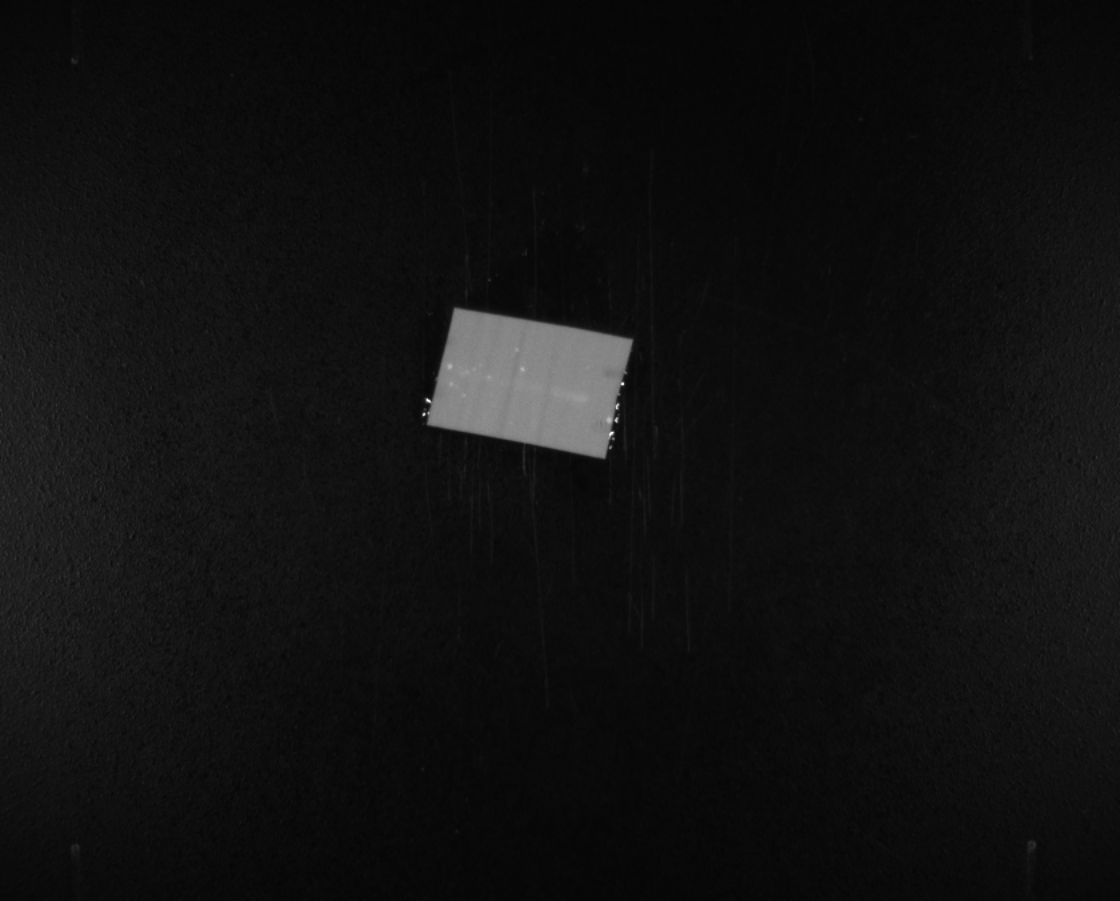

Supplement: Supplementary file 4 — Source Data [file 41467_2023_41520_MOESM4_ESM.zip › Source Data/Uncropped and Unprocessed Scans/Fig. 3b/IB GAPDH - Marker.tif]

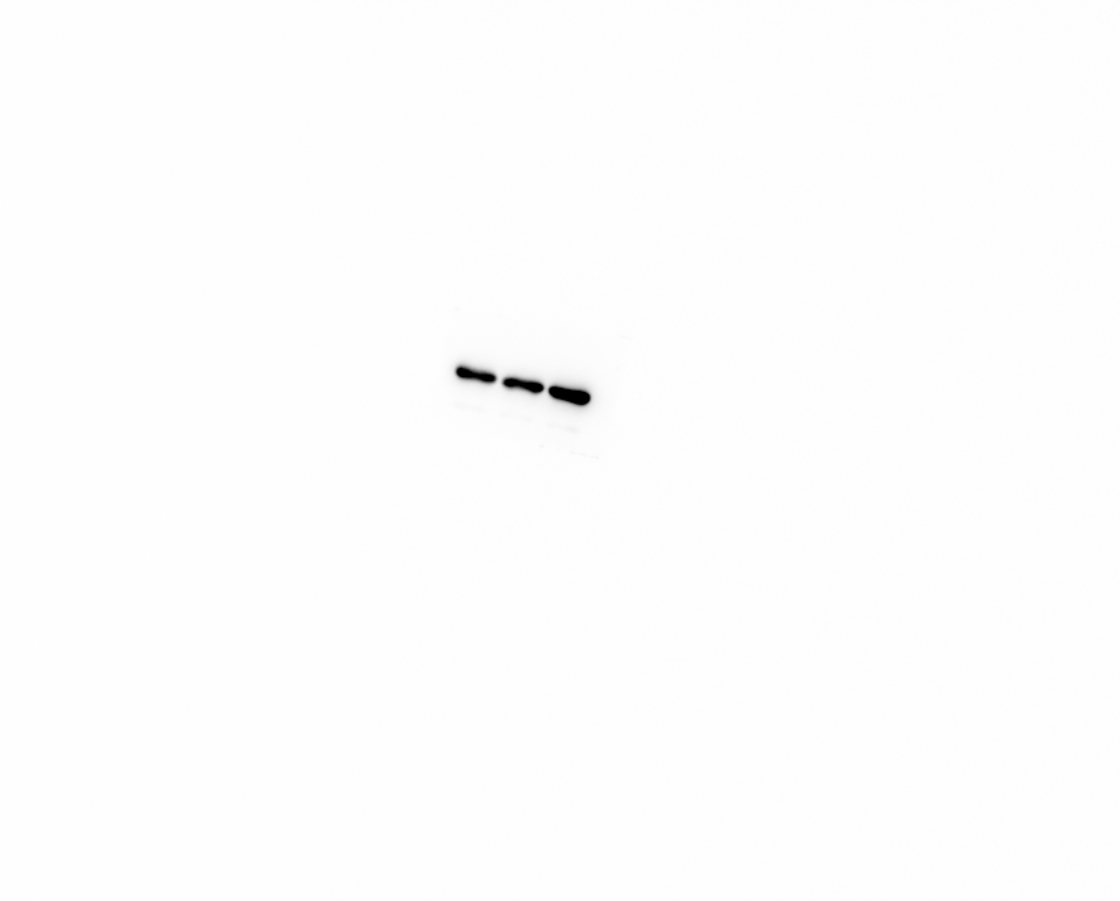

Supplement: Supplementary file 4 — Source Data [file 41467_2023_41520_MOESM4_ESM.zip › Source Data/Uncropped and Unprocessed Scans/Fig. 3b/IB GAPDH.tif]

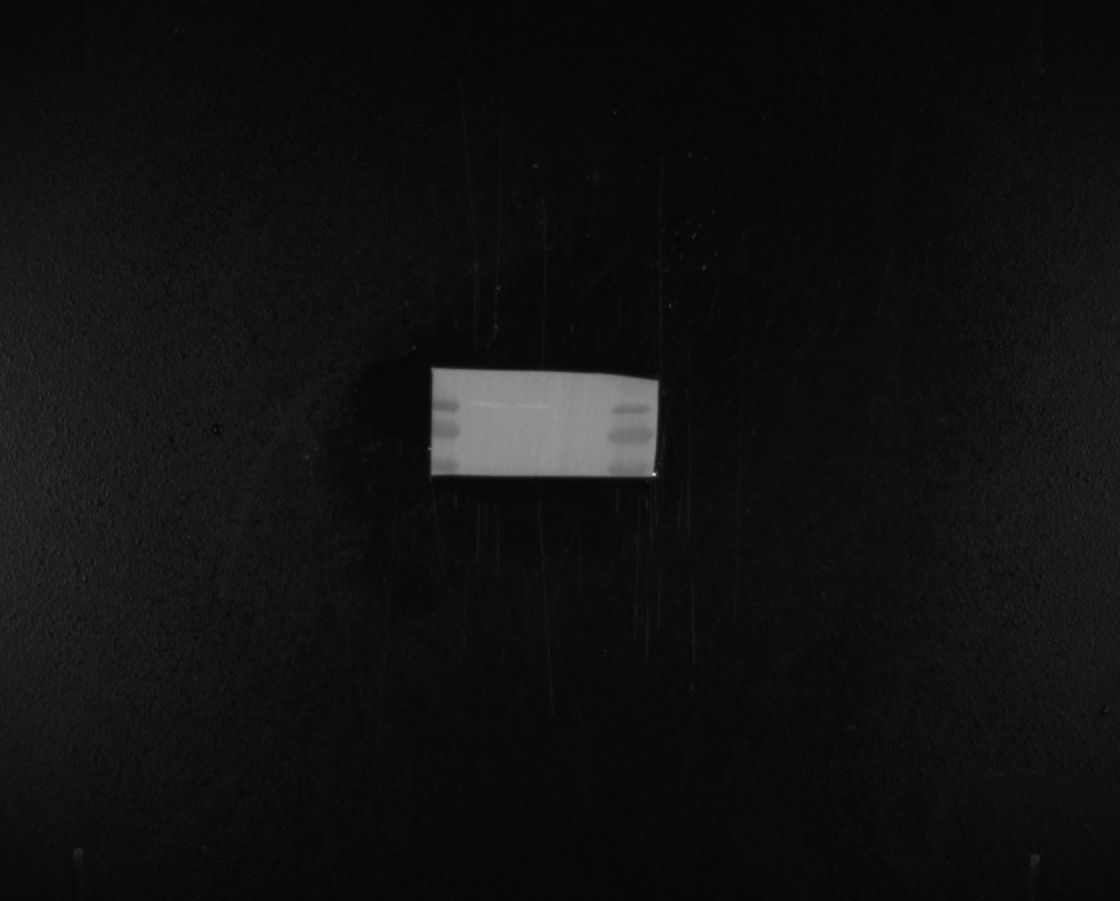

Supplement: Supplementary file 4 — Source Data [file 41467_2023_41520_MOESM4_ESM.zip › Source Data/Uncropped and Unprocessed Scans/Fig. 3b/IB SAE2 - Marker.tif]

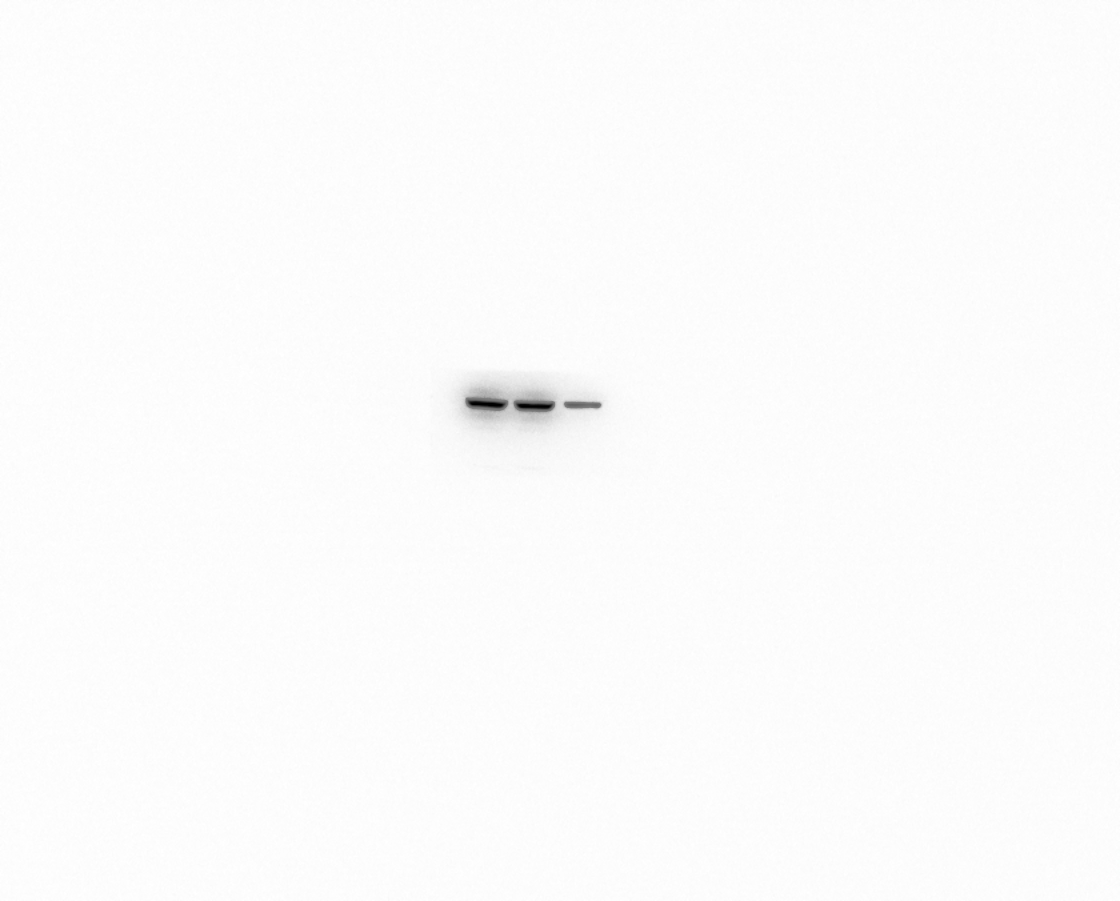

Supplement: Supplementary file 4 — Source Data [file 41467_2023_41520_MOESM4_ESM.zip › Source Data/Uncropped and Unprocessed Scans/Fig. 3b/IB SAE2.tif]

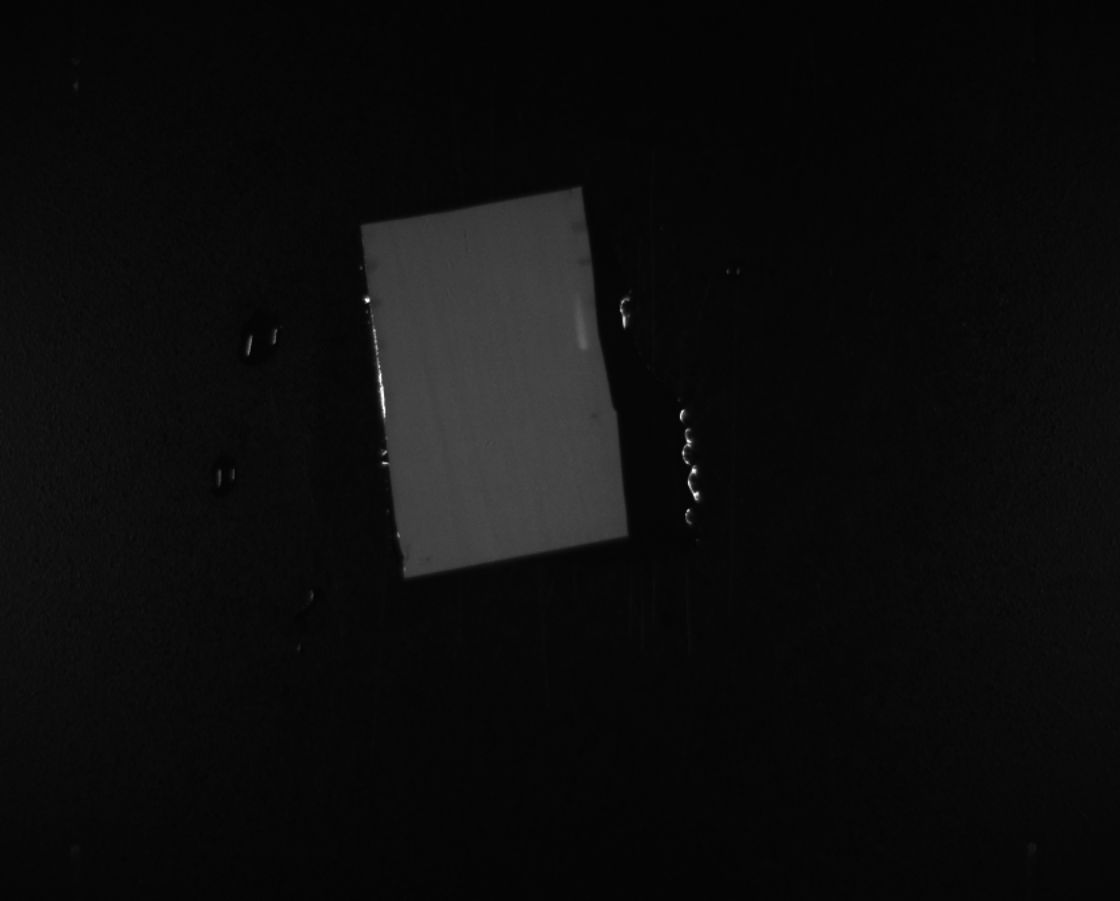

Supplement: Supplementary file 4 — Source Data [file 41467_2023_41520_MOESM4_ESM.zip › Source Data/Uncropped and Unprocessed Scans/Fig. 3b/IP CFL1 (mouse); IB CFL1 (rabbit) - Marker.tif]

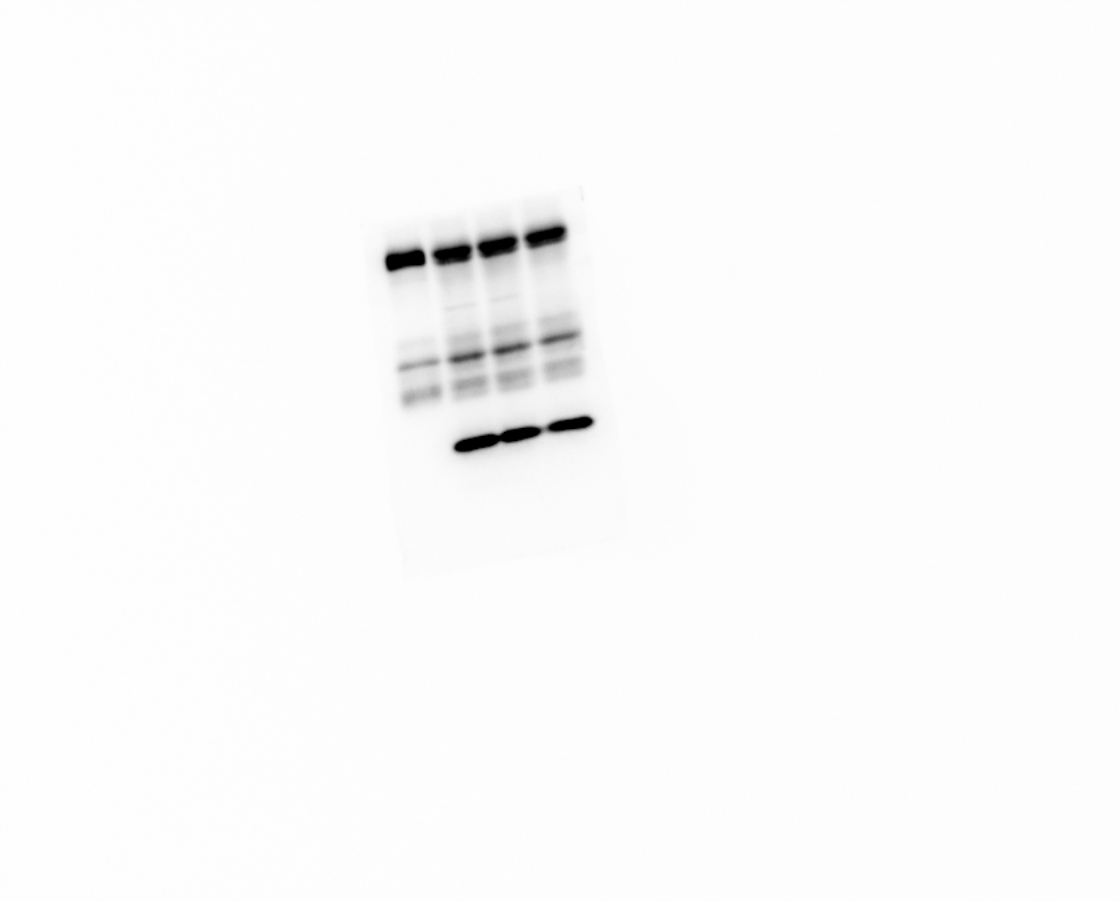

Supplement: Supplementary file 4 — Source Data [file 41467_2023_41520_MOESM4_ESM.zip › Source Data/Uncropped and Unprocessed Scans/Fig. 3b/IP CFL1 (mouse); IB CFL1 (rabbit).tif]

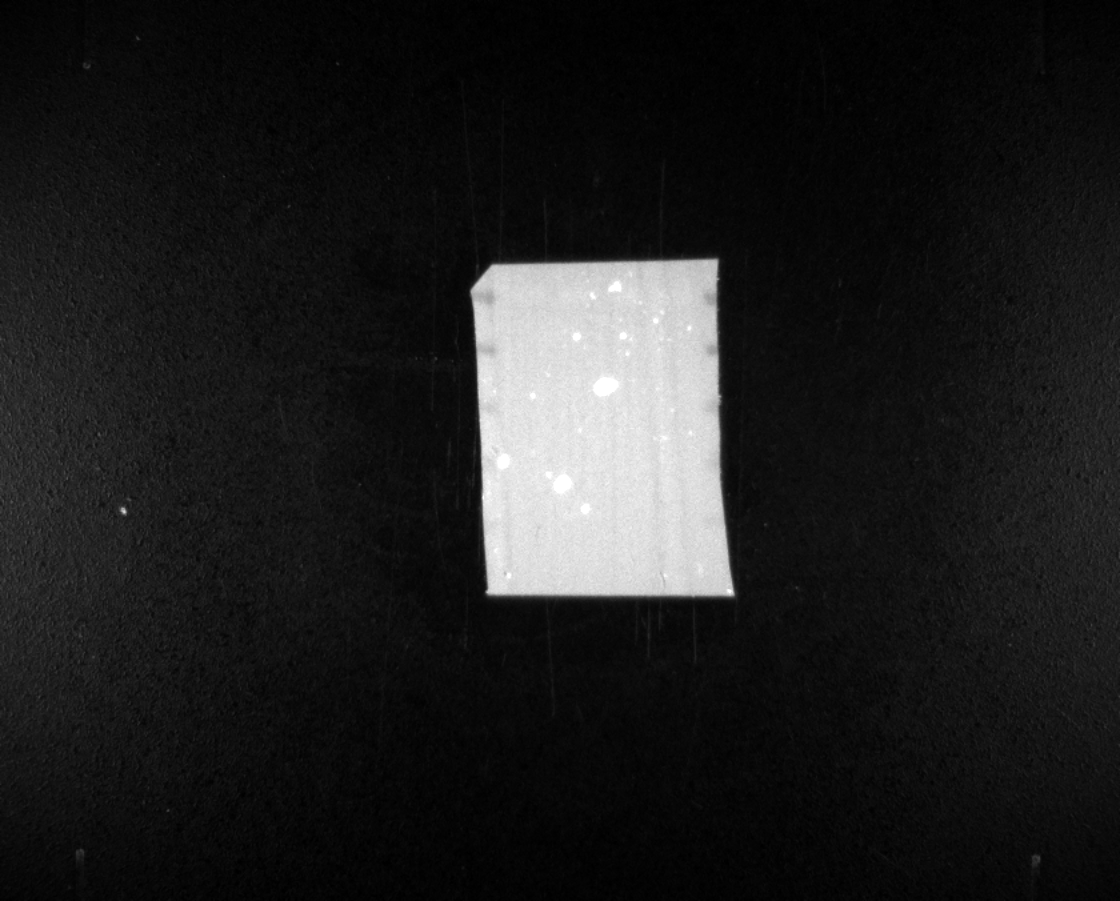

Supplement: Supplementary file 4 — Source Data [file 41467_2023_41520_MOESM4_ESM.zip › Source Data/Uncropped and Unprocessed Scans/Fig. 3b/IP CFL1 (mouse); IB SUMO1 - Marker.tif]

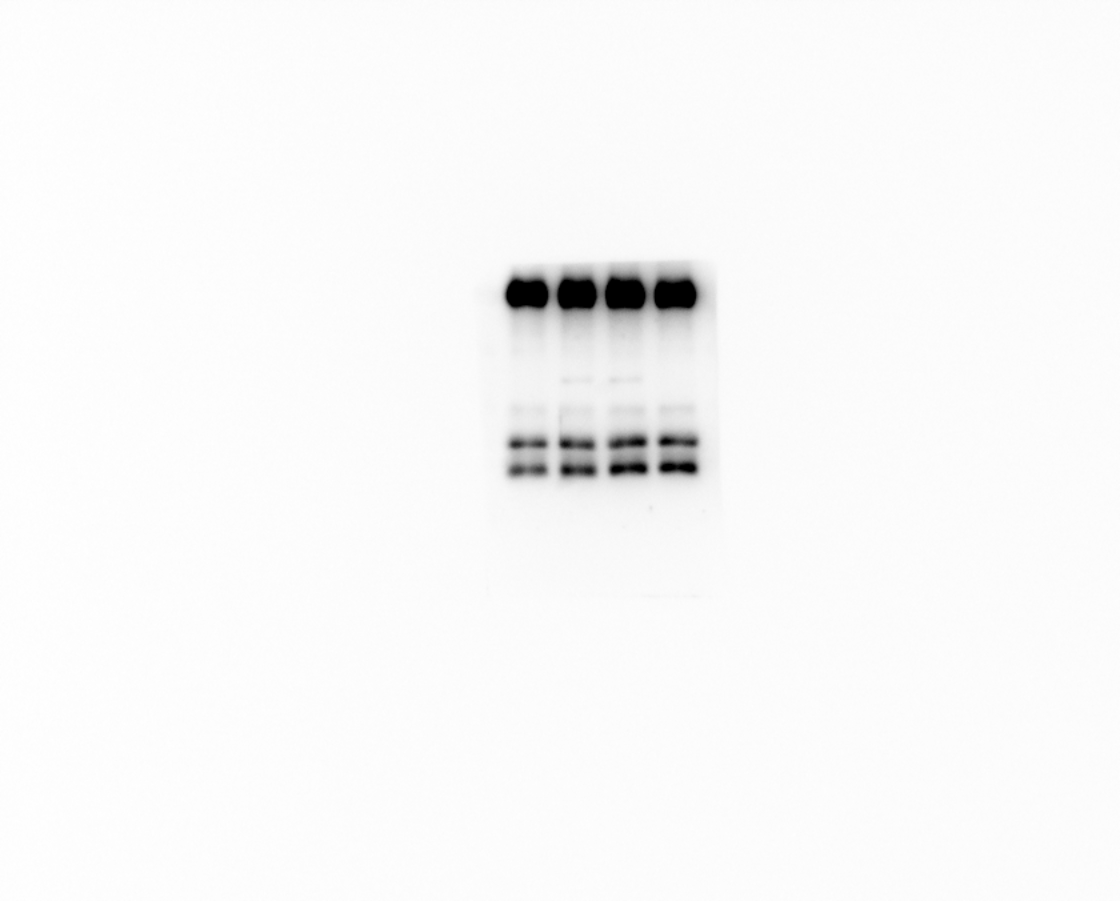

Supplement: Supplementary file 4 — Source Data [file 41467_2023_41520_MOESM4_ESM.zip › Source Data/Uncropped and Unprocessed Scans/Fig. 3b/IP CFL1 (mouse); IB SUMO1.tif]

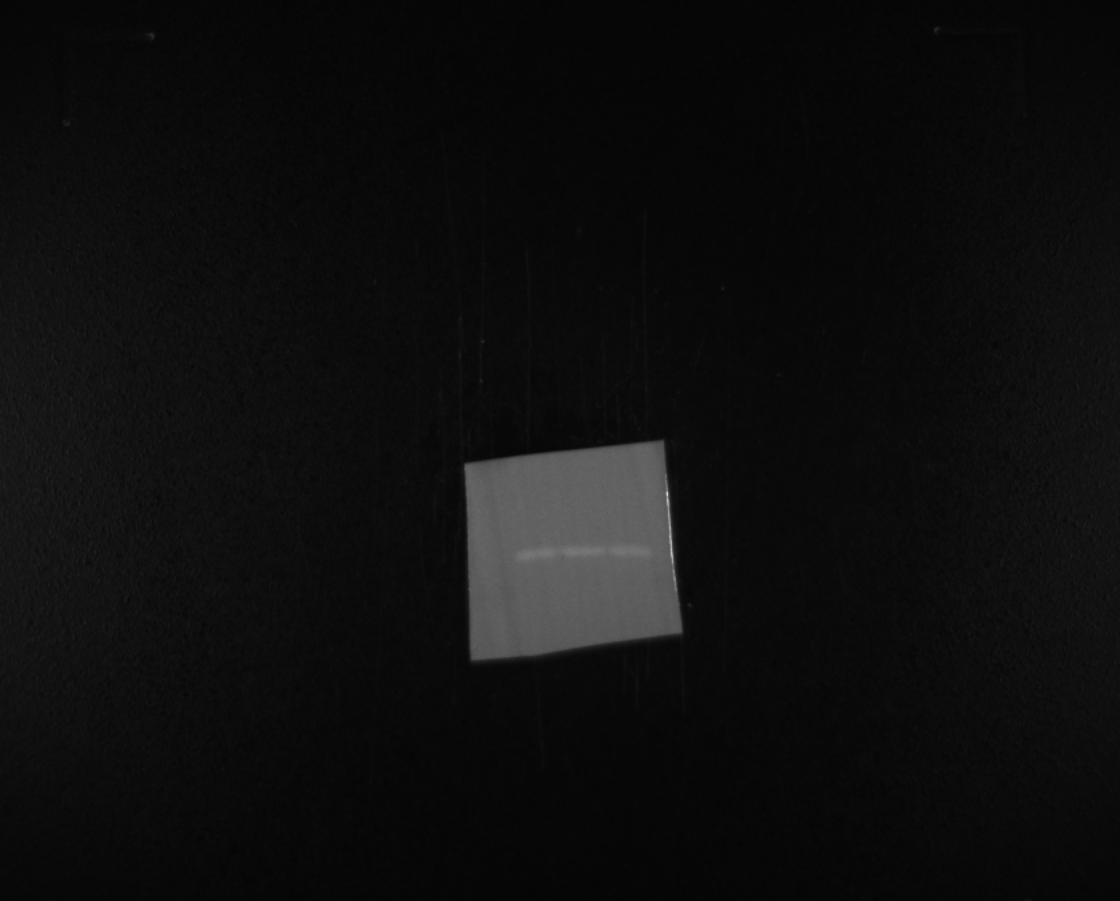

Supplement: Supplementary file 4 — Source Data [file 41467_2023_41520_MOESM4_ESM.zip › Source Data/Uncropped and Unprocessed Scans/Fig. 3c/IB CFL1 (rabbit) - Marker.tif]

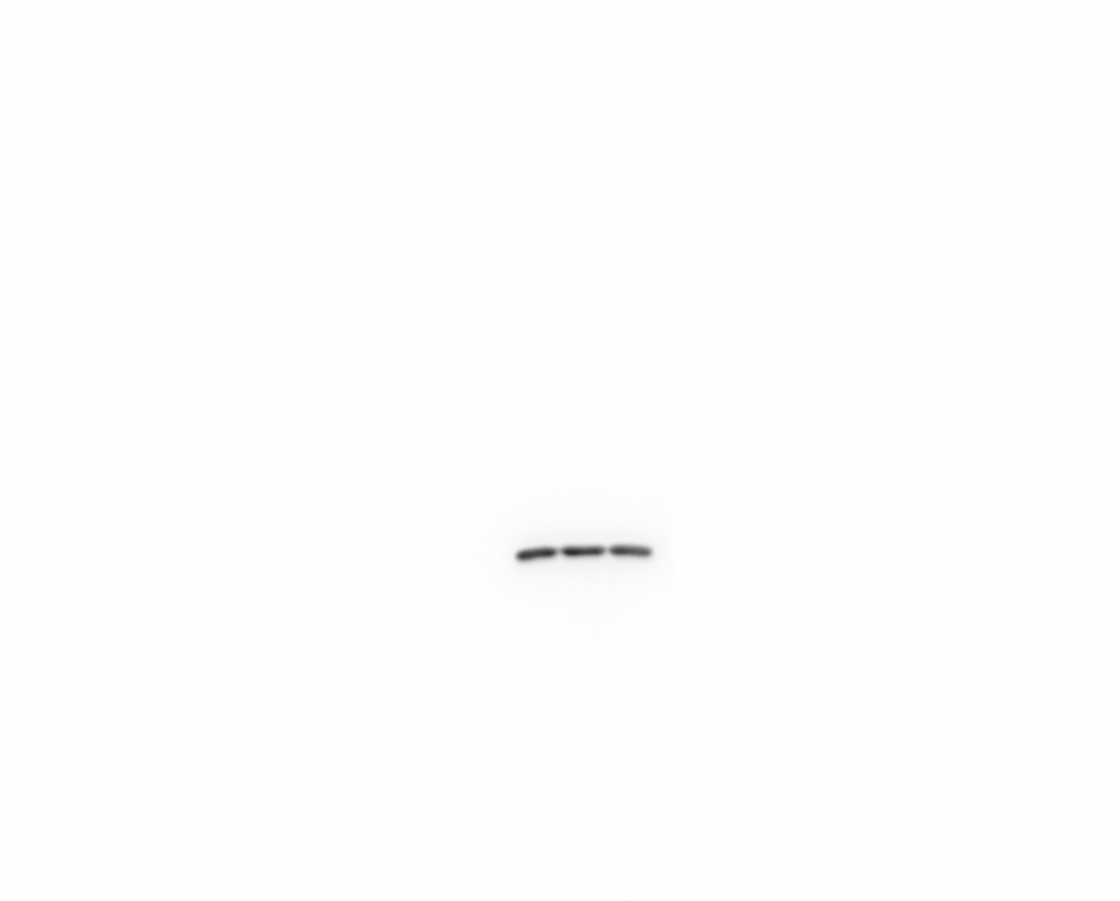

Supplement: Supplementary file 4 — Source Data [file 41467_2023_41520_MOESM4_ESM.zip › Source Data/Uncropped and Unprocessed Scans/Fig. 3c/IB CFL1 (rabbit).tif]

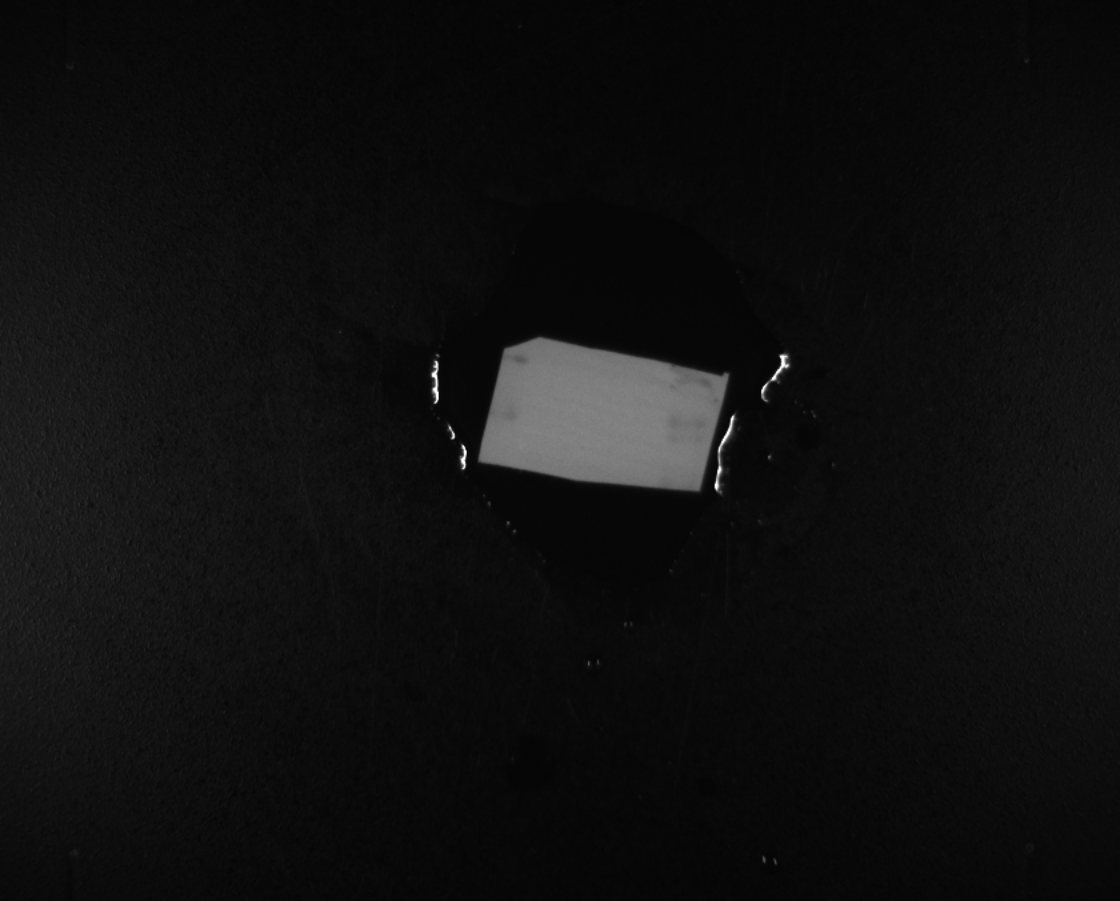

Supplement: Supplementary file 4 — Source Data [file 41467_2023_41520_MOESM4_ESM.zip › Source Data/Uncropped and Unprocessed Scans/Fig. 3c/IB GAPDH - Marker.tif]

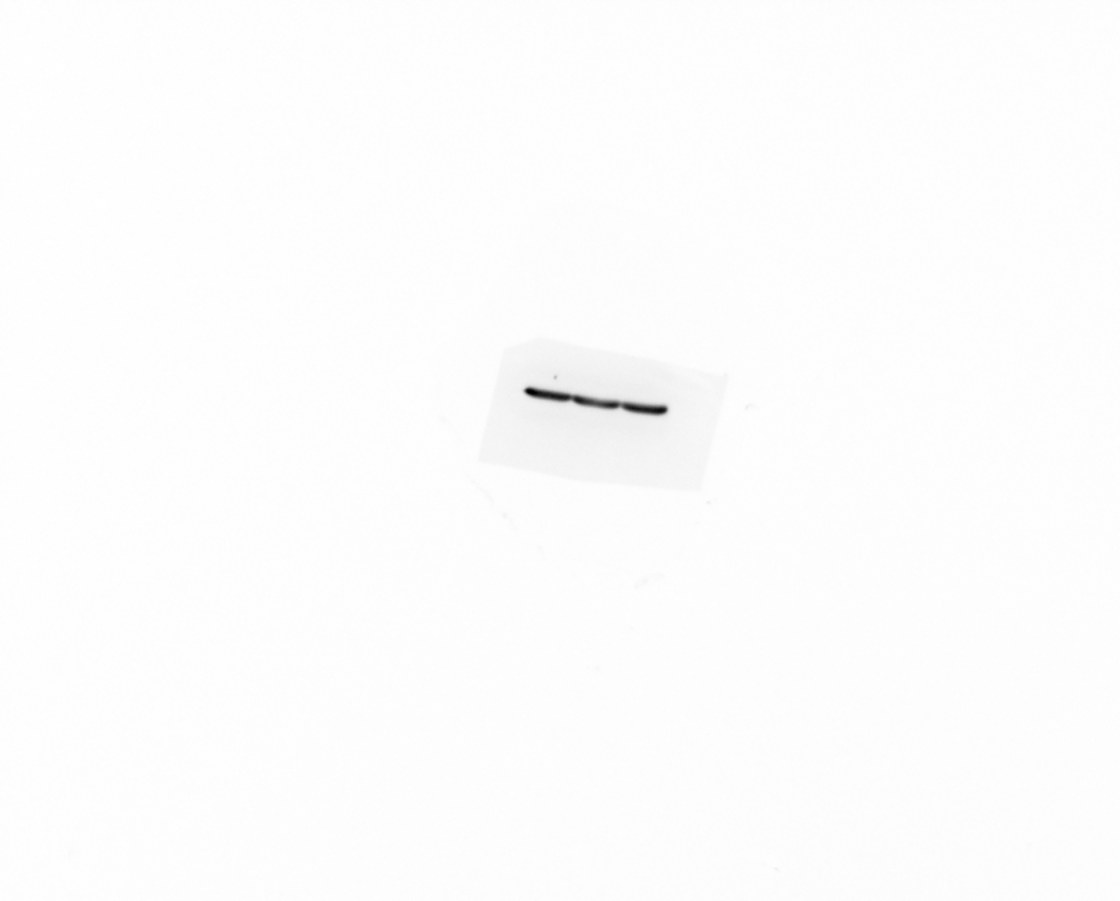

Supplement: Supplementary file 4 — Source Data [file 41467_2023_41520_MOESM4_ESM.zip › Source Data/Uncropped and Unprocessed Scans/Fig. 3c/IB GAPDH.tif]

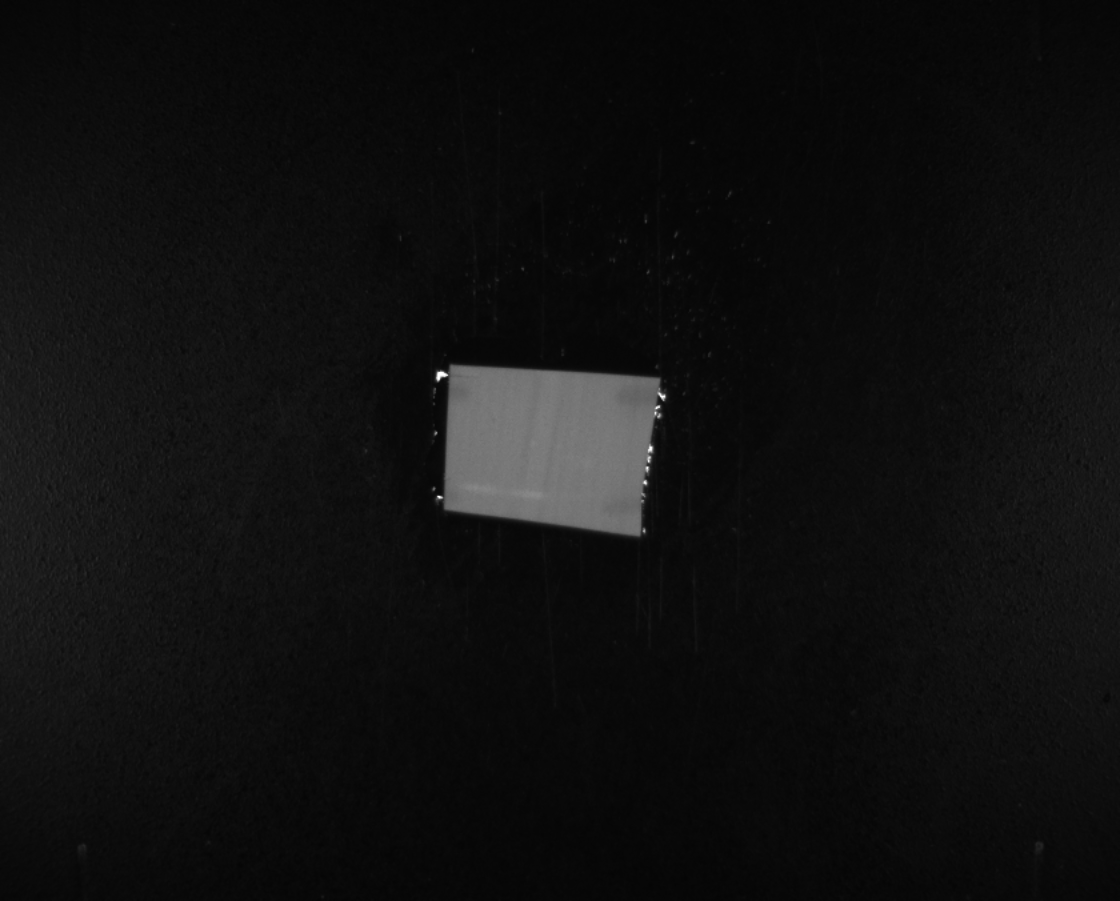

Supplement: Supplementary file 4 — Source Data [file 41467_2023_41520_MOESM4_ESM.zip › Source Data/Uncropped and Unprocessed Scans/Fig. 3c/IB Ubc9 - Marker.tif]

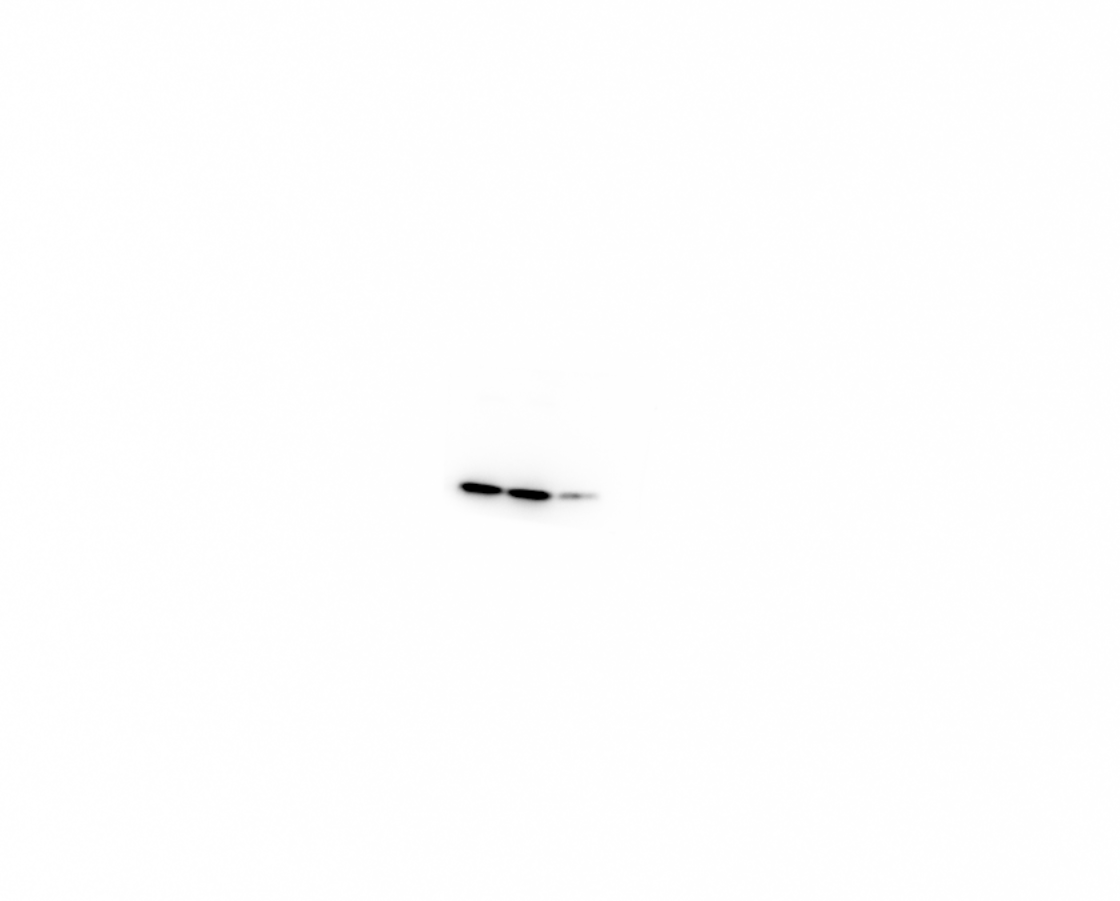

Supplement: Supplementary file 4 — Source Data [file 41467_2023_41520_MOESM4_ESM.zip › Source Data/Uncropped and Unprocessed Scans/Fig. 3c/IB Ubc9.tif]

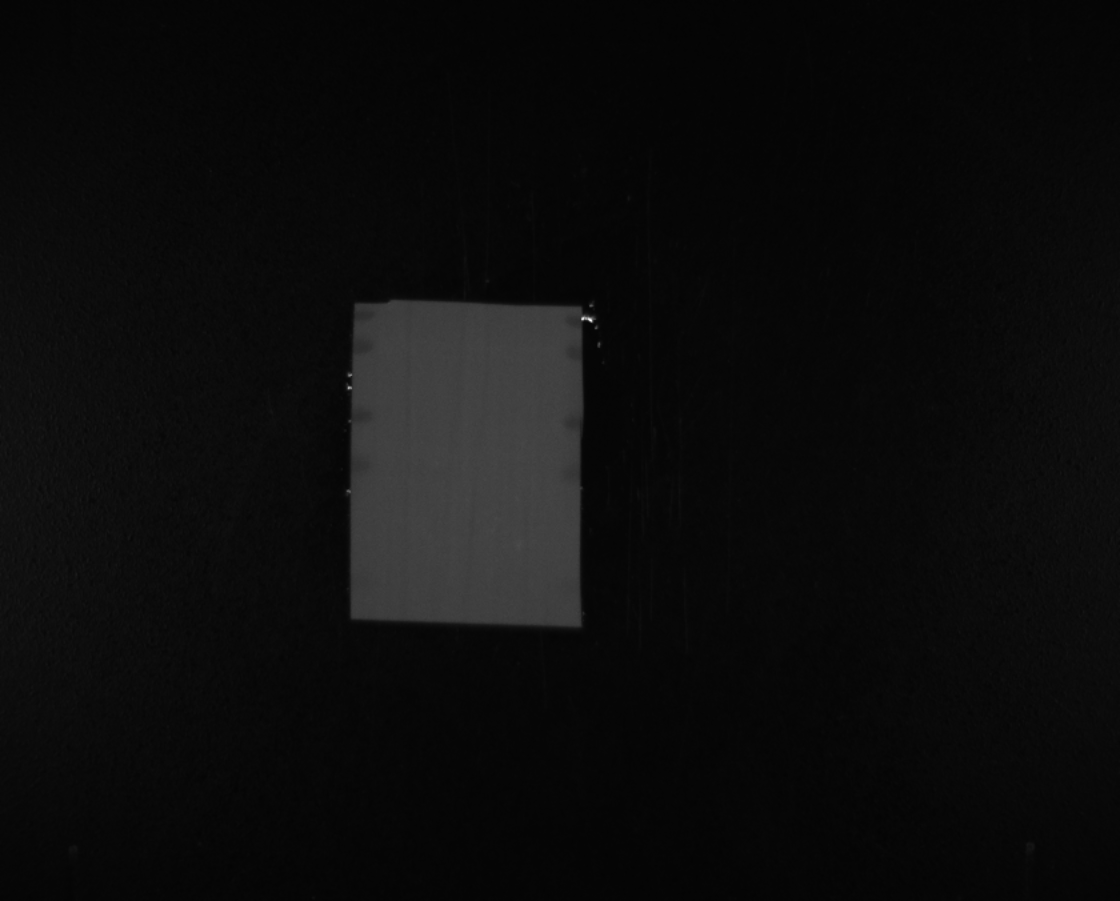

Supplement: Supplementary file 4 — Source Data [file 41467_2023_41520_MOESM4_ESM.zip › Source Data/Uncropped and Unprocessed Scans/Fig. 3c/IP CFL1 (mouse); IB CFL1 (rabbit) - Marker.tif]

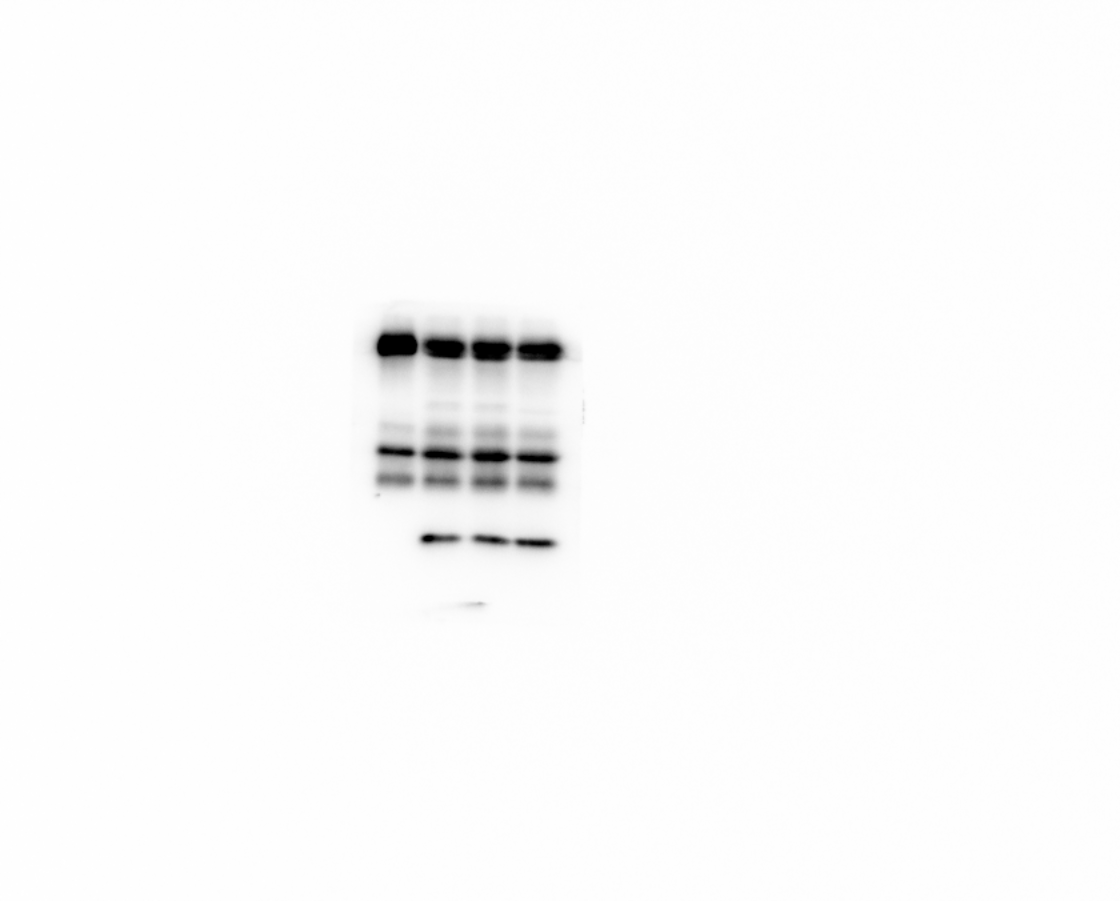

Supplement: Supplementary file 4 — Source Data [file 41467_2023_41520_MOESM4_ESM.zip › Source Data/Uncropped and Unprocessed Scans/Fig. 3c/IP CFL1 (mouse); IB CFL1 (rabbit).tif]

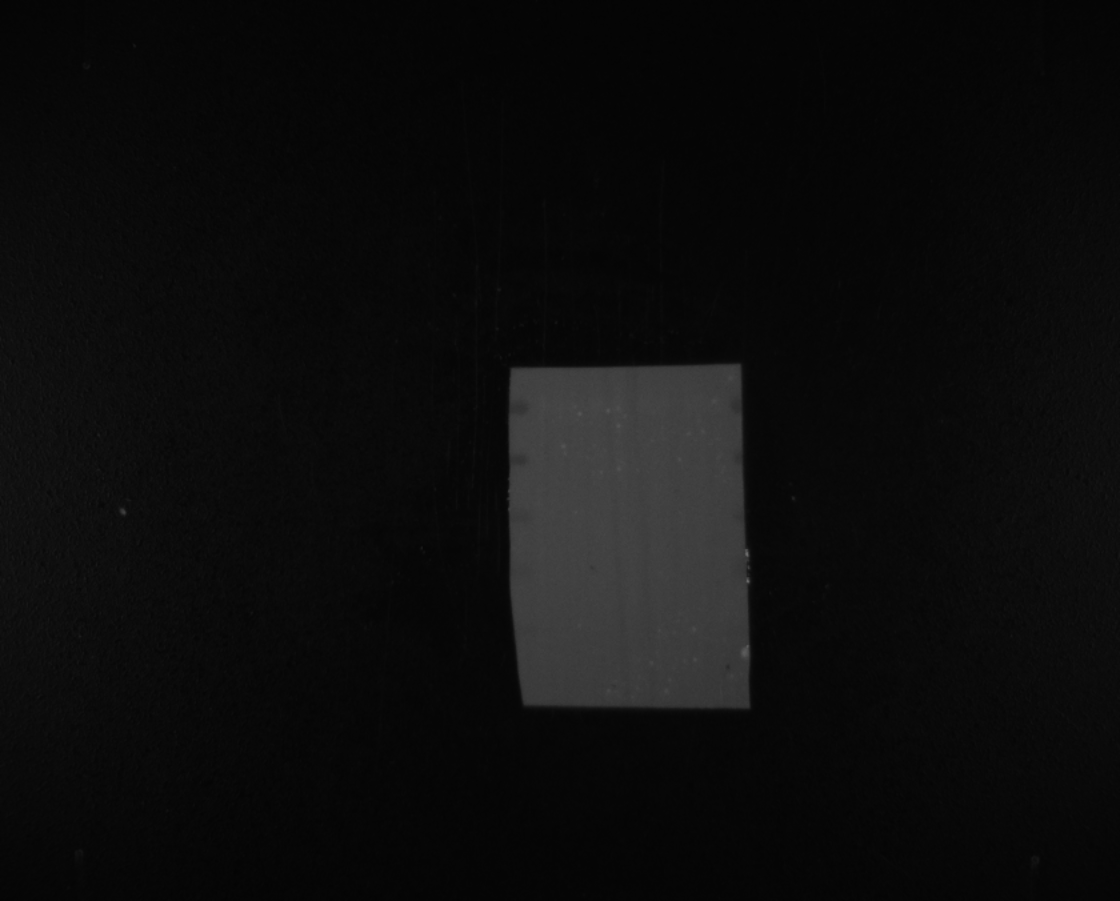

Supplement: Supplementary file 4 — Source Data [file 41467_2023_41520_MOESM4_ESM.zip › Source Data/Uncropped and Unprocessed Scans/Fig. 3c/IP CFL1 (mouse); IB SUMO1 (rabbit) - Marker.tif]

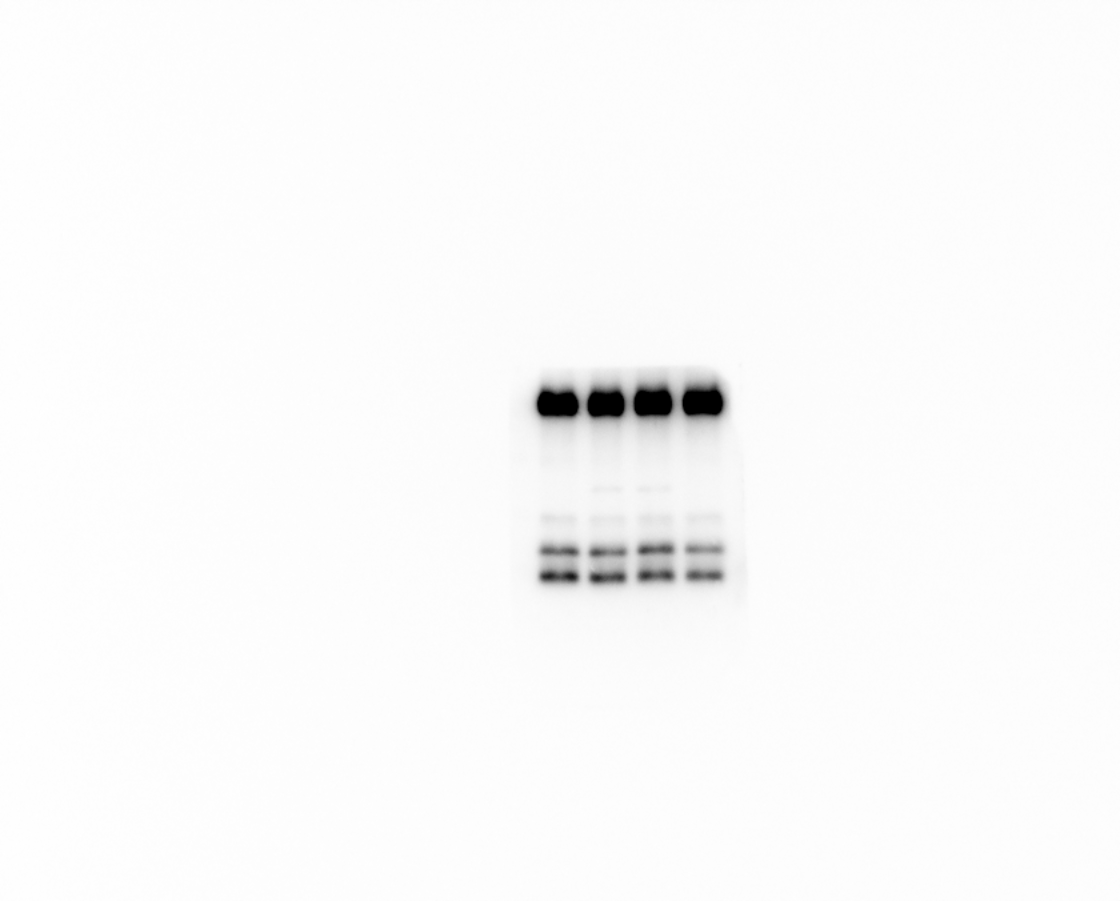

Supplement: Supplementary file 4 — Source Data [file 41467_2023_41520_MOESM4_ESM.zip › Source Data/Uncropped and Unprocessed Scans/Fig. 3c/IP CFL1 (mouse); IB SUMO1 (rabbit).tif]

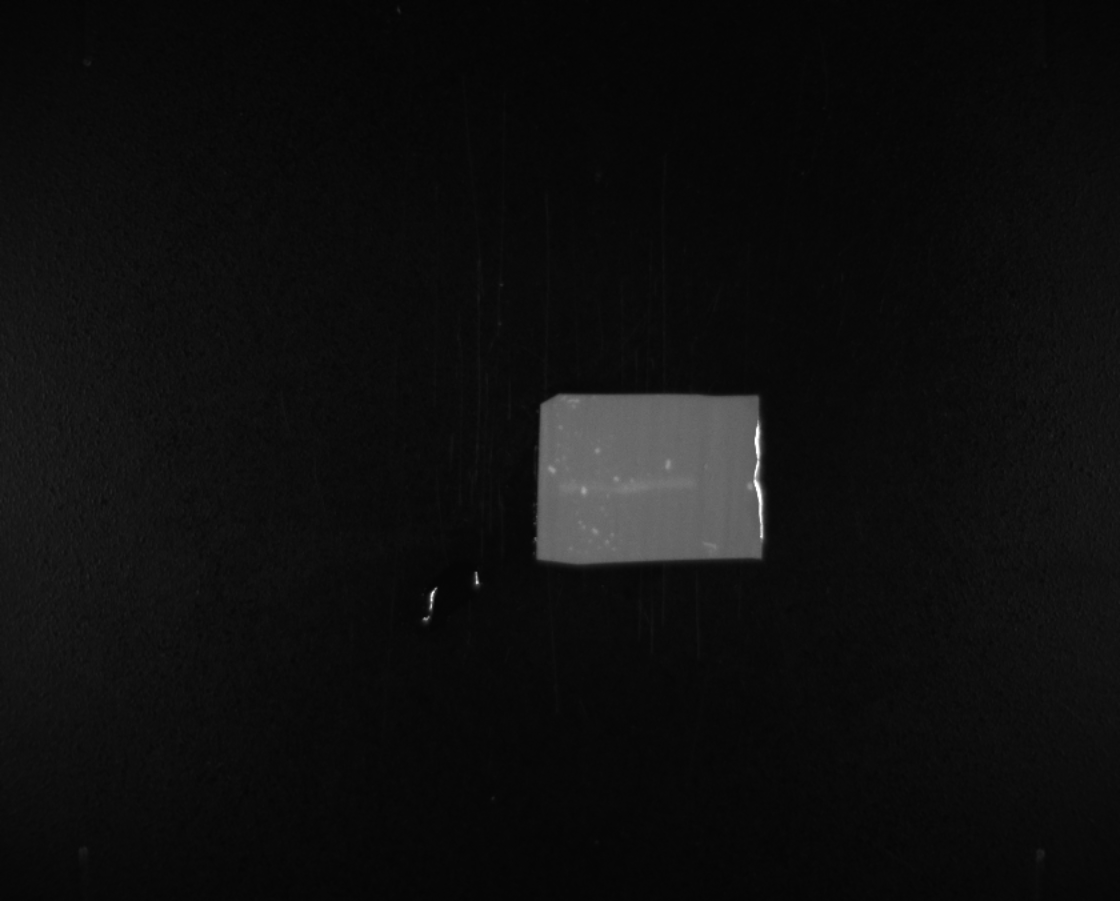

Supplement: Supplementary file 4 — Source Data [file 41467_2023_41520_MOESM4_ESM.zip › Source Data/Uncropped and Unprocessed Scans/Fig. 3d/IB CFL1 (rabbit) - Marker.tif]

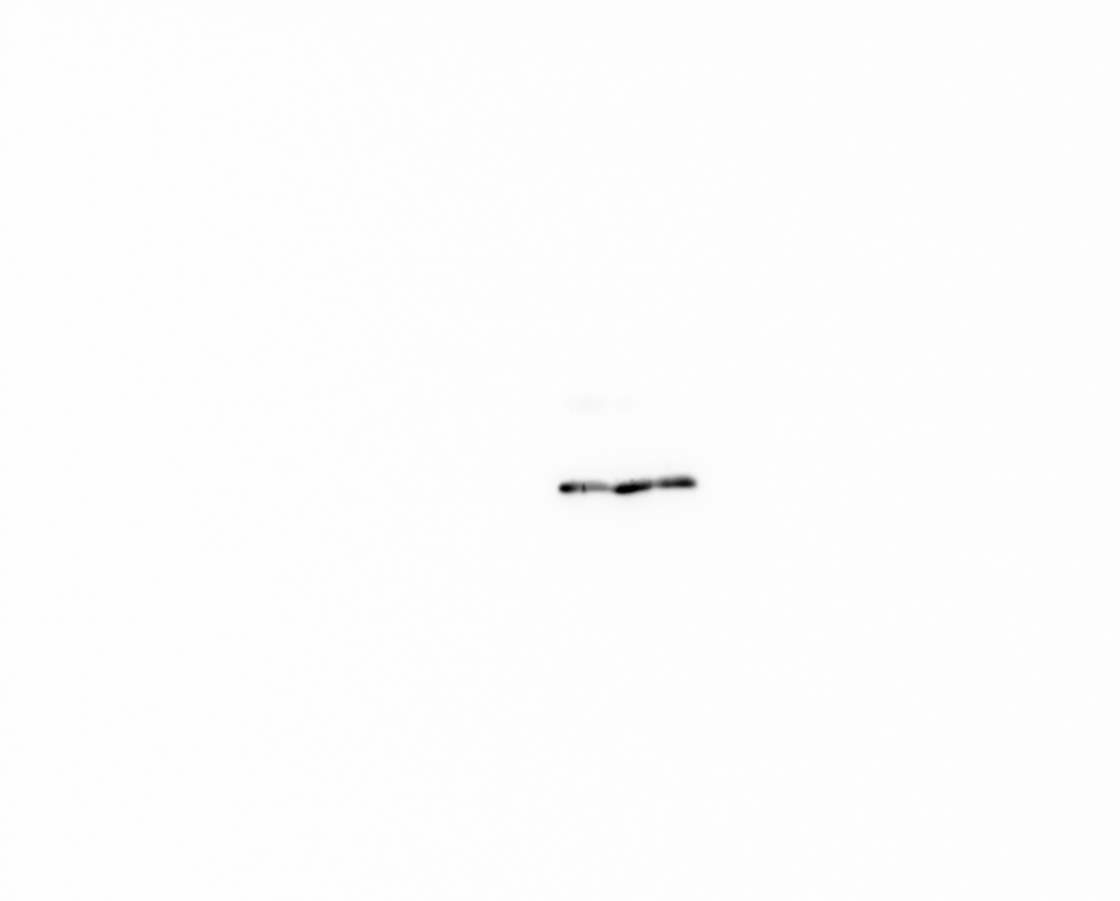

Supplement: Supplementary file 4 — Source Data [file 41467_2023_41520_MOESM4_ESM.zip › Source Data/Uncropped and Unprocessed Scans/Fig. 3d/IB CFL1 (rabbit).tif]

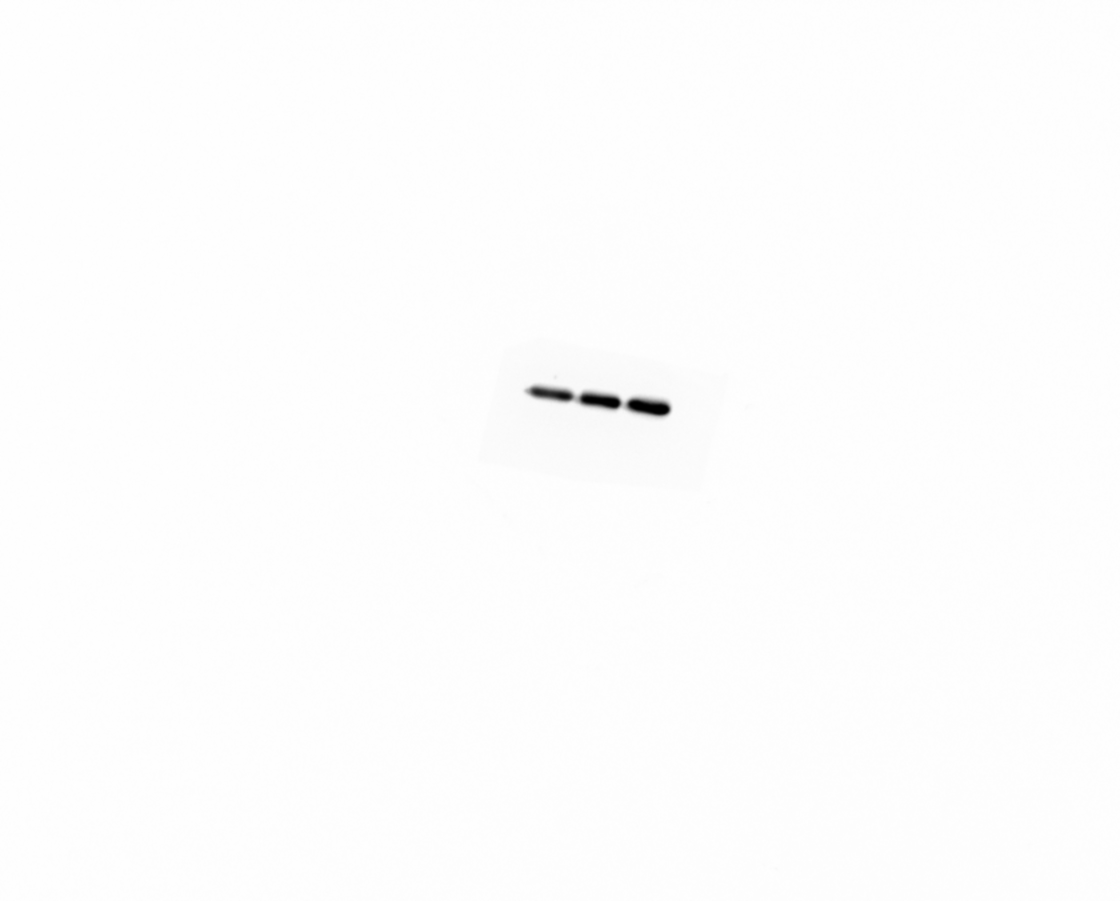

Supplement: Supplementary file 4 — Source Data [file 41467_2023_41520_MOESM4_ESM.zip › Source Data/Uncropped and Unprocessed Scans/Fig. 3d/IB GAPDH.tif]

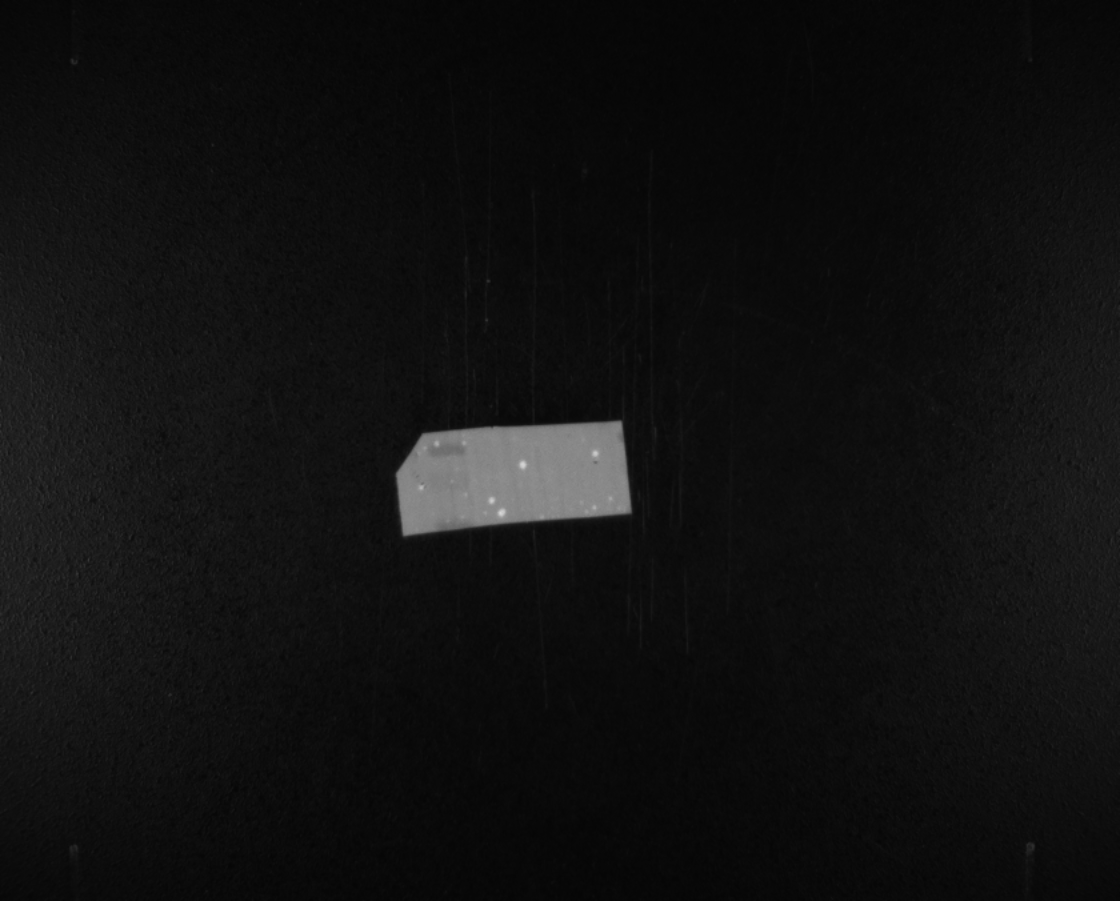

Supplement: Supplementary file 4 — Source Data [file 41467_2023_41520_MOESM4_ESM.zip › Source Data/Uncropped and Unprocessed Scans/Fig. 3d/IB SENP1 - Marker.tif]

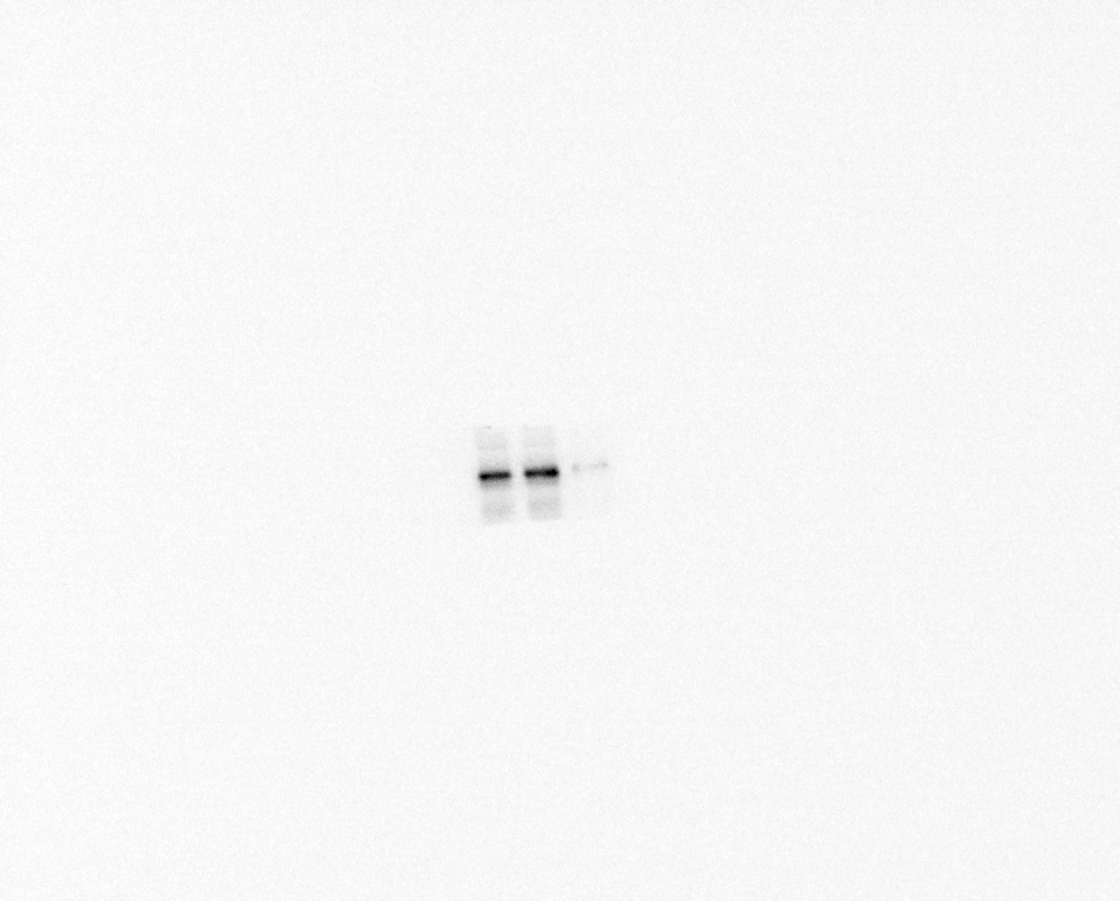

Supplement: Supplementary file 4 — Source Data [file 41467_2023_41520_MOESM4_ESM.zip › Source Data/Uncropped and Unprocessed Scans/Fig. 3d/IB SENP1.tif]

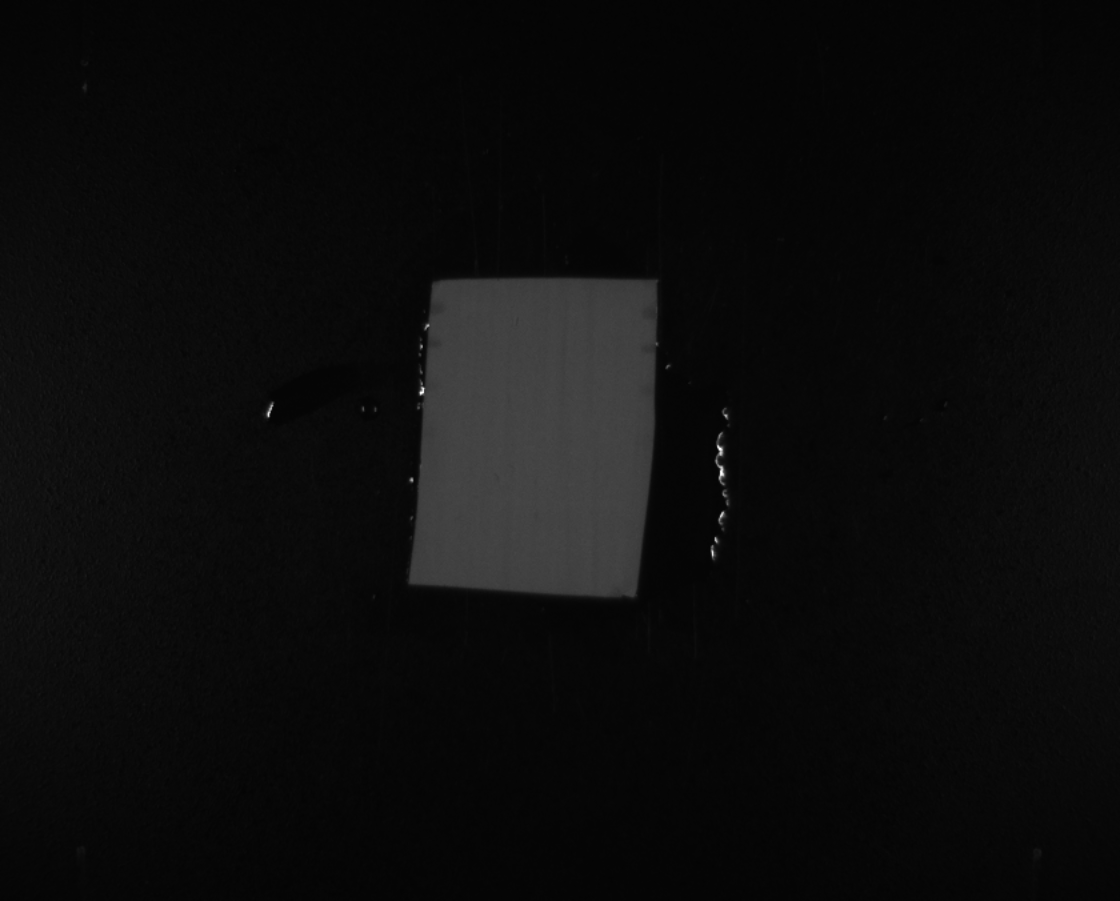

Supplement: Supplementary file 4 — Source Data [file 41467_2023_41520_MOESM4_ESM.zip › Source Data/Uncropped and Unprocessed Scans/Fig. 3d/IP CFL1 (mouse); IB CFL1 (rabbit) - Marker.tif]

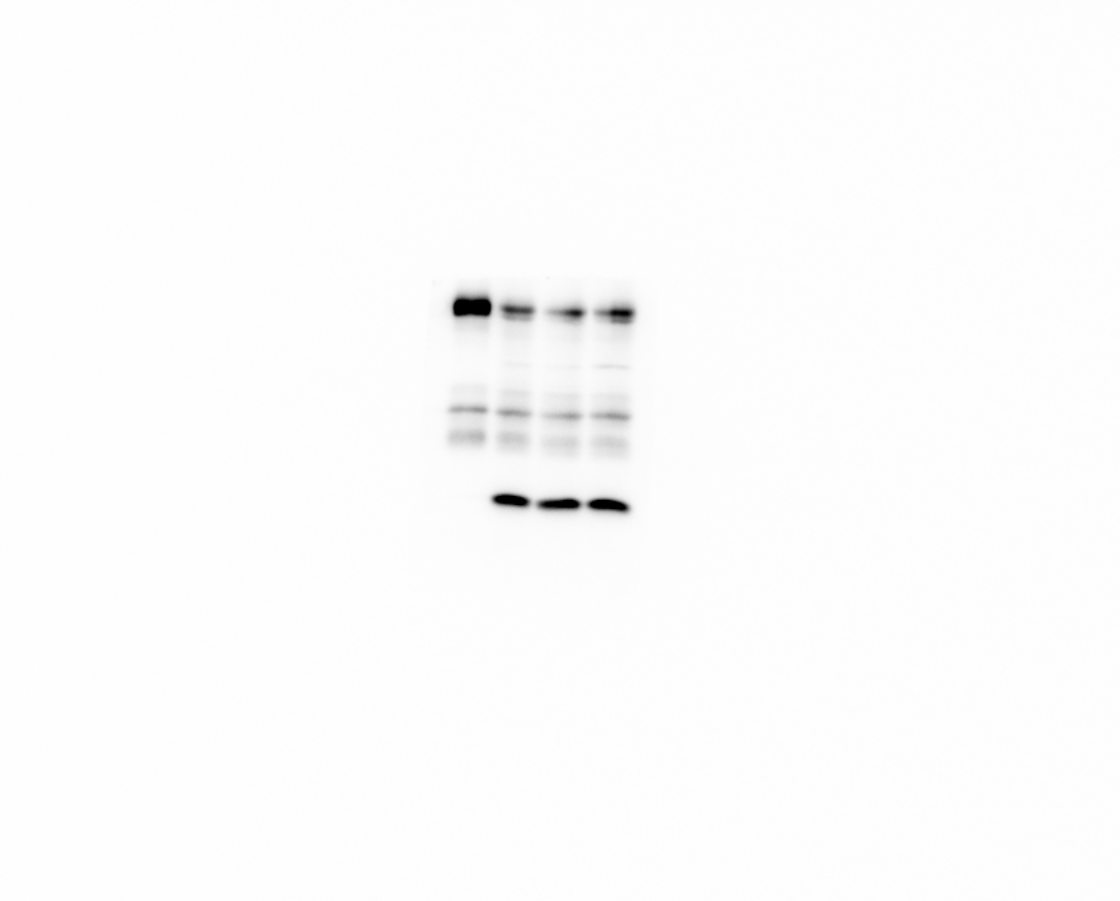

Supplement: Supplementary file 4 — Source Data [file 41467_2023_41520_MOESM4_ESM.zip › Source Data/Uncropped and Unprocessed Scans/Fig. 3d/IP CFL1 (mouse); IB CFL1 (rabbit).tif]

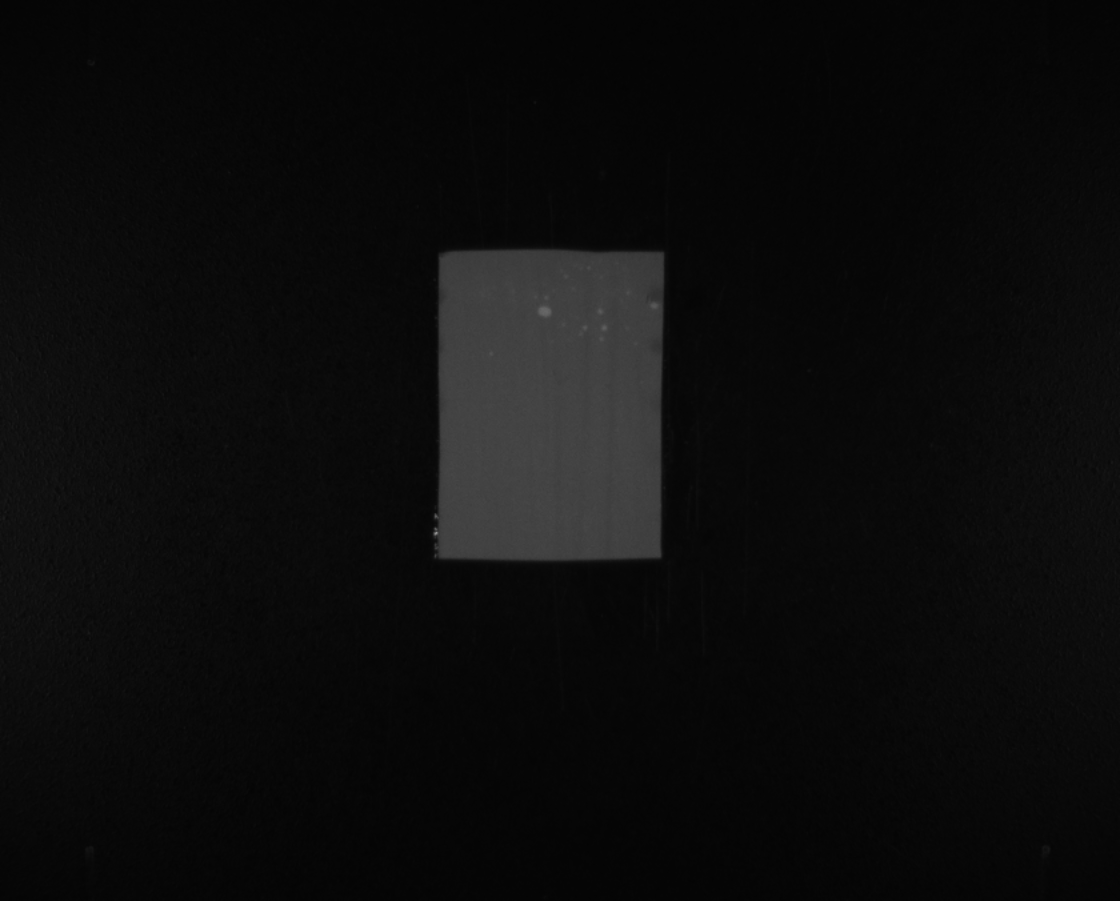

Supplement: Supplementary file 4 — Source Data [file 41467_2023_41520_MOESM4_ESM.zip › Source Data/Uncropped and Unprocessed Scans/Fig. 3d/IP CFL1 (mouse); IB SUMO1 (rabbit) - Marker.tif]
